# Characterization and Expression of the *Lucina pectinata* Oxygen and Sulfide Binding Hemoglobin Genes

**DOI:** 10.1371/journal.pone.0147977

**Published:** 2016-01-29

**Authors:** Ingrid M. Montes-Rodríguez, Linda E. Rivera, Juan López-Garriga, Carmen L. Cadilla

**Affiliations:** 1 Department of Chemistry, Arts and Sciences Faculty, University of Puerto Rico-Mayaguüez Campus, P.O. Box 9019, Mayagüez, Puerto Rico 00681–9019; 2 Department of Biochemistry, School of Medicine, University of Puerto Rico-Medical Sciences Campus, P.O. Box 365067, San Juan, Puerto Rico 00936–5067; University of the West of England, UNITED KINGDOM

## Abstract

The clam *Lucina pectinata* lives in sulfide-rich muds and houses intracellular symbiotic bacteria that need to be supplied with hydrogen sulfide and oxygen. This clam possesses three hemoglobins: hemoglobin I (HbI), a sulfide-reactive protein, and hemoglobin II (HbII) and III (HbIII), which are oxygen-reactive. We characterized the complete gene sequence and promoter regions for the oxygen reactive hemoglobins and the partial structure and promoters of the HbI gene from *Lucina pectinata*. We show that HbI has two mRNA variants, where the 5’end had either a sequence of 96 bp (long variant) or 37 bp (short variant). The gene structure of the oxygen reactive Hbs is defined by having 4-exons/3-introns with conservation of intron location at B12.2 and G7.0 and the presence of pre-coding introns, while the partial gene structure of HbI has the same intron conservation but appears to have a 5-exon/ 4-intron structure. A search for putative transcription factor binding sites (TFBSs) was done with the promoters for HbII, HbIII, HbI short and HbI long. The HbII, HbIII and HbI long promoters showed similar predicted TFBSs. We also characterized MITE-like elements in the HbI and HbII gene promoters and intronic regions that are similar to sequences found in other mollusk genomes. The gene expression levels of the clam Hbs, from sulfide-rich and sulfide-poor environments showed a significant decrease of expression in the symbiont-containing tissue for those clams in a sulfide-poor environment, suggesting that the sulfide concentration may be involved in the regulation of these proteins. Gene expression evaluation of the two HbI mRNA variants indicated that the longer variant is expressed at higher levels than the shorter variant in both environments.

## Introduction

The bivalve clam *Lucina pectinata* (*L*. *pectinata*) belongs to the Lucinidae family and lives in the sulfide-rich muds in the Southwestern coast of Puerto Rico and throughout the Caribbean Sea. It houses intracellular symbiotic bacteria that need to be supplied with both hydrogen sulfide (H_2_S) and oxygen (O_2_) in order to fix carbon dioxide into organic compounds that the host uses as nutrients [1]. These sulfide-oxidizing chemosymbiotic bacteria are localized in specialized organelles known as bacteriocytes found in the gill filaments or ctenidia tissue of the clam [2]. Hemoglobins (Hbs) transport O_2_ and H_2_S, which are required for symbiosis and cellular respiration [3]. Three different hemoglobins have been identified in *L*. *pectinata*: hemoglobin I (HbI), a sulfide-reactive protein, and the oxygen-reactive hemoglobin II (HbII) and hemoglobin III (HbIII) [1]. HbI is a 142 amino acid residues monomeric globular protein [4], showing the conventional globin fold, lacking the D helix, just like plant and truncated hemoglobins [5]. HbI is characterized by an unusual distribution of aromatic residues surrounding the distal site in the heme pocket (Phe-29 (B10), Phe-43 (CD1) and Phe-68 (E11)) [6]. This peculiar arrangement of phenylalanine residues at the distal ligand binding site has not been observed before in the globin family, and is unique to HbI. The heme pocket distal E7 position has a glutamine residue instead of the typical histidine. HbI, HbII and HbIII, have similar O_2_ affinities [7], but in presence of H_2_S, HbI reacts with H_2_S to form ferric haemoglobin sulfide [1], while HbII and HbIII remain oxygenated. The H_2_S affinity for HbI is believed to be achieved through fast association (k_on_) (2.3 x 105 M-1 s-1) and very slow dissociation processes (k_off_) (0.22 x 10–3 s-1) [8]. The association rate for HbI with H_2_S is the highest of the known hemoglobins while its dissociation rate is the slowest [6]. The O_2_ reactive Hbs have similar O_2_ affinities [1, 9], having low O_2_ association and dissociation rates in comparison with other monomeric hemoglobins. This slow dissociation rate can be explained by the direct contact of the tyrosine (B10) residue with O_2_, since the resolved crystal structure of dimeric oxy-HbII showed that oxygen is tightly anchored to the heme through hydrogen bonds with Tyr30 (B10) and Gln65(E7) [10]. HbII and HbIII can form dimers and tetramers in a concentration-dependent manner [11]. The crystal structures of HbII homodimers and the HbII-HbIII heterodimer have been resolved [12, 13]. The full-length cDNA sequences of all *L*.*pectinata* hemoglobins have been determined [14–16], but their cognate gene structures remain unreported.

These three hemoglobins have been extensively studied to understand their chemistry and the factors that affect their specificity, as well as used as models to understand distal ligand binding control [6, 17–19]. However, how the environment contributes to the function/chemistry of these hemoglobins and what are their specific roles are aspects that are not completely clear.

In this work we wish to clarify some of these aspects. Here we present the complete gene structures of the oxygen binding hemoglobins and a partial gene structure for the sulfide-reactive hemoglobin. We also show that HbI from *L*. *pectinata* has two mRNA variants coding for the same protein. The promoter regions for HbII, HbIII and both HbI variants are also described, identifying possible transcription factor binding sites (TFBSs) that could contribute to their regulation. Furthermore, comparative analysis of these gene structures led to the identification of repetitive regions with the HbI and HbII promoter and intron regions. In order to have a better understanding of how these Hbs are affected by the symbiosis and their environment, we evaluated hemoglobin gene expression in different juvenile clam tissues at the RNA level, comparing two groups of clams living in two different environments, sulfide-rich and sulfide-poor. We also measured the expression levels of the two HbI mRNA variants in ctenidia tissue for the two groups of clams in the two different environments, to verify if the regulation of these variants was dependent of the environmental conditions.

## Materials and Methods

*Ethics statement***—**Juvenile *Lucina pectinata* clams were purchased from a local fisherman in the town of Cabo Rojo, PR. *L*. *pectinata* is not an endangered or protected species, hence, no specific permits were required to obtain the clams since they are a local food item.

### DNA and RNA isolation

Genomic DNA (gDNA) from *L*. *pectinata* was isolated from ctenidia tissue using either the DNeasy Tissue Kit (QIAGEN) or the Omega Biotek E.Z.N.A. Mollusk DNA Kit, as recommended by the manufacturers.

For the RNA isolation, juvenile clams were transported in seawater containing mud extracted from the site where they were harvested, in order to maintain them in conditions similar to their environment until RNA isolation. Clams were carefully dissected within 24 hours after clam harvest. The rest of the clams were put in a fish-tank with seawater and a pump filter. They were fed phytoplankton twice a week. At 108 days they were snap frozen in liquid nitrogen and stored at -80°C until processed. RNA was extracted from the ctenidia, mantle, muscle, foot, and visceral mass (S1 Fig) using the TRIzol reagent (Sigma-Aldrich), following the modified procedure of RNA isolation described by Chomczynski [20]. To improve the A_260/230_ ratio of the RNA samples, RNAs were extracted with 1-Butanol followed with two consecutive diethyl ether extractions [21], in order to remove traces of TRIzol reagent.

### Genome walking experiments

Genome walking (GW) experiments were carried out using either the DNA Walking Speedup Kit (Seegene) or the Universal Genome Walker^™^ kit from CLONTECH Laboratories, Inc, following to the manufacturer’s instructions.

### PCR, XL-PCR, RT-PCR, cloning, purification and sequencing of PCR or RT-PCR products

All polymerase chain reaction (PCR) amplifications were performed using the Advantage 2 Polymerase Mix kit (Clontech) following the manufacturer instructions, in some cases including 4% of GC Rich solution (Roche Applied Science) in the master mix. Extra-long PCR (XL-PCR) amplifications were performed using the Expanded Long Template PCR System (Roche Applied Science), following the manufacturer instructions. Reverse transcription PCR (RT-PCR) were performed using either the ThermosTable rTh Reverse Transcriptase RNA PCR kit from Perking Elmer or the One^®^Step RT-PCR kit (QIAGEN) following the manufacturer’s instructions. The PCR, XL-PCR or RT-PCR products were purified using the Qiaquick PCR purification kit from QIAGEN and cloned either into pSTBlue-1 Vector Vector using the Perfectly Blunt Cloning kit (Novagen) or into the pCR^®^II Vector using the TA Cloning^®^ Kit Dual Promoter (pCR^®^II) with One^®^Shot INVαF’ kit from Invitrogen, as recommended by the manufacturers. Plasmids were isolated and purified using the QIAprep Spin Miniprep Kit (QIAGEN) and sequenced in both strands using the ABI 310 automated DNA sequencer and dye terminator chemistry (Big Dye V3 105 Dye Terminator Sequencing Kit, Applied Biosystems).

### Determination of *Lucina pectinata* hemoglobin gene structures

The genome walking method and generation of overlapping gene fragments by PCR and XL-PCR were used to obtain unknown intron sequences adjacent to known exon sequences. Target specific primers (TSPs) were designed from the published HbI, HbII and HbIII cDNA sequences [14–16] using the Primer3 software [22]. GW products were cloned, purified and sequenced. Intron sequences were also obtained by PCR or XL-PCR using pairs of oligonucleotide primers that annealed in the exon sequences flanking each intron. All primer sequences are listed in Table A in S1 File. Intron sizes for the HbI, HbII and HbIII genes were determined by PCR using pairs of oligonucleotide primers corresponding to the exon sequences flanking both sides of each putative intron, assuming conservation at intron positions B12.2 and G7. Intron sizes were estimated by subtracting the size of each cDNA PCR product from that of its corresponding genomic PCR product. Overlapping fragments confirmed these sequences; named HbI-Segment1, to HbISegment-3 for HbI gene, HbII-Segment 1 to HbII-Segment 4 for the HbII gene and HbIII-Segment1 to HbIII-Segment 3 for the HbIII gene.

The 5’UTR analysis of HbI mRNA was performed using the SMARTer^™^ RACE cDNA Amplification Kit from Clontech following the manufacturer’s instructions from ctenidia RNA. The sequences obtained were aligned using ClustalW [23] and visualized using the GeneDoc program [24]. The HbI mRNA variants were also confirmed by RT-PCR using two forward primers designed from the new sequences and a reverse primer designed from the third exon of the HbI gene. To confirm these mRNA products, the PCR products were purified and directly sequenced as described above.

The genome walking (GW) method was used in order to obtain the 5’end of the HbI gene sequence, two sets of GW experiments were performed. Firstly, gene specific primers (GSP) annealing to the second exon of the HbI incomplete gene sequence previously obtained were designed. Genome walking products were purified, cloned and sequenced in both strands as described above. Secondly, gene specific primers (GSP) were designed from the first incomplete intron of the HbI gene sequence. The final PCR products were purified, cloned and sequenced. The gene sequence obtained by the first GW was confirmed by PCR using four different combinations of oligonucleotides and PCR products were purified and sequenced directly. The gene sequence segment obtained by the second GW was also confirmed by PCR. Two forward primers were designed from the new sequence and as a reverse primer we used a primer located in exon 3 of the HbI gene sequence.

### Determination of promoter regions of *L*. *pectinata* genes and bioinformatics analysis

In order to characterize the promoter regions of HbI, HbII and HbIII genes we used the GW method. Reverse GSPs were designed from the first non-coding exon of the HbII and HbIII genes. Since HbI appears to have two different first exons, GSPs were designed from both of these exons (short exon 1_S_ and for the long exon 1_L_). The final PCR products were purified, cloned and sequenced as described above. The sequences were assembled and nucleotide positions with conflicts resolved based on their chromatograms with CodonCode Aligner 2.0.6 (CodonCode Corporation, www.codoncode.com). A search for CpG islands was performed using the tools available on the Sequence Manipulation Suite [25], while putative conserved transcription factor binding sites (TFBS) that are positionally conserved between the oxygen-binding HbII and HbIII promoters were identified using the CONREAL web server [26]. The parameters used were: 80% threshold for position weight matrices; length of flanks to calculate identity = 10 bp; threshold for identity = 50%. Conserved motifs between HbII and HbIII promoters were identified with the MEME program using zero or one occurrence per sequence, number of motif = five, minimum width = 6 and maximum width = 50 [27]. The positionally conserved motifs predicted by these methods were searched against the JASPAR CORE database [28] to identify possible transcription factors that may bind to these motifs, using the Tomtom Motif Comparison Tool, Version 4.10.0 [29]. For the HbI short and long variant promoters, the search for potential TFBSs was performed using AliBaba 2.1 (http://www.generegulation.com/pub/programs/alibaba2/index.html) and TFBIND (http://tfbind.hgc.jp/) [30]. We used the AliBaba2.1 program with the following parameters: pairsim to known sites = 50, matrix width in bp = 10, minimum number of sites of which a matrix is build = 4, minimum matrix conservation = 80%, similarity of sequence to matrix = 1% (1% means that the unknown sequence is just similar to the matrix) and factor class level = 4. We also search for conserved motifs with the MEME analysis between the four promoters as described above.

### Comparative analysis of *L*. *pectinata* Hbs gene structures

*L*. *pectinata* hemoglobin gene structures were compared using the BLAST tool [31–33], using Blastn with default parameters and optimized for somewhat dissimilar sequences. The JDotter program [34] was used to generate dotplots of those gene sequences found to contain repetitive regions. Clustal W multiple sequence alignments were performed with repetitive elements found across sequences and GeneDoc [35] was used to visualize and format those alignments. Blastn searches, using Blastn optimized for somewhat similar sequences, were performed using consensus sequences of the repetitive regions found in *L*. *pectinata*’s Hbs as query against the mollusk (taxid:6447) reference genomic sequences (refseq_genomic) in the nucleotide database and the TIGR plant repeat data base [36] to compare to repetitive elements found in the rice plant *Oryza sativa* (database: TIGR_Oryza_Repeats.v3.3).

### Real time RT-PCR analysis for the three hemoglobins and two 5’end HbI mRNA variants of *L*. *pectinata*: H_2_S-rich environment vs. H_2_S-poor environment

Real-time quantitative PCR was carried out in a StepOnePlus^™^ Real-Time PCR System from Applied Biosystems. *L*. *pectinata* hemoglobin mRNA levels were measured using the SYBR^®^ Green PCR Master Mix from Applied Biosystems. Primers for each hemoglobin mRNAs (HbI, HbII and HbIII), and a housekeeping gene (18S rRNA) were designed using Primer Express Software (Applied Biosystems) 3.0 and Primer 3 Software (see Table A in S1 File). Primer set validation was made with 1:2 serial dilutions of ctenidia total RNA ranging from 20–1.25ng to obtain the amplification efficiencies (E) for each primer set. The RT-PCR master mix included 10μM of each primer and 10 ng of template RNA in a 20 μL total reaction volume. The PCR program employed was: 48°C for 30 min (reverse transcription step), 95°C for 10 min, followed by 40 cycles of 95°C for 15 sec (denaturation), 60°C for 1 min (annealing and extension), and detection of fluorescent signals at 76°C for 15 sec. For melt curve analysis, the program used was: 95°C for 15 sec, 60°C for 1 min, 95°C for 15 sec, with data collection at the ramp between 60°C to 95°C.

For the 5’end HbI mRNA variants, primer set validation was done with 1:2 serial dilutions of ctenidia total RNA ranging from 50–6.25 ng. The RT-PCR master mix recipe used 20 ng of total ctenidia RNA and the other components remained as mentioned in the previous section. The PCR program used was that recommended by the manufacturer.

Five clams (biological replicates) harvested in their natural environment and five clams kept in a fish tank for 108 days were tested and each sample was analyzed in duplicate, including no template controls. The template quantity (R_o_) was calculated using the following equation: R_0_ = (1+E)^-Ct^ [37]. In order to obtain the mRNA expression for each target gene, the average amount of mRNA for each target gene was normalized by dividing it against the average amount of mRNA of the endogenous control in each tissue: mRNA expression = (R_0 Target gene_)/ (R_0 Endogenous control_).

The mRNA expression levels were presented as aligned dot plots using a logarithmic scale showing means as horizontal bars and standard error of the mean (SEM) with whiskers. The un-paired t test with Welch’s correction, used for samples with unequal variances, was performed on normalized data. Differences were considered significant at p<0.05.

### Reverse transcription PCR analysis for HbI mRNA variants, HbII and HbIII in ctenidia tissue: H_2_S-rich environment vs. H_2_S-poor environment

In order to verify the cDNA coding sequences for both HbI mRNA variants, and the HbII and HbII mRNAs we performed RT-PCR for all transcripts. Using ctenidia tissue from three biological replicates from each environments tested in order to determine if environmental changes affected mRNAs at the sequence level. PCR products were purified and sequenced directly. Sequences were aligned with ClustalW [23] and visualized using the program GeneDoc [35].

## Results

### Determination of *Lucina pectinata* gene structures

**Fig 1:**
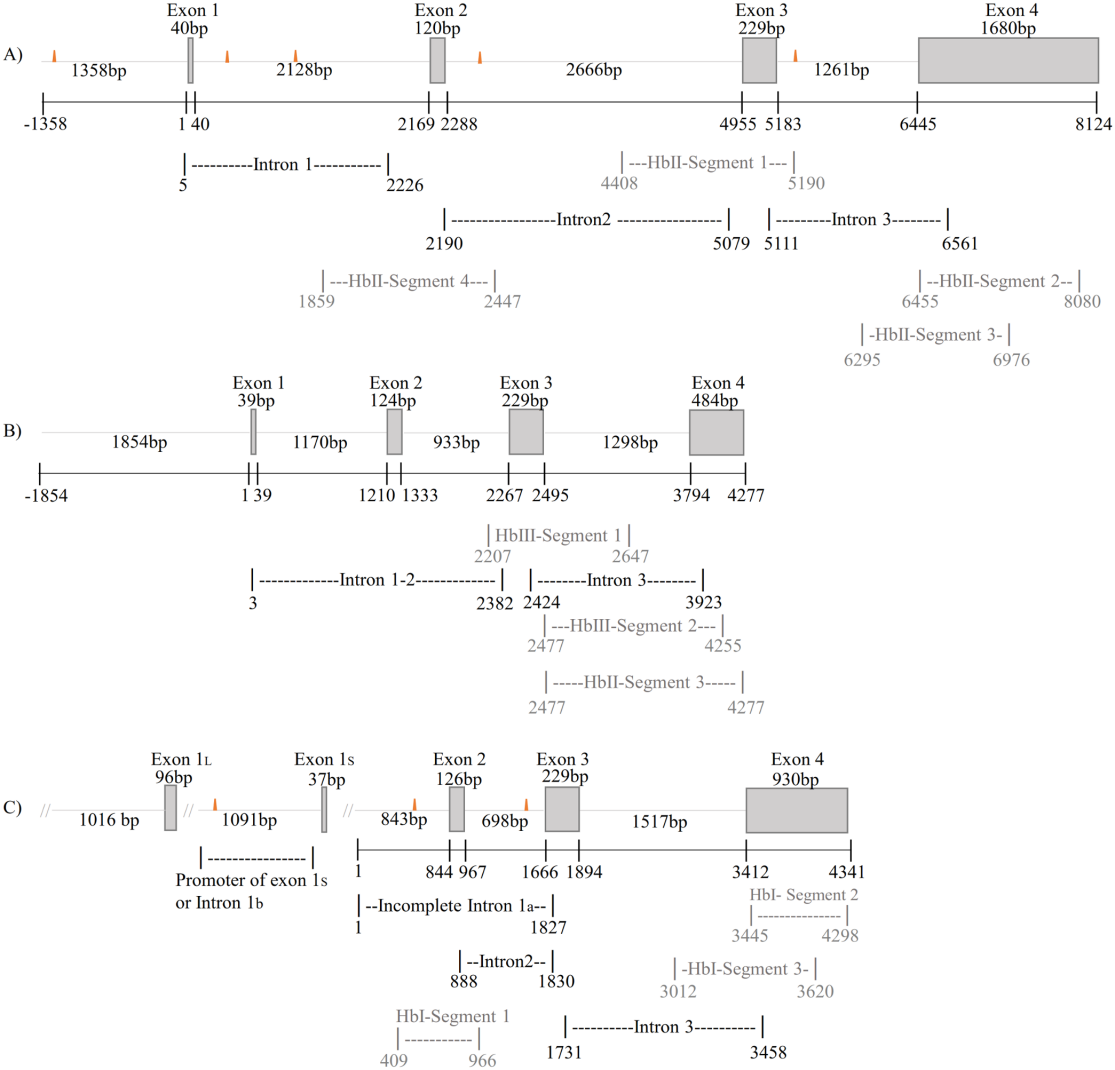
*Lucina pectinata* hemoglobin gene structures. Schematic diagram of *L*. *pectinata*’s hemoglobin genes. The exon regions are represented by boxes and introns as dashed lines. The orange triangles represent the location of MITE-like element found in these genes. The sequences preceding exon(s) 1 are the promoter regions obtained by the GW method. **A)** Schematic diagram of HbII gene structure. Shown are the localization and length of the XL-PCR products amplified to determine the intron sizes and overlapping PCR products used to verify the 3’ end of the gene. The total length of the four exons and three introns in the *L*. *pectinata* HbII gene is 8,055 bp. **B)** Schematic diagram of HbIII gene structure. The exon regions, the localization and length of the XL-PCR products and overlapping PCR products are indicated as for the HbII gene in A. The total length of the four exons and three introns in the *L*. *pectinata* HbIII gene is 4,277 bp. **C)** Proposed schematic diagram of HbI partial gene structure. The localization and length of PCR amplified to determine the intron sizes and overlapping products are shown. HbI incomplete gene length (from incomplete intron 1_a_ to the end of exon 4) is 4,341 bp. Alternate first exons are label exon 1_L_ and exon 1_S_.

For the HbII and HbIII genes, DNA sequencing of cloned amplified products derived from genomic DNA provided a partial sequence. We employed XL-PCR to obtain additional information and generate overlapping PCR products to confirm the obtained sequences. Fig 1A and 1B shows the gene structures of the HbII and HbIII genes with the overlapping segments that corroborated these sequences. For HbII and HbIII, additional pre-coding introns were found located in the regions corresponding to the beginning of the 5’UTRs. The full-length sequences of the *L*. *pectinata* HbII and HbIII genes have Genbank accession numbers **EU999997** and **EU937817**, respectively.

For HbI, intron sizes were determined by sequence analysis of PCR products and Genome Walking. Fig 1C shows the incomplete HbI gene structure with the overlapping segments (HbI Segment-1 to HbI Segment-3) that corroborated this sequence. With the first and second GW experiments, 843 bp belonging to the intron preceding the putative exon 2 (Incomplete intron 1_a_ in Fig 1C) were obtained, but the first twelve bases of the HbI cDNA sequence previously reported by F. Antommattei [14] were absent. This is indicative of an additional intron in the pre-coding region, as was determined for the genes of the oxygen-binding hemoglobins. The *L*. *pectinata* HbI partial gene sequence has GenBank accession number **KU323925**.

### Confirmation of 5’ end of HbI cDNA by 5’- RACE method

**Fig 2:**
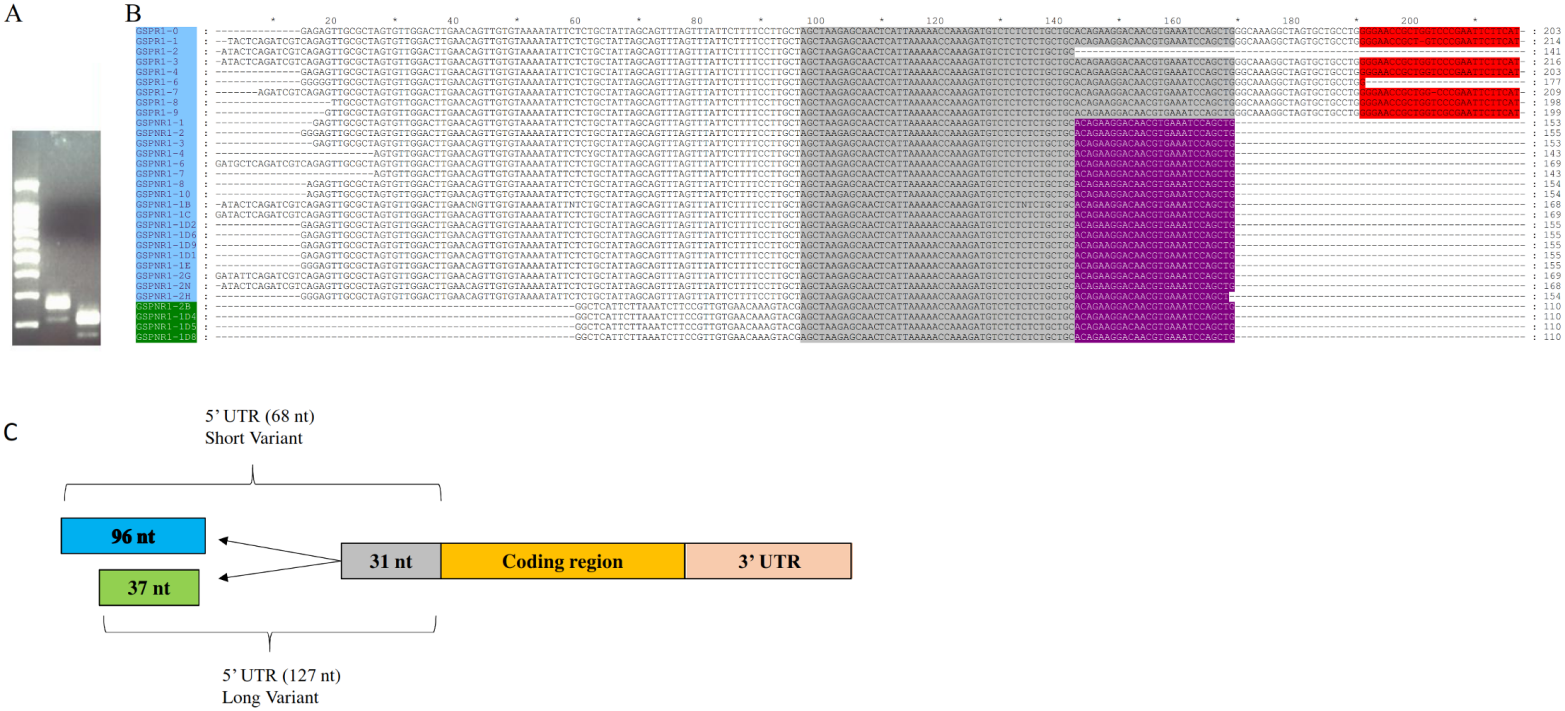
HbI produces two mRNA variants that differ in their 5’ ends. **A)** HbI 5’RACE products analyzed in a 2% electrophoresis gel: Lane 1) 100bp plus ladder from QIAGEN, 2) RACE product with GSP1R reverse primer, 3) RACE product with GSPN1R reverse primer. **B)** Multiple Sequence Alignment of HbI’s mRNA variants sequences using ClustalW and visualized in GeneDoc**:** The long variant sequences are highlighted in blue and the short variant sequences are highlighted in green. The bases were the GSP1R and GSPN1R primers annealed are highlighted in red and purple, respectively. **C)** Schematic representation of HbI mRNA variants.

The 5’RACE method was used to confirm the HbI cDNA sequence previously reported by Antommattei *et al*., [14], with two nested primers, both designed from the HbI exon 2 (the exon that ends on B12.2), in order to ensure specificity. These 5’RACE products were analyzed by gel electrophoresis, showing the existence of two products with an approximately 50 bp molecular weight difference (see Fig 2A). The sequencing results of the cloned RACE products showed that for the smaller product the initiation codon was preceded by 68 nucleotides while the product with higher molecular weight had 127 nt preceding the initiation codon. Both of these variants share 29 nt before the start codon that belong to exon 2 of HbI at the gene level, resulting in a short (S) variant having 37 nt (which contains the first 12 nt reported by Antommattei [14]) and a longer (L) variant having 96 nt. Fig 2B shows these sequences aligned and visualized in GeneDoc, the sequence shaded in black is the common region between both variants, which corresponds to the boundary of exon 2 at the gene level. The open reading frame in both transcripts starts at the same position in exon 2. Fig 2C shows the schematic representation of these mRNA variants. The HbI mRNA variants partial sequences have GenBank accession numbers **KU524081** and **KU524082** for the short and long variants, respectively.

### Determination of promoter regions of *L*. *pectinata* genes

We obtained 1358 bp and 1854 bp upstream of the transcription start site (TSS) of HbII and HbIII by GW, respectively (S2A and S2B Fig). The promoter regions of the HbII and HbIII genes have Genbank accession numbers **KU524077** and **KU524078**, respectively. Since two 5’UTR’s were found for HbI mRNA, two promoters were found for each variant using the GW method, of 1,091 bp and 1,016 bp for the HbI short (HbI_SV) and HbI long (HbI_LV) variants, respectively (S3A and S3B Fig). The promoter regions of HbI_SV and HbI_LV have Genbank accession numbers **KU524079** and **KU524080**, respectively. For the oxygen-binding hemoglobins, TATA boxes were located at -36 and -29 from the TSS for HbII and HbIII, respectively. For the promoters of HbI variants, a TATA box was found at -32 from the TSS for the HbI_SV, while no TATA box was found for the HbI_LV. On the other hand, no CpG islands were found in either the HbI_SV or HbI_LV, or the HbII and HbIII promoters.

**Fig 3:**
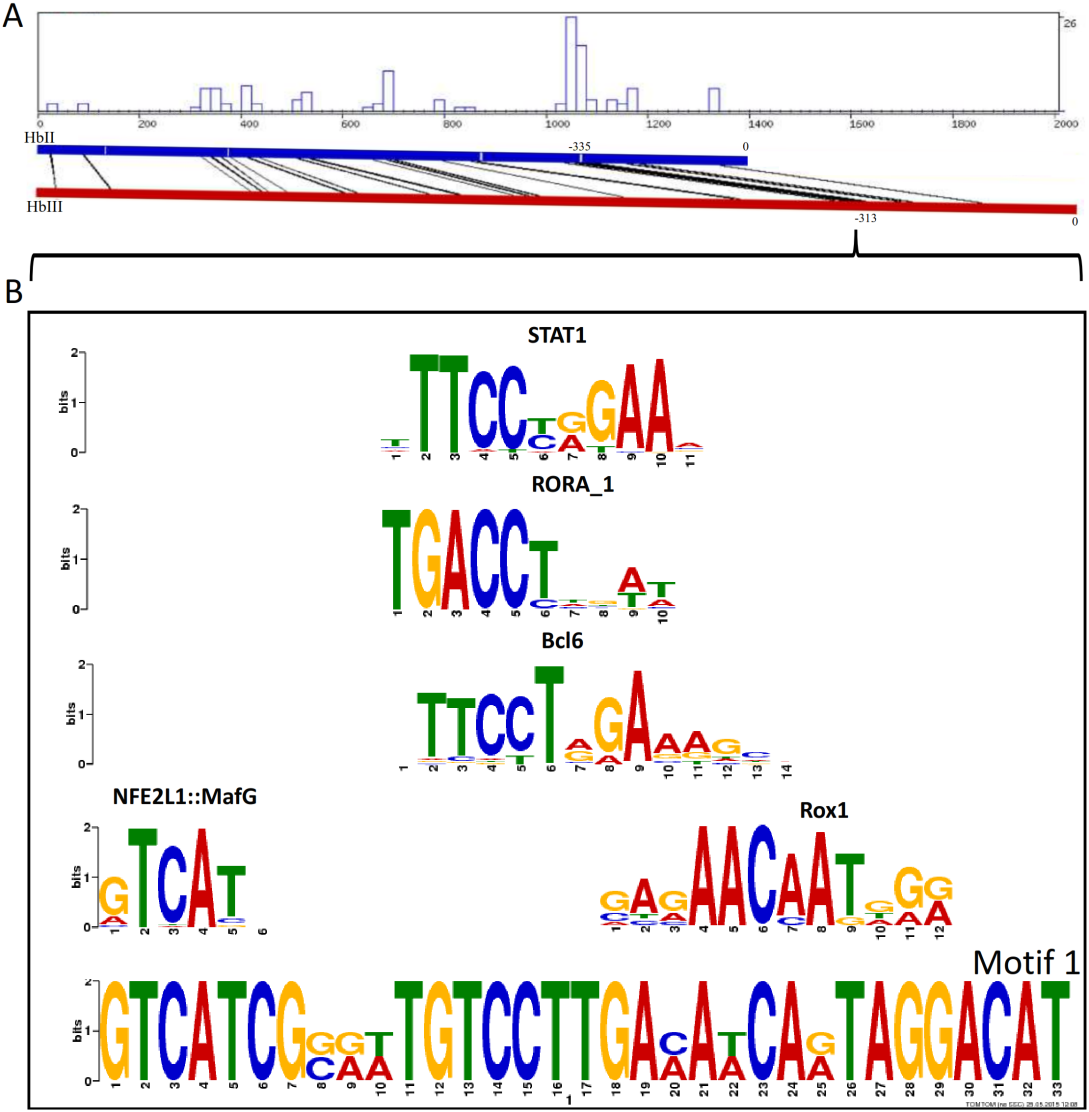
Bioinformatics analysis of the HbII and HbIII promoters. **A)** Output of the CONREAL web-interface. The results are visualized graphically showing the aligned positions of the HbII and HbIII promoter regions performed using the LAGAN method. The graph show the positions of aligned hits and the distribution/concentration of conserved TFBSs along the sequences. The regions with higher concentration of TFBSs are -335 to -267 for HbII and -313 to -246 for HbIII. Motif 1, 33 bp long, was found by MEME analysis and is located at this region (starts at position -306 in HbII and -288 in HbIII). **B)** Logo of the motif found by MEME analysis (below, named motif 1) with the first five TFBSs found by TOMTOM analysis against the JASPAR core (2014) database.

Since HbII and HbIII are oxygen-reactive Hbs, we compared their promoter regions in order to have a better idea of the transcription factors that may regulate them. For these two promoters several positionally conserved TFBSs were predicted using the CONREAL web server [26, 38] with LAGAND and AVID alignments. For HbII and HbIII, the upstream regulatory regions from -180 to—335 and -160 to -313, respectively, showed the highest number of predicted TFBSs, the strongest ones include GATA-1, GATA-2, STAT-4, STAT-6, p53, Oct-1, RORalpha1 (RORα), TCF11-MafG, CREB, and CdxA (see Table B in S1 File). With MEME analysis, we found one motif that correlates with the region that has the highest number of TFBSs found by the CONREAL. TFBSs predictions for this motif were done with TOMTOM and by searching the two 32 bp long sequences from HbII and HbIII that comprised this motif, independently, against the JASPAR database [28]. These analyses showed TFBSs that are common for these two sequences in this motif region: RORα, ROX1, NFE2L1, Esrrb, D, vnd, GLN3, Sox-5, Pax-2, and Nr5a2 (see Table C in S1 File). In Fig 3 we present the LAGAN alignment summary, specifying the region that corresponds to this motif found by MEME, with the five most significant TFBS matches found for this motif.

A different approach was used to analyze the HbI_SV and HbI_LV regions. Each one was analyzed independently, taking into consideration that they may be subject to different regulation as a consequence of an alternative promoter mechanism [39]. We also searched for TFBSs with the TFBIND webserver in order to compare and select the best matches between the two programs. The localization of the TFBSs found by Alibaba2.1 are represented in S4 Fig; those that were also found with TFBINB are colored in green. The TFBSs predicted by TFBIND with a score > 80% are listed in Tables D and E in S1 File, for the HbI_SV and HbI_LV promoters, respectively.

MEME analysis of the four promoters showed conservation of a positionally conserved motif of 40 bp long starting at -317, -296, -203 and -74 for HbII, HbIII, HbI_SV and HbI_LV promoters, respectively (data not shown). For the HbII and HbIII promoters, this motif overlaps with the motif region found by MEME analysis using only these two promoters (described above), therefore we will discuss this motif for HbI_SV and HbI_LV promoters. The location of this motif in the HbI_SV promoter is similar to the location of this motif in the HbII and HbIII promoters. A TFBSs search of the sequence that comprised this motif in HbI_SV and HbI_LV were analyzed independently against the JASPAR database [28] with a relative profile score threshold of 80% (see Table F in S1 File, for HbI_SV promoter, and Table G in S1 File, for HbI_LV). The motif found in HbI_LV promoter showed more predicted TFBS than the motif found in HbI_SV promoter and it also had similar predicted TFBS to the motif found in HbII and HbIII promoters, like RORα, GLN3 and MafG, among others.

### Comparative analysis of *L*. *pectinata* Hbs gene structures

**Fig 4:**
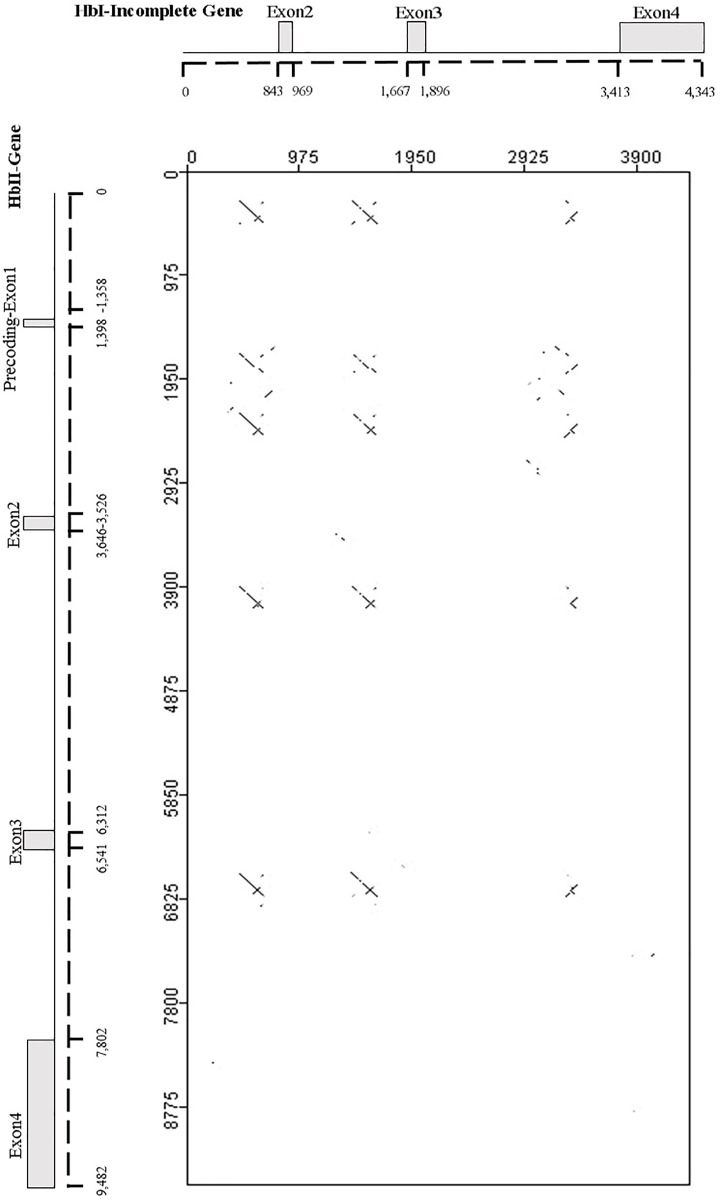
Dot plot comparison of the incomplete HbI gene to the HbII gene. A repetitive region in HbI intron and HbII promoter sequence and introns was detected. The HbII gene structure is represented in the horizontal axis and the HbI incomplete gene structure is shown in the vertical axis.

To find similarities across the gene structures of these three Hbs we performed comparisons using blastn. First we compared the HbI incomplete gene sequence with the HbII gene sequence including the promoter region (gene-promoter). Interestingly, the blastn results showed segments with high similarities (>80%) between the HbII promoter and intronic regions of both genes: for the HbI, similarities occur nearer the 3’ end of introns 1_a_, 2 and 3; for HbII, they occur in its promoter region (from -865 to -1,077, relative to the TSS), nearer the 5’ end of introns 2 and 3 and two repeats in intron 1. To better visualize this, we used the JDotter program [34] to align the known HbI incomplete gene against the HbII gene-promoter and generated a dot plot graph. The resulting dot plot graph showed regions with similarities representing the alignments as lines; plus strand slanted from the top left to the bottom right corner, minus strand matches are slanted from the bottom left to the upper right. The dotplot graph of the HbI incomplete gene vs the HbII gene-promoter is illustrated in Fig 4, the tabulated results of the blastn alignment are provided in Table H in S1 File. When the HbIII gene-promoter was compared against the HbII gene-promoter no such similarities were observed across the introns. There were similarities across HbII and HbIII exons 2 and 3, but this was expected since these exons have the nucleotides coding for the proteins and the amino acid sequence identity between these two hemoglobins is relatively high (65%) (not shown). Comparing the HbI incomplete gene and the HbIII gene-promoter region, there was only a segment of 78 bp located in intron 3 for both genes that had 74% identity.

**Fig 5:**
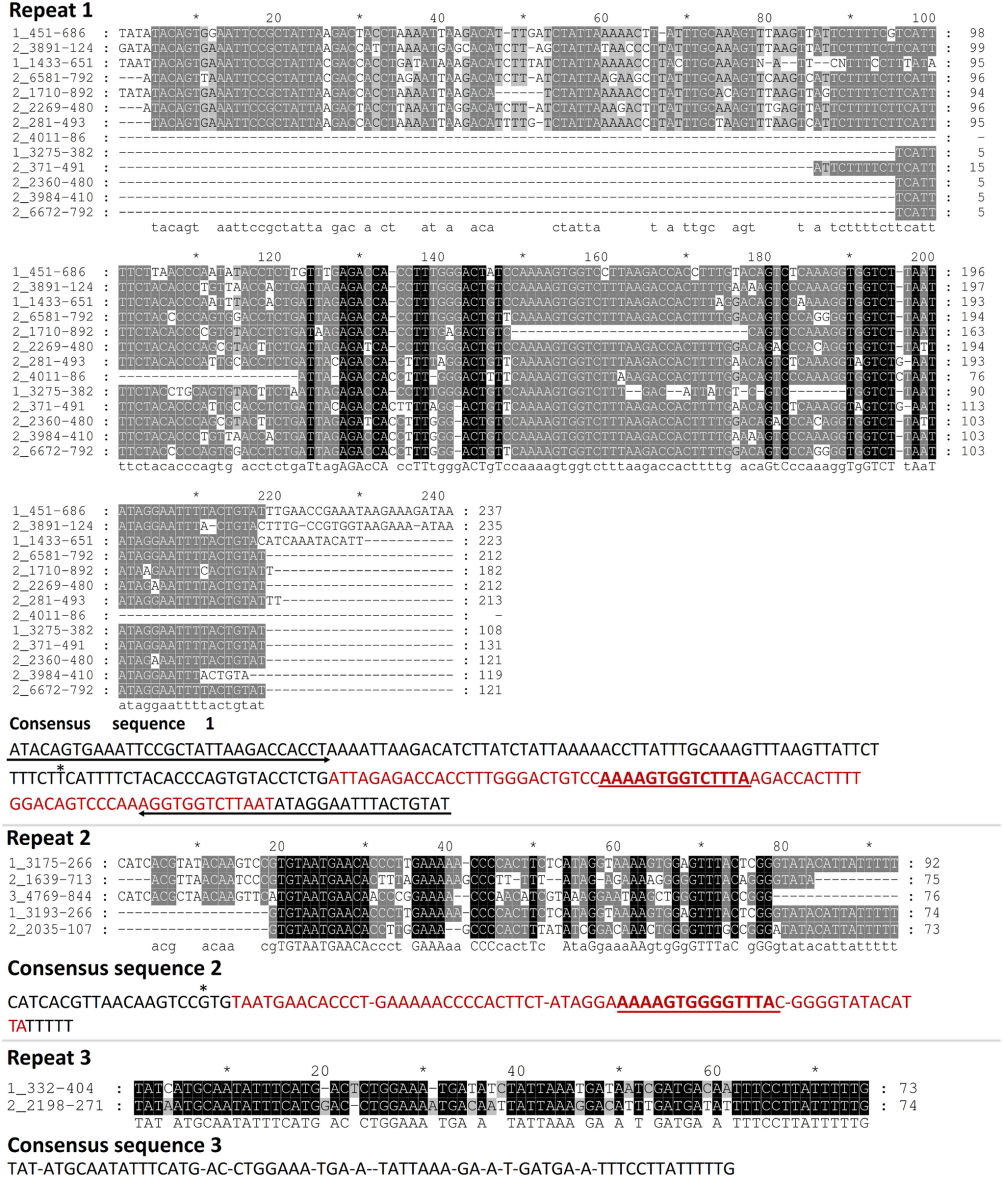
Alignment of repetitive segments found in *L*. *pectinata’*s Hbs genes. The first number of each sequence is 1, 2 and 3 for HbI, HbII and HbIII, respectively. The following number indicates the region of each sequence in its respective gene. Three alignments, named repeat 1, 2 and 3 were generated using Clustal W [23] and visualized and formatted with GeneDoc [35]. For each repeat, a consensus sequence was derived according to the majority principle and is shown below each alignment. In consensus sequence 1, arrows specified imperfect inverted repeats found at the flanking regions of this sequence, bases in red indicate the self-complementary segment and the asterisk indicates the start of the truncated sequences. Consensus sequence 2 has a self-complementary segment represented with letters in red. It also has a short segment in common with consensus sequence 1 which is indicated as underlined letters. Consensus sequence 3 has few similarities with other consensus sequences.

Clustal W alignments with these repetitive segments generated three different alignments (see Fig 5). A consensus sequence of 210 bp is produced by the first alignment, named repeat 1 in Fig 5. This consensus sequence 1 has a 30 bp imperfect inverted repeat (IR), with a 90% of identity (27/30), at both terminal ends and a self-complementary or palindromic region with 96% identity central segment of 77 bp. As shown in the alignment for repeat one, not all the sequences have the same length. The start of the truncated sequences is indicated with an asterisk in the consensus sequence. Consensus sequence 2 is 92 bp long and has a 68 bp self-complementary segment with 74% identity. No IR were found in consensus sequence 2. Lastly, consensus sequence 3, derived from the alignment of two segments only, does not share any of the features found in the previous sequences. We searched HbI_SV and HbI_LV promoters with the three consensus sequences using blastn. Only consensus sequence 1 produced significant hits against the HbI_SV promoter at 3 different regions, see Table 1.

**Table 1:** Tabulated Blastn results of most significant hits of consensus sequence 1 (query sequence) and HbI short promoter (subject sequence).

Sequence regions	% similarity	length	Mismatches	Gap openings	Start query	End query	Start subject (fromTSS)	End subject (fromTSS)	E-value	Bit score
1	69.027	113	35	0	91	203	156 (-935)	44 (-1047)	2.36E-09	46.4
2	73.134	67	18	0	124	190	57 (-1034)	123 (-968)	1.00E-07	41
3	81.579	38	5	2	159	195	57 (-1034)	93 (-997)	1.81E-04	30.1

**Fig 6:**
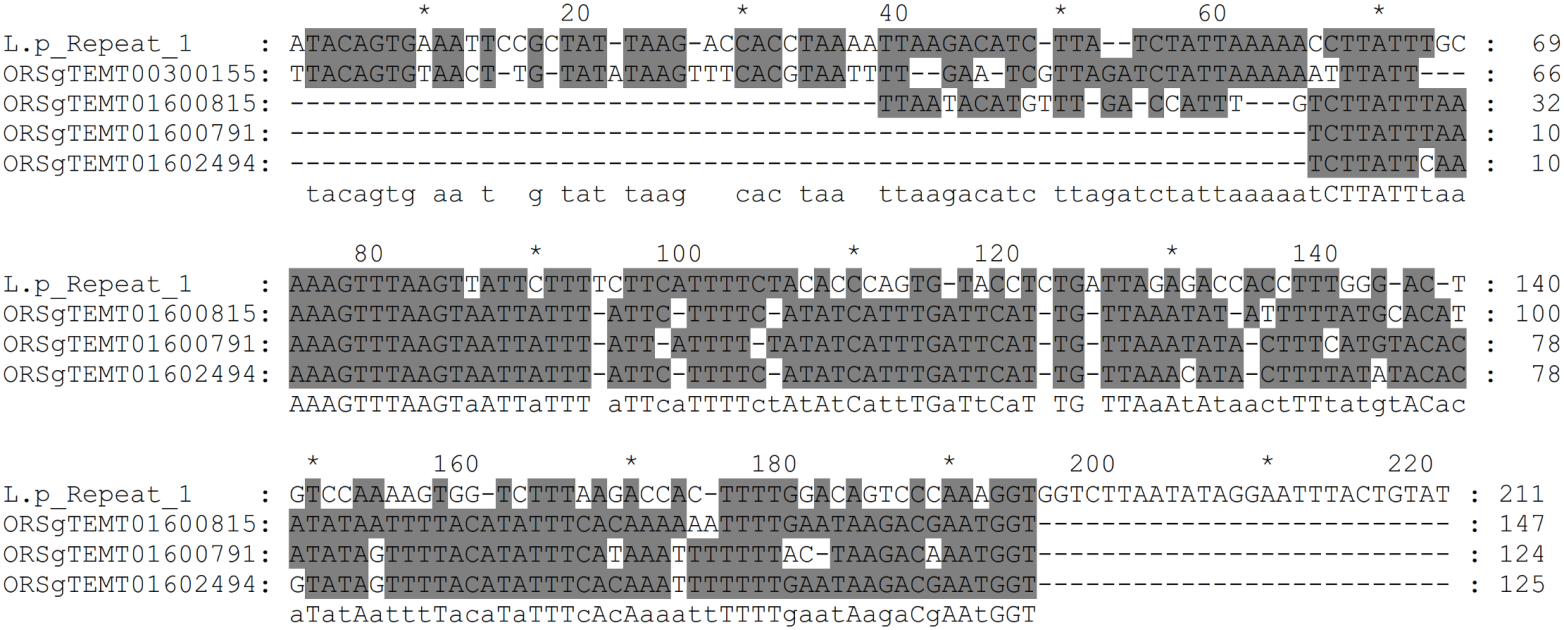
Sequence similarities between repetitive consensus sequence 1 and MITE adh, like B elements. Similarities found between repetitive consensus sequence 1 of *Lucina pectinata* and MITE adh, like B elements sections of taken from the *Oryza* repeat database (TIGR_Oryza_Repeats.v3.3). The sequences used for this comparison are ORSgTEMT01602494 (gi19698268), ORSgTEMT01600815 (gi11034690), ORSgTEMT01600791 (gi22091854) and ORSgTEMT00300155 (gi24371322).

Consensus sequence 1 is the most abundant and possesses some characteristics of a type of transposable element known as miniature inverted-repeat transposable elements (MITEs). These elements are characterized by: having a relatively small size (50-500bp), high copy number, AT-rich sequence, lack the capacity for coding for transposases, the ability to fold into secondary structures, terminal inverted repeats and two flanking short direct repeats [40, 41]. In our case, we could not identify the short direct repeats that are generated by the target site at the moment of transposon insertion. Of the three sequences described, consensus sequence 1 is capable of forming a stable secondary structure (ΔG = -43.95 kcal.mol^-1^). MITE families have been extensively studied in the rice genome of *Oryza sativa*, they create large genome diversity by causing alterations in gene functions and gene regulation [42, 43]. We performed blastn searches using the rice plant *Oryza sativa* database and some similarities were found with sequences that are characterized as MITE-adh, type B like elements (see Fig 6). Similar transposable elements have been described in other mollusks [41, 44, 45]. However no similarities were found between these elements and consensus sequence 1. Furthermore, searches against mollusks database with consensus sequences 1, 2 and 3 used as query, showed fragments that share high similarities (>80%) with sequences of other mollusk such as, *Biomphalaria glabrata*, *Aplysia californica*, *Crassostrea gigas* and *Lottia gigantea* (see S5 Fig and the tabulated blastn results for consensus sequence 1, 2 and 3 in Tables I-K in S1 File, respectively).

### Real time RT-PCR analysis for the three hemoglobins and 5’end HbI mRNA variants of *L*. *pectinata*: H_2_S-rich environment vs. H_2_S-poor environment

We compared the three *L*. *pectinata* hemoglobin gene expression profiles of two groups of clams subjected to different habitat conditions. A group of clams was harvested from their natural environment (sulfide-rich environment) and the other group was kept in a fish tank with fresh sea water (sulfide-poor environment). Gene expression analysis at the mRNA level of *L*. *pectinata* Hbs in different tissues was done by quantitative Real Time RT-PCR, using 18S rRNA as endogenous control. Since the SYBR Green molecule may bind to double-strand DNA in a non-specific manner, secondary products contributed to the Ct values of the samples derived from tissues of lowest expression. To overcome the contribution of secondary products, such as primer dimers, that were formed in tissues with low hemoglobin expression, we added an additional step in the PCR reaction for detection of fluorescence at 76°C for 10 sec instead of during the 60°, 1 min step. This temperature was lower that the melting temperature (Tm) of the amplification products, but higher than the Tm for the primer dimers.

**Fig 7:**
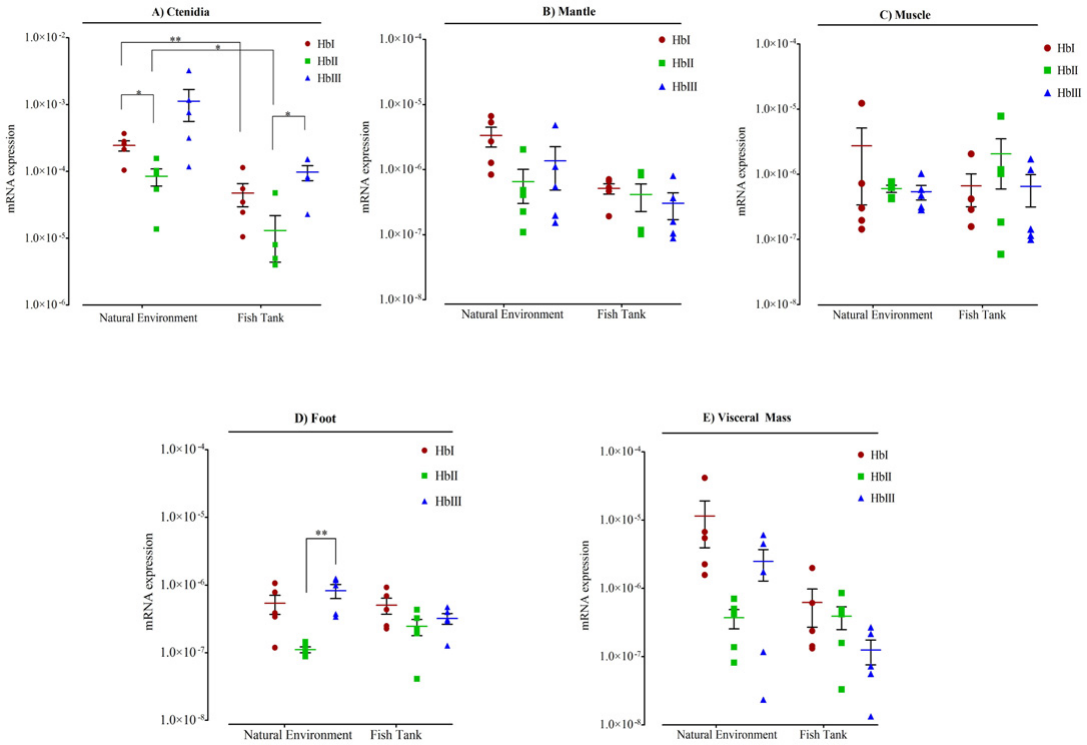
Relative quantitation of mRNAs of *Lucina pectinata*’s Hbs in all tissues examined for both conditions. Data is represented as aligned dot plot using logarithmic scale, showing mean (horizontal bars) and SEM (whiskers). One group was harvested in their sulfide-rich mud natural environment (labeled on X axis as Natural environment) and another group of clams were kept in a fish tank in fresh sea water (labeled on X axis as Fish Tank). Significant differences are indicated with an asterisk at p < 0.05, and with two asterisks at p < 0.01. Gene expression analysis at the mRNA level of *L*. *pectinata* hemoglobins (Hbs) was measured using Real Time RT-PCR. **A)** Hbs expression in ctenidia tissue; **B)** Hbs expression in mantle tissue; **C)** Hbs expression in muscle tissue; **D)** Hbs expression in foot tissue; **E)** Hbs expression in visceral mass tissue.

Fig 7 shows the mRNA expression levels of the three *L*. *pectinata* Hbs in the five tissues examined for both conditions. To assess differences in Hb expression two comparisons were made: differences in expression levels of the Hbs in the same tissue in each environment and differences in expression level of the same Hb in each tissue between the two environments. The three Hbs were expressed in all tissues examined for both conditions. In both conditions, ctenidia tissue had the highest expression of the three Hbs when compared to all of the other tissues. However, when we compared the expression of the Hbs in one tissue in both environments, we observed that ctenidia was the only tissue with a significant change in Hb expression level across environments. Ctenidia from the clams kept in a fish tank had lower expression levels of HbI and HbII than ctenidia from the clams harvested in their natural environment. Even though HbIII appears to have higher expression in ctenidia from the clams harvested from sulfide-rich environments, when compared to its expression in ctenidia from those clams kept in a sulfide-poor environment, this change is not significant due to the variability encountered in the expression of this mRNA across samples from the sulfide-rich environment.

The clams that were fed with phytoplankton for 108 days in a fish-tank were in a mud free environment, with much lower H_2_S concentrations than their natural habitat. It is important to point out that air was continuously supplied by a filter pump in the fish tank, in comparison with their natural habitat, where these clams build hollow tubes to the sediment/water interface and filter the water column to bring nutrients and O_2_ for respiration [46, 47]. The Hbs mRNA expression levels for this group shows that the three Hbs are also highly expressed in the ctenidia tissue, where HbII is also the transcript with lower expression. In this tissue, HbII is expressed 28 times less than HbIII, while no significant difference is detected for HbI and HbIII or HbI and HbII. Contrary to the group that was harvested from their natural environment, in this group HbII has no significant difference in expression among all tissues. HbI and HbIII have a significant lower expression in foot, mantle, muscle and visceral mass tissues when compared to ctenidia tissue.

**Fig 8:**
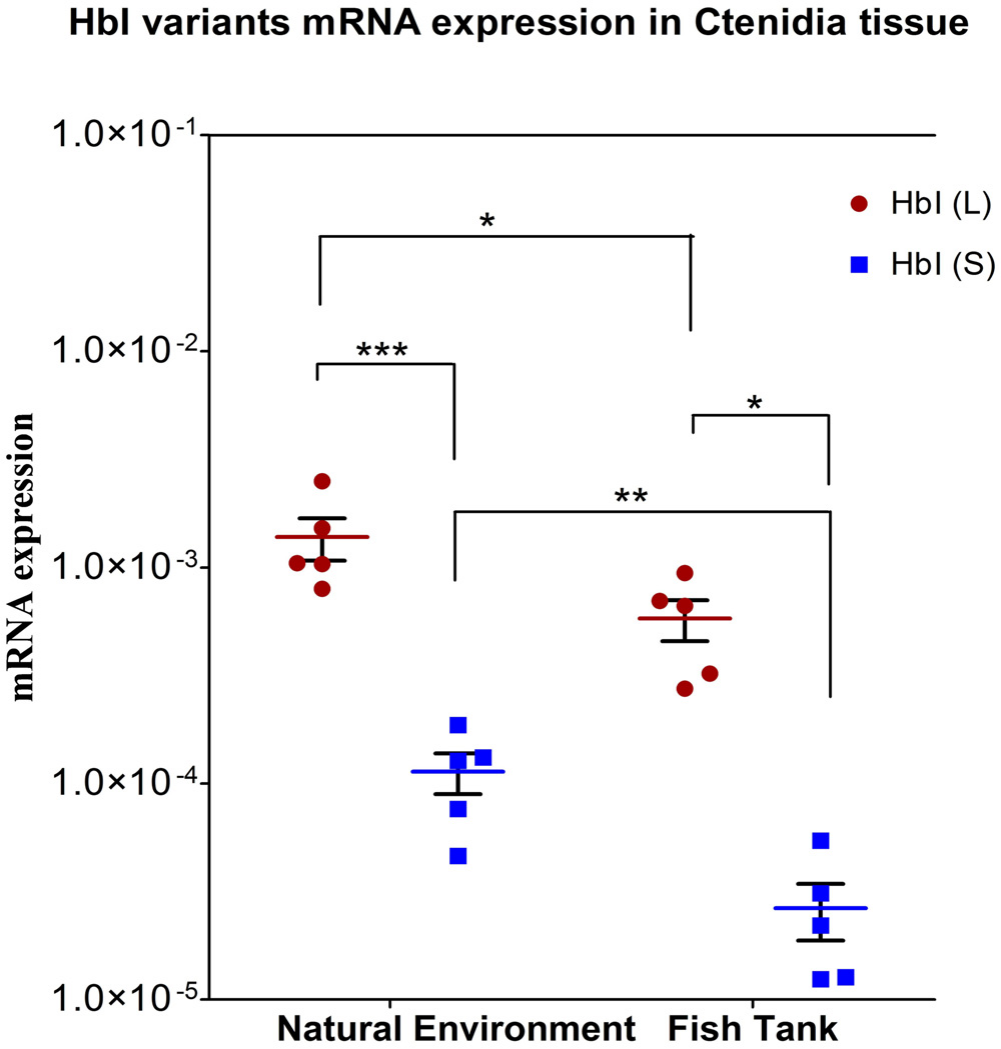
Relative quantitation of the two HbI mRNAs variants in ctenidia tissue for both studied conditions. Data is represented as aligned dot plot using logarithmic scale, showing mean (horizontal bars) and SEM (whiskers). One group was harvested in their sulfide rich mud natural environment (labeled on X axis as Natural environment) and another group were kept in a fish tank in fresh sea water (labeled on X axis as Fish Tank). Significant differences are indicated with an asterisk at P < 0.05, and with two asterisks at P < 0.01. Gene expression analysis at the mRNA level of L. pectinata hemoglobins (Hbs) was measured using Real Time RT-PCR.

In order to understand the presence of two mRNA HbI variants coding for the same protein we conducted qRT-PCR with ctenidia RNA from clams subjected to two different environments: sulfide-rich and sulfide-poor environment. As we saw in the previous section, the ctenidia tissue had the higher expression of hemoglobins, while the expression in other tissues were relatively similar and had no statistical differential expression. For this reason we decided to measure the variant mRNA levels just in the ctenidia tissue. The mRNA expression for each variant in the two conditions are shown in Fig 8. For both conditions the longer variant has significantly higher expression levels than the shorter. The long variant was expressed 12 times more than the shorter variant in ctenidia from the group harvested in their natural environment and 22 times more in ctenidia from the group kept in a fish tank. The level of expression for both variants decreased in the group from the fish tank, the longer variant decreased by a magnitude of approximately 2 and the shorter variant decreased by a magnitude of 4.

### Reverse transcription PCR analysis for HbI mRNA variants, HbII and HbIII in ctenidia tissue: H_2_S-rich environment vs. H_2_S-poor environment

**Fig 9:**
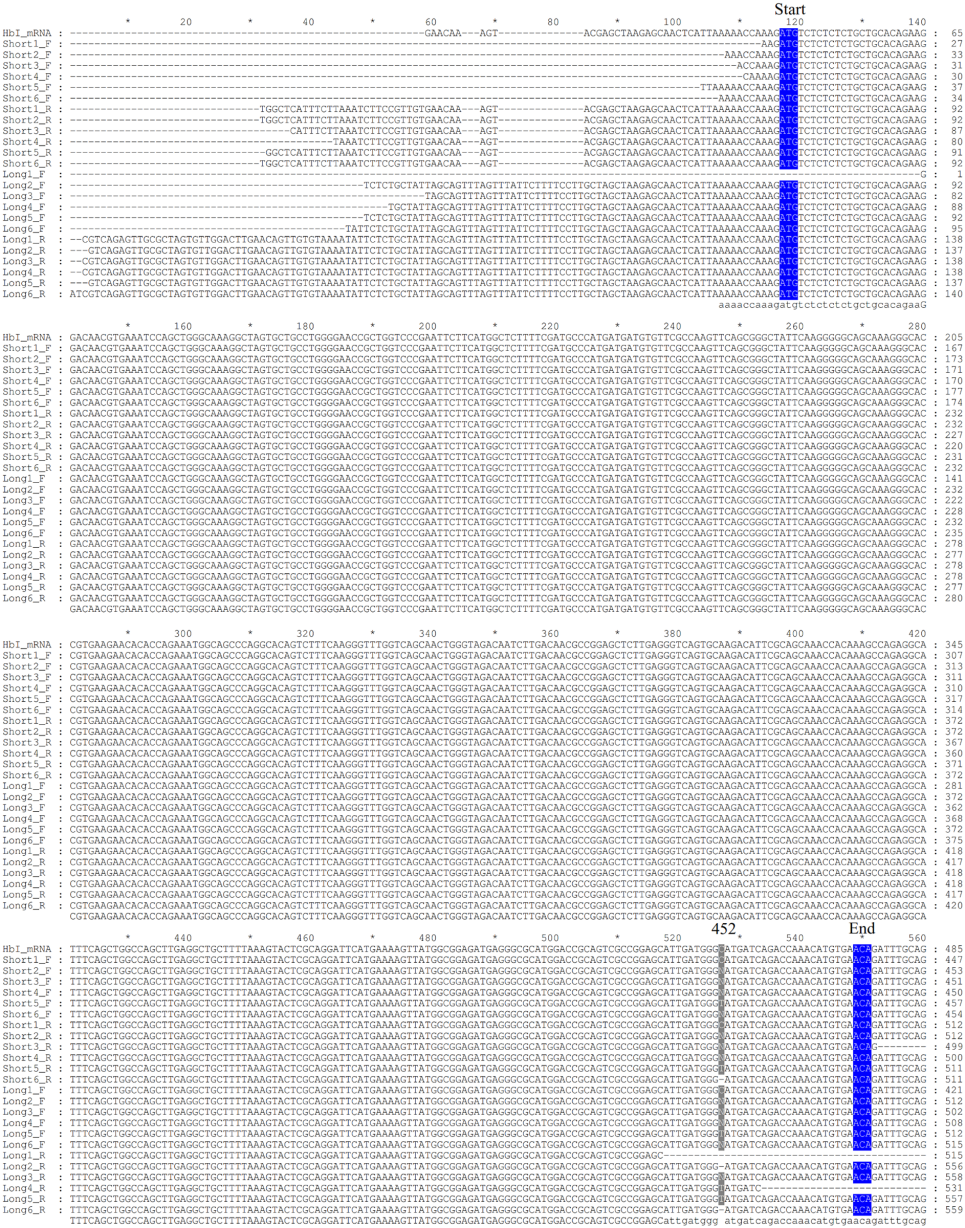
Alignment of HbI cDNA sequences obtained by RT-PCR. HbI cDNA was amplified with forwards primers designed from short and long variant and a common reverse primer, the sequences are named short and long, respectively. Samples from 1 to 3 correspond to RNA isolated from clams kept in a fish tank and samples 4 to 6 correspond to RNA isolated from clams harvested from their natural environment. F indicates that the sequence was obtained with the corresponding forward primer used for the cDNA amplification and R indicates that the sequence was obtained with the reverse primer. The first three bases coding for the first amino acid at the protein level are marked as start and the last marked as end, both highlighted in blue. Each position that presented different nucleotides are indicated by the number of this position in the reported mRNA for each Hb. The alignments were generated using Clustal W [23] and visualized and formatted with GeneDoc [35].

**Fig 10:**
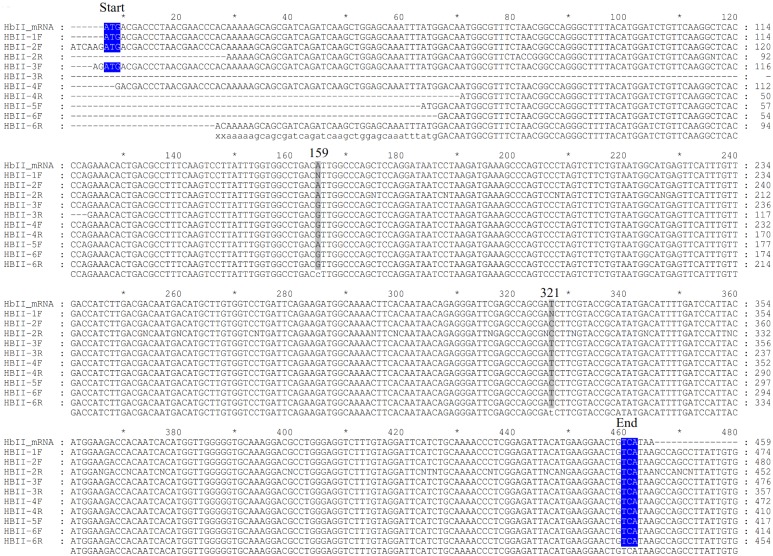
Alignment of HbII cDNA sequences obtained by RT-PCR. Samples from 1 to 3 correspond to RNA isolated from clams kept in a fish tank and samples 4 to 6 correspond to RNA isolated from clams harvested from their natural environment. F indicates that the sequence was obtained with the corresponding forward primer used for the cDNA amplification and R indicates that the sequence was obtained with the reverse primer. The first three bases coding for the first amino acid at the protein level are marked as start and the last marked as end, both highlighted in blue. Each position that presented different nucleotides are indicated by the number of this position in the reported mRNA for each Hb. The alignments were generated using Clustal W [23] and visualized and formatted with GeneDoc [35].

**Fig 11:**
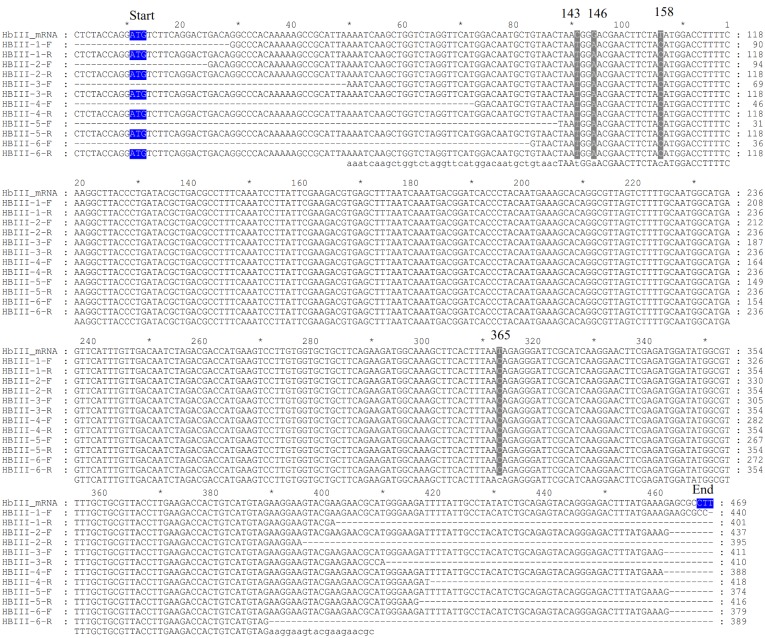
Alignment of HbIII cDNA sequences obtained by RT-PCR. Samples from 1 to 3 correspond to RNA isolated from clams kept in a fish tank and samples 4 to 6 correspond to RNA isolated from clams harvested from their natural environment. F indicates that the sequence was obtained with the corresponding forward primer used for the cDNA amplification and R indicates that the sequence was obtained with the reverse primer. The first three bases coding for the first amino acid at the protein level are marked as start and the last marked as end, both highlighted in blue. Each position that presented different nucleotides are indicated by the number of this position in the reported mRNA for each Hb. The alignments were generated using Clustal W [23] and visualized and formatted with GeneDoc [35].

We amplified the cDNA of the three *L*.*pectinata’*s Hbs from RNA isolated from clams subjected to different environments in order to verify if the environment influenced the mRNA integrity or stability. We analyzed three biological replicates per environment (6 samples) for the HbI_SV, HbI_LV, HbII and HbIII. Sequence analysis of the amplified cDNA of *Lucina pectinata’s* Hbs confirmed their reported cDNA sequence. Both HbI mRNA variants, short and long variant, have the same coding region. The HbII and HbIII cDNA sequences were also confirmed. A multiple sequence alignment for each Hb was generated with all of the sequences obtained and the reported mRNA (accession numbers: AF187049.1, AY243364.1 and EU040120.1 for HbI, HbII and HbIII respectively), shown in Figs 9–11 for HbI, HbII and HbIII, respectively. For HbI, there is one position in HbI transcript, position 452 in the reported HbI mRNA, (in both the short and long variant) that can be either a cytosine or a thymine (C/T). For HbI, at the gene level this position is a C, base 87 in exon 4. For HbII we also observed that the coding region positions 159 and position 321 (positions in the reported mRNA) can be either adenine or guanine (A/G) and C/T, respectively. For HbII, at the gene level these positions are A and T, bases 61 and 223 in exon 3, respectively. For HbIII we observed that the coding region positions 143, 146, 158 and 365 are C, G, T and T, but all the sequences obtained are T, A, C and C in these positions, respectively. For HbIII, at the gene level these positions are T, A, C and C for bases 104, 107, 119 in exon 2 and 202 in exon 3, respectively.

## Discussion

### Determination of *Lucina pectinata* gene structures

Many vertebrate globin genes have a 3-exons/2-introns gene structure, with conserved intron insertion positions at B12.2 (between the second and third base of the 12^th^ codon in helix B of the globin) and G7.0 [48]. In the case of HbII and HbIII genes of *L*. *pectinata*, their gene structure is a 4-exons/3-introns with two intron insertions at the conserved positions B12.2 and G7.0, while the third is a pre-coding intron. Comparison with other mollusk genes having pre-coding or A-helix introns, as well as introns after a signal sequence, showed similar gene structure with conservation of intron insertion at positions B12.2 and G7.0. These have been observed in genes coding for didomain hemoglobins such as the extracellular Hbs from *Pseudoterranova decipiens* and *Ascaris suum* and the intracellular hemoglobins of *Barbatia reeveana* (*B*. *reeveana*) and *Yoldia eightsi* [49–51]. *B*. *reeveana*, for example, expresses two types of hemoglobins in erythrocytes, a tetramer of α and β chains and a polymer consisting of a two-domain chain. The gene structure for the two-domain hemoglobin contained a pre-coding intron in addition to two conventional introns at positions B12.2 and G7.0. The equivalence between the 3’ end of pre-coding and bridge introns for the two-domain globin indicate that the bridge intron arose by unequal crossing over between two genes for a single-domain globin [50]. A-helix introns have also been described in the *Biomphalaria glabrata* (*B*. galabarta) myoglobin (Mb) gene, in the *Yoldia eightsi* monomeric hemoglobin and the homodimeric hemoglobins of *Calyptogena soyoae* [51–54].

On the other hand, HbI shows a more complex gene structure, possibly having 2 alternate first exons and 4 introns, with two introns with insertions at the conserved positions B12.2 and G7.0 and two pre-coding introns. We propose that in the HbI gene, the longer exon 1_L_ precedes the shorter exon 1_S_, since exon 1_S_ has similar size to exon 1 of both HbII and HbIII. According to this proposed model, the HbI gene would have two pre-coding introns: the characterized promoter region preceding exon 1_S_ being intron 1_b_ and the intron preceding exon 2 being intron 1_a_ (see Fig 1C).

### Confirmation of 5’ end of HbI cDNA by 5’- RACE method

The 5’RACE method revealed that there are two mRNA for HbI differing in the 5’UTR region and both sharing the first 29 nt of exon 2. These variants, 37 nt for the shorter and 96 nt for the longer, have not been yet characterized at the gene level, we propose that each variant corresponds to alternate exons 1_S_ and 1_L_, which remains to be found in the *L*. *pectinata* genome. The different alternative splicing (AS) mechanisms that can generate different mRNA isoforms from the same gene are: cassette exons, mutually exclusive exons, intron retention, alternative 5’ or 3’splice sites, alternative promoters and alternative polyadenylation sites [55]. The identification of two mRNA isoforms coding for the same protein, with variability in the UTR regions, could be due to post-transcriptional or transcriptional regulation mechanisms. UTRs contain motifs capable of regulating mRNA transport outside the nucleus, subcellular localization, translational efficiency and stability [56]. The HbI mRNA 5’UTR variants may be a consequence of the use of an alternative promoter mechanism, since none of the 5’ end variants (37 nt (short) and 97 nt (long)) share a common sequence at the 3’ portion, which may suggest that each variant comes from a variable alternate first exon (exon 1_L_ for the long 96 nt variant and exon 1_S_ for the short 37 nt variant), that is likely preceded by a distinct promoter. The generation of a unique first exon is a process regulated by transcription instead of a post-transcriptional process (alternative splicing) [55]. Many genes have alternative promoters in order to regulate gene expression, since the level of transcription of each promoter is influenced by tissue specific transcription factors. The generation of mRNAs with different 5’UTR sequences (or leader sequences) can affect turnover or translation efficiency and some can form protein isoforms differing at the amino terminal region [57]. The use of alternative promoters is a mechanism observed for cell or developmental specific gene expression [39]. For example the gene BRCA1 produces two mRNA transcripts differing only in their 5’UTR sequence by the use of different promoters, α and β. The BRCA1 shortest transcript is highly expressed and efficiently translated in normal mammary glands, while the longer transcript is predominantly expressed in breast cancer tissues. Even though the BRCA1 longer transcript is highly expressed in breast cancer tissue, the level of translation and stability is affected by the presence of several AUGs upstream of the main ORF, resulting in an overall decrease of the protein level in cancer cells (due to 10-fold less translation efficiency than the shorter transcript) [58]. Another protein that produces multiple 5’UTR mRNA variants, coding for the same protein, by the use of multiple promoters is the human estrogen receptor R gene (ESR1). The alternative splicing and the use of multiple promoters to produce these variants are controlled in a tissue-specific manner [59].

### Determination of promoter regions of *L*. *pectinata* genes

We analyzed the promoter sequences obtained by the GW method. TATA boxes were identifies for HbI_SV, HbII and HbIII, but not for HbI_LV. The presence of a TATA box in the HbI_SV promoter and absence of a TATA box at the expected location in the HbI_LV promoter resembles the alternative promoters of the human hemoglobin γA gene. This gene has two alternative promoters, one containing a TATA box and the other lacking one, that are used during and after embryonic development, respectively [60]. The basal transcription apparatus was proposed to be recruited to different types of core promoters in a developmental stage-specific manner [39]. Since the HbI_SV is consistent with a pre-coding exon of similar size as those found in HbII and HbIII, it is not surprising to find that a TATA box is present in a similar position relative to the TSS. No CpG islands were found in any of these promoter sequences, this findings resemble the β-globin gene clusters of human and other mammals, where the lack of CpG islands precludes DNA methylation and leads to an open chromatin structure. In contrast, methylation plays a role in regulation of the α-globin gene clusters, which have CpG islands [48, 61].

The gene regulation mechanisms of the two oxygen-binding Hbs and the sulfide-reactive Hb are unknown. The TFBSs analysis provide some insight of the possible TFs that may be involved in the regulation of these globin genes such as RORα, ROX1, NF-E2, GLN3, MafG, GATA-1, GATA-2 and Oct-1, which require additional experiments to confirm them. In Table L in S1 File we described the location of the most relevant TFBSs predicted by these methods in HbII and HbIII promoters and their function. The most relevant TFBSs predicted for HbI_SV and HbI_LV promoters also include TF known to be involved in other globin genes regulation, these are summarize in Table M in S1 File with their location in HbI_SV and HbI_LV promoters and function. Despite the fact that the HbI_SV promoter has a TATA box located at similar distance like that of HbII and HbIII, the predicted TFBSs are different, while the HbI_LV promoter shares more similarities with the TFBS of HbII and HbIII. This may indicate that the HbI_LV promoter, HbII and HbIII genes have a similar predicted regulation mechanism. Interestingly, some of the TFBSs predicted for the HbI_LV, HbII and HbIII are transcription factors that are activated and act to induce the expression of target stress response genes. Furthermore, the prediction of a TFBS for NF-kappa B, which is directly regulated by H_2_S [62], may suggest that the environmental H_2_S levels may play a role in the regulation of expression of these globins.

### Comparative analysis of *L*. *pectinata* Hbs gene structures

The gene structures of *L*. *pectinata* hemoglobins have similarities as well as differences. The three hemoglobins of *L*. *pectinata* have pre-coding exons that represent 5’ UTR sequences (exon 1 of HbII and HbIII and the variable exon 1_L_ and exon 1_S_ for HbI). The 5’UTR sequences of these hemoglobins are found in exon 1 and the first bases of exon 2 (29 bp, 22 bp, and 23 bp for HbI, HbII and HbIII respectively) to give 5’UTR sequences of 66 bp /129 bp for S/L HbI variants, 62 bp for both HbII and HbIII. For the three Hbs, the size of exons 2 and 3 are similar to those previously reported for globins, while exon 4 size is more variable, 930 bp for HbI, 1680 bp for HbII and 484 bp for HbIII (see Fig 1A, 1B and 1C). The size of non-coding exon 1 for HbI-exon1_S_, HbII and HbIII are similar, 37 bp, 40 bp and 39 respectively. The primary translation product is encoded by exons 2 to 4 in the three genes. On the other hand, the intron sizes of these three genes differ considerably: intron 1 of HbII (2,128 bp) is 958 bp longer than intron 1 of HbIII (1,170); HbI has the smallest intron 2 (698 bp) followed by HbIII (933 bp) and HbII (2,666 bp); intron 3 is less variable for the three hemoglobins having a size of 1,517 bp, 1,261 bp and 1,298 bp for HbI, HbII and HbIII, respectively. Intron length has proven to be an important determinant in the rate of RNA processing, longer genes having longer introns exhibit delay of expression [63]. In Table 2, we show the comparison of globin gene sequences having pre-coding or A-helix introns with their corresponding intron splice donor sites. All splice junctions follow the 5’GT/3’AG splice rule, except the 3’ splice acceptor preceding exon 2 of HbI which has a 3’CC acceptor site. Bioinformatic analysis of splice sites with mammalian genomes revealed that 99.24% followed the 5’GT/3’AG, 0.69% 5’GC/3’AG, 0.05% 5’AT/3’AC and only 0.02% have different non-canonical splice sites [64]. According to the analysis of 43,337 spliced junction pairs studied, at least 20 were annotated at GenBank having CC as 3’ splice acceptor. Nevertheless, the position of the pre-coding introns relative to the start codon differs from that of other mollusk genes described. Pre-coding introns were present at 29, 22, 23 bp before the start codons for HbI, HbII and HbIII respectively, while pre-coding introns identified in the hemoglobin genes of *Barbatia reeveana*, *Barbatia lima*, *Barbatia viriscens* and *Anadara trapezia* were been found to be 1 to 2 bases upstream of the start codon [50, 51, 53].

**Table 2:** Intron Positions and splice junction sequences in globin genes of *Lucina pectinata*, *Calyptogena soyoae*, *Barbatia reeveana*, *Biomphalaria glabrata* and *Anadara trapezia*.

Globin	Intron	Position	Size (bp)	Splicing donor (A/C)AGgt (a/g) agt	Splicing acceptor (c/t)_rich_n(c/t) agG/A
*Lucina pectinata* (this work)					
HbI	Intron 1_a/b_ (partial)	Pre-coding	?	?	tttt**cc**AGC
HbI	Intron 2	B12.2	698	GGC**gt**gagt	cgac**ag**TCT
HbI	Intron 3	G7.0	1517	CGG**gt**aggt	tttt**ag**GCT
HbII	Intron 1	Pre-coding	2128	CTT**gt**aagt	ttac**ag**AGA
HbII	Intron 2	B12.2	2666	GGA**gt**aaga	acac**ag**TCT
HbII	Intron 3	G7.0	1261	CGT**gt**atgt	cttc**ag**ACC
HbIII	Intron 1	Pre-coding	1170	GCA**gt**gagt	ttat**ag**AAA
HbIII	Intron 2	B12.2	933	GGA**gt**aaga	ttgc**ag**CCT
HbIII	Intron 3	G7.0	1298	CGA**gt**atgc	ctgc**ag**GAT
*Calyptogena soyoae* ^[^^54^^]^					
HbII	Intron 1	A1.3	594	AAG**gt**aaaa	tttc**ag**CCG
HbII	Intron 2	B12.2	312	AAC**gt**atgt	ttgc**ag**CCT
HbII	Intron 3	G7.0	250	GCG**gt**aagt	tttt**ag**ATT
HbI	Intron 1	A1.3	329	CCA**gt**aagt	tttt**ag**ATG
HbI	Intron 2	B12.2	672	CAA**gt**aatt	tttt**ag**GCT
HbI	Intron 3	G7.0	382	GCG**gt**aggt	tttc**ag**ATT
*Barbatia reeveana* ^[^^50^^]^					
2-domain globin (first domain)	Intron 1	Pre-coding	3115	AAC**gt**aagt	ttct**ag**AAT
2-domain globin (first domain)	Intron 2	B12.2	1494	TTT**gt**aagt	tcat**ag**ATT
2-domain globin (first domain)	Intron 3	G7.0	2175	GGT**gt**aagc	tttt**ag**TGG
*Biomphalaria glabrata* ^[^^52^^]^					
Myoglobin	Intron 1	A3.2	1116	TGC**gt**aagt	taac**ag**TGA
Myoglobin	Intron 2	B12.2	581	GTG**gt**gagt	caaa**ag**GAT
Myoglobin	Intron 3	G7.0	1008	GGA**gt**aagt	tttc**ag**CCT
*Anadara trapezia* ^[^^53^^]^					
Minor Globin	Intron 1	Pre-coding	?	?	ttc**ag**AAT
Minor Globin	Intron 2	B12.2	1213	GAA**gt**aagt	tcgt**ag**TCT
Minor Globin	Intron 3	G7.0	1435	GGG**gt**aagt	tttt**ag**AAA

When the three Hbs were compared, we found repetitive regions between HbI and HbII gene sequences, which resulted in three consensus sequences. From these three sequences, consensus sequence 1 have characteristics of a type of transposable elements known as MITEs, similar to the MITE-adh, type B element found in *Oryza sativa*. We also performed blastn searches against the mollusk database with the three consensus sequences as query and find similarities between these sequences with other mollusk genomes. Even though more information is needed in order to categorize the repeats found at intronic regions of *L*. *pectinata*’s Hbs genes, this analysis may suggests that these are common intersperse elements that have been, to some extent, conserved across the mollusk taxa.

Transposable elements (TE) are large components of most eukaryotic genomes and are thought to contribute to genome evolution and genetic plasticity [65, 66]. The occurrence of transposable elements within eukaryotic introns are thought to be one of the causes for the increase of genome size and a decrease of genome compactness during evolution [67]. They may have diverse genomic functions, such as: basic transcription, regulation at transcriptional and post-transcriptional levels, chromatin and nuclear organization, genome transmission at cell division, damage repair and DNA restructuring [68]. MITEs are short non-autonomous derivatives of full-length transposons, capable of transposing into different sites [42, 69]. In plants, they are mostly found within gene regions [70, 71] and subsequently their sequences are transcribed. For *Oryza sativa*, MITEs generate 23.5% of all small RNAs identified in the rice genome [42], some of them generated from MITE’s terminals and other generated from the central region. Lu *et al*. showed that genes with MITE insertions in upstream, intron, and downstream have significantly lower expression than genes away from MITEs, since these genes are small RNAs potential targets. It has also been demonstrated that the presence of MITE elements in the promoter region of a gene may affect gene regulation [72, 73]. However, genome-wide comparison showed that MITE insertions in a genome have significantly lower expression than genes with no MITE insertions [74]. As previously shown, for *L*. *pectinata*, a MITE-like element (Consensus sequence 1) is found in HbII (promoter region and in all introns) and HbI (HbI_SV promoter region and in introns 1 and 2), while HbIII does not have this element, see Fig 1A and 1C.

### Real time RT-PCR analysis for the three hemoglobins and 5’end HbI mRNA variants of *L*. *pectinata*: H_2_S-rich environment vs. H_2_S-poor environment

We used Real Time PCR in order to compare the *L*. *pectinata*’s Hbs expression in two groups of clams in different environments. The first group was harvested from their natural environment, a sulfide-enriched sediment as a consequence of anaerobic degradation processes that takes place in the mangrove swamp [75]. We found that the mRNA for the three Hbs was expressed in all five tissues examined. These results contrast to the prevalent thought that oxygen-reactive Hbs (HbII and HbIII) are widely distributed and that the sulfide-reactive hemoglobin (HbI) is only present in the tissue where the endosymbiont is located [1]. The three Hbs mRNAs are highly expressed in ctenidia tissue (see Fig 7), which was expected since it has been shown that *L*. *pectinata* has the highest concentrations of Hbs in its gills, estimated at 1.5mmol/kg wet weight [76]. HbI has a significantly higher expression (3 fold) than HbII, which shows the lowest expression level in this tissue. On the other hand, HbIII appears to have the highest mRNA expression in ctenidia but, due to the high expression variability encountered for this gene in the biological replicates, there is no significant difference between the expression of HbIII with respect to HbI and HbII. Furthermore, there is no significant difference in the expression of HbIII across all five tissues due to this variability. It is known that HbII and HbIII can form dimers and tetramers in a concentration depending manner [1]. The crystal structures of HbII homodimers and HbII-HbIII heterodimer have been resolved [12, 13]. Taking this into consideration, the expression analysis correlates with the protein elution profile observed in size exclusion chromatography using crude extracts of ctenidia tissue, where the HbI absorbance peak had lower intensity than the one corresponding to that for the HbII-HbIII dimer [11]. For HbI and HbII, a significant decrease on expression levels is observed when their expression in ctenidia tissue is compared with their expression in foot, mantle, muscle and visceral mass tissues. In general, HbII seems to have the lowest expression level in all tissues but there is no significant difference when compared to the expression of HbI and HbIII, with the exception of the foot tissue of those clams from sulfide-rich environment, where HbIII shows higher expression (7-fold) than HbII.

When comparing the mRNA expression values obtained in different tissues for the two groups it is apparent that the three Hbs mRNAs remains highly expressed in ctenidia tissue and they have lower expression levels in mantle, muscle, and visceral mass. However, expression of these Hbs in ctenidia tissue from clams kept in a fish tank seems to be down regulated, a significant difference was observed for HbI and HbII which are expressed about 5 and 6 times more, respectively, in clams harvested from their natural environment. Meanwhile, HbIII expression in ctenidia is not significantly different between groups. For the other four tissues, the Hbs expression levels where comparable for both environments and showed no statistical difference. It has been postulated that the physiological roles of *L*. *pectinata* hemoglobins in ctenidia tissue is the delivery of O_2_ and H_2_S to the symbionts [11] localized in specialized organelles known as bacteriocytes. *Codakia orbicularis*, another member of the *Lucinidae* family, showed a significant decrease in symbiont population after three months of being subjected to starvation in artificial sea water, suggesting that the host used symbiont digestion as a nutritional source or that symbionts were enzymatically autolysed [77]. However, a relationship between Hbs expression levels and symbiont population size has not been established. The HbI transcript was detected in ctenidia tissues despite the fact that the clams were in a sulfide-poor environment. Vetter (1985) confirmed the presence of sulfide globules that served as a source of inorganic energy when environmental sulfide levels are low in the three symbiont harboring organism, *Lucinoma annulata*, *Calyptogena elongata* and *Lucina floridana* [78]. Similar vesicles containing elemental sulfur were characterized in *L*. *pectinata* gill tissue, suggesting that these structures may also serve as energy source for the endosymbiont in case of H_2_S depletion [79]. It is not known whether HbI may interact with the sulfur contained in these storage vesicles for delivery to the symbiont. On the other hand, this also suggests that clam Hbs gene expression may be subject to environmental control, which may include response to changes in O_2_ and H_2_S concentration, however there may be other environmental factors influencing the expression of these Hbs.

As mentioned before, we speculate that these alternative 5’UTR mRNA variants are the result of an alternative promoter mechanism which can have different tissue specificity and can react differently to signals. Both HbI mRNA variants are expressed, but the shorter one was always found in lesser amounts. The lower expression of the shorter variant in the sulfide-poor environment is double the decrease of expression of the longer variant. Whether or not the expression of these two variants is related to the symbiosis remains to be tested.

### Reverse transcription PCR analysis for HbI mRNA variants, HbII and HbIII in ctenidia tissue: H_2_S-rich environment vs. H_2_S-poor environment

The cDNA amplification of these three Hbs using RNA isolated from clams in the two conditions tested showed that for all Hbs there are positions that presented substitutions. Nonetheless, these changes in the mRNA are synonymous substitutions. When the sequences from different environments were compared no correlation was found between the nucleotides changes and the environment. Some of the differences between the gene and mRNA are similar to the substitutions caused by the known RNA editing mechanisms. The substitutional RNA editing observed in these sequences are Adenosine to Inosine (A-to-I), which are interpreted as a Guanine, and Cytosine to Uracil (C-to-U), interpreted as a Thymine. A-to-I RNA editing is mediated by a family of adenosine deaminases acting on double-stranded RNA (ADARs), this mechanism is confined to unspliced transcripts [80]. C-to-U requires two proteins, the deaminase Apolipoprotein-b-mRNA-editing-enzyme-1 (APOBEC1) and a cofactor that is believed to be the RNA-binding protein (RBP) APOBEC1-Complementation-Factor. This process is confined to spliced and polyadenylated nuclear transcripts [81]. It has been shown, for some invertebrates, that RNA editing is a common occurrence, for example the majority of the squid mRNAs harbor one or more editing sites [82]. RNA editing occurs in the coding region of the squid Na^+^/K^+^ ATPase α-subunit mRNAs changing the pump intrinsic voltage dependence, accelerating Na^+^ release to the extracellular medium [82, 83]. For the octopus mRNAs encoding Kv1.1 potassium channels, editing seems to be correlated to environmental temperature. Recoding an isoleucine to a valine makes the channel close faster for arctic and Antarctic species, while tropical octopuses edit this position at low levels [82]. However, another possibility for the detected base changes is the presence of different alleles, representing single nucleotide polymorphisms. On the other hand, the enzyme mix used for this assay, the Omniscript and Sensiscript Reverse Transcriptases from the One^®^Step RT-PCR kit from QIAGEN, are designed to provide highly efficient and specific reverse transcription, so we do not consider that these base changes are due to PCR errors.

## Conclusions

Studies of hemoglobins have established two general principles: that all globin genes are derived from a common ancestor and that hemoglobins can serve additional functions beyond transporting O_2_ between tissues. The different hemoglobin functions illustrate the acquisition of new roles of a pre-existing structural gene, an acquisition that requires changes not only in the coding regions but also in the regulatory elements of the genes [61] in order to adapt to different living conditions. Genome evolution may involve rearrangement of existing genes, and new proteins probably arose in the course of evolution by the shuffling of exons [61]. For polypeptide-encoding genes, the alternative combinations of coding exons or variant coding exons result in amino acid differences with important functional consequences. In the case of noncoding exons, it may result in different 5’ or 3’ untranslated sequences and sometimes different polyadenylation sites, which may influence mRNA stability and/or translation efficiency. The *L*. *pectinata* HbIII gene has been shown to be subject to this type of mechanism by which different forms of mature mRNAs result from an alternative polyadenylation site that could be associated with regulatory mechanisms at the mRNA and/or protein expression levels [16, 56, 84, 85]. In this work we show that HbI has two mRNA variants differing in the 5’UTR region, which we suggest are a consequence of an alternate promoter mechanism.

For some mollusks, it is common to have a 4-exon/3-intron globin gene structure, having two introns that are highly conserved in animal globin genes and an additional pre-coding intron [86]. HbII and HbIII gene structures present a 4-exon/3-intron gene structure, with a pre-coding intron and two introns at conserved insertion positions B12.2 and G7.0. HbI gene presents a more complex structure with the possibility of having a 5-exon/4-intron gene structure. Nevertheless, HbI gene also have introns at conserved positions B12.2 and G.7, the other two introns are pre-coding. An ancestral globin gene structure having 4-exon/3-intron structure, interrupting the B (B12.2), E (E15.0) and G (G7.0) helices, have been proposed [87, 88]. This ancestral gene structure appears to have been retained in plant and nematode hemoglobins, which have two introns in homologous positions that those found in vertebrates and also have a central intron that interrupts the coding region for the E helix [49, 89, 90]. It has been proposed that all other gene structures have evolved from this ancestral gene by the loss of this central intron [91]. *L*. *pectinata*’s hemoglobins genes retained introns at the ancient positions B12.2 and G7.0, lack introns at E helix coding region and have pre-coding introns, which may be products of intron loss and intron gain events [89]. The conservation of intron locations in the coding region is further evidence of the conservation of globin gene structure in invertebrate hemoglobins. We also observed synonymous substitutions in several positions of the cDNAs of the three Hbs when comparing replicates. These kind of substitutions can be explained by RNA editing mechanism even thou this mechanism is mostly associated to protein modification. Further studies are necessary to understand the purpose of these changes.

We also characterized a repetitive region (consensus sequence 1) across HbI and HbII promoter and intronic regions that share characteristics of transposable elements known as miniature inverted-repeat transposable elements (MITEs), a group of non-autonomous class II transposable elements.

The promoter region analysis of these two hemoglobins suggested the presence of several TFBSs for TFs that have been reported to regulate globin genes as well as TFs that regulate genes when the cells are under environmental stress. The presence of such TFBSs may be indicative of regulation mechanisms of *L*. *pectinata*’s hemoglobins that allow this clam to thrive in a sulfide-rich environment. The gene expression analysis of the three Hbs comparing sulfide-rich and sulfide-poor environments showed a significant decrease of expression of the hemoglobins in the symbiont-containing tissue for those clams in a sulfide-poor environment. The observed expression profile of *L*. *pectinata’s* Hbs suggests that a change in environmental sulfide concentration may be involved in the regulation of these proteins, but other factors cannot be ruled out. The HbII transcript has the lowest expression for both environmental conditions in all tissues. One factor that may contribute to HbII low expression may be that the HbII gene has longer introns than HbI an HbIII and, as previously stated, genes with longer introns have lower transcription rates. Furthermore, the presence of the sulfide-reactive HbI in all the tissues examined, for both studied environmental conditions, suggest that this hemoglobin may be involved in the process of protecting cellular respiration from sulfide toxicity, which requires further studies. The evaluation of gene expression of the two HbI mRNA variants suggests that the longer variant is expressed at comparable levels of that of HbII and HbIII in both environments, while the shorter variant expression is significantly lower in both environments. This supports our hypothesis that the HbI longer variant, as well as the HbII and HbIII genes share similarities in TFBSs predicted in their promoters, and may have similar regulation. On the other hand, the HbI_SV promoter had different predicted TFBSs which may indicate different regulation mechanisms. The HbII and HbI_SV promoters possess a MITE-like element at similar positions which may be involved in the low expression of both transcripts, but other possible mechanisms may be proposed with further studies.

## Supporting Information

S1 Fig *Lucina pectinata* and tissues examined. **A**) Juvenile *Lucina pectinata* clam. **B)** Tissues of the clam *Lucina pectinata* examined in this study: **a**. Ctenidia. **b**. Mantle. **c**. Muscle. **d**. Foot. **e**. Visceral Mass.
(TIFF)

S2 Fig HbII and HbIII promoter sequences. **A)** HbII promoter sequence obtained by GW. **B)** HbIII promoter sequence obtained by GW.
(TIFF)

S3 Fig HbI Short and HbI Long variants promoter sequences. **A)** HbI Short Variant promoter sequence obtained by GW. **B)** HbI Short Long promoter sequence obtained by GW.
(TIFF)

S4 Fig Predicted TFBSs for HbI Short and HbI Long variants promoters. The Alibaba2.1 software was used to analyze the HbI Short variant (left) and HbI Long variant (right) promoter regions. The last nucleotide of each sequence corresponds to the -1 position from the TSS. The TF colored in green are those that were also predicted by the TFBIND program.
(TIFF)

S5 Fig Graphic representation of blastn results of repetitive sequences found in *L*. *pectinata*’s Hbs genes against the mollusk (taxid:6447) Genomic Reference Sequences Data Base. **A)** Using consensus sequence 1 as query; **B)** Using consensus sequence 2 as query; **C)** Using consensus sequence 3 as query. For each one of the alignments, the query sequence and its complementary strand are shown at the top. The organism name, scaffold and location are indicated by arrows next to the subject accession number.
(TIFF)

S1 File This file contains Supplementary Tables 1–13. **Table A.** Primers used in this work. **Table B.** LAGAND Aligned hits for HbII and HbIII promoter regions. **Table C.** TFBSs shared between HbII and HbIII motif 1 region. **Table D.** Predicted TFBSs for HbI Short Variant Promoter (>80%). **Table E.** Predicted TFBSs for HbI Long Variant Promoter (>80%). **Table F.** TFBSs predicted for motif 1 of HbI Short variant promoter using JAPAR CORE database. **Table G.** TFBSs predicted for motif 1 of HbI Long variant promoter using JAPAR CORE database. **Table H.** Tabulated Blastn results of HbII promoter-gene against HbI incomplete gene sequence. **Table I.** Tabulated Blastn results of Consensus sequence 1 against mollusk (taxid:6447) database reference genomic sequences. **Table J.** Tabulated Blastn results of Consensus sequence 2 against mollusk (taxid:6447) database reference genomic sequences. **Table K.** Tabulated Blastn results of Consensus sequence 3 against mollusk (taxid:6447) database reference genomic sequences. **Table L.** Most relevant TF predicted for HbII and HbIII promoters with their location relative to the TSS and function. **Table M.** Most relevant TF predicted for HbI_SV and HbI_LV promoters with their location relative to the TSS and function.
(DOCX)

## Abbreviations

cDNA: complementary deoxyribonucleic acid
gDNA: genomic deoxyribonucleic acid
GW: genome walking
H_2_S: hydrogen sulfide
O_2_: Oxygen
nt: nucleotide
bp: base pair
Hb: hemoglobin
GSP: gene specific primer
TSP: Target specific primers
PCR: polymerase chain reaction
XL-PCR: extra-long polymerase chain reaction
HbI_SV: HbI short variant
HbI_LV: HbI long variant
TFBS: Transcription factor binding site
TSS: Transcription start site
TF: Transcription factor
MITE: miniature inverted-repeat transposable elements
PCR: polymerase chain reaction
RT-PCR: reverse transcription PCR
