# Supplementary figures and images for: Characterization and Expression of the Lucina pectinata Oxygen and Sulfide Binding Hemoglobin Genes
Source: PLoS One. 2016 Jan 29;11(1):e0147977. doi: 10.1371/journal.pone.0147977 (PMC4732748; doi:10.1371/journal.pone.0147977)

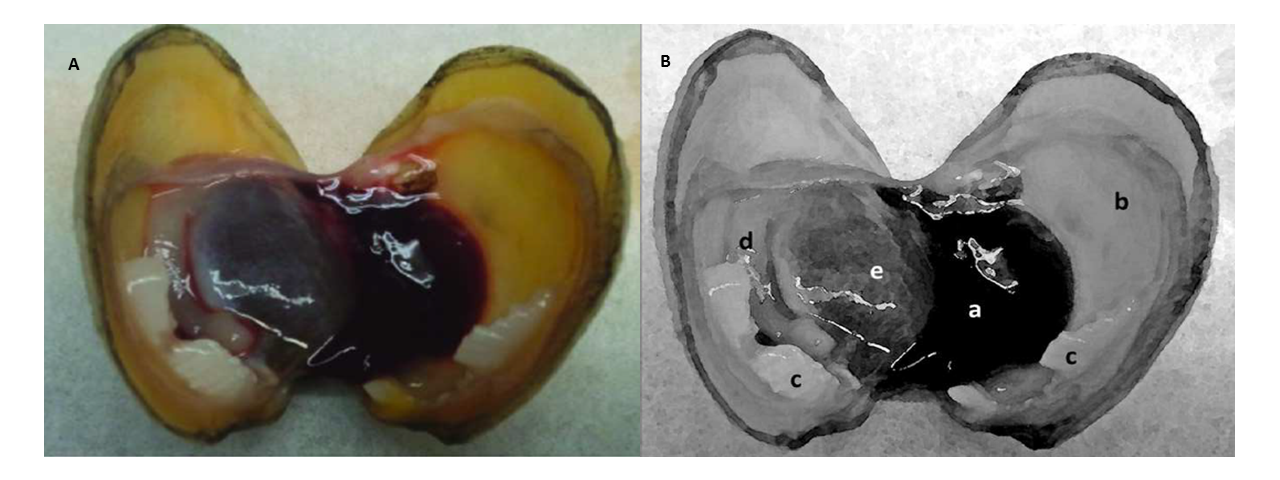

Supplement: S1 Fig — A) Juvenile Lucina pectinata clam. B) Tissues of the clam Lucina pectinata examined in this study: a. Ctenidia. b. Mantle. c. Muscle. d. Foot. e. Visceral Mass. (TIFF) [file pone.0147977.s001.tiff]

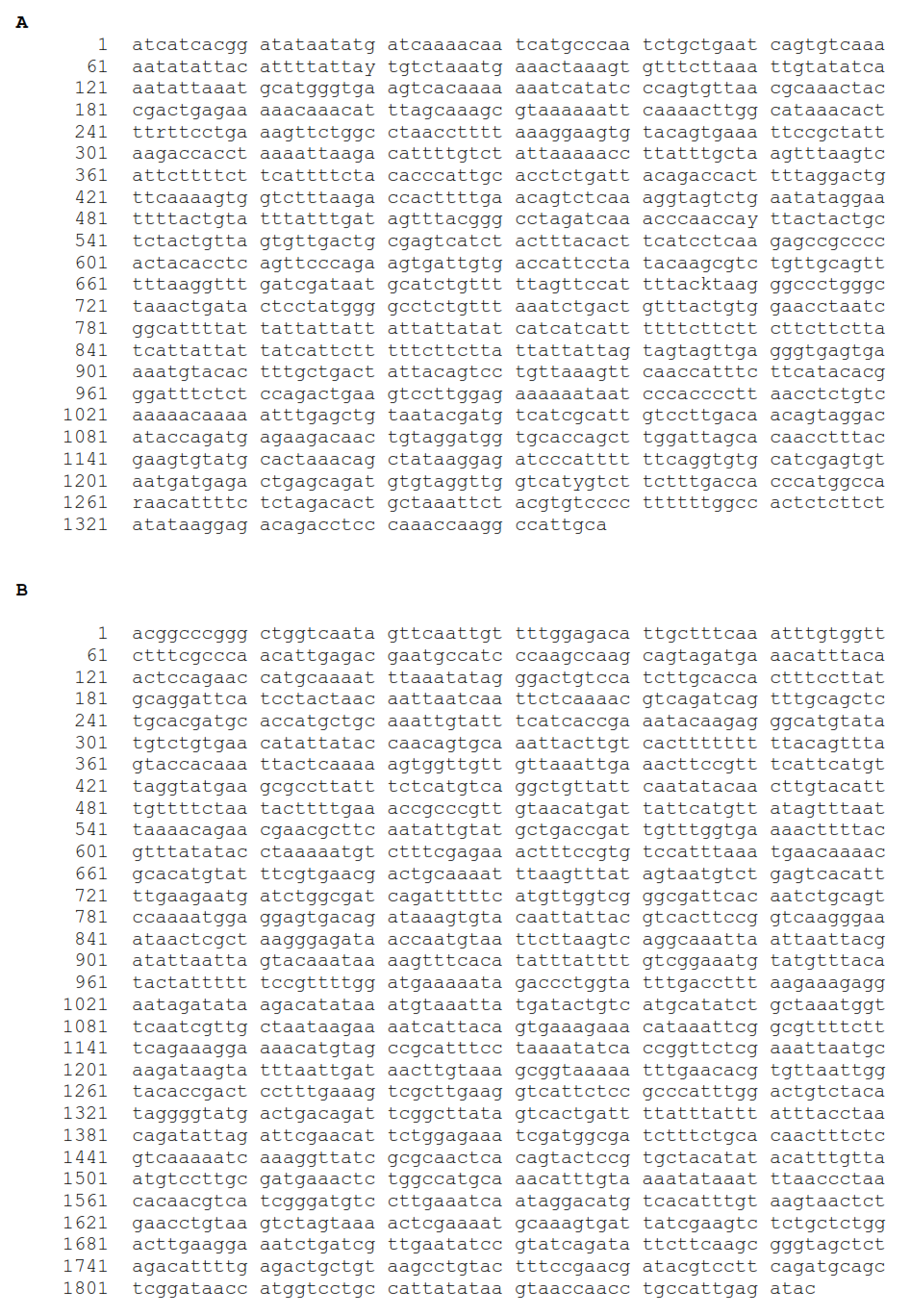

Supplement: S2 Fig — A) HbII promoter sequence obtained by GW. B) HbIII promoter sequence obtained by GW. (TIFF) [file pone.0147977.s002.tiff]

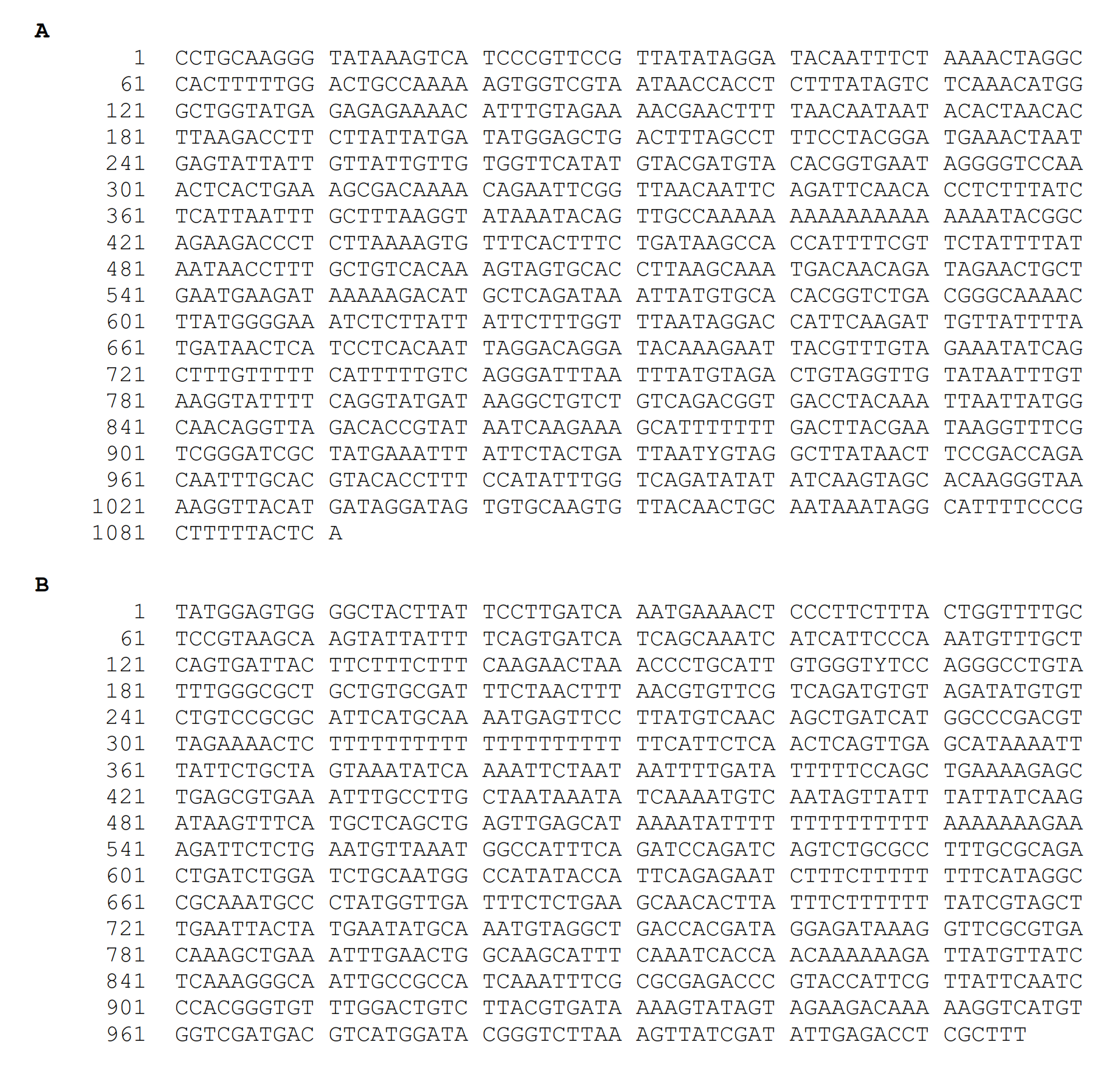

Supplement: S3 Fig — A) HbI Short Variant promoter sequence obtained by GW. B) HbI Short Long promoter sequence obtained by GW. (TIFF) [file pone.0147977.s003.tiff]

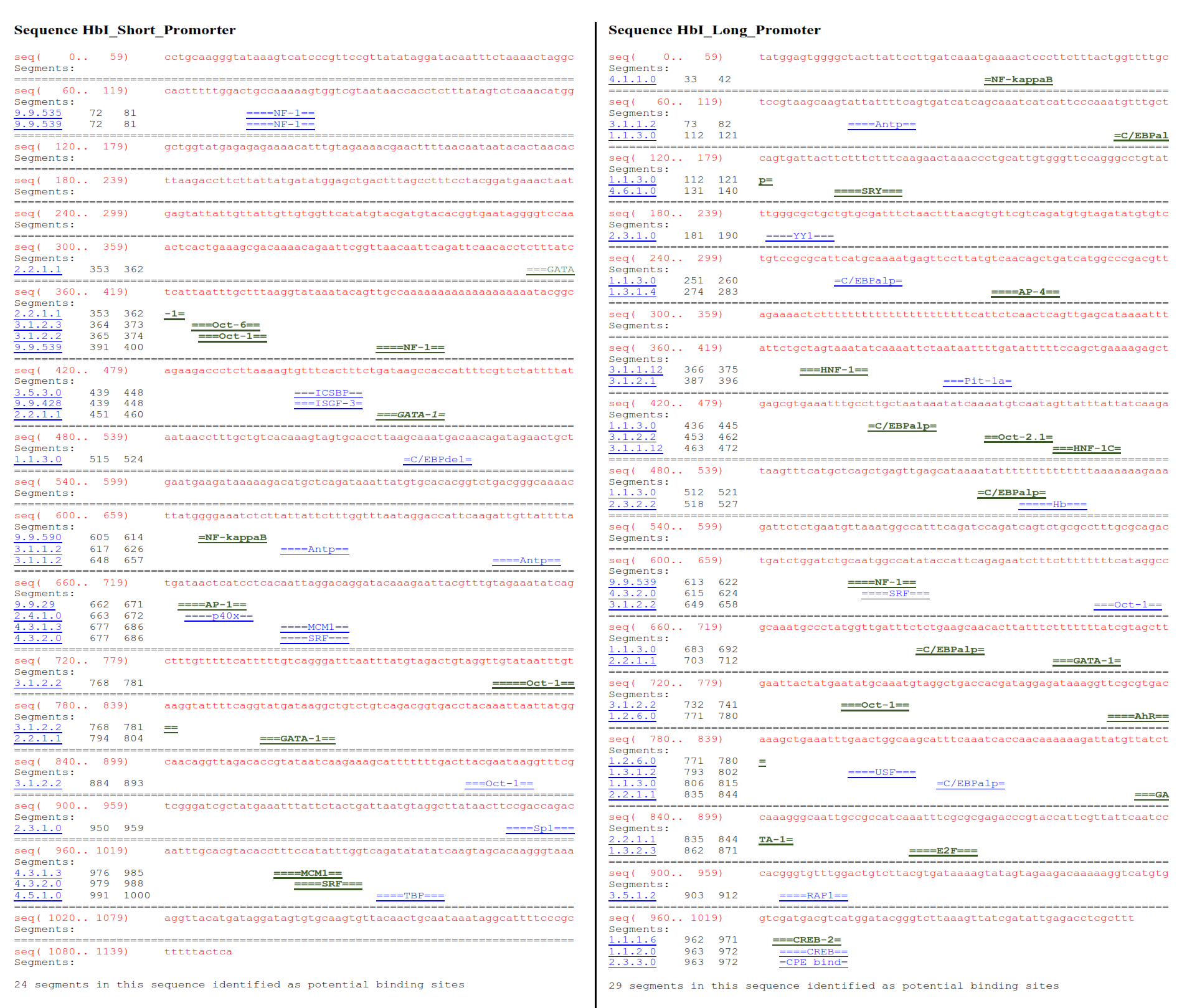

Supplement: S4 Fig — The Alibaba2.1 software was used to analyze the HbI Short variant (left) and HbI Long variant (right) promoter regions. The last nucleotide of each sequence corresponds to the -1 position from the TSS. The TF colored in green are those that were also predicted by the TFBIND program. (TIFF) [file pone.0147977.s004.tiff]

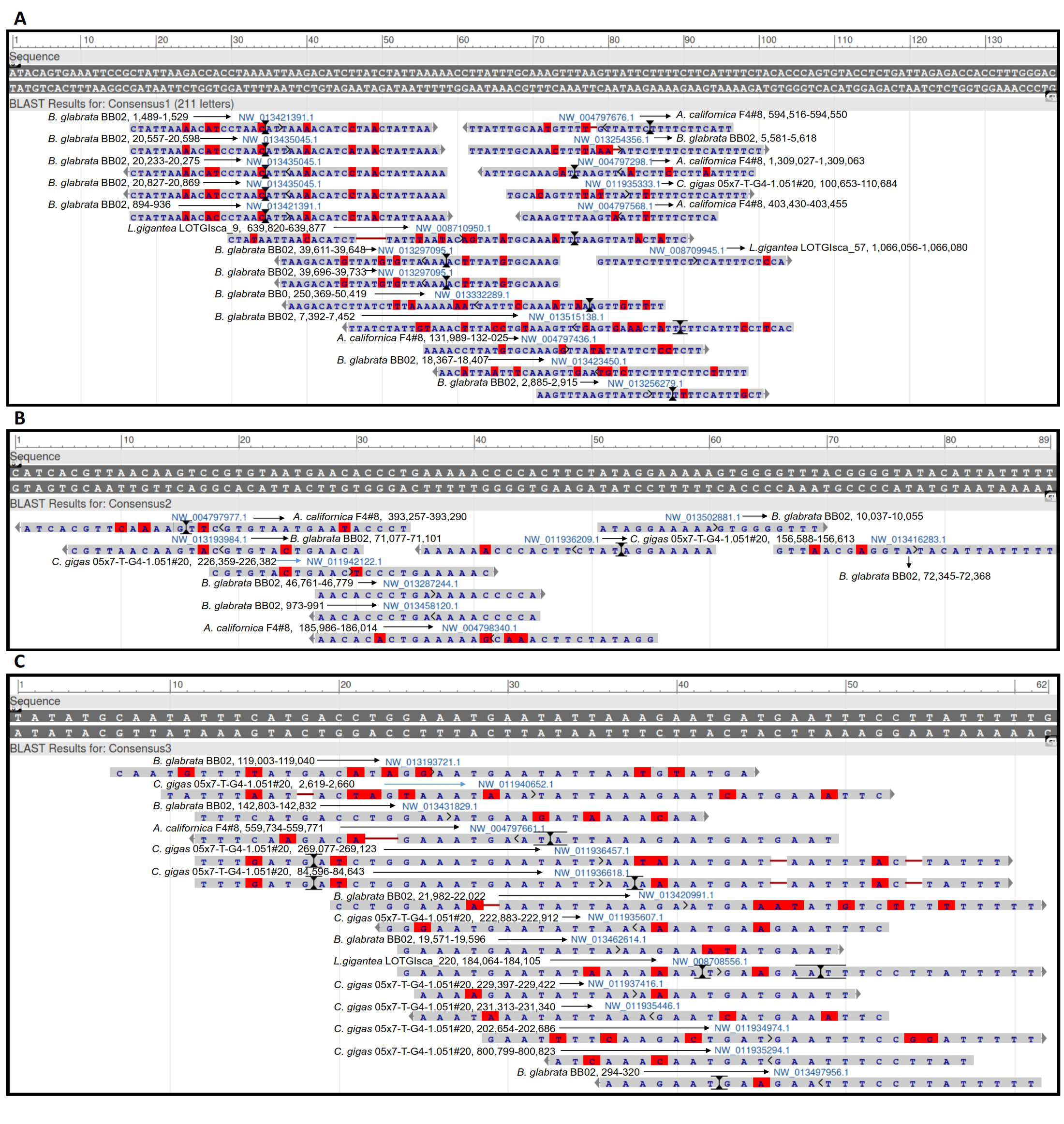

Supplement: S5 Fig — A) Using consensus sequence 1 as query; B) Using consensus sequence 2 as query; C) Using consensus sequence 3 as query. For each one of the alignments, the query sequence and its complementary strand are shown at the top. The organism name, scaffold and location are indicated by arrows next to the subject accession number. (TIFF) [file pone.0147977.s005.tiff]
