# Supplementary material for: Characterization and Expression of the Lucina pectinata Oxygen and Sulfide Binding Hemoglobin Genes
Source: PLoS One. 2016 Jan 29;11(1):e0147977. doi: 10.1371/journal.pone.0147977 (PMC4732748; doi:10.1371/journal.pone.0147977)
Supplement: S1 File — Table A. Primers used in this work. Table B. LAGAND Aligned hits for HbII and HbIII promoter regions. Table C. TFBSs shared between HbII and HbIII motif 1 region. Table D. Predicted TFBSs for HbI Short Variant Promoter (>80%). Table E. Predicted TFBSs for HbI Long Variant Promoter (>80%). Table F. TFBSs predicted for motif 1 of HbI Short variant promoter using JAPAR CORE database. Table G. TFBSs predicted for motif 1 of HbI Long variant promoter using JAPAR CORE database. Table H. Tabulated Blastn results of HbII promoter-gene against HbI incomplete gene sequence. Table I. Tabulated Blastn results of Consensus sequence 1 against mollusk (taxid:6447) database reference genomic sequences. Table J. Tabulated Blastn results of Consensus sequence 2 against mollusk (taxid:6447) database reference genomic sequences. Table K. Tabulated Blastn results of Consensus sequence 3 against mollusk (taxid:6447) database reference genomic sequences. Table L. Most relevant TF predicted for HbII and HbIII promoters with their location relative to the TSS and function. Table M. Most relevant TF predicted for HbI_SV and HbI_LV promoters with their location relative to the TSS and function. (DOCX) [file pone.0147977.s006.docx]

**Table A.** Primers used in this work.

| Primers used to determine HbI, HbII and HbIII gene sequences | | | |
| --- | --- | --- | --- |
| Region | **GSP primer** | | **Sequence** |
| HbII Intron 1 | HbIIUTR2 | 5’ TGA CAA TTT GCA GTA TAG ACC ATT CAG AGG 3’ | |
|  | HbIINUGW1R | 5’ ATC GCT GCT TTT TGT GGG TTC GTT A 3’ | |
| HbII Intron 2 | HbIIF1 | 5’ ATG ACG ACC CTA ACG AAC CCC ACA AAA G 3’ | |
|  | HbIIR1 | 5’ TCA TGC CAT TAC AGA AGA CTA GGG ACT GG 3’ | |
| HbII Intron 3 | HbIIF2 | 5’ ATC CTT GTG GT CCT GAT TCA GAA GAT GG 3’ | |
|  | HbIIR2 | 5’ CAT GTA ATC TCC GAG GGT TTT GCA GAT 3’ | |
| HbIII Introns 1 & 2 | HbIIIUGSP1F | 5’ TCG GTT TTG GGG TAC AGA CTT TAT CTG AGG AAC TGC 3’ | |
|  | HbIIITSP11R | 5’ TGC AAA AGA CTA ACG CCT GTG 3’ | |
| HbIII Intron 3 | HbIIITSP14F | 5’ GTC CTT GTG GTG CTG CTT CA 3’ | |
|  | HbIIITSP4R | 5’ AAA GGC GCT CTT TCA TAA AGT CTC 3’ | |
| HbII Segment1 | HbII2283F | 5’ TGG GCC GAG AAC TTT TTG ATC ATA CCT 3’ | |
|  | HbII3064R | 5’ ACG AAG ATC GCT GGC TCG AAT 3’ | |
| HbII Segment 2 | HbII3RACEF2 | 5’ ACA TTT TGA TCC ATT ACA TGG AA 3’ | |
|  | HbIIUTRRev2 | 5’ CGT TAG TAA ACC ACG CCC TTA AGT CAA ACC A 3’ | |
| HbII Segment 3 | HbII6295F | 5’ CTC ATC TGT GCT TTA TCT CGG GCA TT 3’ | |
|  | HbII6976R | 5’ CGT CAC CCA TAT CTA CAA AAG CAC GTG AG 3’ | |
| HbII Segment 4 | HbII1859F | 5’ TAT CCC ACC GCA GCG CAG TAA TAT AGC 3’ | |
|  | HbII2447R | 5’ ATC TTC TTT TCA GCA AGC CCC CTT CAG 3’ | |
| HbIII Segment 1 | HbIII2207fow | 5’ AAC CAC TTA TTG CAC GAA GCC TCC ATC T 3’ | |
|  | HbIII2647Rev | 5’ TGC GTC ATC TCT CTG TAC CTG CGT GTT AAG 3’ | |
| HbIII Segment 2 | HbIII2477fow | 5’ ATT CGC ATC AAG GAA CTT CG 3’ | |
|  | HbIII4255Rev | 5’ ACG TAC AAA GTC TAT CCC TAA GGG CAA TTC 3’ | |
| HbIII Segment 3 | HbIIITSP14F | 5’ GTC CTT GTG GTG CTG CTT CA 3’ | |
|  | HbIII3end | 5’ AAT GTG TAA TAG GAT TGT GTA ATA GGA TTC TCT T 3’ | |
| HbI Intron 2 | HbIF1 | 5’ TGC TGC ACA GAA GGA CAA CGT GAA A 3’ | |
|  | HbIR1 | 5’AGA GCT CCG GCG TTG TCA AGA TTG T 3’ | |
| HbI Intron 3 | HbIF2 | 5’ GGC ACC GTG AAG AAC ACA CCA GAA A 3’ | |
|  | HbIR2 | 5’ CGC CCT CAT CTC CGC CAT AAC TTT T 3’ | |
| HbI Segment 1 | HbIpromoter2F | 5’ GTG GGT ATT CGT TGG AAG CAA ACT AAC GAG 3’ | |
|  | HbIUGW1R | 5’ ATG AAG AAT TCG GGA CCA GCG GTT CCC 3’ | |
| HbI Segment 2 | HbIRACEF | 5’ AAG TTA TGG CGG AGA TGA GG GCG CA 3’ | |
|  | HbIUTR-R | 5’ TCC AAT ATG CAT TCC ACA TAC AAA C 3’ | |
| HbI Segment 3 | HbIOL3F | 5’ GAG GCT GCT TTA ACG AGC ACC CAG AGT 3’ | |
|  | HbIOL3R | 5’ CAG CCC ATT CTT CTC TTT GGC CTG TTC 3’ | |
| Confirmation of 5’ RACE products | HbIUTRF1 | 5’ GGT ATT CAG ATC GTC AGA GTT GCG CTA GTG TTG 3’ | |
|  | HbI.V1.F | 5’ GGC TCA TTC TTA AAT CTT CCG TTG 3’ | |
|  | HbIOL3R | 5’ CAG CCC ATT CTT CTC TTT GGC CTG TTC 3’ | |
| First genome walking | HbI NUGW1R | 5’ CAG CTG GAT TTC ACG TTG TCC TTC TGT 3’ | |
|  | HbI UGW1R | 5’ ATG AAG AAT TCG GGA CCA GCG GTT CC 3’ | |
| Second genome walking | GSP 1 | 5’ CAA AGG TGG TCT CAA ACA A 3’ | |
|  | GSP 2 | 5’ AGG TAG TCT TAA TAG CGG AAT TCC 3’ | |
| Third genome walking | GSP 1 | 5’ GGC TTC CCT AAA AAG GCT TGG AAA ATA 3’ | |
|  | GSP 2 | 5’ ATC AGG ACC AAA ATC GTC AGC AGC AGA TAG 3’ | |
| Confirmation of new sequence obtained by 2^nd^ genome walking | N1.HbI.FW | 5’ CGT TGA ACC TAC AGG GCT TTT 3’ | |
|  | N2.HbI.FW | 5’ CTG TAA TAC GGT TGC CAAT GTA CCA C3 ’ | |
|  | HbI.R1 | 5’ AGA GCT CCG GCG TTG TCA AGA TTG T 3’ | |
| HbII Promoter | HbII.GSP1.RV | 5’ TGC TGT AAG CAT CAC TTG TGA CAT TCT 3’ | |
|  | HbII.GSP2.RV | 5’ CAA GTT TCC TCT GAA TGG TCT ATA CTG CAA 3’ | |
| HbIII Promoter | HbIII.GSP1.RV | 5’ GTT ATG AGC AGA CTC CAG TGT CTT TGC 3’ | |
|  | HbIII.GSP2.RV | 5’ TCT TTG CCG GTC AAA AGA CAA CAT AG 3’ | |
| HbI Short Promoter | HbI.S.GSP1.RV | 5’ CGT ACT TTG TTC ACA ACG GAA GAT T 3’ | |
|  | HbI.S.GSP2.RV | 5’ CAC AAC GGA AGA TTT AAG AAT GAG C 3’ | |
| HbI Long Promoter | HbI.L.GSP1.RV | 5’ CTG TTC AAG TCC AAC ACT AGC GCA AC 3’ | |
|  | HbI.L.GSP2.RV | 5’ AGC GCA ACT CTG ACG ATC TGA ATA CC 3’ | |
| Primers used for the SYBR Green Real Time RT-PCR analysis | | | |
| Target | **Primer** | | **Sequence** |
| *L. pectinata* Hb I | Forward | | 5’CTC TTT TCG ATG CCC ATG AT 3’ |
|  | Reverse | | 5’AGT TGC TGA CCA AAC CCT TG 3’ |
| *L. pectinata* Hb II | Forward | | 5’ACG GCC AGG GCT TTT ACA T 3’ |
|  | Reverse | | 5’TTC AAG TCC TTA TTT GGT GGC C 3’ |
| *L. pectinata* Hb III | Forward | | 5’CAA TGA AAG CAC AGG CGT TA 3’ |
|  | Reverse | | 5’CGC CAT ATC CAT CTC GAA GT 3’ |
| *L. pectinata* HbI _Long Variant | Forward | | 5’GGT ATT CAG ATC GTC AGA GTT GCG CTA GTG TTG 3’ |
|  | Reverse | | 5’CAG CCC ATT CTT CTC TTT GGC CTG TTC 3’ |
| *L. pectinata* HbI _Short Variant | Forward | | 5’GGC TCA TTC TTA AAT CTT CCG TTG 3’ |
|  | Reverse | | 5’CAG CCC ATT CTT CTC TTT GGC CTG TTC 3’ |
| *L. pectinata* 18S rRNA | Forward | | 5’ GGA CGA GCG CTT TTG TTA GTA CA 3’ |
|  | Reverse | | 5’AGT GGT CCA GAG TCA ACA AGC TT 3’ |
| Primers used for the amplification of HbI, HbII and HbIII cDNA by RT-PCR | | | |
| Region | **GSP primer** | **Sequence** | |
| HbII | HbIIUTRFow | 5’ TGA CAA TTT GCA GTA TAG ACC ATT CAG AGG 3' | |
|  | HbII6976R | 5’ CGT CAC CCA TAT CTA CAA AAG CAC GTG AG 3’ | |
| HbIII | HbIIIUGSP1F | 5’ TCG GTT TTG GGG TAC AGA CTT TAT CTG AGG AAC TGC 3’ | |
|  | HbIIITSP4R | 5’ AAA GGC GCT CTT TCA TAA AGT CTC 3’ | |
| HbI Long Variant | HbIUTRF1 | 5’ GGT ATT CAG ATC GTC AGA GTT GCG CTA GTG TTG 3’ | |
|  | HbIOL3R | 5’ CAG CCC ATT CTT CTC TTT GGC CTG TTC 3’ | |
| HbI Short Variant | HbI.V1.F | 5’ GGC TCA TTC TTA AAT CTT CCG TTG 3’ | |
|  | HbIOL3R | 5’ CAG CCC ATT CTT CTC TTT GGC CTG TTC 3’ | |

**Table B.** LAGAND Aligned hits for HbII and HbIII promoter regions.

| Matrix | Identity | HbII Seq from (relative to TSS) | HbII Seq to (relative to TSS) | HbIII Seq from  (relative to TSS) | HbIII Seq to  (relative to TSS) | Strand | Score1 | Score2 | Rel.Score1 | Rel.Score2 | Factor | Found by: |
| --- | --- | --- | --- | --- | --- | --- | --- | --- | --- | --- | --- | --- |
|  |  |  |  |  |  |  |  |  |  |  |  | C = CONREAL |
|  |  |  |  |  |  |  |  |  |  |  |  | L = LAGAN |
|  |  |  |  |  |  |  |  |  |  |  |  | M = MAVID |
|  |  |  |  |  |  |  |  |  |  |  |  | Z = LASTZ |
| M00253 | 50 | -1308 | -1301 | -1742 | -1735 | 1 | 6.008 | 3.61 | 0.94 | 0.84 | cap | L only |
| M00042 | 50 | -1250 | -1241 | -1593 | -1584 | 2 | 7.472 | 6.5 | 0.83 | 0.8 | Sox-5 | L only |
| M00472 | 51.61 | -1250 | -1240 | -1593 | -1583 | 2 | 7.393 | 6.552 | 0.83 | 0.81 | FOXO4 | L only |
| MA0027 | 51.61 | -1250 | -1240 | -1593 | -1583 | 1 | 4.52 | 5.94 | 0.81 | 0.86 | EN-1 | L only |
| MA0063 | 51.85 | -1250 | -1244 | -1593 | -1587 | 2 | 5.939 | 5.939 | 0.86 | 0.86 | Nkx | L only |
| MA0075 | 52 | -1249 | -1245 | -1592 | -1588 | 1 | 4.766 | 4.766 | 0.82 | 0.82 | S8 | L only |
| MA0084 | 51.72 | -1248 | -1240 | -1591 | -1583 | 2 | 8.961 | 8.342 | 0.91 | 0.89 | SRY | L only |
| M00101 | 51.85 | -1247 | -1241 | -1590 | -1584 | 2 | 2.92 | 3.823 | 0.81 | 0.85 | CdxA | L only |
| M00101 | 55.56 | -1246 | -1240 | -1589 | -1583 | 2 | 4.419 | 4.263 | 0.88 | 0.87 | CdxA | L only |
| M00101 | 59.26 | -1244 | -1238 | -1587 | -1581 | 2 | 2.623 | 2.623 | 0.8 | 0.8 | CdxA | L only |
| MA0033 | 57.14 | -1244 | -1237 | -1587 | -1580 | 2 | 7.462 | 6.231 | 0.94 | 0.89 | FREAC-7 | L only |
| M00253 | 67.86 | -1240 | -1233 | -1583 | -1576 | 1 | 2.877 | 5.328 | 0.81 | 0.92 | cap | L only |
| M00100 | 55.56 | -1234 | -1228 | -1577 | -1571 | 2 | 4.507 | 4.495 | 0.83 | 0.83 | CdxA | L only |
| M00486 | 55.17 | -1234 | -1226 | -1577 | -1569 | 1 | 5.311 | 5.501 | 0.86 | 0.87 | Pax-2 | L only |
| M00496 | 57.14 | -1234 | -1227 | -1577 | -1570 | 2 | 4.244 | 5.257 | 0.81 | 0.86 | STAT11 | L only |
| M00498 | 57.14 | -1234 | -1227 | -1577 | -1570 | 2 | 5.394 | 5.797 | 0.87 | 0.89 | STAT4 | L only |
| M00498 | 50 | -1117 | -1110 | -1443 | -1436 | 1 | 4.501 | 4.668 | 0.84 | 0.85 | STAT4 | L only |
| M00500 | 50 | -1117 | -1110 | -1443 | -1436 | 1 | 5.664 | 3.993 | 0.87 | 0.81 | STAT6 | L only |
| M00921 | 50 | -1081 | -1074 | -1383 | -1376 | 1 | 6.303 | 5.512 | 0.85 | 0.82 | GR | L only |
| M00921 | 50 | -1079 | -1072 | -1381 | -1374 | 2 | 6.635 | 6.173 | 0.87 | 0.85 | GR | L only |
| MA0032 | 53.57 | -1077 | -1070 | -1379 | -1372 | 2 | 5.238 | 5.045 | 0.88 | 0.86 | FREAC-3 | L only |
| M00253 | 50 | -1076 | -1069 | -1378 | -1371 | 1 | 2.638 | 4.329 | 0.8 | 0.87 | cap | L only |
| M00724 | 51.61 | -1032 | -1022 | -1273 | -1263 | 1 | 5.776 | 7.261 | 0.81 | 0.84 | HNF3α | L only |
| M00486 | 51.72 | -1025 | -1017 | -1266 | -1258 | 1 | 5.049 | 4.306 | 0.85 | 0.81 | Pax-2 | L only |
| M00253 | 50 | -969 | -962 | -1193 | -1186 | 1 | 4.567 | 4.07 | 0.88 | 0.86 | cap | L only |
| MA0103 | 50 | -968 | -963 | -1192 | -1187 | 1 | 6.503 | 4.633 | 0.89 | 0.82 | deltaEF1 | L only |
| M00486 | 51.72 | -927 | -919 | -1161 | -1153 | 2 | 7.149 | 6.307 | 0.94 | 0.9 | Pax-2 | L only |
| M00100 | 51.85 | -925 | -919 | -1159 | -1153 | 1 | 6.269 | 5.686 | 0.92 | 0.89 | CdxA | L only |
| M00253 | 50 | -894 | -887 | -1125 | -1118 | 2 | 5.67 | 3.333 | 0.93 | 0.83 | cap | L only |
| M00101 | 55.56 | -882 | -876 | -1113 | -1107 | 2 | 3.063 | 5.167 | 0.82 | 0.91 | CdxA | L only |
| M00498 | 50 | -830 | -823 | -1043 | -1036 | 1 | 4.662 | 5.693 | 0.85 | 0.88 | STAT4 | L M |
| MA0075 | 52 | -829 | -825 | -1042 | -1038 | 1 | 5.384 | 9.124 | 0.84 | 1 | S8 | L M |
| M00101 | 51.85 | -828 | -822 | -1041 | -1035 | 1 | 5.49 | 3.475 | 0.92 | 0.84 | CdxA | L M |
| M00396 | 51.85 | -827 | -821 | -1040 | -1034 | 2 | 3.728 | 4.065 | 0.81 | 0.83 | En-1 | L M |
| MA0032 | 50 | -823 | -816 | -1036 | -1029 | 2 | 5.179 | 5.079 | 0.87 | 0.87 | FREAC-3 | L M |
| M00415 | 51.72 | -708 | -700 | -933 | -925 | 1 | 6.164 | 5.705 | 0.84 | 0.83 | AREB6 | L M |
| M00253 | 53.57 | -683 | -676 | -910 | -903 | 2 | 5.052 | 4.467 | 0.9 | 0.88 | cap | L M |
| M00396 | 55.56 | -682 | -676 | -909 | -903 | 1 | 4.748 | 4.132 | 0.87 | 0.83 | En-1 | L M |
| M00496 | 50 | -574 | -567 | -721 | -714 | 1 | 4.472 | 6.17 | 0.82 | 0.9 | STAT1 | L M |
| M00499 | 50 | -574 | -567 | -721 | -714 | 1 | 3.849 | 8.061 | 0.81 | 0.99 | ----- | L M |
| M00101 | 51.85 | -573 | -567 | -720 | -714 | 1 | 4.502 | 3.902 | 0.88 | 0.85 | CdxA | L M |
| M00101 | 51.85 | -564 | -558 | -648 | -642 | 2 | 7.288 | 4.648 | 0.99 | 0.88 | CdxA | L only |
| MA0075 | 52 | -564 | -560 | -648 | -644 | 1 | 4.918 | 4.766 | 0.83 | 0.82 | S8 | L only |
| MA0033 | 53.57 | -519 | -512 | -507 | -500 | 2 | 4.403 | 4.654 | 0.8 | 0.82 | FREAC-7 | L M |
| M00101 | 51.85 | -507 | -501 | -495 | -489 | 2 | 4.886 | 4.423 | 0.89 | 0.88 | CdxA | L M |
| M00805 | 57.69 | -425 | -420 | -412 | -407 | 1 | 5.716 | 5.94 | 0.83 | 0.84 | ----- | L only |
| M00130 | 50 | -335 | -324 | -313 | -302 | 2 | 11.556 | 7.497 | 0.91 | 0.82 | FOXD3 | L only |
| MA0041 | 50 | -335 | -324 | -313 | -302 | 2 | 11.72 | 7.053 | 0.9 | 0.81 | HFH-2 | L only |
| M00100 | 59.26 | -332 | -326 | -310 | -304 | 2 | 5.37 | 7.393 | 0.87 | 0.97 | CdxA | L only |
| M00101 | 59.26 | -332 | -326 | -310 | -304 | 2 | 3.394 | 7.444 | 0.83 | 1 | CdxA | L only |
| M00486 | 55.17 | -332 | -324 | -310 | -302 | 1 | 4.276 | 4.585 | 0.81 | 0.83 | Pax-2 | L only |
| M00101 | 59.26 | -331 | -325 | -309 | -303 | 1 | 3.808 | 3.964 | 0.85 | 0.86 | CdxA | L only |
| M00486 | 51.72 | -329 | -321 | -307 | -299 | 2 | 4.866 | 4.534 | 0.84 | 0.82 | Pax-2 | L only |
| M00100 | 51.85 | -327 | -321 | -305 | -299 | 1 | 5.53 | 5.476 | 0.88 | 0.88 | CdxA | L only |
| M00156 | 66.67 | -316 | -304 | -295 | -283 | 1 | 9.028 | 8.124 | 0.83 | 0.8 | RORalpha1 | L M |
| M00172 | 67.74 | -313 | -303 | -292 | -282 | 2 | 8.552 | 6.76 | 0.87 | 0.81 | AP-1 | L M |
| M00173 | 67.74 | -313 | -303 | -292 | -282 | 2 | 8.02 | 6.67 | 0.85 | 0.81 | AP-1 | L M |
| M00039 | 67.86 | -312 | -305 | -291 | -284 | 2 | 6.605 | 7.363 | 0.81 | 0.84 | CREB | L M |
| M00113 | 71.88 | -312 | -301 | -291 | -280 | 2 | 7.379 | 10.227 | 0.82 | 0.9 | CREB | L M |
| M00917 | 70.97 | -312 | -302 | -291 | -281 | 2 | 7.869 | 10.668 | 0.83 | 0.91 | CREB | L M |
| M00981 | 68.97 | -312 | -304 | -291 | -283 | 2 | 6.072 | 9.632 | 0.8 | 0.91 | CREB | L M |
| MA0018 | 71.88 | -311 | -300 | -290 | -279 | 2 | 7.48 | 11.286 | 0.83 | 0.93 | CREB | L M |
| M00076 | 73.33 | -309 | -300 | -288 | -279 | 2 | 4.731 | 6.236 | 0.82 | 0.88 | GATA-2 | L M |
| M00462 | 73.33 | -309 | -300 | -288 | -279 | 2 | 4.553 | 5.45 | 0.8 | 0.84 | GATA-6 | L M |
| M00801 | 69.23 | -309 | -304 | -288 | -283 | 1 | 6.711 | 9.198 | 0.9 | 1 | CREB | L M |
| M00975 | 72.41 | -309 | -301 | -288 | -280 | 2 | 6.731 | 7.005 | 0.87 | 0.88 | RFX | L M |
| MA0067 | 71.43 | -309 | -302 | -288 | -281 | 1 | 4.491 | 4.182 | 0.82 | 0.81 | Pax-2 | L M |
| M00285 | 72.73 | -308 | -296 | -287 | -275 | 1 | 7.03 | 6.215 | 0.86 | 0.83 | TCF11 | L M |
| MA0089 | 73.08 | -308 | -303 | -287 | -282 | 2 | 8.692 | 8.692 | 0.99 | 0.99 | TCF11-MafG | L M |
| MA0095 | 73.08 | -308 | -303 | -287 | -282 | 1 | 5.315 | 5.315 | 0.84 | 0.84 | Yin-Yang | L M |
| M00253 | 78.57 | -307 | -300 | -286 | -279 | 1 | 3.941 | 3.884 | 0.86 | 0.85 | cap | L M |
| MA0035 | 76.92 | -307 | -302 | -286 | -281 | 2 | 5.41 | 5.41 | 0.91 | 0.91 | GATA-1 | L M |
| MA0037 | 76.92 | -307 | -302 | -286 | -281 | 2 | 3.658 | 3.658 | 0.8 | 0.8 | GATA-3 | L M |
| MA0036 | 76 | -306 | -302 | -285 | -281 | 2 | 5.559 | 5.559 | 0.94 | 0.94 | GATA-2 | L M |
| M00511 | 79.41 | -300 | -287 | -279 | -266 | 2 | 10.642 | 11.038 | 0.87 | 0.88 | ERR | L M |
| M00921 | 75 | -300 | -293 | -279 | -272 | 1 | 8.433 | 7.153 | 0.93 | 0.89 | GR | L M |
| M00963 | 75.86 | -300 | -292 | -279 | -271 | 1 | 9.346 | 6.913 | 0.95 | 0.87 | T3R | L M |
| M00156 | 78.79 | -299 | -287 | -278 | -266 | 2 | 8.373 | 10.799 | 0.81 | 0.87 | RORalpha1 | L M |
| M00960 | 76.67 | -299 | -290 | -278 | -269 | 2 | 7.125 | 7.914 | 0.85 | 0.88 | PR | L M |
| M00727 | 78.57 | -298 | -291 | -277 | -270 | 1 | 9.076 | 9.076 | 0.88 | 0.88 | SF1 | L M |
| MA0067 | 78.57 | -298 | -291 | -277 | -270 | 1 | 4.006 | 4.006 | 0.8 | 0.8 | Pax-2 | L M |
| MA0071 | 80 | -298 | -289 | -277 | -268 | 2 | 7.651 | 8.995 | 0.81 | 0.85 | RORalfa-1 | L M |
| MA0098 | 76.92 | -298 | -293 | -277 | -272 | 1 | 3.674 | 3.674 | 0.81 | 0.81 | c-ETS | L M |
| M00691 | 77.42 | -295 | -285 | -274 | -264 | 1 | 9.738 | 8.264 | 0.85 | 0.81 | ATF1 | L M |
| M00805 | 76.92 | -295 | -290 | -274 | -269 | 2 | 7.016 | 7.016 | 0.88 | 0.88 | ----- | L M |
| MA0087 | 77.78 | -289 | -283 | -268 | -262 | 1 | 5.801 | 6.624 | 0.81 | 0.84 | Sox-5 | L M |
| M00395 | 79.31 | -288 | -280 | -267 | -259 | 2 | 5.414 | 4.547 | 0.9 | 0.85 | HOXA3 | L M |
| M00253 | 78.57 | -287 | -280 | -266 | -259 | 1 | 3.94 | 3.358 | 0.86 | 0.83 | cap | L M |
| M00162 | 70.59 | -286 | -273 | -265 | -252 | 1 | 6.415 | 7.579 | 0.81 | 0.84 | Oct-1 | L M |
| M00468 | 77.78 | -286 | -280 | -265 | -259 | 1 | 7.069 | 4.643 | 0.93 | 0.8 | AP-2rep | L M |
| M00960 | 73.33 | -285 | -276 | -264 | -255 | 1 | 6.948 | 9.465 | 0.85 | 0.93 | PR | L M |
| M00963 | 68.97 | -283 | -275 | -262 | -254 | 2 | 5.956 | 6.998 | 0.84 | 0.87 | T3R | L M |
| M00921 | 67.86 | -282 | -275 | -261 | -254 | 2 | 8.96 | 7.772 | 0.95 | 0.91 | GR | L M |
| MA0098 | 69.23 | -282 | -277 | -261 | -256 | 2 | 3.674 | 3.674 | 0.81 | 0.81 | c-ETS | L M |
| M00272 | 63.33 | -281 | -272 | -260 | -251 | 2 | 7.472 | 11.581 | 0.87 | 0.97 | p53 | L M |
| M00272 | 63.33 | -281 | -272 | -260 | -251 | 1 | 4.844 | 10.21 | 0.8 | 0.94 | p53 | L M |
| M00761 | 63.33 | -281 | -272 | -260 | -251 | 1 | 5.459 | 8.873 | 0.81 | 0.92 | p53 | L M |
| M00704 | 65.38 | -278 | -273 | -257 | -252 | 2 | 5.761 | 5.285 | 0.86 | 0.83 | ----- | L M |
| M00253 | 64.29 | -274 | -267 | -253 | -246 | 1 | 3.125 | 5.449 | 0.82 | 0.92 | cap | L M |
| M01029 | 64.29 | -274 | -267 | -253 | -246 | 2 | 7.841 | 6.181 | 0.86 | 0.8 | TFE | L M |
| M00253 | 57.14 | -214 | -207 | -186 | -179 | 2 | 4.015 | 3.247 | 0.86 | 0.83 | cap | L M |
| M00962 | 58.62 | -212 | -204 | -184 | -176 | 2 | 5.094 | 7.741 | 0.82 | 0.92 | AR | L M |
| M00498 | 50 | -192 | -185 | -167 | -160 | 2 | 3.598 | 8.955 | 0.81 | 1 | STAT-4 | L M |
| M00500 | 50 | -192 | -185 | -167 | -160 | 2 | 4.01 | 9.367 | 0.81 | 1 | STAT-6 | L M |
| MA0098 | 53.85 | -192 | -187 | -167 | -162 | 2 | 3.674 | 7.633 | 0.81 | 0.99 | c-ETS | L M |
| M00076 | 50 | -191 | -182 | -166 | -157 | 2 | 5.406 | 4.987 | 0.85 | 0.83 | GATA-2 | L M |
| MA0035 | 57.69 | -189 | -184 | -164 | -159 | 2 | 4.188 | 4.474 | 0.84 | 0.86 | GATA-1 | L M |
| MA0036 | 60 | -188 | -184 | -163 | -159 | 2 | 5.047 | 4.454 | 0.91 | 0.87 | GATA-2 | L M |
| M00101 | 51.85 | -37 | -31 | -29 | -23 | 1 | 3.058 | 3.058 | 0.82 | 0.82 | CdxA | L M |
| M00789 | 51.85 | -37 | -31 | -29 | -23 | 1 | 5.19 | 5.19 | 0.83 | 0.83 | GATA | L M |
| M00980 | 51.85 | -37 | -31 | -29 | -23 | 2 | 6.186 | 6.186 | 0.83 | 0.83 | TBP | L M |
| M00471 | 53.57 | -36 | -29 | -28 | -21 | 1 | 6.968 | 9.219 | 0.83 | 0.91 | TBP | L M |

**Table C.** TFBSs shared between HbII and HbIII motif 1 region.

|  |  | HbII | | | | | | HbIII | | | | | | |
| --- | --- | --- | --- | --- | --- | --- | --- | --- | --- | --- | --- | --- | --- | --- |
| Model ID | **Model name** | **Score** | **Relative score** | **Start** | **End** | **Strand** | **predicted site sequence** | **Score** | **Relative score** | **Start** | **End** | **Strand** | **predicted site sequence** |  |
| MA0089.1 | NFE2L1::MafG | 4.427 | 0.810077650776285 | -293 | -288 | 1 | CTTGAC | 8.692 | 0.994791423381168 | -286 | -281 | -1 | GATGAC | |
| MA0505.1 | Nr5a2 | 11.046 | 0.853159494922009 | -299 | -285 | -1 | GTTGTCAAGGACAAT | 15.878 | 0.922278917338794 | -278 | -264 | -1 | GATTTCAAGGACATC | |
| MA0077.1 | BRCA1 | 7.844 | 0.975700756185932 | -289 | -283 | 1 | ACAACAG | 10.022 | 0.91470401580296 | -267 | -259 | -1 | CTATTGATT | |
| MA0141.2 | Esrrb | 11.642 | 0.91097999414614 | -297 | -286 | -1 | TTGTCAAGGACA | 11.505 | 0.908672632927791 | -276 | -265 | -1 | ATTTCAAGGACA | |
| MA0084.1 | SRY | 6.125 | 0.814627104534829 | -299 | -291 | -1 | AAGGACAAT | 8.436 | 0.891998249212746 | -269 | -261 | 1 | GAAATCAAT | |
| MA0253.1 | vnd | 6.747 | 0.810335760957948 | -295 | -287 | -1 | TGTCAAGGA | 9.019 | 0.8770293849868 | -274 | -266 | -1 | TTTCAAGGA | |
| MA0592.1 | ESRRA | 9.397 | 0.848583944896876 | -299 | -289 | -1 | TCAAGGACAAT | 10.133 | 0.862790620883114 | -278 | -268 | -1 | TCAAGGACATC | |
| MA0445.1 | D | 7.300 | 0.807411951867238 | -302 | -292 | 1 | CGCATTGTCCT | 9.306 | 0.85751268073961 | -268 | -258 | -1 | CCTATTGATTT | |
|  |  | 8.812 | 0.84517481430743 | -289 | -279 | -1 | CCTACTGTTGT |  |  |  |  |  |  |  |
| MA0071.1 | RORA_1 | 7.651 | 0.814636543523554 | -297 | -288 | -1 | GTCAAGGACA | 8.995 | 0.85174003851613 | -276 | -267 | -1 | TTCAAGGACA | |
| MA0087.1 | Sox5 | 5.801 | 0.810059756128162 | -288 | -282 | -1 | ACTGTTG | 6.624 | 0.840728791704037 | -267 | -261 | -1 | ATTGATT | |
|  |  | 6.431 | 0.833536660639463 | -299 | -293 | 1 | ATTGTCC |  |  |  |  |  |  |  |
| MA0515.1 | Sox6 | 13.172 | 0.965565967633313 | -301 | -292 | 1 | GCATTGTCCT | 6.160 | 0.831926893808861 | -268 | -259 | -1 | CTATTGATTT | |
| MA0393.1 | STE12 | 2.279 | 0.82795360541088 | -282 | -276 | 1 | TAGGACA | 2.350 | 0.829169365806106 | -270 | -264 | 1 | TGAAATC | |
|  |  |  |  |  |  |  |  | 2.279 | 0.82795360541088 | -261 | -255 | 1 | TAGGACA | |
| MA0044.1 | HMG-1 | 5.359 | 0.823977100241154 | -285 | -277 | -1 | GTCCTACTG | 5.481 | 0.82814610279022 | -272 | -264 | 1 | CTTGAAATC | |
| MA0514.1 | Sox3 | 9.847 | 0.911384935376409 | -301 | -292 | 1 | GCATTGTCCT | 4.017 | 0.825098860688596 | -268 | -259 | -1 | CTATTGATTT | |
| MA0317.1 | HCM1 | 6.625 | 0.838558996074597 | -290 | -283 | 1 | GACAACAG | 6.178 | 0.824724532701957 | -266 | -259 | 1 | ATCAATAG | |
| MA0044.1 | HMG-1 | 5.359 | 0.823977100241154 | -285 | -277 | -1 | GTCCTACTG | 5.359 | 0.823977100241154 | -264 | -256 | -1 | GTCCTATTG | |
| MA0157.1 | FOXO3 | 6.851 | 0.822263934339279 | -291 | -284 | 1 | TGACAACA | 6.851 | 0.822263934339279 | -270 | -263 | 1 | TGAAATCA | |
| MA0160.1 | NR4A2 | 8.550 | 0.891602334496081 | -298 | -291 | -1 | AAGGACAA | 6.467 | 0.818413218905264 | -277 | -270 | -1 | AAGGACAT | |
| MA0406.1 | TEC1 | 7.774 | 0.870717841483386 | -301 | -294 | 1 | GCATTGTC | 5.549 | 0.808626415815888 | -281 | -274 | -1 | ACATCCCG | |
| MA0307.1 | GLN3 | 3.442 | 0.807760903641786 | -306 | -302 | -1 | GATGA | 3.442 | 0.807760903641786 | -285 | -281 | -1 | GATGA | |
| MA0371.1 | ROX1 | 9.965 | 0.843483185491974 | -290 | -279 | -1 | CCTACTGTTGTC | 10.630 | 0.860375191587535 | -269 | -258 | -1 | CCTATTGATTTC | |
| MA0067.1 | Pax2 | 6.494 | 0.89900813399907 | -294 | -287 | -1 | TGTCAAGG | 4.006 | 0.80130747899199 | -276 | -269 | 1 | TGTCCTTG | |
|  |  | 4.006 | 0.80130747899199 | -297 | -290 | 1 | TGTCCTTG |  |  |  |  |  |  |  |
| MA0480.1 | Foxo1 | 8.835 | 0.876594318132154 | -291 | -281 | -1 | TACTGTTGTCA | 3.896 | 0.801224840557659 | -267 | -257 | -1 | TCCTATTGATT | |

**Table D.** Predicted TFBSs for HbI Short Variant Promoter (>80%).

| AC | ID | Score | Loc | Location relative to TSS | Str | Consensus Sequence | Signal Sequence |
| --- | --- | --- | --- | --- | --- | --- | --- |
| M00176 | V$AP4_Q6 | 0.806815 | 1075 | 16 | (-) | CWCAGCTGGN | TCCCGCTTTT |
| M00050 | V$E2F_02 | 0.956679 | 1073 | 18 | (+) | TTTSGCGC | TTTCCCGC |
| M00238 | V$BARBIE_01 | 0.81334 | 1073 | 18 | (-) | ATNNAAAGCNGRNGG | TTTCCCGCTTTTTAC |
| M00087 | V$IK2_01 | 0.879581 | 1071 | 20 | (-) | NNNYGGGAWNNN | ATTTTCCCGCTT |
| M00180 | V$E2F_Q6 | 0.927628 | 1070 | 21 | (-) | NNGCGCGAAANTK | CATTTTCCCGCTT |
| M00086 | V$IK1_01 | 0.852297 | 1070 | 21 | (-) | NNNTGGGAATRCC | CATTTTCCCGCTT |
| M00074 | V$CETS1P54_02 | 0.83606 | 1070 | 21 | (-) | NNAMMGGAWRWNN | CATTTTCCCGCTT |
| M00253 | V$CAP_01 | 0.922129 | 1069 | 22 | (+) | NCANNNNN | GCATTTTC |
| M00024 | V$E2F_01 | 0.878503 | 1069 | 22 | (-) | TWSGCGCGAAAAYKR | GCATTTTCCCGCTTT |
| M00052 | V$NFKAPPAB65_01 | 0.852455 | 1068 | 23 | (+) | GGGRATTTCC | GGCATTTTCC |
| M00054 | V$NFKAPPAB_01 | 0.851186 | 1068 | 23 | (+) | GGGAMTTYCC | GGCATTTTCC |
| M00147 | V$HSF2_01 | 0.843623 | 1068 | 23 | (-) | NGAANNWTCK | GGCATTTTCC |
| M00053 | V$CREL_01 | 0.840378 | 1068 | 23 | (+) | SGGRNWTTCC | GGCATTTTCC |
| M00147 | V$HSF2_01 | 0.82329 | 1068 | 23 | (+) | NGAANNWTCK | GGCATTTTCC |
| M00146 | V$HSF1_01 | 0.815675 | 1068 | 23 | (-) | RGAANRTTCN | GGCATTTTCC |
| M00127 | V$GATA1_03 | 0.801323 | 1068 | 23 | (-) | RNSNNGATAANNGN | GGCATTTTCCCGCT |
| M00208 | V$NFKB_C | 0.854603 | 1067 | 24 | (+) | NGGGACTTTCCA | AGGCATTTTCCC |
| M00272 | V$P53_02 | 0.822978 | 1067 | 24 | (+) | NGRCWTGYCY | AGGCATTTTC |
| M00272 | V$P53_02 | 0.814478 | 1067 | 24 | (-) | NGRCWTGYCY | AGGCATTTTC |
| M00194 | V$NFKB_Q6 | 0.837479 | 1066 | 25 | (+) | NGGGGAMTTTCCNN | TAGGCATTTTCCCG |
| M00008 | V$SP1_01 | 0.818233 | 1066 | 25 | (+) | GRGGCRGGGW | TAGGCATTTT |
| M00255 | V$GC_01 | 0.835375 | 1064 | 27 | (+) | NRGGGGCGGGGCNK | AATAGGCATTTTCC |
| M00136 | V$OCT1_02 | 0.8281 | 1064 | 27 | (-) | NNGAATATKCANNNN | AATAGGCATTTTCCC |
| M00136 | V$OCT1_02 | 0.81294 | 1061 | 30 | (+) | NNGAATATKCANNNN | ATAAATAGGCATTTT |
| M00131 | V$HNF3B_01 | 0.881097 | 1059 | 32 | (-) | NNNTRTTTRYTY | CAATAAATAGGC |
| M00252 | V$TATA_01 | 0.873129 | 1059 | 32 | (+) | STATAAAWRNNNNNN | CAATAAATAGGCATT |
| M00104 | V$CDPCR1_01 | 0.827743 | 1058 | 33 | (-) | NATCGATCGS | GCAATAAATA |
| M00138 | V$OCT1_04 | 0.820159 | 1058 | 33 | (-) | NNNNNNNWATGCAAATNNNWNNW | GCAATAAATAGGCATTTTCCCGC |
| M00026 | V$RSRFC4_01 | 0.838394 | 1056 | 35 | (-) | RNKCTATTTWTAGMWN | CTGCAATAAATAGGCA |
| M00231 | V$MEF2_02 | 0.818007 | 1052 | 39 | (+) | NNNNNNKCTAWAAATAGMNNNN | ACAACTGCAATAAATAGGCATT |
| M00227 | V$VMYB_02 | 0.907942 | 1051 | 40 | (+) | NSYAACGGN | TACAACTGC |
| M00003 | V$VMYB_01 | 0.869748 | 1051 | 40 | (+) | AAYAACGGNN | TACAACTGCA |
| M00183 | V$MYB_Q6 | 0.827801 | 1051 | 40 | (+) | NNNAACKGNC | TACAACTGCA |
| M00071 | V$E47_02 | 0.800676 | 1048 | 43 | (-) | NNNMRCAGGTGTTMNN | TGTTACAACTGCAATA |
| M00281 | V$RFX1_02 | 0.809339 | 1046 | 45 | (-) | NNGTNRCNATRGYAACNNN | AGTGTTACAACTGCAATAA |
| M00042 | V$SOX5_01 | 0.839199 | 1044 | 47 | (-) | NNAACAATNN | CAAGTGTTAC |
| M00240 | V$NKX25_01 | 0.950176 | 1043 | 48 | (+) | TYAAGTG | GCAAGTG |
| M00217 | V$USF_C | 0.826233 | 1043 | 48 | (-) | NCACGTGN | GCAAGTGT |
| M00184 | V$MYOD_Q6 | 0.845774 | 1042 | 49 | (+) | NNCANCTGNY | TGCAAGTGTT |
| M00277 | V$LMO2COM_01 | 0.846837 | 1041 | 50 | (+) | SNNCAGGTGNNN | GTGCAAGTGTTA |
| M00055 | V$NMYC_01 | 0.80444 | 1041 | 50 | (-) | NNNCACGTGNNN | GTGCAAGTGTTA |
| M00071 | V$E47_02 | 0.821646 | 1039 | 52 | (+) | NNNMRCAGGTGTTMNN | GTGTGCAAGTGTTACA |
| M00158 | V$COUP_01 | 0.819147 | 1039 | 52 | (-) | TGAMCTTTGMMCYT | GTGTGCAAGTGTTA |
| M00162 | V$OCT1_06 | 0.821484 | 1038 | 53 | (-) | CWNAWTKWSATRYN | AGTGTGCAAGTGTT |
| M00109 | V$CEBPB_01 | 0.838534 | 1036 | 55 | (+) | RNRTKNNGMAAKNN | ATAGTGTGCAAGTG |
| M00116 | V$CEBPA_01 | 0.813453 | 1036 | 55 | (-) | NNATTRCNNAANNN | ATAGTGTGCAAGTG |
| M00077 | V$GATA3_01 | 0.916704 | 1033 | 58 | (+) | NNGATARNG | AGGATAGTG |
| M00076 | V$GATA2_01 | 0.958502 | 1032 | 59 | (+) | NNNGATRNNN | TAGGATAGTG |
| M00075 | V$GATA1_01 | 0.873149 | 1032 | 59 | (+) | SNNGATNNNN | TAGGATAGTG |
| M00235 | V$AHRARNT_01 | 0.854307 | 1031 | 60 | (+) | KNNKNNTYGCGTGCMS | ATAGGATAGTGTGCAA |
| M00126 | V$GATA1_02 | 0.822188 | 1030 | 61 | (+) | NNNNNGATANKGNN | GATAGGATAGTGTG |
| M00082 | V$EVI1_05 | 0.9164 | 1029 | 62 | (+) | AGATAAGATAN | TGATAGGATAG |
| M00079 | V$EVI1_02 | 0.854278 | 1029 | 62 | (+) | AGAYAAGATAA | TGATAGGATAG |
| M00203 | V$GATA_C | 0.844051 | 1029 | 62 | (+) | NGATAAGNMNN | TGATAGGATAG |
| M00080 | V$EVI1_03 | 0.8393 | 1029 | 62 | (+) | AGATAAGATAA | TGATAGGATAG |
| M00254 | V$CAAT_01 | 0.835357 | 1029 | 62 | (-) | NNNRRCCAATSA | TGATAGGATAGT |
| M00077 | V$GATA3_01 | 0.924236 | 1028 | 63 | (+) | NNGATARNG | ATGATAGGA |
| M00278 | V$LMO2COM_02 | 0.87938 | 1028 | 63 | (+) | NMGATANSG | ATGATAGGA |
| M00185 | V$NFY_Q6 | 0.806783 | 1028 | 63 | (-) | TRRCCAATSRN | ATGATAGGATA |
| M00075 | V$GATA1_01 | 0.924482 | 1027 | 64 | (+) | SNNGATNNNN | CATGATAGGA |
| M00076 | V$GATA2_01 | 0.905277 | 1027 | 64 | (+) | NNNGATRNNN | CATGATAGGA |
| M00128 | V$GATA1_04 | 0.874081 | 1026 | 65 | (+) | NNCWGATARNNNN | ACATGATAGGATA |
| M00011 | V$EVI1_06 | 0.855027 | 1026 | 65 | (+) | ACAAGATAA | ACATGATAG |
| M00126 | V$GATA1_02 | 0.890313 | 1025 | 66 | (+) | NNNNNGATANKGNN | TACATGATAGGATA |
| M00040 | V$CREBP1_01 | 0.816013 | 1024 | 67 | (+) | TTACGTAA | TTACATGA |
| M00082 | V$EVI1_05 | 0.81254 | 1024 | 67 | (+) | AGATAAGATAN | TTACATGATAG |
| M00079 | V$EVI1_02 | 0.802006 | 1024 | 67 | (+) | AGAYAAGATAA | TTACATGATAG |
| M00260 | V$HLF_01 | 0.933124 | 1023 | 68 | (+) | RTTACRYAAT | GTTACATGAT |
| M00228 | V$VBP_01 | 0.888437 | 1023 | 68 | (+) | GTTACRTMAK | GTTACATGAT |
| M00220 | V$SREBP1_01 | 0.804775 | 1023 | 68 | (-) | NATCACGTGAY | GTTACATGATA |
| M00045 | V$E4BP4_01 | 0.870257 | 1022 | 69 | (-) | NRTTAYGTAAYN | GGTTACATGATA |
| M00117 | V$CEBPB_02 | 0.851013 | 1021 | 70 | (+) | NKNTTGCNYAAYNN | AGGTTACATGATAG |
| M00158 | V$COUP_01 | 0.874033 | 1013 | 78 | (-) | TGAMCTTTGMMCYT | AGGGTAAAAGGTTA |
| M00133 | V$TST1_01 | 0.862822 | 1011 | 80 | (-) | NNKGAWTWANANTNN | CAAGGGTAAAAGGTT |
| M00134 | V$HNF4_01 | 0.862069 | 1011 | 80 | (+) | NNNRGGNCAAAGKTCANNN | CAAGGGTAAAAGGTTACAT |
| M00227 | V$VMYB_02 | 0.805656 | 1009 | 82 | (+) | NSYAACGGN | CACAAGGGT |
| M00123 | V$MYCMAX_02 | 0.87129 | 1006 | 85 | (+) | NANCACGTGNNW | TAGCACAAGGGT |
| M00260 | V$HLF_01 | 0.824239 | 1005 | 86 | (+) | RTTACRYAAT | GTAGCACAAG |
| M00072 | V$CP2_01 | 0.800172 | 1004 | 87 | (+) | GCNMNAMCMAG | AGTAGCACAAG |
| M00240 | V$NKX25_01 | 0.944427 | 1001 | 90 | (+) | TYAAGTG | TCAAGTA |
| M00075 | V$GATA1_01 | 0.825271 | 996 | 95 | (-) | SNNGATNNNN | ATATATCAAG |
| M00162 | V$OCT1_06 | 0.873437 | 995 | 96 | (-) | CWNAWTKWSATRYN | TATATATCAAGTAG |
| M00252 | V$TATA_01 | 0.837605 | 994 | 97 | (+) | STATAAAWRNNNNNN | ATATATATCAAGTAG |
| M00128 | V$GATA1_04 | 0.818934 | 994 | 97 | (-) | NNCWGATARNNNN | ATATATATCAAGT |
| M00203 | V$GATA_C | 0.852128 | 993 | 98 | (-) | NGATAAGNMNN | GATATATATCA |
| M00203 | V$GATA_C | 0.863312 | 992 | 99 | (+) | NGATAAGNMNN | AGATATATATC |
| M00252 | V$TATA_01 | 0.829485 | 992 | 99 | (+) | STATAAAWRNNNNNN | AGATATATATCAAGT |
| M00253 | V$CAP_01 | 0.905372 | 990 | 101 | (+) | NCANNNNN | TCAGATAT |
| M00076 | V$GATA2_01 | 0.862427 | 990 | 101 | (+) | NNNGATRNNN | TCAGATATAT |
| M00075 | V$GATA1_01 | 0.800592 | 990 | 101 | (+) | SNNGATNNNN | TCAGATATAT |
| M00128 | V$GATA1_04 | 0.888174 | 989 | 102 | (+) | NNCWGATARNNNN | GTCAGATATATAT |
| M00271 | V$AML1_01 | 0.853077 | 985 | 106 | (+) | TGTGGT | TTTGGT |
| M00172 | V$AP1FJ_Q2 | 0.840379 | 984 | 107 | (-) | RSTGACTNMNW | ATTTGGTCAGA |
| M00174 | V$AP1_Q6 | 0.831892 | 984 | 107 | (-) | NNTGACTCANN | ATTTGGTCAGA |
| M00188 | V$AP1_Q4 | 0.817322 | 984 | 107 | (-) | RSTGACTMANN | ATTTGGTCAGA |
| M00159 | V$CEBP_01 | 0.933225 | 983 | 108 | (+) | NNTKTGGWNANNN | TATTTGGTCAGAT |
| M00254 | V$CAAT_01 | 0.831404 | 983 | 108 | (-) | NNNRRCCAATSA | TATTTGGTCAGA |
| M00109 | V$CEBPB_01 | 0.863546 | 982 | 109 | (+) | RNRTKNNGMAAKNN | ATATTTGGTCAGAT |
| M00241 | V$NKX25_02 | 0.858671 | 981 | 110 | (+) | CWTAATTG | CATATTTG |
| M00201 | V$CEBP_C | 0.843638 | 981 | 110 | (+) | NGWNTKNKGYAAKNSAYA | CATATTTGGTCAGATATA |
| M00131 | V$HNF3B_01 | 0.882067 | 980 | 111 | (+) | NNNTRTTTRYTY | CCATATTTGGTC |
| M00253 | V$CAP_01 | 0.878758 | 980 | 111 | (+) | NCANNNNN | CCATATTT |
| M00278 | V$LMO2COM_02 | 0.802898 | 980 | 111 | (-) | NMGATANSG | CCATATTTG |
| M00215 | V$SRF_C | 0.936226 | 979 | 112 | (+) | NCCWTATATGGNCWN | TCCATATTTGGTCAG |
| M00186 | V$SRF_Q6 | 0.954776 | 978 | 113 | (-) | GNCCAWATAWGGMN | TTCCATATTTGGTC |
| M00186 | V$SRF_Q6 | 0.856298 | 978 | 113 | (+) | GNCCAWATAWGGMN | TTCCATATTTGGTC |
| M00228 | V$VBP_01 | 0.816518 | 977 | 114 | (+) | GTTACRTMAK | TTTCCATATT |
| M00215 | V$SRF_C | 0.92955 | 976 | 115 | (-) | NCCWTATATGGNCWN | CTTTCCATATTTGGT |
| M00152 | V$SRF_01 | 0.883208 | 976 | 115 | (-) | ATGCCCATATATGGWNNT | CTTTCCATATTTGGTCAG |
| M00152 | V$SRF_01 | 0.808726 | 976 | 115 | (+) | ATGCCCATATATGGWNNT | CTTTCCATATTTGGTCAG |
| M00059 | V$YY1_01 | 0.920052 | 975 | 116 | (+) | NNNNNCCATNTWNNNWN | CCTTTCCATATTTGGTC |
| M00159 | V$CEBP_01 | 0.927715 | 974 | 117 | (-) | NNTKTGGWNANNN | ACCTTTCCATATT |
| M00109 | V$CEBPB_01 | 0.894255 | 974 | 117 | (-) | RNRTKNNGMAAKNN | ACCTTTCCATATTT |
| M00161 | V$OCT1_05 | 0.870995 | 973 | 118 | (+) | MKNATTTGCATAYY | CACCTTTCCATATT |
| M00162 | V$OCT1_06 | 0.868359 | 973 | 118 | (+) | CWNAWTKWSATRYN | CACCTTTCCATATT |
| M00210 | V$OCT_C | 0.823102 | 973 | 118 | (+) | CTNATTTGCATAY | CACCTTTCCATAT |
| M00253 | V$CAP_01 | 0.883687 | 972 | 119 | (+) | NCANNNNN | ACACCTTT |
| M00184 | V$MYOD_Q6 | 0.835595 | 971 | 120 | (+) | NNCANCTGNY | TACACCTTTC |
| M00135 | V$OCT1_01 | 0.818962 | 971 | 120 | (-) | NNNNWTATGCAAATNTNNN | TACACCTTTCCATATTTGG |
| M00201 | V$CEBP_C | 0.801737 | 971 | 120 | (-) | NGWNTKNKGYAAKNSAYA | TACACCTTTCCATATTTG |
| M00073 | V$DELTAEF1_01 | 0.861598 | 970 | 121 | (+) | NNNCACCTNAN | GTACACCTTTC |
| M00277 | V$LMO2COM_01 | 0.81319 | 970 | 121 | (-) | SNNCAGGTGNNN | GTACACCTTTCC |
| M00033 | V$P300_01 | 0.837764 | 967 | 124 | (-) | NNNRGGAGTNNNNS | CACGTACACCTTTC |
| M00255 | V$GC_01 | 0.809652 | 967 | 124 | (-) | NRGGGGCGGGGCNK | CACGTACACCTTTC |
| M00235 | V$AHRARNT_01 | 0.839984 | 964 | 127 | (-) | KNNKNNTYGCGTGCMS | TTGCACGTACACCTTT |
| M00236 | V$ARNT_01 | 0.80046 | 962 | 129 | (+) | NNNNNCACGTGNNNNN | ATTTGCACGTACACCT |
| M00248 | V$OCT1_07 | 0.819165 | 961 | 130 | (-) | TNTATGNTAATT | AATTTGCACGTA |
| M00251 | V$XBP1_01 | 0.815812 | 961 | 130 | (-) | NNGNTGACGTGKNNNWT | AATTTGCACGTACACCT |
| M00195 | V$OCT1_Q6 | 0.856168 | 959 | 132 | (-) | NNNNATGCAAATNAN | ACAATTTGCACGTAC |
| M00136 | V$OCT1_02 | 0.802112 | 958 | 133 | (+) | NNGAATATKCANNNN | GACAATTTGCACGTA |
| M00042 | V$SOX5_01 | 0.852438 | 956 | 135 | (+) | NNAACAATNN | CAGACAATTT |
| M00271 | V$AML1_01 | 0.873587 | 954 | 137 | (-) | TGTGGT | ACCAGA |
| M00079 | V$EVI1_02 | 0.832515 | 952 | 139 | (+) | AGAYAAGATAA | CGACCAGACAA |
| M00254 | V$CAAT_01 | 0.847783 | 950 | 141 | (+) | NNNRRCCAATSA | TCCGACCAGACA |
| M00032 | V$CETS1P54_01 | 0.830527 | 946 | 145 | (-) | NCMGGAWGYN | AACTTCCGAC |
| M00108 | V$NRF2_01 | 0.816134 | 946 | 145 | (-) | ACCGGAAGNS | AACTTCCGAC |
| M00025 | V$ELK1_02 | 0.870217 | 945 | 146 | (-) | NNNNCCGGAARYNN | TAACTTCCGACCAG |
| M00074 | V$CETS1P54_02 | 0.830497 | 945 | 146 | (-) | NNAMMGGAWRWNN | TAACTTCCGACCA |
| M00183 | V$MYB_Q6 | 0.86639 | 943 | 148 | (+) | NNNAACKGNC | TATAACTTCC |
| M00127 | V$GATA1_03 | 0.815532 | 938 | 153 | (+) | RNSNNGATAANNGN | AGGCTTATAACTTC |
| M00101 | V$CDXA_02 | 0.997531 | 930 | 161 | (+) | WWTWMTR | ATTAATG |
| M00100 | V$CDXA_01 | 0.9436 | 930 | 161 | (+) | MTTTATR | ATTAATG |
| M00199 | V$AP1_C | 0.915452 | 927 | 164 | (-) | NTGASTCAG | CTGATTAAT |
| M00188 | V$AP1_Q4 | 0.848734 | 926 | 165 | (+) | RSTGACTMANN | ACTGATTAATG |
| M00173 | V$AP1_Q2 | 0.833382 | 926 | 165 | (+) | RSTGACTNMNW | ACTGATTAATG |
| M00174 | V$AP1_Q6 | 0.828883 | 926 | 165 | (+) | NNTGACTCANN | ACTGATTAATG |
| M00103 | V$CLOX_01 | 0.820748 | 923 | 168 | (+) | NNTATCGATTANYNW | TCTACTGATTAATGT |
| M00160 | V$SRY_02 | 0.804463 | 923 | 168 | (-) | NWWAACAAWANN | TCTACTGATTAA |
| M00099 | V$S8_01 | 0.86397 | 922 | 169 | (+) | WNNANYYAATTANYNN | TTCTACTGATTAATGT |
| M00162 | V$OCT1_06 | 0.860547 | 909 | 182 | (-) | CWNAWTKWSATRYN | GCTATGAAATTTAT |
| M00216 | V$TATA_C | 0.837338 | 909 | 182 | (+) | NCTATAAAAR | GCTATGAAAT |
| M00162 | V$OCT1_06 | 0.898828 | 908 | 183 | (+) | CWNAWTKWSATRYN | CGCTATGAAATTTA |
| M00195 | V$OCT1_Q6 | 0.804039 | 908 | 183 | (+) | NNNNATGCAAATNAN | CGCTATGAAATTTAT |
| M00190 | V$CEBP_Q2 | 0.832168 | 907 | 184 | (-) | NNNTTGCNNAANNN | TCGCTATGAAATTT |
| M00109 | V$CEBPB_01 | 0.811293 | 907 | 184 | (+) | RNRTKNNGMAAKNN | TCGCTATGAAATTT |
| M00104 | V$CDPCR1_01 | 0.852165 | 903 | 188 | (-) | NATCGATCGS | GGGATCGCTA |
| M00106 | V$CDPCR3HD_01 | 0.821223 | 903 | 188 | (-) | NATYGATSSS | GGGATCGCTA |
| M00076 | V$GATA2_01 | 0.917005 | 902 | 189 | (+) | NNNGATRNNN | CGGGATCGCT |
| M00075 | V$GATA1_01 | 0.899803 | 902 | 189 | (+) | SNNGATNNNN | CGGGATCGCT |
| M00076 | V$GATA2_01 | 0.845737 | 902 | 189 | (-) | NNNGATRNNN | CGGGATCGCT |
| M00075 | V$GATA1_01 | 0.835637 | 902 | 189 | (-) | SNNGATNNNN | CGGGATCGCT |
| M00087 | V$IK2_01 | 0.91034 | 899 | 192 | (+) | NNNYGGGAWNNN | CGTCGGGATCGC |
| M00189 | V$AP2_Q6 | 0.847426 | 896 | 195 | (-) | MKCCCSCNGGCG | TTTCGTCGGGAT |
| M00177 | V$CREB_Q2 | 0.806475 | 894 | 197 | (-) | NSTGACGTAANN | GGTTTCGTCGGG |
| M00178 | V$CREB_Q4 | 0.806159 | 894 | 197 | (-) | NSTGACGTMANN | GGTTTCGTCGGG |
| M00209 | V$NFY_C | 0.80363 | 882 | 209 | (-) | NCTGATTGGYTASY | ACTTACGAATAAGG |
| M00199 | V$AP1_C | 0.806414 | 879 | 212 | (-) | NTGASTCAG | TTGACTTAC |
| M00174 | V$AP1_Q6 | 0.810079 | 878 | 213 | (+) | NNTGACTCANN | TTTGACTTACG |
| M00160 | V$SRY_02 | 0.815913 | 875 | 216 | (-) | NWWAACAAWANN | TTTTTTGACTTA |
| M00269 | V$XFD3_01 | 0.884602 | 873 | 218 | (-) | WNWGTMAACAWWMW | ATTTTTTTGACTTA |
| M00162 | V$OCT1_06 | 0.805469 | 873 | 218 | (+) | CWNAWTKWSATRYN | ATTTTTTTGACTTA |
| M00253 | V$CAP_01 | 0.931 | 871 | 220 | (+) | NCANNNNN | GCATTTTT |
| M00042 | V$SOX5_01 | 0.806587 | 871 | 220 | (-) | NNAACAATNN | GCATTTTTTT |
| M00272 | V$P53_02 | 0.806858 | 869 | 222 | (-) | NGRCWTGYCY | AAGCATTTTT |
| M00241 | V$NKX25_02 | 0.839809 | 860 | 231 | (-) | CWTAATTG | TAATCAAG |
| M00160 | V$SRY_02 | 0.838227 | 859 | 232 | (+) | NWWAACAAWANN | ATAATCAAGAAA |
| M00096 | V$PBX1_01 | 0.910564 | 858 | 233 | (+) | ANCAATCAW | TATAATCAA |
| M00162 | V$OCT1_06 | 0.805859 | 858 | 233 | (-) | CWNAWTKWSATRYN | TATAATCAAGAAAG |
| M00129 | V$HFH1_01 | 0.814674 | 857 | 234 | (-) | NAWTGTTTATWT | GTATAATCAAGA |
| M00137 | V$OCT1_03 | 0.915844 | 856 | 235 | (+) | NNNRTAATNANNN | CGTATAATCAAGA |
| M00147 | V$HSF2_01 | 0.846211 | 856 | 235 | (-) | NGAANNWTCK | CGTATAATCA |
| M00268 | V$XFD2_01 | 0.8388 | 856 | 235 | (+) | WNWATAAACAWNNR | CGTATAATCAAGAA |
| M00271 | V$AML1_01 | 0.832985 | 854 | 237 | (-) | TGTGGT | ACCGTA |
| M00199 | V$AP1_C | 0.808455 | 847 | 244 | (-) | NTGASTCAG | GTTAGACAC |
| M00188 | V$AP1_Q4 | 0.818237 | 846 | 245 | (-) | RSTGACTMANN | GGTTAGACACC |
| M00183 | V$MYB_Q6 | 0.840249 | 839 | 252 | (+) | NNNAACKGNC | GGCAACAGGT |
| M00175 | V$AP4_Q5 | 0.81012 | 839 | 252 | (+) | NNCAGCTGNN | GGCAACAGGT |
| M00223 | V$STAT_01 | 0.863121 | 835 | 256 | (-) | TTCCCRKAA | TTATGGCAA |
| M00109 | V$CEBPB_01 | 0.827885 | 835 | 256 | (-) | RNRTKNNGMAAKNN | TTATGGCAACAGGT |
| M00193 | V$NF1_Q6 | 0.812978 | 835 | 256 | (+) | NNTTGGCNNNNNNCCNNN | TTATGGCAACAGGTTAGA |
| M00101 | V$CDXA_02 | 0.992593 | 833 | 258 | (+) | WWTWMTR | AATTATG |
| M00100 | V$CDXA_01 | 0.940167 | 833 | 258 | (+) | MTTTATR | AATTATG |
| M00116 | V$CEBPA_01 | 0.813453 | 833 | 258 | (-) | NNATTRCNNAANNN | AATTATGGCAACAG |
| M00241 | V$NKX25_02 | 0.958615 | 832 | 259 | (-) | CWTAATTG | TAATTATG |
| M00096 | V$PBX1_01 | 0.9178 | 830 | 261 | (+) | ANCAATCAW | ATTAATTAT |
| M00241 | V$NKX25_02 | 0.846002 | 830 | 261 | (+) | CWTAATTG | ATTAATTA |
| M00137 | V$OCT1_03 | 0.892533 | 829 | 262 | (-) | NNNRTAATNANNN | AATTAATTATGGC |
| M00281 | V$RFX1_02 | 0.818162 | 829 | 262 | (+) | NNGTNRCNATRGYAACNNN | AATTAATTATGGCAACAGG |
| M00059 | V$YY1_01 | 0.809633 | 829 | 262 | (-) | NNNNNCCATNTWNNNWN | AATTAATTATGGCAACA |
| M00099 | V$S8_01 | 0.894311 | 828 | 263 | (-) | WNNANYYAATTANYNN | AAATTAATTATGGCAA |
| M00099 | V$S8_01 | 0.875601 | 826 | 265 | (+) | WNNANYYAATTANYNN | ACAAATTAATTATGGC |
| M00145 | V$BRN2_01 | 0.8513 | 826 | 265 | (-) | NNCATNSRWAATNMRN | ACAAATTAATTATGGC |
| M00100 | V$CDXA_01 | 0.93281 | 825 | 266 | (-) | MTTTATR | TACAAAT |
| M00099 | V$S8_01 | 0.825537 | 822 | 269 | (+) | WNNANYYAATTANYNN | ACCTACAAATTAATTA |
| M00039 | V$CREB_01 | 0.825782 | 820 | 271 | (+) | TGACGTMA | TGACCTAC |
| M00156 | V$RORA1_01 | 0.929315 | 819 | 272 | (-) | NWAWNNAGGTCAN | GTGACCTACAAAT |
| M00157 | V$RORA2_01 | 0.884055 | 819 | 272 | (-) | NWAWNTAGGTCAN | GTGACCTACAAAT |
| M00172 | V$AP1FJ_Q2 | 0.906625 | 818 | 273 | (+) | RSTGACTNMNW | GGTGACCTACA |
| M00173 | V$AP1_Q2 | 0.872621 | 818 | 273 | (+) | RSTGACTNMNW | GGTGACCTACA |
| M00188 | V$AP1_Q4 | 0.863983 | 818 | 273 | (+) | RSTGACTMANN | GGTGACCTACA |
| M00174 | V$AP1_Q6 | 0.84355 | 818 | 273 | (+) | NNTGACTCANN | GGTGACCTACA |
| M00113 | V$CREB_02 | 0.825138 | 816 | 275 | (+) | NNGNTGACGYNN | ACGGTGACCTAC |
| M00253 | V$CAP_01 | 0.917201 | 812 | 279 | (+) | NCANNNNN | TCAGACGG |
| M00141 | V$LYF1_01 | 0.86251 | 808 | 283 | (-) | TTTGGGAGR | TCTGTCAGA |
| M00172 | V$AP1FJ_Q2 | 0.818612 | 806 | 285 | (-) | RSTGACTNMNW | TGTCTGTCAGA |
| M00162 | V$OCT1_06 | 0.813672 | 805 | 286 | (+) | CWNAWTKWSATRYN | CTGTCTGTCAGACG |
| M00278 | V$LMO2COM_02 | 0.805593 | 803 | 288 | (-) | NMGATANSG | GGCTGTCTG |
| M00008 | V$SP1_01 | 0.848528 | 801 | 290 | (+) | GRGGCRGGGW | AAGGCTGTCT |
| M00222 | V$TH1E47_01 | 0.80224 | 801 | 290 | (+) | NNNNGNRTCTGGMWTT | AAGGCTGTCTGTCAGA |
| M00203 | V$GATA_C | 0.934452 | 797 | 294 | (+) | NGATAAGNMNN | TGATAAGGCTG |
| M00278 | V$LMO2COM_02 | 0.935984 | 796 | 295 | (+) | NMGATANSG | ATGATAAGG |
| M00077 | V$GATA3_01 | 0.930882 | 796 | 295 | (+) | NNGATARNG | ATGATAAGG |
| M00076 | V$GATA2_01 | 0.887686 | 795 | 296 | (+) | NNNGATRNNN | TATGATAAGG |
| M00075 | V$GATA1_01 | 0.845508 | 795 | 296 | (+) | SNNGATNNNN | TATGATAAGG |
| M00128 | V$GATA1_04 | 0.947304 | 794 | 297 | (+) | NNCWGATARNNNN | GTATGATAAGGCT |
| M00210 | V$OCT_C | 0.834006 | 794 | 297 | (-) | CTNATTTGCATAY | GTATGATAAGGCT |
| M00161 | V$OCT1_05 | 0.896397 | 793 | 298 | (-) | MKNATTTGCATAYY | GGTATGATAAGGCT |
| M00159 | V$CEBP_01 | 0.875851 | 793 | 298 | (+) | NNTKTGGWNANNN | GGTATGATAAGGC |
| M00126 | V$GATA1_02 | 0.859688 | 793 | 298 | (+) | NNNNNGATANKGNN | GGTATGATAAGGCT |
| M00127 | V$GATA1_03 | 0.805488 | 793 | 298 | (+) | RNSNNGATAANNGN | GGTATGATAAGGCT |
| M00162 | V$OCT1_06 | 0.800781 | 793 | 298 | (-) | CWNAWTKWSATRYN | GGTATGATAAGGCT |
| M00082 | V$EVI1_05 | 0.869269 | 792 | 299 | (+) | AGATAAGATAN | AGGTATGATAA |
| M00080 | V$EVI1_03 | 0.809877 | 792 | 299 | (+) | AGATAAGATAA | AGGTATGATAA |
| M00076 | V$GATA2_01 | 0.805593 | 792 | 299 | (-) | NNNGATRNNN | AGGTATGATA |
| M00079 | V$EVI1_02 | 0.802859 | 792 | 299 | (+) | AGAYAAGATAA | AGGTATGATAA |
| M00073 | V$DELTAEF1_01 | 0.909032 | 789 | 302 | (-) | NNNCACCTNAN | TTCAGGTATGA |
| M00162 | V$OCT1_06 | 0.847266 | 783 | 308 | (+) | CWNAWTKWSATRYN | GGTATTTTCAGGTA |
| M00147 | V$HSF2_01 | 0.832532 | 783 | 308 | (-) | NGAANNWTCK | GGTATTTTCA |
| M00053 | V$CREL_01 | 0.820912 | 783 | 308 | (+) | SGGRNWTTCC | GGTATTTTCA |
| M00053 | V$CREL_01 | 0.820634 | 783 | 308 | (-) | SGGRNWTTCC | GGTATTTTCA |
| M00146 | V$HSF1_01 | 0.805041 | 783 | 308 | (+) | RGAANRTTCN | GGTATTTTCA |
| M00073 | V$DELTAEF1_01 | 0.861274 | 779 | 312 | (-) | NNNCACCTNAN | GTAAGGTATTT |
| M00248 | V$OCT1_07 | 0.800085 | 778 | 313 | (+) | TNTATGNTAATT | TGTAAGGTATTT |
| M00100 | V$CDXA_01 | 0.93281 | 775 | 316 | (+) | MTTTATR | ATTTGTA |
| M00199 | V$AP1_C | 0.831778 | 775 | 316 | (+) | NTGASTCAG | ATTTGTAAG |
| M00162 | V$OCT1_06 | 0.821094 | 774 | 317 | (-) | CWNAWTKWSATRYN | AATTTGTAAGGTAT |
| M00160 | V$SRY_02 | 0.814151 | 773 | 318 | (-) | NWWAACAAWANN | TAATTTGTAAGG |
| M00109 | V$CEBPB_01 | 0.885587 | 772 | 319 | (+) | RNRTKNNGMAAKNN | ATAATTTGTAAGGT |
| M00101 | V$CDXA_02 | 0.995062 | 771 | 320 | (-) | WWTWMTR | TATAATT |
| M00100 | V$CDXA_01 | 0.92104 | 771 | 320 | (-) | MTTTATR | TATAATT |
| M00201 | V$CEBP_C | 0.836655 | 771 | 320 | (+) | NGWNTKNKGYAAKNSAYA | TATAATTTGTAAGGTATT |
| M00252 | V$TATA_01 | 0.814768 | 770 | 321 | (+) | STATAAAWRNNNNNN | GTATAATTTGTAAGG |
| M00137 | V$OCT1_03 | 0.856183 | 769 | 322 | (+) | NNNRTAATNANNN | TGTATAATTTGTA |
| M00260 | V$HLF_01 | 0.917431 | 767 | 324 | (+) | RTTACRYAAT | GTTGTATAAT |
| M00228 | V$VBP_01 | 0.907243 | 767 | 324 | (+) | GTTACRTMAK | GTTGTATAAT |
| M00260 | V$HLF_01 | 0.896185 | 767 | 324 | (-) | RTTACRYAAT | GTTGTATAAT |
| M00045 | V$E4BP4_01 | 0.808766 | 766 | 325 | (-) | NRTTAYGTAAYN | GGTTGTATAATT |
| M00190 | V$CEBP_Q2 | 0.894001 | 765 | 326 | (+) | NNNTTGCNNAANNN | AGGTTGTATAATTT |
| M00116 | V$CEBPA_01 | 0.861557 | 765 | 326 | (-) | NNATTRCNNAANNN | AGGTTGTATAATTT |
| M00117 | V$CEBPB_02 | 0.845749 | 765 | 326 | (+) | NKNTTGCNYAAYNN | AGGTTGTATAATTT |
| M00109 | V$CEBPB_01 | 0.842992 | 765 | 326 | (+) | RNRTKNNGMAAKNN | AGGTTGTATAATTT |
| M00116 | V$CEBPA_01 | 0.83027 | 765 | 326 | (+) | NNATTRCNNAANNN | AGGTTGTATAATTT |
| M00109 | V$CEBPB_01 | 0.81575 | 765 | 326 | (-) | RNRTKNNGMAAKNN | AGGTTGTATAATTT |
| M00201 | V$CEBP_C | 0.822177 | 762 | 329 | (-) | NGWNTKNKGYAAKNSAYA | TGTAGGTTGTATAATTTG |
| M00040 | V$CREBP1_01 | 0.811078 | 752 | 339 | (-) | TTACGTAA | TTATGTAG |
| M00269 | V$XFD3_01 | 0.864609 | 751 | 340 | (-) | WNWGTMAACAWWMW | TTTATGTAGACTGT |
| M00100 | V$CDXA_01 | 1 | 750 | 341 | (+) | MTTTATR | ATTTATG |
| M00101 | V$CDXA_02 | 0.997531 | 750 | 341 | (+) | WWTWMTR | ATTTATG |
| M00096 | V$PBX1_01 | 0.895514 | 747 | 344 | (-) | ANCAATCAW | TTAATTTAT |
| M00267 | V$XFD1_01 | 0.865993 | 745 | 346 | (-) | YAWGTAAAYAWWRY | ATTTAATTTATGTA |
| M00162 | V$OCT1_06 | 0.855469 | 742 | 349 | (-) | CWNAWTKWSATRYN | GGGATTTAATTTAT |
| M00249 | V$CHOP_01 | 0.832969 | 741 | 350 | (-) | NNRTGCAATMCCC | AGGGATTTAATTT |
| M00162 | V$OCT1_06 | 0.808594 | 741 | 350 | (+) | CWNAWTKWSATRYN | AGGGATTTAATTTA |
| M00033 | V$P300_01 | 0.827539 | 739 | 352 | (+) | NNNRGGAGTNNNNS | TCAGGGATTTAATT |
| M00087 | V$IK2_01 | 0.881545 | 738 | 353 | (+) | NNNYGGGAWNNN | GTCAGGGATTTA |
| M00141 | V$LYF1_01 | 0.823154 | 735 | 356 | (+) | TTTGGGAGR | TTTGTCAGG |
| M00199 | V$AP1_C | 0.851603 | 734 | 357 | (+) | NTGASTCAG | TTTTGTCAG |
| M00172 | V$AP1FJ_Q2 | 0.867192 | 733 | 358 | (-) | RSTGACTNMNW | TTTTTGTCAGG |
| M00173 | V$AP1_Q2 | 0.851245 | 733 | 358 | (-) | RSTGACTNMNW | TTTTTGTCAGG |
| M00174 | V$AP1_Q6 | 0.821737 | 733 | 358 | (-) | NNTGACTCANN | TTTTTGTCAGG |
| M00188 | V$AP1_Q4 | 0.820372 | 733 | 358 | (-) | RSTGACTMANN | TTTTTGTCAGG |
| M00177 | V$CREB_Q2 | 0.820488 | 732 | 359 | (-) | NSTGACGTAANN | ATTTTTGTCAGG |
| M00160 | V$SRY_02 | 0.811803 | 732 | 359 | (-) | NWWAACAAWANN | ATTTTTGTCAGG |
| M00253 | V$CAP_01 | 0.967965 | 730 | 361 | (+) | NCANNNNN | TCATTTTT |
| M00148 | V$SRY_01 | 0.923256 | 728 | 363 | (-) | AAACWAM | TTTCATT |
| M00062 | V$IRF1_01 | 0.859108 | 726 | 365 | (-) | SAAAAGYGAAACC | TTTTTCATTTTTG |
| M00162 | V$OCT1_06 | 0.855078 | 724 | 367 | (-) | CWNAWTKWSATRYN | TGTTTTTCATTTTT |
| M00162 | V$OCT1_06 | 0.834375 | 723 | 368 | (+) | CWNAWTKWSATRYN | TTGTTTTTCATTTT |
| M00195 | V$OCT1_Q6 | 0.813046 | 723 | 368 | (-) | NNNNATGCAAATNAN | TTGTTTTTCATTTTT |
| M00148 | V$SRY_01 | 1 | 722 | 369 | (-) | AAACWAM | TTTGTTT |
| M00042 | V$SOX5_01 | 0.805295 | 720 | 371 | (-) | NNAACAATNN | GCTTTGTTTT |
| M00160 | V$SRY_02 | 0.891075 | 719 | 372 | (-) | NWWAACAAWANN | AGCTTTGTTTTT |
| M00138 | V$OCT1_04 | 0.830406 | 719 | 372 | (-) | NNNNNNNWATGCAAATNNNWNNW | AGCTTTGTTTTTCATTTTTGTCA |
| M00062 | V$IRF1_01 | 0.803532 | 719 | 372 | (-) | SAAAAGYGAAACC | AGCTTTGTTTTTC |
| M00272 | V$P53_02 | 0.802169 | 719 | 372 | (+) | NGRCWTGYCY | AGCTTTGTTT |
| M00253 | V$CAP_01 | 0.960079 | 717 | 374 | (+) | NCANNNNN | TCAGCTTT |
| M00175 | V$AP4_Q5 | 0.850653 | 716 | 375 | (+) | NNCAGCTGNN | ATCAGCTTTG |
| M00175 | V$AP4_Q5 | 0.834603 | 716 | 375 | (-) | NNCAGCTGNN | ATCAGCTTTG |
| M00176 | V$AP4_Q6 | 0.819344 | 716 | 375 | (+) | CWCAGCTGGN | ATCAGCTTTG |
| M00238 | V$BARBIE_01 | 0.81334 | 714 | 377 | (-) | ATNNAAAGCNGRNGG | ATATCAGCTTTGTTT |
| M00075 | V$GATA1_01 | 0.917078 | 712 | 379 | (-) | SNNGATNNNN | AAATATCAGC |
| M00076 | V$GATA2_01 | 0.905728 | 712 | 379 | (-) | NNNGATRNNN | AAATATCAGC |
| M00131 | V$HNF3B_01 | 0.856346 | 712 | 379 | (+) | NNNTRTTTRYTY | AAATATCAGCTT |
| M00147 | V$HSF2_01 | 0.85841 | 710 | 381 | (+) | NGAANNWTCK | AGAAATATCA |
| M00128 | V$GATA1_04 | 0.855392 | 710 | 381 | (-) | NNCWGATARNNNN | AGAAATATCAGCT |
| M00147 | V$HSF2_01 | 0.836969 | 710 | 381 | (-) | NGAANNWTCK | AGAAATATCA |
| M00146 | V$HSF1_01 | 0.827491 | 710 | 381 | (+) | RGAANRTTCN | AGAAATATCA |
| M00101 | V$CDXA_02 | 0.981893 | 709 | 382 | (-) | WWTWMTR | TAGAAAT |
| M00100 | V$CDXA_01 | 0.919568 | 709 | 382 | (-) | MTTTATR | TAGAAAT |
| M00133 | V$TST1_01 | 0.874903 | 709 | 382 | (+) | NNKGAWTWANANTNN | TAGAAATATCAGCTT |
| M00252 | V$TATA_01 | 0.801827 | 708 | 383 | (+) | STATAAAWRNNNNNN | GTAGAAATATCAGCT |
| M00088 | V$IK3_01 | 0.826978 | 706 | 385 | (+) | TNYTGGGAATACC | TTGTAGAAATATC |
| M00206 | V$HNF1_C | 0.818182 | 706 | 385 | (-) | NGTTAATKAWTNACCAM | TTGTAGAAATATCAGCT |
| M00109 | V$CEBPB_01 | 0.895988 | 704 | 387 | (+) | RNRTKNNGMAAKNN | GTTTGTAGAAATAT |
| M00116 | V$CEBPA_01 | 0.807978 | 704 | 387 | (-) | NNATTRCNNAANNN | GTTTGTAGAAATAT |
| M00003 | V$VMYB_01 | 0.801551 | 700 | 391 | (-) | AAYAACGGNN | TTACGTTTGT |
| M00241 | V$NKX25_02 | 0.853885 | 697 | 394 | (-) | CWTAATTG | GAATTACG |
| M00162 | V$OCT1_06 | 0.837109 | 695 | 396 | (+) | CWNAWTKWSATRYN | AAGAATTACGTTTG |
| M00100 | V$CDXA_01 | 0.913683 | 691 | 400 | (-) | MTTTATR | TACAAAG |
| M00129 | V$HFH1_01 | 0.821632 | 690 | 401 | (-) | NAWTGTTTATWT | ATACAAAGAATT |
| M00160 | V$SRY_02 | 0.852907 | 688 | 403 | (+) | NWWAACAAWANN | GGATACAAAGAA |
| M00076 | V$GATA2_01 | 0.859269 | 686 | 405 | (+) | NNNGATRNNN | CAGGATACAA |
| M00195 | V$OCT1_Q6 | 0.823963 | 686 | 405 | (+) | NNNNATGCAAATNAN | CAGGATACAAAGAAT |
| M00075 | V$GATA1_01 | 0.821816 | 686 | 405 | (+) | SNNGATNNNN | CAGGATACAA |
| M00032 | V$CETS1P54_01 | 0.939978 | 685 | 406 | (+) | NCMGGAWGYN | ACAGGATACA |
| M00011 | V$EVI1_06 | 0.830743 | 685 | 406 | (+) | ACAAGATAA | ACAGGATAC |
| M00127 | V$GATA1_03 | 0.823371 | 684 | 407 | (+) | RNSNNGATAANNGN | GACAGGATACAAAG |
| M00074 | V$CETS1P54_02 | 0.909686 | 683 | 408 | (+) | NNAMMGGAWRWNN | GGACAGGATACAA |
| M00082 | V$EVI1_05 | 0.826829 | 683 | 408 | (+) | AGATAAGATAN | GGACAGGATAC |
| M00081 | V$EVI1_04 | 0.924125 | 678 | 413 | (+) | NGATANGANWAGATA | AATTAGGACAGGATA |
| M00241 | V$NKX25_02 | 0.884009 | 677 | 414 | (-) | CWTAATTG | CAATTAGG |
| M00109 | V$CEBPB_01 | 0.811045 | 677 | 414 | (+) | RNRTKNNGMAAKNN | CAATTAGGACAGGA |
| M00157 | V$RORA2_01 | 0.814043 | 676 | 415 | (+) | NWAWNTAGGTCAN | ACAATTAGGACAG |
| M00253 | V$CAP_01 | 0.920651 | 674 | 417 | (+) | NCANNNNN | TCACAATT |
| M00137 | V$OCT1_03 | 0.86211 | 674 | 417 | (-) | NNNRTAATNANNN | TCACAATTAGGAC |
| M00137 | V$OCT1_03 | 0.899644 | 673 | 418 | (+) | NNNRTAATNANNN | CTCACAATTAGGA |
| M00042 | V$SOX5_01 | 0.802389 | 673 | 418 | (+) | NNAACAATNN | CTCACAATTA |
| M00160 | V$SRY_02 | 0.82707 | 672 | 419 | (+) | NWWAACAAWANN | CCTCACAATTAG |
| M00083 | V$MZF1_01 | 0.86173 | 671 | 420 | (-) | NGNGGGGA | TCCTCACA |
| M00099 | V$S8_01 | 0.815424 | 671 | 420 | (+) | WNNANYYAATTANYNN | TCCTCACAATTAGGAC |
| M00162 | V$OCT1_06 | 0.801562 | 670 | 421 | (-) | CWNAWTKWSATRYN | ATCCTCACAATTAG |
| M00253 | V$CAP_01 | 0.954657 | 668 | 423 | (+) | NCANNNNN | TCATCCTC |
| M00084 | V$MZF1_02 | 0.836291 | 667 | 424 | (-) | KNNNKAGGGGNAA | CTCATCCTCACAA |
| M00076 | V$GATA2_01 | 0.886333 | 666 | 425 | (-) | NNNGATRNNN | ACTCATCCTC |
| M00075 | V$GATA1_01 | 0.865252 | 666 | 425 | (-) | SNNGATNNNN | ACTCATCCTC |
| M00084 | V$MZF1_02 | 0.811547 | 664 | 427 | (-) | KNNNKAGGGGNAA | TAACTCATCCTCA |
| M00199 | V$AP1_C | 0.840816 | 663 | 428 | (-) | NTGASTCAG | ATAACTCAT |
| M00199 | V$AP1_C | 0.835569 | 663 | 428 | (+) | NTGASTCAG | ATAACTCAT |
| M00037 | V$NFE2_01 | 0.833575 | 663 | 428 | (-) | TGCTGASTCAY | ATAACTCATCC |
| M00174 | V$AP1_Q6 | 0.85408 | 662 | 429 | (-) | NNTGACTCANN | GATAACTCATC |
| M00003 | V$VMYB_01 | 0.815772 | 662 | 429 | (+) | AAYAACGGNN | GATAACTCAT |
| M00174 | V$AP1_Q6 | 0.806318 | 662 | 429 | (+) | NNTGACTCANN | GATAACTCATC |
| M00203 | V$GATA_C | 0.896552 | 661 | 430 | (+) | NGATAAGNMNN | TGATAACTCAT |
| M00037 | V$NFE2_01 | 0.848893 | 661 | 430 | (+) | TGCTGASTCAY | TGATAACTCAT |
| M00278 | V$LMO2COM_02 | 0.835243 | 660 | 431 | (+) | NMGATANSG | ATGATAACT |
| M00077 | V$GATA3_01 | 0.827204 | 660 | 431 | (+) | NNGATARNG | ATGATAACT |
| M00076 | V$GATA2_01 | 0.887235 | 659 | 432 | (+) | NNNGATRNNN | TATGATAACT |
| M00075 | V$GATA1_01 | 0.821816 | 659 | 432 | (+) | SNNGATNNNN | TATGATAACT |
| M00223 | V$STAT_01 | 0.888077 | 658 | 433 | (-) | TTCCCRKAA | TTATGATAA |
| M00128 | V$GATA1_04 | 0.882966 | 658 | 433 | (+) | NNCWGATARNNNN | TTATGATAACTCA |
| M00223 | V$STAT_01 | 0.851525 | 658 | 433 | (+) | TTCCCRKAA | TTATGATAA |
| M00269 | V$XFD3_01 | 0.833041 | 657 | 434 | (-) | WNWGTMAACAWWMW | TTTATGATAACTCA |
| M00248 | V$OCT1_07 | 0.818741 | 657 | 434 | (+) | TNTATGNTAATT | TTTATGATAACT |
| M00100 | V$CDXA_01 | 0.929868 | 656 | 435 | (+) | MTTTATR | TTTTATG |
| M00160 | V$SRY_02 | 0.831767 | 656 | 435 | (-) | NWWAACAAWANN | TTTTATGATAAC |
| M00082 | V$EVI1_05 | 0.818298 | 653 | 438 | (-) | AGATAAGATAN | TTATTTTATGA |
| M00206 | V$HNF1_C | 0.819189 | 651 | 440 | (+) | NGTTAATKAWTNACCAM | TGTTATTTTATGATAAC |
| M00130 | V$HFH2_01 | 0.953667 | 648 | 443 | (+) | NAWTGTTTRTTT | GATTGTTATTTT |
| M00231 | V$MEF2_02 | 0.806469 | 648 | 443 | (-) | NNNNNNKCTAWAAATAGMNNNN | GATTGTTATTTTATGATAACTC |
| M00232 | V$MEF2_03 | 0.805807 | 648 | 443 | (-) | NNNNNWKCTAWAAATAGMNNNN | GATTGTTATTTTATGATAACTC |
| M00042 | V$SOX5_01 | 0.927349 | 647 | 444 | (-) | NNAACAATNN | AGATTGTTAT |
| M00160 | V$SRY_02 | 0.866119 | 646 | 445 | (-) | NWWAACAAWANN | AAGATTGTTATT |
| M00077 | V$GATA3_01 | 0.86132 | 646 | 445 | (+) | NNGATARNG | AAGATTGTT |
| M00162 | V$OCT1_06 | 0.847656 | 646 | 445 | (+) | CWNAWTKWSATRYN | AAGATTGTTATTTT |
| M00075 | V$GATA1_01 | 0.903258 | 645 | 446 | (+) | SNNGATNNNN | CAAGATTGTT |
| M00076 | V$GATA2_01 | 0.875507 | 645 | 446 | (+) | NNNGATRNNN | CAAGATTGTT |
| M00096 | V$PBX1_01 | 0.925904 | 639 | 452 | (+) | ANCAATCAW | ACCATTCAA |
| M00162 | V$OCT1_06 | 0.834766 | 639 | 452 | (-) | CWNAWTKWSATRYN | ACCATTCAAGATTG |
| M00147 | V$HSF2_01 | 0.851756 | 637 | 454 | (-) | NGAANNWTCK | GGACCATTCA |
| M00146 | V$HSF1_01 | 0.839307 | 637 | 454 | (-) | RGAANRTTCN | GGACCATTCA |
| M00147 | V$HSF2_01 | 0.812569 | 637 | 454 | (+) | NGAANNWTCK | GGACCATTCA |
| M00136 | V$OCT1_02 | 0.804277 | 636 | 455 | (+) | NNGAATATKCANNNN | AGGACCATTCAAGAT |
| M00254 | V$CAAT_01 | 0.884496 | 635 | 456 | (+) | NNNRRCCAATSA | TAGGACCATTCA |
| M00059 | V$YY1_01 | 0.836173 | 635 | 456 | (+) | NNNNNCCATNTWNNNWN | TAGGACCATTCAAGATT |
| M00241 | V$NKX25_02 | 0.838119 | 630 | 461 | (+) | CWTAATTG | TTTAATAG |
| M00216 | V$TATA_C | 0.805915 | 628 | 463 | (+) | NCTATAAAAR | GGTTTAATAG |
| M00133 | V$TST1_01 | 0.871005 | 626 | 465 | (-) | NNKGAWTWANANTNN | TTGGTTTAATAGGAC |
| M00271 | V$AML1_01 | 0.853077 | 625 | 466 | (+) | TGTGGT | TTTGGT |
| M00159 | V$CEBP_01 | 0.92577 | 623 | 468 | (+) | NNTKTGGWNANNN | TCTTTGGTTTAAT |
| M00254 | V$CAAT_01 | 0.875741 | 623 | 468 | (-) | NNNRRCCAATSA | TCTTTGGTTTAA |
| M00160 | V$SRY_02 | 0.838227 | 623 | 468 | (-) | NWWAACAAWANN | TCTTTGGTTTAA |
| M00129 | V$HFH1_01 | 0.82627 | 620 | 471 | (+) | NAWTGTTTATWT | TATTCTTTGGTT |
| M00042 | V$SOX5_01 | 0.824992 | 619 | 472 | (-) | NNAACAATNN | TTATTCTTTG |
| M00096 | V$PBX1_01 | 0.890304 | 618 | 473 | (-) | ANCAATCAW | ATTATTCTT |
| M00101 | V$CDXA_02 | 0.995062 | 617 | 474 | (-) | WWTWMTR | TATTATT |
| M00042 | V$SOX5_01 | 0.840491 | 616 | 475 | (-) | NNAACAATNN | TTATTATTCT |
| M00137 | V$OCT1_03 | 0.88068 | 613 | 478 | (-) | NNNRTAATNANNN | CTCTTATTATTCT |
| M00127 | V$GATA1_03 | 0.821166 | 612 | 479 | (-) | RNSNNGATAANNGN | TCTCTTATTATTCT |
| M00082 | V$EVI1_05 | 0.806782 | 611 | 480 | (-) | AGATAAGATAN | ATCTCTTATTA |
| M00147 | V$HSF2_01 | 0.858041 | 607 | 484 | (+) | NGAANNWTCK | GGAAATCTCT |
| M00147 | V$HSF2_01 | 0.82366 | 607 | 484 | (-) | NGAANNWTCK | GGAAATCTCT |
| M00076 | V$GATA2_01 | 0.820929 | 607 | 484 | (-) | NNNGATRNNN | GGAAATCTCT |
| M00053 | V$CREL_01 | 0.820634 | 607 | 484 | (-) | SGGRNWTTCC | GGAAATCTCT |
| M00146 | V$HSF1_01 | 0.80898 | 607 | 484 | (+) | RGAANRTTCN | GGAAATCTCT |
| M00075 | V$GATA1_01 | 0.801086 | 607 | 484 | (-) | SNNGATNNNN | GGAAATCTCT |
| M00054 | V$NFKAPPAB_01 | 0.885393 | 606 | 485 | (+) | GGGAMTTYCC | GGGAAATCTC |
| M00054 | V$NFKAPPAB_01 | 0.84769 | 606 | 485 | (-) | GGGAMTTYCC | GGGAAATCTC |
| M00052 | V$NFKAPPAB65_01 | 0.81509 | 606 | 485 | (-) | GGGRATTTCC | GGGAAATCTC |
| M00052 | V$NFKAPPAB65_01 | 0.804072 | 606 | 485 | (+) | GGGRATTTCC | GGGAAATCTC |
| M00194 | V$NFKB_Q6 | 0.871891 | 605 | 486 | (+) | NGGGGAMTTTCCNN | GGGGAAATCTCTTA |
| M00208 | V$NFKB_C | 0.837866 | 605 | 486 | (-) | NGGGACTTTCCA | GGGGAAATCTCT |
| M00054 | V$NFKAPPAB_01 | 0.801998 | 605 | 486 | (+) | GGGAMTTYCC | GGGGAAATCT |
| M00194 | V$NFKB_Q6 | 0.834784 | 604 | 487 | (+) | NGGGGAMTTTCCNN | TGGGGAAATCTCTT |
| M00194 | V$NFKB_Q6 | 0.81364 | 604 | 487 | (-) | NGGGGAMTTTCCNN | TGGGGAAATCTCTT |
| M00083 | V$MZF1_01 | 0.927465 | 602 | 489 | (+) | NGNGGGGA | TATGGGGA |
| M00159 | V$CEBP_01 | 0.876823 | 602 | 489 | (+) | NNTKTGGWNANNN | TATGGGGAAATCT |
| M00141 | V$LYF1_01 | 0.831587 | 602 | 489 | (+) | TTTGGGAGR | TATGGGGAA |
| M00086 | V$IK1_01 | 0.806891 | 602 | 489 | (+) | NNNTGGGAATRCC | TATGGGGAAATCT |
| M00109 | V$CEBPB_01 | 0.934126 | 601 | 490 | (+) | RNRTKNNGMAAKNN | TTATGGGGAAATCT |
| M00223 | V$STAT_01 | 0.85934 | 601 | 490 | (-) | TTCCCRKAA | TTATGGGGA |
| M00117 | V$CEBPB_02 | 0.841537 | 601 | 490 | (-) | NKNTTGCNYAAYNN | TTATGGGGAAATCT |
| M00116 | V$CEBPA_01 | 0.825577 | 601 | 490 | (-) | NNATTRCNNAANNN | TTATGGGGAAATCT |
| M00201 | V$CEBP_C | 0.829331 | 600 | 491 | (+) | NGWNTKNKGYAAKNSAYA | CTTATGGGGAAATCTCTT |
| M00084 | V$MZF1_02 | 0.854536 | 598 | 493 | (+) | KNNNKAGGGGNAA | AACTTATGGGGAA |
| M00148 | V$SRY_01 | 0.924031 | 597 | 494 | (+) | AAACWAM | AAACTTA |
| M00223 | V$STAT_01 | 0.814721 | 588 | 503 | (-) | TTCCCRKAA | TGACGGGCA |
| M00141 | V$LYF1_01 | 0.820598 | 586 | 505 | (+) | TTTGGGAGR | TCTGACGGG |
| M00084 | V$MZF1_02 | 0.815296 | 585 | 506 | (+) | KNNNKAGGGGNAA | GTCTGACGGGCAA |
| M00235 | V$AHRARNT_01 | 0.820544 | 576 | 515 | (-) | KNNKNNTYGCGTGCMS | GTGCACACGGTCTGAC |
| M00123 | V$MYCMAX_02 | 0.801418 | 576 | 515 | (+) | NANCACGTGNNW | GTGCACACGGTC |
| M00159 | V$CEBP_01 | 0.874878 | 573 | 518 | (+) | NNTKTGGWNANNN | TATGTGCACACGG |
| M00101 | V$CDXA_02 | 0.992593 | 570 | 521 | (+) | WWTWMTR | AATTATG |
| M00100 | V$CDXA_01 | 0.940167 | 570 | 521 | (+) | MTTTATR | AATTATG |
| M00241 | V$NKX25_02 | 0.872748 | 569 | 522 | (-) | CWTAATTG | AAATTATG |
| M00100 | V$CDXA_01 | 0.948504 | 566 | 525 | (-) | MTTTATR | GATAAAT |
| M00203 | V$GATA_C | 0.910531 | 565 | 526 | (+) | NGATAAGNMNN | AGATAAATTAT |
| M00082 | V$EVI1_05 | 0.872041 | 565 | 526 | (+) | AGATAAGATAN | AGATAAATTAT |
| M00077 | V$GATA3_01 | 0.840939 | 564 | 527 | (+) | NNGATARNG | CAGATAAAT |
| M00106 | V$CDPCR3HD_01 | 0.828431 | 564 | 527 | (-) | NATYGATSSS | CAGATAAATT |
| M00278 | V$LMO2COM_02 | 0.823787 | 564 | 527 | (+) | NMGATANSG | CAGATAAAT |
| M00076 | V$GATA2_01 | 0.847542 | 563 | 528 | (+) | NNNGATRNNN | TCAGATAAAT |
| M00128 | V$GATA1_04 | 0.829044 | 562 | 529 | (+) | NNCWGATARNNNN | CTCAGATAAATTA |
| M00082 | V$EVI1_05 | 0.818298 | 560 | 531 | (+) | AGATAAGATAN | TGCTCAGATAA |
| M00272 | V$P53_02 | 0.967468 | 555 | 536 | (+) | NGRCWTGYCY | AGACATGCTC |
| M00272 | V$P53_02 | 0.939332 | 555 | 536 | (-) | NGRCWTGYCY | AGACATGCTC |
| M00076 | V$GATA2_01 | 0.806044 | 555 | 536 | (-) | NNNGATRNNN | AGACATGCTC |
| M00078 | V$EVI1_01 | 0.804408 | 555 | 536 | (+) | WGAYAAGATAAGATAA | AGACATGCTCAGATAA |
| M00255 | V$GC_01 | 0.802793 | 554 | 537 | (-) | NRGGGGCGGGGCNK | AAGACATGCTCAGA |
| M00148 | V$SRY_01 | 0.924419 | 553 | 538 | (+) | AAACWAM | AAAGACA |
| M00001 | V$MYOD_01 | 0.840354 | 553 | 538 | (-) | SRACAGGTGKYG | AAAGACATGCTC |
| M00162 | V$OCT1_06 | 0.816016 | 551 | 540 | (-) | CWNAWTKWSATRYN | AAAAAGACATGCTC |
| M00162 | V$OCT1_06 | 0.827344 | 550 | 541 | (+) | CWNAWTKWSATRYN | TAAAAAGACATGCT |
| M00203 | V$GATA_C | 0.889096 | 547 | 544 | (+) | NGATAAGNMNN | AGATAAAAAGA |
| M00252 | V$TATA_01 | 0.875666 | 547 | 544 | (+) | STATAAAWRNNNNNN | AGATAAAAAGACATG |
| M00077 | V$GATA3_01 | 0.8786 | 546 | 545 | (+) | NNGATARNG | AAGATAAAA |
| M00278 | V$LMO2COM_02 | 0.821429 | 546 | 545 | (+) | NMGATANSG | AAGATAAAA |
| M00076 | V$GATA2_01 | 0.824087 | 545 | 546 | (+) | NNNGATRNNN | GAAGATAAAA |
| M00128 | V$GATA1_04 | 0.919424 | 544 | 547 | (+) | NNCWGATARNNNN | TGAAGATAAAAAG |
| M00127 | V$GATA1_03 | 0.931161 | 543 | 548 | (+) | RNSNNGATAANNGN | ATGAAGATAAAAAG |
| M00241 | V$NKX25_02 | 0.839809 | 541 | 550 | (-) | CWTAATTG | GAATGAAG |
| M00138 | V$OCT1_04 | 0.818068 | 535 | 556 | (+) | NNNNNNNWATGCAAATNNNWNNW | ACTGCTGAATGAAGATAAAAAGA |
| M00175 | V$AP4_Q5 | 0.84358 | 534 | 557 | (-) | NNCAGCTGNN | AACTGCTGAA |
| M00183 | V$MYB_Q6 | 0.846473 | 531 | 560 | (+) | NNNAACKGNC | TAGAACTGCT |
| M00203 | V$GATA_C | 0.869835 | 528 | 563 | (+) | NGATAAGNMNN | AGATAGAACTG |
| M00147 | V$HSF2_01 | 0.819963 | 528 | 563 | (+) | NGAANNWTCK | AGATAGAACT |
| M00077 | V$GATA3_01 | 0.901196 | 527 | 564 | (+) | NNGATARNG | CAGATAGAA |
| M00278 | V$LMO2COM_02 | 0.857817 | 527 | 564 | (+) | NMGATANSG | CAGATAGAA |
| M00076 | V$GATA2_01 | 0.836716 | 526 | 565 | (+) | NNNGATRNNN | ACAGATAGAA |
| M00075 | V$GATA1_01 | 0.803554 | 526 | 565 | (+) | SNNGATNNNN | ACAGATAGAA |
| M00227 | V$VMYB_02 | 0.802948 | 526 | 565 | (-) | NSYAACGGN | ACAGATAGA |
| M00128 | V$GATA1_04 | 0.926164 | 525 | 566 | (+) | NNCWGATARNNNN | AACAGATAGAACT |
| M00184 | V$MYOD_Q6 | 0.807835 | 525 | 566 | (-) | NNCANCTGNY | AACAGATAGA |
| M00126 | V$GATA1_02 | 0.844375 | 524 | 567 | (+) | NNNNNGATANKGNN | CAACAGATAGAACT |
| M00003 | V$VMYB_01 | 0.834842 | 522 | 569 | (+) | AAYAACGGNN | GACAACAGAT |
| M00227 | V$VMYB_02 | 0.803249 | 522 | 569 | (+) | NSYAACGGN | GACAACAGA |
| M00192 | V$GR_Q6 | 0.847098 | 521 | 570 | (-) | NNNNNNCNNTNTGTNCTNN | TGACAACAGATAGAACTGC |
| M00172 | V$AP1FJ_Q2 | 0.819874 | 519 | 572 | (+) | RSTGACTNMNW | AATGACAACAG |
| M00173 | V$AP1_Q2 | 0.816984 | 519 | 572 | (+) | RSTGACTNMNW | AATGACAACAG |
| M00188 | V$AP1_Q4 | 0.807258 | 519 | 572 | (+) | RSTGACTMANN | AATGACAACAG |
| M00109 | V$CEBPB_01 | 0.838782 | 518 | 573 | (-) | RNRTKNNGMAAKNN | AAATGACAACAGAT |
| M00184 | V$MYOD_Q6 | 0.809994 | 515 | 576 | (-) | NNCANCTGNY | AGCAAATGAC |
| M00123 | V$MYCMAX_02 | 0.83005 | 514 | 577 | (+) | NANCACGTGNNW | AAGCAAATGACA |
| M00185 | V$NFY_Q6 | 0.815262 | 514 | 577 | (+) | TRRCCAATSRN | AAGCAAATGAC |
| M00122 | V$USF_02 | 0.816967 | 513 | 578 | (+) | NNRNCACGTGNYNN | TAAGCAAATGACAA |
| M00122 | V$USF_02 | 0.816967 | 513 | 578 | (-) | NNRNCACGTGNYNN | TAAGCAAATGACAA |
| M00210 | V$OCT_C | 0.851777 | 512 | 579 | (-) | CTNATTTGCATAY | TTAAGCAAATGAC |
| M00141 | V$LYF1_01 | 0.831587 | 512 | 579 | (+) | TTTGGGAGR | TTAAGCAAA |
| M00254 | V$CAAT_01 | 0.828579 | 512 | 579 | (+) | NNNRRCCAATSA | TTAAGCAAATGA |
| M00160 | V$SRY_02 | 0.808867 | 512 | 579 | (+) | NWWAACAAWANN | TTAAGCAAATGA |
| M00161 | V$OCT1_05 | 0.854558 | 511 | 580 | (-) | MKNATTTGCATAYY | CTTAAGCAAATGAC |
| M00159 | V$CEBP_01 | 0.896921 | 510 | 581 | (-) | NNTKTGGWNANNN | CCTTAAGCAAATG |
| M00195 | V$OCT1_Q6 | 0.805131 | 510 | 581 | (+) | NNNNATGCAAATNAN | CCTTAAGCAAATGAC |
| M00109 | V$CEBPB_01 | 0.875433 | 509 | 582 | (+) | RNRTKNNGMAAKNN | ACCTTAAGCAAATG |
| M00190 | V$CEBP_Q2 | 0.867133 | 509 | 582 | (-) | NNNTTGCNNAANNN | ACCTTAAGCAAATG |
| M00116 | V$CEBPA_01 | 0.85569 | 509 | 582 | (-) | NNATTRCNNAANNN | ACCTTAAGCAAATG |
| M00135 | V$OCT1_01 | 0.814765 | 508 | 583 | (+) | NNNNWTATGCAAATNTNNN | CACCTTAAGCAAATGACAA |
| M00073 | V$DELTAEF1_01 | 0.929175 | 505 | 586 | (+) | NNNCACCTNAN | GTGCACCTTAA |
| M00277 | V$LMO2COM_01 | 0.80969 | 505 | 586 | (-) | SNNCAGGTGNNN | GTGCACCTTAAG |
| M00249 | V$CHOP_01 | 0.862144 | 500 | 591 | (-) | NNRTGCAATMCCC | AAGTAGTGCACCT |
| M00033 | V$P300_01 | 0.83606 | 498 | 593 | (+) | NNNRGGAGTNNNNS | CAAAGTAGTGCACC |
| M00100 | V$CDXA_01 | 0.93281 | 496 | 595 | (-) | MTTTATR | CACAAAG |
| M00109 | V$CEBPB_01 | 0.812531 | 490 | 601 | (-) | RNRTKNNGMAAKNN | TGCTGTCACAAAGT |
| M00172 | V$AP1FJ_Q2 | 0.832808 | 489 | 602 | (-) | RSTGACTNMNW | TTGCTGTCACA |
| M00227 | V$VMYB_02 | 0.801143 | 485 | 606 | (-) | NSYAACGGN | ACCTTTGCT |
| M00158 | V$COUP_01 | 0.858377 | 483 | 608 | (+) | TGAMCTTTGMMCYT | TAACCTTTGCTGTC |
| M00223 | V$STAT_01 | 0.861356 | 477 | 614 | (-) | TTCCCRKAA | TTATAATAA |
| M00137 | V$OCT1_03 | 0.877519 | 476 | 615 | (+) | NNNRTAATNANNN | TTTATAATAACCT |
| M00042 | V$SOX5_01 | 0.804004 | 476 | 615 | (+) | NNAACAATNN | TTTATAATAA |
| M00100 | V$CDXA_01 | 0.910741 | 475 | 616 | (+) | MTTTATR | TTTTATA |
| M00260 | V$HLF_01 | 0.88508 | 474 | 617 | (-) | RTTACRYAAT | ATTTTATAAT |
| M00260 | V$HLF_01 | 0.831482 | 474 | 617 | (+) | RTTACRYAAT | ATTTTATAAT |
| M00228 | V$VBP_01 | 0.818806 | 474 | 617 | (+) | GTTACRTMAK | ATTTTATAAT |
| M00228 | V$VBP_01 | 0.814231 | 474 | 617 | (-) | GTTACRTMAK | ATTTTATAAT |
| M00216 | V$TATA_C | 0.801162 | 474 | 617 | (+) | NCTATAAAAR | ATTTTATAAT |
| M00045 | V$E4BP4_01 | 0.809856 | 473 | 618 | (+) | NRTTAYGTAAYN | TATTTTATAATA |
| M00045 | V$E4BP4_01 | 0.809856 | 473 | 618 | (-) | NRTTAYGTAAYN | TATTTTATAATA |
| M00109 | V$CEBPB_01 | 0.8316 | 472 | 619 | (-) | RNRTKNNGMAAKNN | CTATTTTATAATAA |
| M00116 | V$CEBPA_01 | 0.825577 | 472 | 619 | (-) | NNATTRCNNAANNN | CTATTTTATAATAA |
| M00190 | V$CEBP_Q2 | 0.820758 | 472 | 619 | (+) | NNNTTGCNNAANNN | CTATTTTATAATAA |
| M00082 | V$EVI1_05 | 0.815099 | 472 | 619 | (-) | AGATAAGATAN | CTATTTTATAA |
| M00206 | V$HNF1_C | 0.805092 | 472 | 619 | (-) | NGTTAATKAWTNACCAM | CTATTTTATAATAACCT |
| M00079 | V$EVI1_02 | 0.802006 | 472 | 619 | (-) | AGAYAAGATAA | CTATTTTATAA |
| M00160 | V$SRY_02 | 0.813564 | 471 | 620 | (-) | NWWAACAAWANN | TCTATTTTATAA |
| M00180 | V$E2F_Q6 | 0.815442 | 462 | 629 | (-) | NNGCGCGAAANTK | CATTTTCGTTCTA |
| M00253 | V$CAP_01 | 0.901922 | 461 | 630 | (+) | NCANNNNN | CCATTTTC |
| M00024 | V$E2F_01 | 0.82045 | 461 | 630 | (-) | TWSGCGCGAAAAYKR | CCATTTTCGTTCTAT |
| M00181 | V$E2_Q6 | 0.889597 | 458 | 633 | (-) | NNACCRNNANCGGTRN | CCACCATTTTCGTTCT |
| M00253 | V$CAP_01 | 0.873337 | 458 | 633 | (+) | NCANNNNN | CCACCATT |
| M00107 | V$E2_01 | 0.856129 | 458 | 633 | (-) | NNACCRNNANCGGTRN | CCACCATTTTCGTTCT |
| M00107 | V$E2_01 | 0.80736 | 458 | 633 | (+) | NNACCRNNANCGGTRN | CCACCATTTTCGTTCT |
| M00059 | V$YY1_01 | 0.880079 | 456 | 635 | (+) | NNNNNCCATNTWNNNWN | AGCCACCATTTTCGTTC |
| M00069 | V$YY1_02 | 0.838808 | 455 | 636 | (+) | NNNCGGCCATCTTGNCTSNW | AAGCCACCATTTTCGTTCTA |
| M00188 | V$AP1_Q4 | 0.814578 | 452 | 639 | (-) | RSTGACTMANN | GATAAGCCACC |
| M00053 | V$CREL_01 | 0.813404 | 452 | 639 | (-) | SGGRNWTTCC | GATAAGCCAC |
| M00203 | V$GATA_C | 0.94781 | 451 | 640 | (+) | NGATAAGNMNN | TGATAAGCCAC |
| M00272 | V$P53_02 | 0.816237 | 451 | 640 | (+) | NGRCWTGYCY | TGATAAGCCA |
| M00278 | V$LMO2COM_02 | 0.940701 | 450 | 641 | (+) | NMGATANSG | CTGATAAGC |
| M00077 | V$GATA3_01 | 0.858219 | 450 | 641 | (+) | NNGATARNG | CTGATAAGC |
| M00076 | V$GATA2_01 | 0.879116 | 449 | 642 | (+) | NNNGATRNNN | TCTGATAAGC |
| M00075 | V$GATA1_01 | 0.840079 | 449 | 642 | (+) | SNNGATNNNN | TCTGATAAGC |
| M00128 | V$GATA1_04 | 0.933517 | 448 | 643 | (+) | NNCWGATARNNNN | TTCTGATAAGCCA |
| M00223 | V$STAT_01 | 0.895387 | 448 | 643 | (+) | TTCCCRKAA | TTCTGATAA |
| M00223 | V$STAT_01 | 0.895135 | 448 | 643 | (-) | TTCCCRKAA | TTCTGATAA |
| M00126 | V$GATA1_02 | 0.850625 | 447 | 644 | (+) | NNNNNGATANKGNN | TTTCTGATAAGCCA |
| M00100 | V$CDXA_01 | 0.919568 | 446 | 645 | (+) | MTTTATR | CTTTCTG |
| M00032 | V$CETS1P54_01 | 0.830798 | 444 | 647 | (-) | NCMGGAWGYN | CACTTTCTGA |
| M00278 | V$LMO2COM_02 | 0.801887 | 444 | 647 | (-) | NMGATANSG | CACTTTCTG |
| M00253 | V$CAP_01 | 0.953179 | 443 | 648 | (+) | NCANNNNN | TCACTTTC |
| M00033 | V$P300_01 | 0.841854 | 440 | 651 | (-) | NNNRGGAGTNNNNS | GTTTCACTTTCTGA |
| M00258 | V$ISRE_01 | 0.851469 | 438 | 653 | (+) | CAGTTTCWCTTTYCC | GTGTTTCACTTTCTG |
| M00249 | V$CHOP_01 | 0.833455 | 436 | 655 | (-) | NNRTGCAATMCCC | AAGTGTTTCACTT |
| M00042 | V$SOX5_01 | 0.829512 | 435 | 656 | (-) | NNAACAATNN | AAAGTGTTTC |
| M00241 | V$NKX25_02 | 0.831926 | 431 | 660 | (+) | CWTAATTG | CTTAAAAG |
| M00216 | V$TATA_C | 0.873515 | 429 | 662 | (+) | NCTATAAAAR | CTCTTAAAAG |
| M00141 | V$LYF1_01 | 0.90161 | 428 | 663 | (-) | TTTGGGAGR | CCTCTTAAA |
| M00255 | V$GC_01 | 0.814062 | 423 | 668 | (-) | NRGGGGCGGGGCNK | AAGACCCTCTTAAA |
| M00059 | V$YY1_01 | 0.834862 | 422 | 669 | (+) | NNNNNCCATNTWNNNWN | GAAGACCCTCTTAAAAG |
| M00147 | V$HSF2_01 | 0.816266 | 421 | 670 | (-) | NGAANNWTCK | AGAAGACCCT |
| M00001 | V$MYOD_01 | 0.81378 | 417 | 674 | (-) | SRACAGGTGKYG | CGGCAGAAGACC |
| M00039 | V$CREB_01 | 0.831628 | 414 | 677 | (-) | TGACGTMA | ATACGGCA |
| M00042 | V$SOX5_01 | 0.822732 | 408 | 683 | (+) | NNAACAATNN | AAAAAAATAC |
| M00267 | V$XFD1_01 | 0.850505 | 407 | 684 | (+) | YAWGTAAAYAWWRY | AAAAAAAATACGGC |
| M00160 | V$SRY_02 | 0.81973 | 407 | 684 | (+) | NWWAACAAWANN | AAAAAAAATACG |
| M00130 | V$HFH2_01 | 0.90448 | 406 | 685 | (-) | NAWTGTTTRTTT | AAAAAAAAATAC |
| M00130 | V$HFH2_01 | 0.906017 | 405 | 686 | (-) | NAWTGTTTRTTT | AAAAAAAAAATA |
| M00129 | V$HFH1_01 | 0.806662 | 405 | 686 | (-) | NAWTGTTTATWT | AAAAAAAAAATA |
| M00130 | V$HFH2_01 | 0.896574 | 404 | 687 | (-) | NAWTGTTTRTTT | AAAAAAAAAAAT |
| M00160 | V$SRY_02 | 0.810041 | 403 | 688 | (+) | NWWAACAAWANN | AAAAAAAAAAAA |
| M00081 | V$EVI1_04 | 0.910502 | 402 | 689 | (+) | NGATANGANWAGATA | AAAAAAAAAAAAATA |
| M00160 | V$SRY_02 | 0.810041 | 402 | 689 | (+) | NWWAACAAWANN | AAAAAAAAAAAA |
| M00160 | V$SRY_02 | 0.810041 | 401 | 690 | (+) | NWWAACAAWANN | AAAAAAAAAAAA |
| M00081 | V$EVI1_04 | 0.893395 | 400 | 691 | (+) | NGATANGANWAGATA | AAAAAAAAAAAAAAA |
| M00160 | V$SRY_02 | 0.810041 | 400 | 691 | (+) | NWWAACAAWANN | AAAAAAAAAAAA |
| M00081 | V$EVI1_04 | 0.893395 | 399 | 692 | (+) | NGATANGANWAGATA | AAAAAAAAAAAAAAA |
| M00160 | V$SRY_02 | 0.810041 | 399 | 692 | (+) | NWWAACAAWANN | AAAAAAAAAAAA |
| M00081 | V$EVI1_04 | 0.893395 | 398 | 693 | (+) | NGATANGANWAGATA | AAAAAAAAAAAAAAA |
| M00160 | V$SRY_02 | 0.810041 | 398 | 693 | (+) | NWWAACAAWANN | AAAAAAAAAAAA |
| M00081 | V$EVI1_04 | 0.893395 | 397 | 694 | (+) | NGATANGANWAGATA | AAAAAAAAAAAAAAA |
| M00160 | V$SRY_02 | 0.810041 | 397 | 694 | (+) | NWWAACAAWANN | AAAAAAAAAAAA |
| M00081 | V$EVI1_04 | 0.893395 | 396 | 695 | (+) | NGATANGANWAGATA | AAAAAAAAAAAAAAA |
| M00160 | V$SRY_02 | 0.810041 | 396 | 695 | (+) | NWWAACAAWANN | AAAAAAAAAAAA |
| M00160 | V$SRY_02 | 0.810041 | 395 | 696 | (+) | NWWAACAAWANN | CAAAAAAAAAAA |
| M00216 | V$TATA_C | 0.827304 | 393 | 698 | (+) | NCTATAAAAR | GCCAAAAAAA |
| M00254 | V$CAAT_01 | 0.806269 | 389 | 702 | (+) | NNNRRCCAATSA | AGTTGCCAAAAA |
| M00116 | V$CEBPA_01 | 0.898318 | 388 | 703 | (+) | NNATTRCNNAANNN | CAGTTGCCAAAAAA |
| M00117 | V$CEBPB_02 | 0.854435 | 388 | 703 | (-) | NKNTTGCNYAAYNN | CAGTTGCCAAAAAA |
| M00190 | V$CEBP_Q2 | 0.828119 | 388 | 703 | (+) | NNNTTGCNNAANNN | CAGTTGCCAAAAAA |
| M00109 | V$CEBPB_01 | 0.813769 | 388 | 703 | (-) | RNRTKNNGMAAKNN | CAGTTGCCAAAAAA |
| M00227 | V$VMYB_02 | 0.907641 | 387 | 704 | (-) | NSYAACGGN | ACAGTTGCC |
| M00253 | V$CAP_01 | 0.898472 | 387 | 704 | (+) | NCANNNNN | ACAGTTGC |
| M00184 | V$MYOD_Q6 | 0.867366 | 386 | 705 | (+) | NNCANCTGNY | TACAGTTGCC |
| M00183 | V$MYB_Q6 | 0.843568 | 386 | 705 | (-) | NNNAACKGNC | TACAGTTGCC |
| M00003 | V$VMYB_01 | 0.841629 | 386 | 705 | (-) | AAYAACGGNN | TACAGTTGCC |
| M00175 | V$AP4_Q5 | 0.81284 | 386 | 705 | (-) | NNCAGCTGNN | TACAGTTGCC |
| M00277 | V$LMO2COM_01 | 0.829341 | 385 | 706 | (-) | SNNCAGGTGNNN | ATACAGTTGCCA |
| M00222 | V$TH1E47_01 | 0.836073 | 383 | 708 | (-) | NNNNGNRTCTGGMWTT | AAATACAGTTGCCAAA |
| M00193 | V$NF1_Q6 | 0.836547 | 382 | 709 | (-) | NNTTGGCNNNNNNCCNNN | TAAATACAGTTGCCAAAA |
| M00101 | V$CDXA_02 | 1 | 380 | 711 | (-) | WWTWMTR | TATAAAT |
| M00100 | V$CDXA_01 | 0.980873 | 380 | 711 | (-) | MTTTATR | TATAAAT |
| M00252 | V$TATA_01 | 0.932758 | 379 | 712 | (+) | STATAAAWRNNNNNN | GTATAAATACAGTTG |
| M00131 | V$HNF3B_01 | 0.865081 | 379 | 712 | (-) | NNNTRTTTRYTY | GTATAAATACAG |
| M00267 | V$XFD1_01 | 0.86835 | 378 | 713 | (+) | YAWGTAAAYAWWRY | GGTATAAATACAGT |
| M00216 | V$TATA_C | 0.820174 | 378 | 713 | (+) | NCTATAAAAR | GGTATAAATA |
| M00026 | V$RSRFC4_01 | 0.834904 | 376 | 715 | (-) | RNKCTATTTWTAGMWN | AAGGTATAAATACAGT |
| M00073 | V$DELTAEF1_01 | 0.898635 | 374 | 717 | (-) | NNNCACCTNAN | TTAAGGTATAA |
| M00141 | V$LYF1_01 | 0.82162 | 373 | 718 | (+) | TTTGGGAGR | TTTAAGGTA |
| M00100 | V$CDXA_01 | 0.937224 | 372 | 719 | (+) | MTTTATR | CTTTAAG |
| M00190 | V$CEBP_Q2 | 0.839529 | 366 | 725 | (+) | NNNTTGCNNAANNN | AATTTGCTTTAAGG |
| M00248 | V$OCT1_07 | 0.819165 | 366 | 725 | (-) | TNTATGNTAATT | AATTTGCTTTAA |
| M00272 | V$P53_02 | 0.82796 | 365 | 726 | (+) | NGRCWTGYCY | TAATTTGCTT |
| M00210 | V$OCT_C | 0.808158 | 364 | 727 | (+) | CTNATTTGCATAY | TTAATTTGCTTTA |
| M00195 | V$OCT1_Q6 | 0.802948 | 364 | 727 | (-) | NNNNATGCAAATNAN | TTAATTTGCTTTAAG |
| M00131 | V$HNF3B_01 | 0.870905 | 363 | 728 | (+) | NNNTRTTTRYTY | ATTAATTTGCTT |
| M00162 | V$OCT1_06 | 0.839453 | 363 | 728 | (+) | CWNAWTKWSATRYN | ATTAATTTGCTTTA |
| M00101 | V$CDXA_02 | 0.997531 | 362 | 729 | (-) | WWTWMTR | CATTAAT |
| M00100 | V$CDXA_01 | 0.9436 | 362 | 729 | (-) | MTTTATR | CATTAAT |
| M00253 | V$CAP_01 | 0.913751 | 361 | 730 | (+) | NCANNNNN | TCATTAAT |
| M00099 | V$S8_01 | 0.87914 | 361 | 730 | (-) | WNNANYYAATTANYNN | TCATTAATTTGCTTTA |
| M00099 | V$S8_01 | 0.80354 | 359 | 732 | (+) | WNNANYYAATTANYNN | TCTCATTAATTTGCTT |
| M00137 | V$OCT1_03 | 0.865666 | 358 | 733 | (-) | NNNRTAATNANNN | ATCTCATTAATTT |
| M00082 | V$EVI1_05 | 0.818298 | 356 | 735 | (-) | AGATAAGATAN | TTATCTCATTA |
| M00099 | V$S8_01 | 0.879646 | 355 | 736 | (+) | WNNANYYAATTANYNN | TTTATCTCATTAATTT |
| M00162 | V$OCT1_06 | 0.873437 | 355 | 736 | (-) | CWNAWTKWSATRYN | TTTATCTCATTAAT |
| M00077 | V$GATA3_01 | 0.930882 | 354 | 737 | (-) | NNGATARNG | CTTTATCTC |
| M00100 | V$CDXA_01 | 0.929377 | 354 | 737 | (+) | MTTTATR | CTTTATC |
| M00278 | V$LMO2COM_02 | 0.886792 | 354 | 737 | (-) | NMGATANSG | CTTTATCTC |
| M00076 | V$GATA2_01 | 0.860622 | 354 | 737 | (-) | NNNGATRNNN | CTTTATCTCA |
| M00075 | V$GATA1_01 | 0.801579 | 354 | 737 | (-) | SNNGATNNNN | CTTTATCTCA |
| M00128 | V$GATA1_04 | 0.944547 | 352 | 739 | (-) | NNCWGATARNNNN | CTCTTTATCTCAT |
| M00127 | V$GATA1_03 | 0.815287 | 352 | 739 | (-) | RNSNNGATAANNGN | CTCTTTATCTCATT |
| M00203 | V$GATA_C | 0.864244 | 351 | 740 | (-) | NGATAAGNMNN | CCTCTTTATCT |
| M00137 | V$OCT1_03 | 0.896879 | 350 | 741 | (-) | NNNRTAATNANNN | ACCTCTTTATCTC |
| M00057 | V$COMP1_01 | 0.800479 | 349 | 742 | (-) | NNTNWKGATTGRCNRSRANMRRNN | CACCTCTTTATCTCATTAATTTGC |
| M00184 | V$MYOD_Q6 | 0.8248 | 347 | 744 | (+) | NNCANCTGNY | AACACCTCTT |
| M00175 | V$AP4_Q5 | 0.817193 | 347 | 744 | (+) | NNCAGCTGNN | AACACCTCTT |
| M00001 | V$MYOD_01 | 0.895866 | 346 | 745 | (-) | SRACAGGTGKYG | CAACACCTCTTT |
| M00008 | V$SP1_01 | 0.844281 | 345 | 746 | (-) | GRGGCRGGGW | TCAACACCTC |
| M00071 | V$E47_02 | 0.840361 | 344 | 747 | (-) | NNNMRCAGGTGTTMNN | TTCAACACCTCTTTAT |
| M00249 | V$CHOP_01 | 0.842937 | 341 | 750 | (+) | NNRTGCAATMCCC | AGATTCAACACCT |
| M00253 | V$CAP_01 | 0.94135 | 339 | 752 | (+) | NCANNNNN | TCAGATTC |
| M00133 | V$TST1_01 | 0.871005 | 335 | 756 | (-) | NNKGAWTWANANTNN | CAATTCAGATTCAAC |
| M00241 | V$NKX25_02 | 0.869932 | 335 | 756 | (-) | CWTAATTG | CAATTCAG |
| M00162 | V$OCT1_06 | 0.816016 | 333 | 758 | (-) | CWNAWTKWSATRYN | AACAATTCAGATTC |
| M00042 | V$SOX5_01 | 0.971585 | 331 | 760 | (+) | NNAACAATNN | TTAACAATTC |
| M00160 | V$SRY_02 | 0.936289 | 330 | 761 | (+) | NWWAACAAWANN | GTTAACAATTCA |
| M00209 | V$NFY_C | 0.821262 | 330 | 761 | (-) | NCTGATTGGYTASY | GTTAACAATTCAGA |
| M00227 | V$VMYB_02 | 0.84627 | 327 | 764 | (-) | NSYAACGGN | TCGGTTAAC |
| M00223 | V$STAT_01 | 0.80968 | 326 | 765 | (-) | TTCCCRKAA | TTCGGTTAA |
| M00253 | V$CAP_01 | 0.884672 | 320 | 771 | (+) | NCANNNNN | ACAGAATT |
| M00148 | V$SRY_01 | 0.931008 | 318 | 773 | (+) | AAACWAM | AAACAGA |
| M00181 | V$E2_Q6 | 0.824038 | 318 | 773 | (+) | NNACCRNNANCGGTRN | AAACAGAATTCGGTTA |
| M00003 | V$VMYB_01 | 0.807369 | 316 | 775 | (+) | AAYAACGGNN | CAAAACAGAA |
| M00072 | V$CP2_01 | 0.825472 | 313 | 778 | (+) | GCNMNAMCMAG | CGACAAAACAG |
| M00079 | V$EVI1_02 | 0.805633 | 313 | 778 | (+) | AGAYAAGATAA | CGACAAAACAG |
| M00050 | V$E2F_02 | 0.876701 | 312 | 779 | (-) | TTTSGCGC | GCGACAAA |
| M00278 | V$LMO2COM_02 | 0.806604 | 306 | 785 | (+) | NMGATANSG | CTGAAAGCG |
| M00148 | V$SRY_01 | 0.942636 | 299 | 792 | (+) | AAACWAM | AAACTCA |
| M00076 | V$GATA2_01 | 0.819125 | 272 | 819 | (+) | NNNGATRNNN | TACGATGTAC |
| M00253 | V$CAP_01 | 0.917201 | 265 | 826 | (+) | NCANNNNN | TCATATGT |
| M00217 | V$USF_C | 0.850721 | 265 | 826 | (+) | NCACGTGN | TCATATGT |
| M00217 | V$USF_C | 0.83529 | 265 | 826 | (-) | NCACGTGN | TCATATGT |
| M00277 | V$LMO2COM_01 | 0.803499 | 263 | 828 | (+) | SNNCAGGTGNNN | GTTCATATGTAC |
| M00271 | V$AML1_01 | 1 | 259 | 832 | (+) | TGTGGT | TGTGGT |
| M00042 | V$SOX5_01 | 0.942848 | 252 | 839 | (-) | NNAACAATNN | TTATTGTTGT |
| M00160 | V$SRY_02 | 0.843218 | 251 | 840 | (-) | NWWAACAAWANN | GTTATTGTTGTG |
| M00130 | V$HFH2_01 | 0.896794 | 247 | 844 | (+) | NAWTGTTTRTTT | TATTGTTATTGT |
| M00042 | V$SOX5_01 | 0.964482 | 246 | 845 | (-) | NNAACAATNN | TTATTGTTAT |
| M00160 | V$SRY_02 | 0.936583 | 245 | 846 | (-) | NWWAACAAWANN | ATTATTGTTATT |
| M00096 | V$PBX1_01 | 0.931983 | 245 | 846 | (-) | ANCAATCAW | ATTATTGTT |
| M00248 | V$OCT1_07 | 0.815137 | 245 | 846 | (+) | TNTATGNTAATT | ATTATTGTTATT |
| M00101 | V$CDXA_02 | 0.995062 | 244 | 847 | (-) | WWTWMTR | TATTATT |
| M00042 | V$SOX5_01 | 0.859541 | 243 | 848 | (-) | NNAACAATNN | GTATTATTGT |
| M00136 | V$OCT1_02 | 0.826475 | 236 | 855 | (-) | NNGAATATKCANNNN | CTAATGAGTATTATT |
| M00212 | V$POLY_C | 0.800559 | 234 | 857 | (-) | CAATAAAACCYYYYKCTN | AACTAATGAGTATTATTG |
| M00148 | V$SRY_01 | 0.978682 | 233 | 858 | (+) | AAACWAM | AAACTAA |
| M00137 | V$OCT1_03 | 0.866851 | 233 | 858 | (+) | NNNRTAATNANNN | AAACTAATGAGTA |
| M00099 | V$S8_01 | 0.850569 | 233 | 858 | (-) | WNNANYYAATTANYNN | AAACTAATGAGTATTA |
| M00185 | V$NFY_Q6 | 0.823227 | 233 | 858 | (+) | TRRCCAATSRN | AAACTAATGAG |
| M00254 | V$CAAT_01 | 0.816154 | 231 | 860 | (+) | NNNRRCCAATSA | TGAAACTAATGA |
| M00160 | V$SRY_02 | 0.810628 | 231 | 860 | (+) | NWWAACAAWANN | TGAAACTAATGA |
| M00147 | V$HSF2_01 | 0.83512 | 228 | 863 | (+) | NGAANNWTCK | GGATGAAACT |
| M00106 | V$CDPCR3HD_01 | 0.839965 | 227 | 864 | (-) | NATYGATSSS | CGGATGAAAC |
| M00032 | V$CETS1P54_01 | 0.843835 | 225 | 866 | (+) | NCMGGAWGYN | TACGGATGAA |
| M00074 | V$CETS1P54_02 | 0.906086 | 223 | 868 | (+) | NNAMMGGAWRWNN | CCTACGGATGAAA |
| M00227 | V$VMYB_02 | 0.848977 | 222 | 869 | (+) | NSYAACGGN | TCCTACGGA |
| M00086 | V$IK1_01 | 0.821314 | 217 | 874 | (-) | NNNTGGGAATRCC | GCCTTTCCTACGG |
| M00025 | V$ELK1_02 | 0.814203 | 217 | 874 | (-) | NNNNCCGGAARYNN | GCCTTTCCTACGGA |
| M00063 | V$IRF2_01 | 0.828217 | 211 | 880 | (-) | GAAAAGYGAAASY | ACTTTAGCCTTTC |
| M00148 | V$SRY_01 | 0.903101 | 209 | 882 | (-) | AAACWAM | TGACTTT |
| M00041 | V$CREBP1CJUN_01 | 0.873611 | 209 | 882 | (+) | TGACGTYA | TGACTTTA |
| M00158 | V$COUP_01 | 0.817168 | 209 | 882 | (+) | TGAMCTTTGMMCYT | TGACTTTAGCCTTT |
| M00072 | V$CP2_01 | 0.863636 | 208 | 883 | (-) | GCNMNAMCMAG | CTGACTTTAGC |
| M00178 | V$CREB_Q4 | 0.834679 | 207 | 884 | (+) | NSTGACGTMANN | GCTGACTTTAGC |
| M00172 | V$AP1FJ_Q2 | 0.824921 | 207 | 884 | (+) | RSTGACTNMNW | GCTGACTTTAG |
| M00177 | V$CREB_Q2 | 0.824112 | 207 | 884 | (+) | NSTGACGTAANN | GCTGACTTTAGC |
| M00173 | V$AP1_Q2 | 0.821083 | 207 | 884 | (+) | RSTGACTNMNW | GCTGACTTTAG |
| M00174 | V$AP1_Q6 | 0.816848 | 207 | 884 | (+) | NNTGACTCANN | GCTGACTTTAG |
| M00017 | V$ATF_01 | 0.824023 | 206 | 885 | (+) | CNSTGACGTNNNYC | AGCTGACTTTAGCC |
| M00037 | V$NFE2_01 | 0.80563 | 206 | 885 | (+) | TGCTGASTCAY | AGCTGACTTTA |
| M00175 | V$AP4_Q5 | 0.906964 | 203 | 888 | (-) | NNCAGCTGNN | TGGAGCTGAC |
| M00176 | V$AP4_Q6 | 0.895765 | 203 | 888 | (-) | CWCAGCTGGN | TGGAGCTGAC |
| M00203 | V$GATA_C | 0.833799 | 198 | 893 | (+) | NGATAAGNMNN | TGATATGGAGC |
| M00085 | V$ZID_01 | 0.811067 | 198 | 893 | (-) | NGGCTCYATCAYC | TGATATGGAGCTG |
| M00077 | V$GATA3_01 | 0.898981 | 197 | 894 | (+) | NNGATARNG | ATGATATGG |
| M00278 | V$LMO2COM_02 | 0.897574 | 197 | 894 | (+) | NMGATANSG | ATGATATGG |
| M00076 | V$GATA2_01 | 0.902571 | 196 | 895 | (+) | NNNGATRNNN | TATGATATGG |
| M00075 | V$GATA1_01 | 0.863771 | 196 | 895 | (+) | SNNGATNNNN | TATGATATGG |
| M00128 | V$GATA1_04 | 0.897978 | 195 | 896 | (+) | NNCWGATARNNNN | TTATGATATGGAG |
| M00059 | V$YY1_01 | 0.860419 | 194 | 897 | (-) | NNNNNCCATNTWNNNWN | ATTATGATATGGAGCTG |
| M00241 | V$NKX25_02 | 0.920608 | 192 | 899 | (-) | CWTAATTG | TTATTATG |
| M00109 | V$CEBPB_01 | 0.840763 | 192 | 899 | (+) | RNRTKNNGMAAKNN | TTATTATGATATGG |
| M00162 | V$OCT1_06 | 0.871094 | 191 | 900 | (+) | CWNAWTKWSATRYN | CTTATTATGATATG |
| M00210 | V$OCT_C | 0.810985 | 191 | 900 | (+) | CTNATTTGCATAY | CTTATTATGATAT |
| M00137 | V$OCT1_03 | 0.935994 | 189 | 902 | (-) | NNNRTAATNANNN | TTCTTATTATGAT |
| M00127 | V$GATA1_03 | 0.823371 | 188 | 903 | (-) | RNSNNGATAANNGN | CTTCTTATTATGAT |
| M00156 | V$RORA1_01 | 0.850399 | 183 | 908 | (-) | NWAWNNAGGTCAN | AAGACCTTCTTAT |
| M00255 | V$GC_01 | 0.814307 | 183 | 908 | (-) | NRGGGGCGGGGCNK | AAGACCTTCTTATT |
| M00240 | V$NKX25_01 | 0.991057 | 178 | 913 | (-) | TYAAGTG | CACTTAA |
| M00241 | V$NKX25_02 | 0.869932 | 178 | 913 | (-) | CWTAATTG | CACTTAAG |
| M00042 | V$SOX5_01 | 0.855021 | 174 | 917 | (+) | NNAACAATNN | CTAACACTTA |
| M00101 | V$CDXA_02 | 0.995062 | 165 | 926 | (+) | WWTWMTR | AATAATA |
| M00042 | V$SOX5_01 | 0.859541 | 163 | 928 | (+) | NNAACAATNN | ACAATAATAC |
| M00096 | V$PBX1_01 | 0.931983 | 162 | 929 | (+) | ANCAATCAW | AACAATAAT |
| M00042 | V$SOX5_01 | 0.989667 | 160 | 931 | (+) | NNAACAATNN | TTAACAATAA |
| M00160 | V$SRY_02 | 0.946271 | 159 | 932 | (+) | NWWAACAAWANN | TTTAACAATAAT |
| M00145 | V$BRN2_01 | 0.848188 | 159 | 932 | (+) | NNCATNSRWAATNMRN | TTTAACAATAATACAC |
| M00190 | V$CEBP_Q2 | 0.852411 | 156 | 935 | (-) | NNNTTGCNNAANNN | ACTTTTAACAATAA |
| M00116 | V$CEBPA_01 | 0.845131 | 156 | 935 | (-) | NNATTRCNNAANNN | ACTTTTAACAATAA |
| M00109 | V$CEBPB_01 | 0.821694 | 156 | 935 | (-) | RNRTKNNGMAAKNN | ACTTTTAACAATAA |
| M00269 | V$XFD3_01 | 0.800421 | 156 | 935 | (+) | WNWGTMAACAWWMW | ACTTTTAACAATAA |
| M00183 | V$MYB_Q6 | 0.829046 | 152 | 939 | (+) | NNNAACKGNC | ACGAACTTTT |
| M00148 | V$SRY_01 | 0.905039 | 150 | 941 | (+) | AAACWAM | AAACGAA |
| M00216 | V$TATA_C | 0.805651 | 144 | 947 | (+) | NCTATAAAAR | TGTAGAAAAC |
| M00100 | V$CDXA_01 | 0.93281 | 141 | 950 | (+) | MTTTATR | ATTTGTA |
| M00109 | V$CEBPB_01 | 0.862308 | 141 | 950 | (+) | RNRTKNNGMAAKNN | ATTTGTAGAAAACG |
| M00253 | V$CAP_01 | 0.893051 | 139 | 952 | (+) | NCANNNNN | ACATTTGT |
| M00160 | V$SRY_02 | 0.809454 | 139 | 952 | (-) | NWWAACAAWANN | ACATTTGTAGAA |
| M00277 | V$LMO2COM_01 | 0.809152 | 137 | 954 | (-) | SNNCAGGTGNNN | AAACATTTGTAG |
| M00160 | V$SRY_02 | 0.871697 | 135 | 956 | (+) | NWWAACAAWANN | GAAAACATTTGT |
| M00070 | V$TAL1BETAITF2_01 | 0.812602 | 135 | 956 | (-) | NNNAACAGATGKTNNN | GAAAACATTTGTAGAA |
| M00066 | V$TAL1ALPHAE47_01 | 0.806472 | 135 | 956 | (-) | NNNAACAGATGKTNNN | GAAAACATTTGTAGAA |
| M00130 | V$HFH2_01 | 0.927975 | 133 | 958 | (-) | NAWTGTTTRTTT | GAGAAAACATTT |
| M00131 | V$HNF3B_01 | 0.877942 | 133 | 958 | (-) | NNNTRTTTRYTY | GAGAAAACATTT |
| M00269 | V$XFD3_01 | 0.850929 | 132 | 959 | (+) | WNWGTMAACAWWMW | AGAGAAAACATTTG |
| M00141 | V$LYF1_01 | 0.833376 | 126 | 965 | (+) | TTTGGGAGR | TATGAGAGA |
| M00072 | V$CP2_01 | 0.801029 | 122 | 969 | (-) | GCNMNAMCMAG | CTGGTATGAGA |
| M00272 | V$P53_02 | 0.817702 | 119 | 972 | (-) | NGRCWTGYCY | GGGCTGGTAT |
| M00008 | V$SP1_01 | 0.86863 | 118 | 973 | (+) | GRGGCRGGGW | TGGGCTGGTA |
| M00217 | V$USF_C | 0.839651 | 115 | 976 | (-) | NCACGTGN | ACATGGGC |
| M00001 | V$MYOD_01 | 0.856102 | 113 | 978 | (+) | SRACAGGTGKYG | AAACATGGGCTG |
| M00123 | V$MYCMAX_02 | 0.817704 | 113 | 978 | (-) | NANCACGTGNNW | AAACATGGGCTG |
| M00059 | V$YY1_01 | 0.836173 | 109 | 982 | (-) | NNNNNCCATNTWNNNWN | TCTCAAACATGGGCTGG |
| M00186 | V$SRF_Q6 | 0.803466 | 109 | 982 | (+) | GNCCAWATAWGGMN | TCTCAAACATGGGC |
| M00100 | V$CDXA_01 | 0.961746 | 101 | 990 | (+) | MTTTATR | CTTTATA |
| M00241 | V$NKX25_02 | 0.831926 | 101 | 990 | (+) | CWTAATTG | CTTTATAG |
| M00137 | V$OCT1_03 | 0.870802 | 97 | 994 | (-) | NNNRTAATNANNN | ACCTCTTTATAGT |
| M00268 | V$XFD2_01 | 0.8424 | 96 | 995 | (-) | WNWATAAACAWNNR | CACCTCTTTATAGT |
| M00026 | V$RSRFC4_01 | 0.814136 | 96 | 995 | (+) | RNKCTATTTWTAGMWN | CACCTCTTTATAGTCT |
| M00271 | V$AML1_01 | 0.876099 | 94 | 997 | (-) | TGTGGT | ACCACC |
| M00184 | V$MYOD_Q6 | 0.839914 | 94 | 997 | (+) | NNCANCTGNY | ACCACCTCTT |
| M00231 | V$MEF2_02 | 0.802622 | 94 | 997 | (-) | NNNNNNKCTAWAAATAGMNNNN | ACCACCTCTTTATAGTCTCAAA |
| M00001 | V$MYOD_01 | 0.874803 | 93 | 998 | (-) | SRACAGGTGKYG | AACCACCTCTTT |
| M00073 | V$DELTAEF1_01 | 0.814165 | 93 | 998 | (+) | NNNCACCTNAN | AACCACCTCTT |
| M00008 | V$SP1_01 | 0.815119 | 92 | 999 | (-) | GRGGCRGGGW | TAACCACCTC |
| M00122 | V$USF_02 | 0.807334 | 92 | 999 | (+) | NNRNCACGTGNYNN | TAACCACCTCTTTA |
| M00122 | V$USF_02 | 0.807334 | 92 | 999 | (-) | NNRNCACGTGNYNN | TAACCACCTCTTTA |
| M00255 | V$GC_01 | 0.820676 | 90 | 1001 | (-) | NRGGGGCGGGGCNK | AATAACCACCTCTT |
| M00137 | V$OCT1_03 | 0.900435 | 85 | 1006 | (+) | NNNRTAATNANNN | GTCGTAATAACCA |
| M00127 | V$GATA1_03 | 0.813572 | 85 | 1006 | (+) | RNSNNGATAANNGN | GTCGTAATAACCAC |
| M00271 | V$AML1_01 | 0.90833 | 81 | 1010 | (+) | TGTGGT | AGTGGT |
| M00211 | V$PADS_C | 0.84627 | 81 | 1010 | (+) | NGTGGTCTC | AGTGGTCGT |
| M00116 | V$CEBPA_01 | 0.822448 | 81 | 1010 | (-) | NNATTRCNNAANNN | AGTGGTCGTAATAA |
| M00187 | V$USF_Q6 | 0.800951 | 77 | 1014 | (-) | GYCACGTGNC | AAAAAGTGGT |
| M00050 | V$E2F_02 | 0.808109 | 72 | 1019 | (-) | TTTSGCGC | CTGCCAAA |
| M00141 | V$LYF1_01 | 0.840787 | 71 | 1020 | (-) | TTTGGGAGR | ACTGCCAAA |
| M00162 | V$OCT1_06 | 0.813672 | 69 | 1022 | (-) | CWNAWTKWSATRYN | GGACTGCCAAAAAG |
| M00008 | V$SP1_01 | 0.800396 | 69 | 1022 | (-) | GRGGCRGGGW | GGACTGCCAA |
| M00162 | V$OCT1_06 | 0.813672 | 63 | 1028 | (+) | CWNAWTKWSATRYN | CTTTTTGGACTGCC |
| M00193 | V$NF1_Q6 | 0.801041 | 63 | 1028 | (-) | NNTTGGCNNNNNNCCNNN | CTTTTTGGACTGCCAAAA |
| M00253 | V$CAP_01 | 0.904879 | 60 | 1031 | (+) | NCANNNNN | CCACTTTT |
| M00187 | V$USF_Q6 | 0.85267 | 59 | 1032 | (+) | GYCACGTGNC | GCCACTTTTT |
| M00188 | V$AP1_Q4 | 0.806038 | 54 | 1037 | (-) | RSTGACTMANN | ACTAGGCCACT |
| M00148 | V$SRY_01 | 0.912791 | 52 | 1039 | (+) | AAACWAM | AAACTAG |
| M00272 | V$P53_02 | 0.819168 | 52 | 1039 | (-) | NGRCWTGYCY | AAACTAGGCC |
| M00157 | V$RORA2_01 | 0.888911 | 51 | 1040 | (+) | NWAWNTAGGTCAN | AAAACTAGGCCAC |
| M00156 | V$RORA1_01 | 0.846768 | 51 | 1040 | (+) | NWAWNNAGGTCAN | AAAACTAGGCCAC |
| M00026 | V$RSRFC4_01 | 0.84014 | 46 | 1045 | (-) | RNKCTATTTWTAGMWN | TTTCTAAAACTAGGCC |
| M00101 | V$CDXA_02 | 0.981893 | 45 | 1046 | (+) | WWTWMTR | ATTTCTA |
| M00100 | V$CDXA_01 | 0.919568 | 45 | 1046 | (+) | MTTTATR | ATTTCTA |
| M00109 | V$CEBPB_01 | 0.89574 | 43 | 1048 | (-) | RNRTKNNGMAAKNN | CAATTTCTAAAACT |
| M00116 | V$CEBPA_01 | 0.856081 | 43 | 1048 | (+) | NNATTRCNNAANNN | CAATTTCTAAAACT |
| M00232 | V$MEF2_03 | 0.844075 | 42 | 1049 | (+) | NNNNNWKCTAWAAATAGMNNNN | ACAATTTCTAAAACTAGGCCAC |
| M00231 | V$MEF2_02 | 0.813986 | 42 | 1049 | (+) | NNNNNNKCTAWAAATAGMNNNN | ACAATTTCTAAAACTAGGCCAC |
| M00042 | V$SOX5_01 | 0.831127 | 39 | 1052 | (+) | NNAACAATNN | GATACAATTT |
| M00203 | V$GATA_C | 0.831625 | 38 | 1053 | (+) | NGATAAGNMNN | GGATACAATTT |
| M00076 | V$GATA2_01 | 0.850248 | 36 | 1055 | (+) | NNNGATRNNN | TAGGATACAA |
| M00032 | V$CETS1P54_01 | 0.816404 | 35 | 1056 | (+) | NCMGGAWGYN | ATAGGATACA |
| M00074 | V$CETS1P54_02 | 0.852094 | 33 | 1058 | (+) | NNAMMGGAWRWNN | ATATAGGATACAA |
| M00082 | V$EVI1_05 | 0.809768 | 33 | 1058 | (+) | AGATAAGATAN | ATATAGGATAC |
| M00228 | V$VBP_01 | 0.824396 | 30 | 1061 | (+) | GTTACRTMAK | GTTATATAGG |
| M00045 | V$E4BP4_01 | 0.812037 | 29 | 1062 | (+) | NRTTAYGTAAYN | CGTTATATAGGA |
| M00227 | V$VMYB_02 | 0.959988 | 27 | 1064 | (-) | NSYAACGGN | TCCGTTATA |
| M00003 | V$VMYB_01 | 0.952165 | 26 | 1065 | (-) | AAYAACGGNN | TTCCGTTATA |
| M00260 | V$HLF_01 | 0.838242 | 25 | 1066 | (+) | RTTACRYAAT | GTTCCGTTAT |
| M00228 | V$VBP_01 | 0.829225 | 25 | 1066 | (+) | GTTACRTMAK | GTTCCGTTAT |
| M00108 | V$NRF2_01 | 0.824612 | 23 | 1068 | (-) | ACCGGAAGNS | CCGTTCCGTT |
| M00025 | V$ELK1_02 | 0.81359 | 22 | 1069 | (-) | NNNNCCGGAARYNN | CCCGTTCCGTTATA |
| M00003 | V$VMYB_01 | 0.800259 | 21 | 1070 | (-) | AAYAACGGNN | TCCCGTTCCG |
| M00189 | V$AP2_Q6 | 0.80216 | 19 | 1072 | (+) | MKCCCSCNGGCG | CATCCCGTTCCG |
| M00253 | V$CAP_01 | 0.917201 | 18 | 1073 | (+) | NCANNNNN | TCATCCCG |
| M00087 | V$IK2_01 | 0.896924 | 17 | 1074 | (-) | NNNYGGGAWNNN | GTCATCCCGTTC |
| M00032 | V$CETS1P54_01 | 0.816676 | 17 | 1074 | (-) | NCMGGAWGYN | GTCATCCCGT |
| M00076 | V$GATA2_01 | 0.901669 | 16 | 1075 | (-) | NNNGATRNNN | AGTCATCCCG |
| M00075 | V$GATA1_01 | 0.885982 | 16 | 1075 | (-) | SNNGATNNNN | AGTCATCCCG |
| M00074 | V$CETS1P54_02 | 0.845223 | 16 | 1075 | (-) | NNAMMGGAWRWNN | AGTCATCCCGTTC |
| M00148 | V$SRY_01 | 0.903101 | 14 | 1077 | (+) | AAACWAM | AAAGTCA |
| M00113 | V$CREB_02 | 0.881672 | 13 | 1078 | (-) | NNGNTGACGYNN | TAAAGTCATCCC |
| M00041 | V$CREBP1CJUN_01 | 0.873611 | 13 | 1078 | (-) | TGACGTYA | TAAAGTCA |
| M00172 | V$AP1FJ_Q2 | 0.866877 | 12 | 1079 | (-) | RSTGACTNMNW | ATAAAGTCATC |
| M00173 | V$AP1_Q2 | 0.856223 | 12 | 1079 | (-) | RSTGACTNMNW | ATAAAGTCATC |
| M00174 | V$AP1_Q6 | 0.844302 | 12 | 1079 | (-) | NNTGACTCANN | ATAAAGTCATC |
| M00188 | V$AP1_Q4 | 0.817017 | 12 | 1079 | (-) | RSTGACTMANN | ATAAAGTCATC |
| M00100 | V$CDXA_01 | 0.961746 | 11 | 1080 | (-) | MTTTATR | TATAAAG |
| M00252 | V$TATA_01 | 0.858412 | 10 | 1081 | (+) | STATAAAWRNNNNNN | GTATAAAGTCATCCC |
| M00239 | V$T3R_01 | 0.825478 | 10 | 1081 | (+) | SNNTRAGGTCACGSNN | GTATAAAGTCATCCCG |
| M00036 | V$VJUN_01 | 0.80359 | 9 | 1082 | (-) | NYGATGACGTCATNCY | GGTATAAAGTCATCCC |
| M00216 | V$TATA_C | 0.802746 | 9 | 1082 | (+) | NCTATAAAAR | GGTATAAAGT |
| M00227 | V$VMYB_02 | 0.838147 | 3 | 1088 | (+) | NSYAACGGN | TGCAAGGGT |

**Table E.** Predicted TFBSs for HbI Long Variant Promoter (>80%).

| AC | ID | Score | Loc. | Location Relative to TSS | Str | Consensus Sequence | Signal Sequence |
| --- | --- | --- | --- | --- | --- | --- | --- |
| M00211 | V$PADS_C | 0.872984 | 1003 | 13 | (-) | NGTGGTCTC | GAGACCTCG |
| M00278 | V$LMO2COM_02 | 0.874663 | 995 | 21 | (+) | NMGATANSG | TCGATATTG |
| M00077 | V$GATA3_01 | 0.847585 | 995 | 21 | (+) | NNGATARNG | TCGATATTG |
| M00075 | V$GATA1_01 | 0.801086 | 994 | 22 | (+) | SNNGATNNNN | ATCGATATTG |
| M00104 | V$CDPCR1_01 | 0.802345 | 993 | 23 | (+) | NATCGATCGS | TATCGATATT |
| M00126 | V$GATA1_02 | 0.82875 | 992 | 24 | (+) | NNNNNGATANKGNN | TTATCGATATTGAG |
| M00104 | V$CDPCR1_01 | 0.83621 | 991 | 25 | (-) | NATCGATCGS | GTTATCGATA |
| M00278 | V$LMO2COM_02 | 0.854784 | 990 | 26 | (-) | NMGATANSG | AGTTATCGA |
| M00102 | V$CDP_02 | 0.932751 | 988 | 28 | (-) | NWNATCGATTANYNN | AAAGTTATCGATATT |
| M00103 | V$CLOX_01 | 0.913063 | 988 | 28 | (-) | NNTATCGATTANYNW | AAAGTTATCGATATT |
| M00003 | V$VMYB_01 | 0.800582 | 987 | 29 | (-) | AAYAACGGNN | TAAAGTTATC |
| M00100 | V$CDXA_01 | 0.937224 | 985 | 31 | (-) | MTTTATR | CTTAAAG |
| M00160 | V$SRY_02 | 0.80182 | 985 | 31 | (-) | NWWAACAAWANN | CTTAAAGTTATC |
| M00162 | V$OCT1_06 | 0.805859 | 982 | 34 | (-) | CWNAWTKWSATRYN | GGTCTTAAAGTTAT |
| M00187 | V$USF_Q6 | 0.804026 | 976 | 40 | (+) | GYCACGTGNC | GATACGGGTC |
| M00203 | V$GATA_C | 0.865797 | 975 | 41 | (+) | NGATAAGNMNN | GGATACGGGTC |
| M00077 | V$GATA3_01 | 0.83385 | 974 | 42 | (+) | NNGATARNG | TGGATACGG |
| M00278 | V$LMO2COM_02 | 0.822776 | 974 | 42 | (+) | NMGATANSG | TGGATACGG |
| M00076 | V$GATA2_01 | 0.82544 | 973 | 43 | (+) | NNNGATRNNN | ATGGATACGG |
| M00075 | V$GATA1_01 | 0.8154 | 973 | 43 | (+) | SNNGATNNNN | ATGGATACGG |
| M00106 | V$CDPCR3HD_01 | 0.83564 | 972 | 44 | (+) | NATYGATSSS | CATGGATACG |
| M00104 | V$CDPCR1_01 | 0.805601 | 972 | 44 | (+) | NATCGATCGS | CATGGATACG |
| M00126 | V$GATA1_02 | 0.88625 | 971 | 45 | (+) | NNNNNGATANKGNN | TCATGGATACGGGT |
| M00039 | V$CREB_01 | 1 | 966 | 50 | (+) | TGACGTMA | TGACGTCA |
| M00039 | V$CREB_01 | 1 | 966 | 50 | (-) | TGACGTMA | TGACGTCA |
| M00041 | V$CREBP1CJUN_01 | 1 | 966 | 50 | (+) | TGACGTYA | TGACGTCA |
| M00041 | V$CREBP1CJUN_01 | 1 | 966 | 50 | (-) | TGACGTYA | TGACGTCA |
| M00113 | V$CREB_02 | 0.923481 | 966 | 50 | (-) | NNGNTGACGYNN | TGACGTCATGGA |
| M00040 | V$CREBP1_01 | 0.911982 | 966 | 50 | (+) | TTACGTAA | TGACGTCA |
| M00040 | V$CREBP1_01 | 0.911982 | 966 | 50 | (-) | TTACGTAA | TGACGTCA |
| M00098 | V$PAX2_01 | 0.817954 | 966 | 50 | (+) | NNNNGTCANGNRTKANNNN | TGACGTCATGGATACGGGT |
| M00260 | V$HLF_01 | 0.852728 | 965 | 51 | (+) | RTTACRYAAT | ATGACGTCAT |
| M00260 | V$HLF_01 | 0.852728 | 965 | 51 | (-) | RTTACRYAAT | ATGACGTCAT |
| M00172 | V$AP1FJ_Q2 | 0.844164 | 965 | 51 | (-) | RSTGACTNMNW | ATGACGTCATG |
| M00228 | V$VBP_01 | 0.843964 | 965 | 51 | (+) | GTTACRTMAK | ATGACGTCAT |
| M00228 | V$VBP_01 | 0.843964 | 965 | 51 | (-) | GTTACRTMAK | ATGACGTCAT |
| M00178 | V$CREB_Q4 | 0.934377 | 964 | 52 | (-) | NSTGACGTMANN | GATGACGTCATG |
| M00178 | V$CREB_Q4 | 0.928319 | 964 | 52 | (+) | NSTGACGTMANN | GATGACGTCATG |
| M00177 | V$CREB_Q2 | 0.91713 | 964 | 52 | (+) | NSTGACGTAANN | GATGACGTCATG |
| M00177 | V$CREB_Q2 | 0.899734 | 964 | 52 | (-) | NSTGACGTAANN | GATGACGTCATG |
| M00172 | V$AP1FJ_Q2 | 0.88612 | 964 | 52 | (+) | RSTGACTNMNW | GATGACGTCAT |
| M00173 | V$AP1_Q2 | 0.851537 | 964 | 52 | (+) | RSTGACTNMNW | GATGACGTCAT |
| M00174 | V$AP1_Q6 | 0.822114 | 964 | 52 | (+) | NNTGACTCANN | GATGACGTCAT |
| M00179 | V$CREBP1_Q2 | 0.811529 | 964 | 52 | (-) | NSTGACGTMASN | GATGACGTCATG |
| M00045 | V$E4BP4_01 | 0.811382 | 964 | 52 | (-) | NRTTAYGTAAYN | GATGACGTCATG |
| M00179 | V$CREBP1_Q2 | 0.806214 | 964 | 52 | (+) | NSTGACGTMASN | GATGACGTCATG |
| M00045 | V$E4BP4_01 | 0.802878 | 964 | 52 | (+) | NRTTAYGTAAYN | GATGACGTCATG |
| M00017 | V$ATF_01 | 0.926567 | 963 | 53 | (-) | CNSTGACGTNNNYC | CGATGACGTCATGG |
| M00017 | V$ATF_01 | 0.908471 | 963 | 53 | (+) | CNSTGACGTNNNYC | CGATGACGTCATGG |
| M00239 | V$T3R_01 | 0.824721 | 963 | 53 | (+) | SNNTRAGGTCACGSNN | CGATGACGTCATGGAT |
| M00113 | V$CREB_02 | 0.968972 | 962 | 54 | (+) | NNGNTGACGYNN | TCGATGACGTCA |
| M00036 | V$VJUN_01 | 0.949603 | 962 | 54 | (+) | NYGATGACGTCATNCY | TCGATGACGTCATGGA |
| M00036 | V$VJUN_01 | 0.889196 | 962 | 54 | (-) | NYGATGACGTCATNCY | TCGATGACGTCATGGA |
| M00251 | V$XBP1_01 | 0.820299 | 962 | 54 | (+) | NNGNTGACGTGKNNNWT | TCGATGACGTCATGGAT |
| M00201 | V$CEBP_C | 0.815193 | 962 | 54 | (+) | NGWNTKNKGYAAKNSAYA | TCGATGACGTCATGGATA |
| M00075 | V$GATA1_01 | 0.847483 | 961 | 55 | (+) | SNNGATNNNN | GTCGATGACG |
| M00239 | V$T3R_01 | 0.819042 | 961 | 55 | (-) | SNNTRAGGTCACGSNN | GTCGATGACGTCATGG |
| M00076 | V$GATA2_01 | 0.81281 | 961 | 55 | (+) | NNNGATRNNN | GTCGATGACG |
| M00106 | V$CDPCR3HD_01 | 0.862745 | 960 | 56 | (+) | NATYGATSSS | GGTCGATGAC |
| M00114 | V$TAXCREB_01 | 0.824848 | 960 | 56 | (+) | GGGGGTTGACGYANA | GGTCGATGACGTCAT |
| M00271 | V$AML1_01 | 1 | 957 | 59 | (+) | TGTGGT | TGTGGT |
| M00217 | V$USF_C | 0.938947 | 954 | 62 | (-) | NCACGTGN | TCATGTGG |
| M00217 | V$USF_C | 0.86783 | 954 | 62 | (+) | NCACGTGN | TCATGTGG |
| M00240 | V$NKX25_01 | 0.852443 | 954 | 62 | (+) | TYAAGTG | TCATGTG |
| M00187 | V$USF_Q6 | 0.885658 | 953 | 63 | (-) | GYCACGTGNC | GTCATGTGGT |
| M00220 | V$SREBP1_01 | 0.866949 | 953 | 63 | (-) | NATCACGTGAY | GTCATGTGGTC |
| M00187 | V$USF_Q6 | 0.847638 | 953 | 63 | (+) | GYCACGTGNC | GTCATGTGGT |
| M00184 | V$MYOD_Q6 | 0.829426 | 953 | 63 | (-) | NNCANCTGNY | GTCATGTGGT |
| M00123 | V$MYCMAX_02 | 0.914631 | 952 | 64 | (-) | NANCACGTGNNW | GGTCATGTGGTC |
| M00277 | V$LMO2COM_01 | 0.875908 | 952 | 64 | (+) | SNNCAGGTGNNN | GGTCATGTGGTC |
| M00001 | V$MYOD_01 | 0.87126 | 952 | 64 | (+) | SRACAGGTGKYG | GGTCATGTGGTC |
| M00055 | V$NMYC_01 | 0.848236 | 952 | 64 | (-) | NNNCACGTGNNN | GGTCATGTGGTC |
| M00220 | V$SREBP1_01 | 0.829147 | 952 | 64 | (+) | NATCACGTGAY | GGTCATGTGGT |
| M00122 | V$USF_02 | 0.914232 | 951 | 65 | (+) | NNRNCACGTGNYNN | AGGTCATGTGGTCG |
| M00122 | V$USF_02 | 0.914232 | 951 | 65 | (-) | NNRNCACGTGNYNN | AGGTCATGTGGTCG |
| M00121 | V$USF_01 | 0.902902 | 951 | 65 | (+) | NNRYCACGTGRYNN | AGGTCATGTGGTCG |
| M00121 | V$USF_01 | 0.902902 | 951 | 65 | (-) | NNRYCACGTGRYNN | AGGTCATGTGGTCG |
| M00119 | V$MAX_01 | 0.821904 | 951 | 65 | (+) | NNANCACGTGNTNN | AGGTCATGTGGTCG |
| M00119 | V$MAX_01 | 0.821904 | 951 | 65 | (-) | NNANCACGTGNTNN | AGGTCATGTGGTCG |
| M00236 | V$ARNT_01 | 0.827618 | 950 | 66 | (+) | NNNNNCACGTGNNNNN | AAGGTCATGTGGTCGA |
| M00041 | V$CREBP1CJUN_01 | 0.829167 | 949 | 67 | (-) | TGACGTYA | AAAGGTCA |
| M00155 | V$ARP1_01 | 0.806747 | 949 | 67 | (-) | TGARCCYTTGAMCCYW | AAAGGTCATGTGGTCG |
| M00162 | V$OCT1_06 | 0.862891 | 947 | 69 | (+) | CWNAWTKWSATRYN | AAAAAGGTCATGTG |
| M00156 | V$RORA1_01 | 0.943597 | 945 | 71 | (+) | NWAWNNAGGTCAN | ACAAAAAGGTCAT |
| M00160 | V$SRY_02 | 0.874927 | 941 | 75 | (+) | NWWAACAAWANN | GAAGACAAAAAG |
| M00079 | V$EVI1_02 | 0.808193 | 938 | 78 | (+) | AGAYAAGATAA | GTAGAAGACAA |
| M00203 | V$GATA_C | 0.900901 | 925 | 91 | (+) | NGATAAGNMNN | TGATAAAAGTA |
| M00252 | V$TATA_01 | 0.813245 | 925 | 91 | (+) | STATAAAWRNNNNNN | TGATAAAAGTATAGT |
| M00077 | V$GATA3_01 | 0.872397 | 924 | 92 | (+) | NNGATARNG | GTGATAAAA |
| M00216 | V$TATA_C | 0.80169 | 924 | 92 | (+) | NCTATAAAAR | GTGATAAAAG |
| M00075 | V$GATA1_01 | 0.83613 | 923 | 93 | (+) | SNNGATNNNN | CGTGATAAAA |
| M00076 | V$GATA2_01 | 0.815516 | 923 | 93 | (+) | NNNGATRNNN | CGTGATAAAA |
| M00128 | V$GATA1_04 | 0.912071 | 922 | 94 | (+) | NNCWGATARNNNN | ACGTGATAAAAGT |
| M00126 | V$GATA1_02 | 0.855 | 921 | 95 | (+) | NNNNNGATANKGNN | TACGTGATAAAAGT |
| M00040 | V$CREBP1_01 | 0.878256 | 920 | 96 | (+) | TTACGTAA | TTACGTGA |
| M00040 | V$CREBP1_01 | 0.878256 | 920 | 96 | (-) | TTACGTAA | TTACGTGA |
| M00039 | V$CREB_01 | 0.857644 | 920 | 96 | (-) | TGACGTMA | TTACGTGA |
| M00039 | V$CREB_01 | 0.835721 | 920 | 96 | (+) | TGACGTMA | TTACGTGA |
| M00041 | V$CREBP1CJUN_01 | 0.823333 | 920 | 96 | (+) | TGACGTYA | TTACGTGA |
| M00217 | V$USF_C | 0.815834 | 920 | 96 | (+) | NCACGTGN | TTACGTGA |
| M00217 | V$USF_C | 0.815834 | 920 | 96 | (-) | NCACGTGN | TTACGTGA |
| M00228 | V$VBP_01 | 0.852605 | 919 | 97 | (+) | GTTACRTMAK | CTTACGTGAT |
| M00073 | V$DELTAEF1_01 | 0.848603 | 919 | 97 | (-) | NNNCACCTNAN | CTTACGTGATA |
| M00220 | V$SREBP1_01 | 0.845561 | 919 | 97 | (-) | NATCACGTGAY | CTTACGTGATA |
| M00260 | V$HLF_01 | 0.841864 | 919 | 97 | (+) | RTTACRYAAT | CTTACGTGAT |
| M00228 | V$VBP_01 | 0.840661 | 919 | 97 | (-) | GTTACRTMAK | CTTACGTGAT |
| M00187 | V$USF_Q6 | 0.810456 | 919 | 97 | (+) | GYCACGTGNC | CTTACGTGAT |
| M00045 | V$E4BP4_01 | 0.847143 | 918 | 98 | (-) | NRTTAYGTAAYN | TCTTACGTGATA |
| M00045 | V$E4BP4_01 | 0.836677 | 918 | 98 | (+) | NRTTAYGTAAYN | TCTTACGTGATA |
| M00123 | V$MYCMAX_02 | 0.835566 | 918 | 98 | (-) | NANCACGTGNNW | TCTTACGTGATA |
| M00251 | V$XBP1_01 | 0.821795 | 916 | 100 | (+) | NNGNTGACGTGKNNNWT | TGTCTTACGTGATAAAA |
| M00098 | V$PAX2_01 | 0.811698 | 914 | 102 | (-) | NNNNGTCANGNRTKANNNN | ACTGTCTTACGTGATAAAA |
| M00194 | V$NFKB_Q6 | 0.800995 | 909 | 107 | (+) | NGGGGAMTTTCCNN | TTTGGACTGTCTTA |
| M00159 | V$CEBP_01 | 0.909562 | 907 | 109 | (+) | NNTKTGGWNANNN | TGTTTGGACTGTC |
| M00254 | V$CAAT_01 | 0.853996 | 907 | 109 | (-) | NNNRRCCAATSA | TGTTTGGACTGT |
| M00148 | V$SRY_01 | 0.94845 | 905 | 111 | (-) | AAACWAM | GGTGTTT |
| M00072 | V$CP2_01 | 0.810892 | 903 | 113 | (-) | GCNMNAMCMAG | CGGGTGTTTGG |
| M00001 | V$MYOD_01 | 0.874803 | 900 | 116 | (+) | SRACAGGTGKYG | CCACGGGTGTTT |
| M00217 | V$USF_C | 0.874203 | 900 | 116 | (-) | NCACGTGN | CCACGGGT |
| M00217 | V$USF_C | 0.831936 | 900 | 116 | (+) | NCACGTGN | CCACGGGT |
| M00277 | V$LMO2COM_01 | 0.821534 | 900 | 116 | (+) | SNNCAGGTGNNN | CCACGGGTGTTT |
| M00187 | V$USF_Q6 | 0.894604 | 899 | 117 | (+) | GYCACGTGNC | CCCACGGGTG |
| M00002 | V$E47_01 | 0.824026 | 899 | 117 | (+) | NSNGCAGGTGKNCNN | CCCACGGGTGTTTGG |
| M00055 | V$NMYC_01 | 0.815085 | 898 | 118 | (+) | NNNCACGTGNNN | TCCCACGGGTGT |
| M00123 | V$MYCMAX_02 | 0.813239 | 898 | 118 | (+) | NANCACGTGNNW | TCCCACGGGTGT |
| M00189 | V$AP2_Q6 | 0.848116 | 897 | 119 | (+) | MKCCCSCNGGCG | ATCCCACGGGTG |
| M00189 | V$AP2_Q6 | 0.808364 | 897 | 119 | (-) | MKCCCSCNGGCG | ATCCCACGGGTG |
| M00189 | V$AP2_Q6 | 0.809283 | 896 | 120 | (+) | MKCCCSCNGGCG | AATCCCACGGGT |
| M00253 | V$CAP_01 | 0.902415 | 894 | 122 | (+) | NCANNNNN | TCAATCCC |
| M00087 | V$IK2_01 | 0.891688 | 894 | 122 | (-) | NNNYGGGAWNNN | TCAATCCCACGG |
| M00008 | V$SP1_01 | 0.81427 | 894 | 122 | (-) | GRGGCRGGGW | TCAATCCCAC |
| M00077 | V$GATA3_01 | 0.84537 | 893 | 123 | (-) | NNGATARNG | TTCAATCCC |
| M00086 | V$IK1_01 | 0.833066 | 893 | 123 | (-) | NNNTGGGAATRCC | TTCAATCCCACGG |
| M00076 | V$GATA2_01 | 0.819125 | 893 | 123 | (-) | NNNGATRNNN | TTCAATCCCA |
| M00249 | V$CHOP_01 | 0.81133 | 890 | 126 | (+) | NNRTGCAATMCCC | TTATTCAATCCCA |
| M00260 | V$HLF_01 | 0.855867 | 889 | 127 | (+) | RTTACRYAAT | GTTATTCAAT |
| M00199 | V$AP1_C | 0.820117 | 889 | 127 | (-) | NTGASTCAG | GTTATTCAA |
| M00117 | V$CEBPB_02 | 0.866807 | 887 | 129 | (-) | NKNTTGCNYAAYNN | TCGTTATTCAATCC |
| M00190 | V$CEBP_Q2 | 0.866397 | 887 | 129 | (-) | NNNTTGCNNAANNN | TCGTTATTCAATCC |
| M00116 | V$CEBPA_01 | 0.847478 | 887 | 129 | (-) | NNATTRCNNAANNN | TCGTTATTCAATCC |
| M00206 | V$HNF1_C | 0.820196 | 886 | 130 | (-) | NGTTAATKAWTNACCAM | TTCGTTATTCAATCCCA |
| M00003 | V$VMYB_01 | 0.834518 | 885 | 131 | (-) | AAYAACGGNN | ATTCGTTATT |
| M00253 | V$CAP_01 | 0.909808 | 883 | 133 | (+) | NCANNNNN | CCATTCGT |
| M00254 | V$CAAT_01 | 0.807964 | 883 | 133 | (-) | NNNRRCCAATSA | CCATTCGTTATT |
| M00185 | V$NFY_Q6 | 0.836074 | 882 | 134 | (-) | TRRCCAATSRN | ACCATTCGTTA |
| M00050 | V$E2F_02 | 0.871702 | 869 | 147 | (-) | TTTSGCGC | GCGCGAGA |
| M00180 | V$E2F_Q6 | 0.801116 | 867 | 149 | (+) | NNGCGCGAAANTK | TCGCGCGAGACCC |
| M00050 | V$E2F_02 | 1 | 865 | 151 | (+) | TTTSGCGC | TTTCGCGC |
| M00180 | V$E2F_Q6 | 0.969488 | 862 | 154 | (-) | NNGCGCGAAANTK | AAATTTCGCGCGA |
| M00024 | V$E2F_01 | 0.861103 | 861 | 155 | (-) | TWSGCGCGAAAAYKR | CAAATTTCGCGCGAG |
| M00253 | V$CAP_01 | 0.875801 | 860 | 156 | (+) | NCANNNNN | TCAAATTT |
| M00106 | V$CDPCR3HD_01 | 0.875721 | 856 | 160 | (-) | NATYGATSSS | GCCATCAAAT |
| M00162 | V$OCT1_06 | 0.873828 | 856 | 160 | (-) | CWNAWTKWSATRYN | GCCATCAAATTTCG |
| M00216 | V$TATA_C | 0.809876 | 856 | 160 | (+) | NCTATAAAAR | GCCATCAAAT |
| M00075 | V$GATA1_01 | 0.86229 | 855 | 161 | (-) | SNNGATNNNN | CGCCATCAAA |
| M00077 | V$GATA3_01 | 0.844484 | 855 | 161 | (-) | NNGATARNG | CGCCATCAA |
| M00076 | V$GATA2_01 | 0.841678 | 855 | 161 | (-) | NNNGATRNNN | CGCCATCAAA |
| M00162 | V$OCT1_06 | 0.808984 | 855 | 161 | (+) | CWNAWTKWSATRYN | CGCCATCAAATTTC |
| M00050 | V$E2F_02 | 0.828381 | 850 | 166 | (+) | TTTSGCGC | ATTGCCGC |
| M00184 | V$MYOD_Q6 | 0.84269 | 846 | 170 | (+) | NNCANCTGNY | GGCAATTGCC |
| M00184 | V$MYOD_Q6 | 0.84269 | 846 | 170 | (-) | NNCANCTGNY | GGCAATTGCC |
| M00194 | V$NFKB_Q6 | 0.811982 | 844 | 172 | (+) | NGGGGAMTTTCCNN | AGGGCAATTGCCGC |
| M00249 | V$CHOP_01 | 0.816679 | 843 | 173 | (+) | NNRTGCAATMCCC | AAGGGCAATTGCC |
| M00141 | V$LYF1_01 | 0.896243 | 836 | 180 | (-) | TTTGGGAGR | TATCTCAAA |
| M00160 | V$SRY_02 | 0.805637 | 836 | 180 | (+) | NWWAACAAWANN | TATCTCAAAGGG |
| M00077 | V$GATA3_01 | 0.899867 | 833 | 183 | (-) | NNGATARNG | TGTTATCTC |
| M00278 | V$LMO2COM_02 | 0.899596 | 833 | 183 | (-) | NMGATANSG | TGTTATCTC |
| M00076 | V$GATA2_01 | 0.876861 | 833 | 183 | (-) | NNNGATRNNN | TGTTATCTCA |
| M00211 | V$PADS_C | 0.852823 | 833 | 183 | (+) | NGTGGTCTC | TGTTATCTC |
| M00128 | V$GATA1_04 | 0.856924 | 831 | 185 | (-) | NNCWGATARNNNN | TATGTTATCTCAA |
| M00126 | V$GATA1_02 | 0.81375 | 831 | 185 | (-) | NNNNNGATANKGNN | TATGTTATCTCAAA |
| M00080 | V$EVI1_03 | 0.904938 | 830 | 186 | (-) | AGATAAGATAA | TTATGTTATCT |
| M00082 | V$EVI1_05 | 0.902538 | 830 | 186 | (-) | AGATAAGATAN | TTATGTTATCT |
| M00079 | V$EVI1_02 | 0.901429 | 830 | 186 | (-) | AGAYAAGATAA | TTATGTTATCT |
| M00203 | V$GATA_C | 0.856477 | 830 | 186 | (-) | NGATAAGNMNN | TTATGTTATCT |
| M00040 | V$CREBP1_01 | 0.816013 | 830 | 186 | (-) | TTACGTAA | TTATGTTA |
| M00260 | V$HLF_01 | 0.908498 | 829 | 187 | (-) | RTTACRYAAT | ATTATGTTAT |
| M00228 | V$VBP_01 | 0.852097 | 829 | 187 | (-) | GTTACRTMAK | ATTATGTTAT |
| M00260 | V$HLF_01 | 0.845727 | 829 | 187 | (+) | RTTACRYAAT | ATTATGTTAT |
| M00228 | V$VBP_01 | 0.831258 | 829 | 187 | (+) | GTTACRTMAK | ATTATGTTAT |
| M00269 | V$XFD3_01 | 0.826026 | 829 | 187 | (-) | WNWGTMAACAWWMW | ATTATGTTATCTCA |
| M00045 | V$E4BP4_01 | 0.851068 | 828 | 188 | (+) | NRTTAYGTAAYN | GATTATGTTATC |
| M00160 | V$SRY_02 | 0.807986 | 828 | 188 | (-) | NWWAACAAWANN | GATTATGTTATC |
| M00109 | V$CEBPB_01 | 0.860327 | 827 | 189 | (+) | RNRTKNNGMAAKNN | AGATTATGTTATCT |
| M00077 | V$GATA3_01 | 0.899867 | 826 | 190 | (+) | NNGATARNG | AAGATTATG |
| M00137 | V$OCT1_03 | 0.856578 | 824 | 192 | (-) | NNNRTAATNANNN | AAAAGATTATGTT |
| M00011 | V$EVI1_06 | 0.825401 | 824 | 192 | (+) | ACAAGATAA | AAAAGATTA |
| M00129 | V$HFH1_01 | 0.803711 | 824 | 192 | (+) | NAWTGTTTATWT | AAAAGATTATGT |
| M00096 | V$PBX1_01 | 0.892041 | 819 | 197 | (+) | ANCAATCAW | AACAAAAAA |
| M00042 | V$SOX5_01 | 0.806264 | 817 | 199 | (+) | NNAACAATNN | CCAACAAAAA |
| M00160 | V$SRY_02 | 0.847622 | 816 | 200 | (+) | NWWAACAAWANN | ACCAACAAAAAA |
| M00253 | V$CAP_01 | 0.876787 | 814 | 202 | (+) | NCANNNNN | TCACCAAC |
| M00083 | V$MZF1_01 | 0.837552 | 814 | 202 | (-) | NGNGGGGA | TCACCAAC |
| M00148 | V$SRY_01 | 0.91124 | 812 | 204 | (+) | AAACWAM | AATCACC |
| M00075 | V$GATA1_01 | 0.878085 | 809 | 207 | (-) | SNNGATNNNN | TCAAATCACC |
| M00077 | V$GATA3_01 | 0.847585 | 809 | 207 | (-) | NNGATARNG | TCAAATCAC |
| M00076 | V$GATA2_01 | 0.817772 | 809 | 207 | (-) | NNNGATRNNN | TCAAATCACC |
| M00172 | V$AP1FJ_Q2 | 0.812618 | 808 | 208 | (-) | RSTGACTNMNW | TTCAAATCACC |
| M00173 | V$AP1_Q2 | 0.8041 | 808 | 208 | (-) | RSTGACTNMNW | TTCAAATCACC |
| M00082 | V$EVI1_05 | 0.812753 | 806 | 210 | (-) | AGATAAGATAN | ATTTCAAATCA |
| M00206 | V$HNF1_C | 0.803222 | 806 | 210 | (-) | NGTTAATKAWTNACCAM | ATTTCAAATCACCAACA |
| M00160 | V$SRY_02 | 0.806812 | 805 | 211 | (+) | NWWAACAAWANN | CATTTCAAATCA |
| M00133 | V$TST1_01 | 0.896726 | 804 | 212 | (-) | NNKGAWTWANANTNN | GCATTTCAAATCACC |
| M00109 | V$CEBPB_01 | 0.884596 | 804 | 212 | (-) | RNRTKNNGMAAKNN | GCATTTCAAATCAC |
| M00253 | V$CAP_01 | 0.876294 | 804 | 212 | (+) | NCANNNNN | GCATTTCA |
| M00162 | V$OCT1_06 | 0.842187 | 803 | 213 | (-) | CWNAWTKWSATRYN | AGCATTTCAAATCA |
| M00195 | V$OCT1_Q6 | 0.825328 | 803 | 213 | (+) | NNNNATGCAAATNAN | AGCATTTCAAATCAC |
| M00145 | V$BRN2_01 | 0.885752 | 802 | 214 | (-) | NNCATNSRWAATNMRN | AAGCATTTCAAATCAC |
| M00272 | V$P53_02 | 0.826788 | 802 | 214 | (-) | NGRCWTGYCY | AAGCATTTCA |
| M00162 | V$OCT1_06 | 0.816016 | 802 | 214 | (+) | CWNAWTKWSATRYN | AAGCATTTCAAATC |
| M00249 | V$CHOP_01 | 0.802577 | 802 | 214 | (-) | NNRTGCAATMCCC | AAGCATTTCAAAT |
| M00272 | V$P53_02 | 0.86313 | 798 | 218 | (-) | NGRCWTGYCY | TGGCAAGCAT |
| M00235 | V$AHRARNT_01 | 0.832617 | 798 | 218 | (-) | KNNKNNTYGCGTGCMS | TGGCAAGCATTTCAAA |
| M00272 | V$P53_02 | 0.802169 | 798 | 218 | (+) | NGRCWTGYCY | TGGCAAGCAT |
| M00193 | V$NF1_Q6 | 0.838384 | 795 | 221 | (+) | NNTTGGCNNNNNNCCNNN | AACTGGCAAGCATTTCAA |
| M00183 | V$MYB_Q6 | 0.923237 | 792 | 224 | (+) | NNNAACKGNC | TTGAACTGGC |
| M00222 | V$TH1E47_01 | 0.82964 | 789 | 227 | (+) | NNNNGNRTCTGGMWTT | AATTTGAACTGGCAAG |
| M00158 | V$COUP_01 | 0.892208 | 786 | 230 | (+) | TGAMCTTTGMMCYT | TGAAATTTGAACTG |
| M00133 | V$TST1_01 | 0.888153 | 785 | 231 | (+) | NNKGAWTWANANTNN | CTGAAATTTGAACTG |
| M00134 | V$HNF4_01 | 0.83598 | 783 | 233 | (-) | NNNRGGNCAAAGKTCANNN | AGCTGAAATTTGAACTGGC |
| M00162 | V$OCT1_06 | 0.816406 | 781 | 235 | (+) | CWNAWTKWSATRYN | AAAGCTGAAATTTG |
| M00175 | V$AP4_Q5 | 0.805767 | 780 | 236 | (+) | NNCAGCTGNN | CAAAGCTGAA |
| M00199 | V$AP1_C | 0.823032 | 776 | 240 | (-) | NTGASTCAG | GTGACAAAG |
| M00172 | V$AP1FJ_Q2 | 0.881388 | 775 | 241 | (+) | RSTGACTNMNW | CGTGACAAAGC |
| M00188 | V$AP1_Q4 | 0.864593 | 775 | 241 | (+) | RSTGACTMANN | CGTGACAAAGC |
| M00174 | V$AP1_Q6 | 0.853328 | 775 | 241 | (+) | NNTGACTCANN | CGTGACAAAGC |
| M00173 | V$AP1_Q2 | 0.84246 | 775 | 241 | (+) | RSTGACTNMNW | CGTGACAAAGC |
| M00177 | V$CREB_Q2 | 0.817347 | 775 | 241 | (+) | NSTGACGTAANN | CGTGACAAAGCT |
| M00217 | V$USF_C | 0.821536 | 772 | 244 | (+) | NCACGTGN | TCGCGTGA |
| M00217 | V$USF_C | 0.815834 | 772 | 244 | (-) | NCACGTGN | TCGCGTGA |
| M00187 | V$USF_Q6 | 0.819961 | 771 | 245 | (-) | GYCACGTGNC | TTCGCGTGAC |
| M00187 | V$USF_Q6 | 0.805144 | 771 | 245 | (+) | GYCACGTGNC | TTCGCGTGAC |
| M00055 | V$NMYC_01 | 0.828163 | 770 | 246 | (+) | NNNCACGTGNNN | GTTCGCGTGACA |
| M00055 | V$NMYC_01 | 0.821776 | 770 | 246 | (-) | NNNCACGTGNNN | GTTCGCGTGACA |
| M00220 | V$SREBP1_01 | 0.813231 | 770 | 246 | (+) | NATCACGTGAY | GTTCGCGTGAC |
| M00121 | V$USF_01 | 0.814776 | 769 | 247 | (+) | NNRYCACGTGRYNN | GGTTCGCGTGACAA |
| M00121 | V$USF_01 | 0.814776 | 769 | 247 | (-) | NNRYCACGTGRYNN | GGTTCGCGTGACAA |
| M00122 | V$USF_02 | 0.804537 | 769 | 247 | (+) | NNRNCACGTGNYNN | GGTTCGCGTGACAA |
| M00122 | V$USF_02 | 0.804537 | 769 | 247 | (-) | NNRNCACGTGNYNN | GGTTCGCGTGACAA |
| M00236 | V$ARNT_01 | 0.809436 | 768 | 248 | (-) | NNNNNCACGTGNNNNN | AGGTTCGCGTGACAAA |
| M00237 | V$AHRARNT_02 | 0.855667 | 767 | 249 | (+) | GRGKATYGCGTGMSWNSCC | AAGGTTCGCGTGACAAAGC |
| M00180 | V$E2F_Q6 | 0.850977 | 767 | 249 | (-) | NNGCGCGAAANTK | AAGGTTCGCGTGA |
| M00235 | V$AHRARNT_01 | 0.935953 | 766 | 250 | (+) | KNNKNNTYGCGTGCMS | AAAGGTTCGCGTGACA |
| M00098 | V$PAX2_01 | 0.812637 | 766 | 250 | (-) | NNNNGTCANGNRTKANNNN | AAAGGTTCGCGTGACAAAG |
| M00100 | V$CDXA_01 | 0.929377 | 763 | 253 | (-) | MTTTATR | GATAAAG |
| M00227 | V$VMYB_02 | 0.80355 | 763 | 253 | (+) | NSYAACGGN | GATAAAGGT |
| M00203 | V$GATA_C | 0.924821 | 762 | 254 | (+) | NGATAAGNMNN | AGATAAAGGTT |
| M00077 | V$GATA3_01 | 0.930882 | 761 | 255 | (+) | NNGATARNG | GAGATAAAG |
| M00278 | V$LMO2COM_02 | 0.886792 | 761 | 255 | (+) | NMGATANSG | GAGATAAAG |
| M00076 | V$GATA2_01 | 0.875056 | 760 | 256 | (+) | NNNGATRNNN | GGAGATAAAG |
| M00075 | V$GATA1_01 | 0.857848 | 760 | 256 | (+) | SNNGATNNNN | GGAGATAAAG |
| M00128 | V$GATA1_04 | 0.935049 | 759 | 257 | (+) | NNCWGATARNNNN | AGGAGATAAAGGT |
| M00134 | V$HNF4_01 | 0.8049 | 759 | 257 | (+) | NNNRGGNCAAAGKTCANNN | AGGAGATAAAGGTTCGCGT |
| M00126 | V$GATA1_02 | 0.896875 | 758 | 258 | (+) | NNNNNGATANKGNN | TAGGAGATAAAGGT |
| M00127 | V$GATA1_03 | 0.819451 | 758 | 258 | (+) | RNSNNGATAANNGN | TAGGAGATAAAGGT |
| M00077 | V$GATA3_01 | 0.918033 | 754 | 262 | (+) | NNGATARNG | ACGATAGGA |
| M00278 | V$LMO2COM_02 | 0.898922 | 754 | 262 | (+) | NMGATANSG | ACGATAGGA |
| M00075 | V$GATA1_01 | 0.918559 | 753 | 263 | (+) | SNNGATNNNN | CACGATAGGA |
| M00076 | V$GATA2_01 | 0.913396 | 753 | 263 | (+) | NNNGATRNNN | CACGATAGGA |
| M00126 | V$GATA1_02 | 0.884375 | 751 | 265 | (+) | NNNNNGATANKGNN | ACCACGATAGGAGA |
| M00271 | V$AML1_01 | 0.855588 | 751 | 265 | (-) | TGTGGT | ACCACG |
| M00127 | V$GATA1_03 | 0.817981 | 751 | 265 | (+) | RNSNNGATAANNGN | ACCACGATAGGAGA |
| M00211 | V$PADS_C | 0.829889 | 748 | 268 | (-) | NGTGGTCTC | CTGACCACG |
| M00156 | V$RORA1_01 | 0.80029 | 748 | 268 | (-) | NWAWNNAGGTCAN | CTGACCACGATAG |
| M00172 | V$AP1FJ_Q2 | 0.902208 | 747 | 269 | (+) | RSTGACTNMNW | GCTGACCACGA |
| M00173 | V$AP1_Q2 | 0.902196 | 747 | 269 | (+) | RSTGACTNMNW | GCTGACCACGA |
| M00188 | V$AP1_Q4 | 0.867338 | 747 | 269 | (+) | RSTGACTMANN | GCTGACCACGA |
| M00174 | V$AP1_Q6 | 0.821737 | 747 | 269 | (+) | NNTGACTCANN | GCTGACCACGA |
| M00008 | V$SP1_01 | 0.800396 | 744 | 272 | (+) | GRGGCRGGGW | TAGGCTGACC |
| M00277 | V$LMO2COM_01 | 0.801346 | 735 | 281 | (+) | SNNCAGGTGNNN | ATGCAAATGTAG |
| M00210 | V$OCT_C | 0.928514 | 733 | 283 | (-) | CTNATTTGCATAY | ATATGCAAATGTA |
| M00159 | V$CEBP_01 | 0.882658 | 732 | 284 | (+) | NNTKTGGWNANNN | AATATGCAAATGT |
| M00162 | V$OCT1_06 | 0.868359 | 732 | 284 | (-) | CWNAWTKWSATRYN | AATATGCAAATGTA |
| M00161 | V$OCT1_05 | 0.862859 | 732 | 284 | (-) | MKNATTTGCATAYY | AATATGCAAATGTA |
| M00248 | V$OCT1_07 | 0.853933 | 732 | 284 | (+) | TNTATGNTAATT | AATATGCAAATG |
| M00228 | V$VBP_01 | 0.83202 | 732 | 284 | (-) | GTTACRTMAK | AATATGCAAA |
| M00159 | V$CEBP_01 | 0.891734 | 731 | 285 | (-) | NNTKTGGWNANNN | GAATATGCAAATG |
| M00195 | V$OCT1_Q6 | 0.854258 | 731 | 285 | (+) | NNNNATGCAAATNAN | GAATATGCAAATGTA |
| M00109 | V$CEBPB_01 | 0.84002 | 730 | 286 | (+) | RNRTKNNGMAAKNN | TGAATATGCAAATG |
| M00116 | V$CEBPA_01 | 0.818928 | 730 | 286 | (-) | NNATTRCNNAANNN | TGAATATGCAAATG |
| M00147 | V$HSF2_01 | 0.817745 | 730 | 286 | (+) | NGAANNWTCK | TGAATATGCA |
| M00146 | V$HSF1_01 | 0.806223 | 730 | 286 | (+) | RGAANRTTCN | TGAATATGCA |
| M00136 | V$OCT1_02 | 0.974012 | 729 | 287 | (+) | NNGAATATKCANNNN | ATGAATATGCAAATG |
| M00135 | V$OCT1_01 | 0.963373 | 729 | 287 | (+) | NNNNWTATGCAAATNTNNN | ATGAATATGCAAATGTAGG |
| M00138 | V$OCT1_04 | 0.885822 | 727 | 289 | (+) | NNNNNNNWATGCAAATNNNWNNW | CTATGAATATGCAAATGTAGGCT |
| M00252 | V$TATA_01 | 0.824918 | 727 | 289 | (+) | STATAAAWRNNNNNN | CTATGAATATGCAAA |
| M00059 | V$YY1_01 | 0.805374 | 727 | 289 | (-) | NNNNNCCATNTWNNNWN | CTATGAATATGCAAATG |
| M00136 | V$OCT1_02 | 0.888468 | 726 | 290 | (-) | NNGAATATKCANNNN | ACTATGAATATGCAA |
| M00268 | V$XFD2_01 | 0.85 | 726 | 290 | (+) | WNWATAAACAWNNR | ACTATGAATATGCA |
| M00138 | V$OCT1_04 | 0.815977 | 726 | 290 | (-) | NNNNNNNWATGCAAATNNNWNNW | ACTATGAATATGCAAATGTAGGC |
| M00101 | V$CDXA_02 | 0.981893 | 723 | 293 | (+) | WWTWMTR | ATTACTA |
| M00248 | V$OCT1_07 | 0.840153 | 722 | 294 | (-) | TNTATGNTAATT | AATTACTATGAA |
| M00138 | V$OCT1_04 | 0.84128 | 721 | 295 | (+) | NNNNNNNWATGCAAATNNNWNNW | GAATTACTATGAATATGCAAATG |
| M00138 | V$OCT1_04 | 0.854663 | 720 | 296 | (-) | NNNNNNNWATGCAAATNNNWNNW | TGAATTACTATGAATATGCAAAT |
| M00133 | V$TST1_01 | 0.863601 | 718 | 298 | (+) | NNKGAWTWANANTNN | CTTGAATTACTATGA |
| M00138 | V$OCT1_04 | 0.803011 | 716 | 300 | (-) | NNNNNNNWATGCAAATNNNWNNW | AGCTTGAATTACTATGAATATGC |
| M00281 | V$RFX1_02 | 0.807403 | 711 | 305 | (-) | NNGTNRCNATRGYAACNNN | ATCGTAGCTTGAATTACTA |
| M00082 | V$EVI1_05 | 0.848155 | 709 | 307 | (-) | AGATAAGATAN | TTATCGTAGCT |
| M00080 | V$EVI1_03 | 0.809877 | 709 | 307 | (-) | AGATAAGATAA | TTATCGTAGCT |
| M00079 | V$EVI1_02 | 0.802859 | 709 | 307 | (-) | AGAYAAGATAA | TTATCGTAGCT |
| M00077 | V$GATA3_01 | 0.848914 | 707 | 309 | (-) | NNGATARNG | TTTTATCGT |
| M00278 | V$LMO2COM_02 | 0.813342 | 707 | 309 | (-) | NMGATANSG | TTTTATCGT |
| M00076 | V$GATA2_01 | 0.806495 | 707 | 309 | (-) | NNNGATRNNN | TTTTATCGTA |
| M00160 | V$SRY_02 | 0.833235 | 702 | 314 | (-) | NWWAACAAWANN | TCTTTTTTTATC |
| M00148 | V$SRY_01 | 0.960465 | 700 | 316 | (-) | AAACWAM | TTTCTTT |
| M00081 | V$EVI1_04 | 0.897038 | 700 | 316 | (-) | NGATANGANWAGATA | TTTCTTTTTTTATCG |
| M00241 | V$NKX25_02 | 0.839809 | 696 | 320 | (+) | CWTAATTG | CTTATTTC |
| M00131 | V$HNF3B_01 | 0.87867 | 695 | 321 | (+) | NNNTRTTTRYTY | ACTTATTTCTTT |
| M00240 | V$NKX25_01 | 0.941233 | 694 | 322 | (-) | TYAAGTG | CACTTAT |
| M00001 | V$MYOD_01 | 0.809252 | 691 | 325 | (-) | SRACAGGTGKYG | CAACACTTATTT |
| M00042 | V$SOX5_01 | 0.812076 | 690 | 326 | (+) | NNAACAATNN | GCAACACTTA |
| M00255 | V$GC_01 | 0.818226 | 688 | 328 | (-) | NRGGGGCGGGGCNK | AAGCAACACTTATT |
| M00147 | V$HSF2_01 | 0.801109 | 686 | 330 | (+) | NGAANNWTCK | TGAAGCAACA |
| M00228 | V$VBP_01 | 0.817281 | 685 | 331 | (-) | GTTACRTMAK | CTGAAGCAAC |
| M00178 | V$CREB_Q4 | 0.811964 | 684 | 332 | (+) | NSTGACGTMANN | TCTGAAGCAACA |
| M00177 | V$CREB_Q2 | 0.809857 | 684 | 332 | (+) | NSTGACGTAANN | TCTGAAGCAACA |
| M00117 | V$CEBPB_02 | 0.869966 | 683 | 333 | (-) | NKNTTGCNYAAYNN | CTCTGAAGCAACAC |
| M00190 | V$CEBP_Q2 | 0.84873 | 683 | 333 | (-) | NNNTTGCNNAANNN | CTCTGAAGCAACAC |
| M00116 | V$CEBPA_01 | 0.809934 | 683 | 333 | (-) | NNATTRCNNAANNN | CTCTGAAGCAACAC |
| M00223 | V$STAT_01 | 0.853542 | 681 | 335 | (+) | TTCCCRKAA | TTCTCTGAA |
| M00223 | V$STAT_01 | 0.833627 | 681 | 335 | (-) | TTCCCRKAA | TTCTCTGAA |
| M00141 | V$LYF1_01 | 0.844365 | 680 | 336 | (-) | TTTGGGAGR | TTTCTCTGA |
| M00147 | V$HSF2_01 | 0.801848 | 677 | 339 | (-) | NGAANNWTCK | TGATTTCTCT |
| M00148 | V$SRY_01 | 0.947287 | 675 | 341 | (-) | AAACWAM | GTTGATT |
| M00075 | V$GATA1_01 | 0.836624 | 675 | 341 | (+) | SNNGATNNNN | GTTGATTTCT |
| M00106 | V$CDPCR3HD_01 | 0.88293 | 674 | 342 | (+) | NATYGATSSS | GGTTGATTTC |
| M00269 | V$XFD3_01 | 0.800421 | 673 | 343 | (-) | WNWGTMAACAWWMW | TGGTTGATTTCTCT |
| M00271 | V$AML1_01 | 0.873587 | 671 | 345 | (+) | TGTGGT | TATGGT |
| M00129 | V$HFH1_01 | 0.808349 | 671 | 345 | (+) | NAWTGTTTATWT | TATGGTTGATTT |
| M00042 | V$SOX5_01 | 0.824346 | 670 | 346 | (-) | NNAACAATNN | CTATGGTTGA |
| M00254 | V$CAAT_01 | 0.832251 | 669 | 347 | (-) | NNNRRCCAATSA | CCTATGGTTGAT |
| M00076 | V$GATA2_01 | 0.806946 | 668 | 348 | (-) | NNNGATRNNN | CCCTATGGTT |
| M00278 | V$LMO2COM_02 | 0.802898 | 668 | 348 | (-) | NMGATANSG | CCCTATGGT |
| M00053 | V$CREL_01 | 0.817575 | 661 | 355 | (-) | SGGRNWTTCC | GCAAATGCCC |
| M00255 | V$GC_01 | 0.809162 | 660 | 356 | (-) | NRGGGGCGGGGCNK | CGCAAATGCCCTAT |
| M00277 | V$LMO2COM_01 | 0.836608 | 659 | 357 | (+) | SNNCAGGTGNNN | CCGCAAATGCCC |
| M00271 | V$AML1_01 | 0.857262 | 658 | 358 | (-) | TGTGGT | GCCGCA |
| M00050 | V$E2F_02 | 0.828381 | 658 | 358 | (-) | TTTSGCGC | GCCGCAAA |
| M00032 | V$CETS1P54_01 | 0.822379 | 658 | 358 | (+) | NCMGGAWGYN | GCCGCAAATG |
| M00253 | V$CAP_01 | 0.883687 | 652 | 364 | (+) | NCANNNNN | TCATAGGC |
| M00254 | V$CAAT_01 | 0.829144 | 652 | 364 | (-) | NNNRRCCAATSA | TCATAGGCCGCA |
| M00057 | V$COMP1_01 | 0.830778 | 647 | 369 | (+) | NNTNWKGATTGRCNRSRANMRRNN | TTTTTTCATAGGCCGCAAATGCCC |
| M00148 | V$SRY_01 | 0.960465 | 641 | 375 | (-) | AAACWAM | TTTCTTT |
| M00109 | V$CEBPB_01 | 0.820456 | 638 | 378 | (-) | RNRTKNNGMAAKNN | ATCTTTCTTTTTTT |
| M00162 | V$OCT1_06 | 0.808203 | 636 | 380 | (+) | CWNAWTKWSATRYN | GAATCTTTCTTTTT |
| M00147 | V$HSF2_01 | 0.801848 | 635 | 381 | (-) | NGAANNWTCK | AGAATCTTTC |
| M00076 | V$GATA2_01 | 0.805142 | 634 | 382 | (-) | NNNGATRNNN | GAGAATCTTT |
| M00223 | V$STAT_01 | 0.853542 | 630 | 386 | (-) | TTCCCRKAA | TTCAGAGAA |
| M00223 | V$STAT_01 | 0.833627 | 630 | 386 | (+) | TTCCCRKAA | TTCAGAGAA |
| M00254 | V$CAAT_01 | 0.821519 | 622 | 394 | (+) | NNNRRCCAATSA | ATATACCATTCA |
| M00209 | V$NFY_C | 0.80484 | 622 | 394 | (-) | NCTGATTGGYTASY | ATATACCATTCAGA |
| M00159 | V$CEBP_01 | 0.873582 | 621 | 395 | (-) | NNTKTGGWNANNN | CATATACCATTCA |
| M00162 | V$OCT1_06 | 0.860937 | 621 | 395 | (-) | CWNAWTKWSATRYN | CATATACCATTCAG |
| M00222 | V$TH1E47_01 | 0.828211 | 616 | 400 | (-) | NNNNGNRTCTGGMWTT | ATGGCCATATACCATT |
| M00059 | V$YY1_01 | 0.873526 | 615 | 401 | (+) | NNNNNCCATNTWNNNWN | AATGGCCATATACCATT |
| M00248 | V$OCT1_07 | 0.800933 | 615 | 401 | (-) | TNTATGNTAATT | AATGGCCATATA |
| M00109 | V$CEBPB_01 | 0.813522 | 614 | 402 | (-) | RNRTKNNGMAAKNN | CAATGGCCATATAC |
| M00254 | V$CAAT_01 | 0.849478 | 613 | 403 | (-) | NNNRRCCAATSA | GCAATGGCCATA |
| M00227 | V$VMYB_02 | 0.844164 | 612 | 404 | (+) | NSYAACGGN | TGCAATGGC |
| M00249 | V$CHOP_01 | 0.892293 | 609 | 407 | (+) | NNRTGCAATMCCC | ATCTGCAATGGCC |
| M00158 | V$COUP_01 | 0.820227 | 609 | 407 | (-) | TGAMCTTTGMMCYT | ATCTGCAATGGCCA |
| M00059 | V$YY1_01 | 0.823722 | 608 | 408 | (-) | NNNNNCCATNTWNNNWN | GATCTGCAATGGCCATA |
| M00057 | V$COMP1_01 | 0.816168 | 608 | 408 | (+) | NNTNWKGATTGRCNRSRANMRRNN | GATCTGCAATGGCCATATACCATT |
| M00075 | V$GATA1_01 | 0.838105 | 605 | 411 | (-) | SNNGATNNNN | CTGGATCTGC |
| M00076 | V$GATA2_01 | 0.836716 | 605 | 411 | (-) | NNNGATRNNN | CTGGATCTGC |
| M00032 | V$CETS1P54_01 | 0.843835 | 604 | 412 | (+) | NCMGGAWGYN | TCTGGATCTG |
| M00222 | V$TH1E47_01 | 0.849655 | 603 | 413 | (+) | NNNNGNRTCTGGMWTT | ATCTGGATCTGCAATG |
| M00074 | V$CETS1P54_02 | 0.849476 | 602 | 414 | (+) | NNAMMGGAWRWNN | GATCTGGATCTGC |
| M00075 | V$GATA1_01 | 0.843534 | 599 | 417 | (-) | SNNGATNNNN | ACTGATCTGG |
| M00076 | V$GATA2_01 | 0.838521 | 599 | 417 | (-) | NNNGATRNNN | ACTGATCTGG |
| M00075 | V$GATA1_01 | 0.824778 | 599 | 417 | (+) | SNNGATNNNN | ACTGATCTGG |
| M00076 | V$GATA2_01 | 0.813712 | 599 | 417 | (+) | NNNGATRNNN | ACTGATCTGG |
| M00106 | V$CDPCR3HD_01 | 0.839965 | 598 | 418 | (+) | NATYGATSSS | GACTGATCTG |
| M00104 | V$CDPCR1_01 | 0.808206 | 598 | 418 | (+) | NATCGATCGS | GACTGATCTG |
| M00147 | V$HSF2_01 | 0.864695 | 597 | 419 | (+) | NGAANNWTCK | AGACTGATCT |
| M00222 | V$TH1E47_01 | 0.853467 | 597 | 419 | (+) | NNNNGNRTCTGGMWTT | AGACTGATCTGGATCT |
| M00147 | V$HSF2_01 | 0.843623 | 597 | 419 | (-) | NGAANNWTCK | AGACTGATCT |
| M00146 | V$HSF1_01 | 0.825916 | 597 | 419 | (-) | RGAANRTTCN | AGACTGATCT |
| M00272 | V$P53_02 | 0.818581 | 597 | 419 | (-) | NGRCWTGYCY | AGACTGATCT |
| M00253 | V$CAP_01 | 0.898965 | 595 | 421 | (+) | NCANNNNN | GCAGACTG |
| M00205 | V$GRE_C | 0.805692 | 592 | 424 | (+) | GGTACAANNTGTYCTK | TGCGCAGACTGATCTG |
| M00116 | V$CEBPA_01 | 0.821666 | 588 | 428 | (+) | NNATTRCNNAANNN | CCTTTGCGCAGACT |
| M00227 | V$VMYB_02 | 0.834838 | 587 | 429 | (-) | NSYAACGGN | GCCTTTGCG |
| M00201 | V$CEBP_C | 0.878385 | 584 | 432 | (-) | NGWNTKNKGYAAKNSAYA | TGCGCCTTTGCGCAGACT |
| M00155 | V$ARP1_01 | 0.805293 | 584 | 432 | (+) | TGARCCYTTGAMCCYW | TGCGCCTTTGCGCAGA |
| M00008 | V$SP1_01 | 0.82333 | 582 | 434 | (-) | GRGGCRGGGW | TCTGCGCCTT |
| M00253 | V$CAP_01 | 0.967965 | 578 | 438 | (+) | NCANNNNN | TCAGTCTG |
| M00147 | V$HSF2_01 | 0.864695 | 575 | 441 | (-) | NGAANNWTCK | AGATCAGTCT |
| M00147 | V$HSF2_01 | 0.843623 | 575 | 441 | (+) | NGAANNWTCK | AGATCAGTCT |
| M00146 | V$HSF1_01 | 0.825916 | 575 | 441 | (+) | RGAANRTTCN | AGATCAGTCT |
| M00272 | V$P53_02 | 0.818581 | 575 | 441 | (+) | NGRCWTGYCY | AGATCAGTCT |
| M00106 | V$CDPCR3HD_01 | 0.839965 | 574 | 442 | (-) | NATYGATSSS | CAGATCAGTC |
| M00205 | V$GRE_C | 0.817391 | 574 | 442 | (-) | GGTACAANNTGTYCTK | CAGATCAGTCTGCGCC |
| M00104 | V$CDPCR1_01 | 0.808206 | 574 | 442 | (-) | NATCGATCGS | CAGATCAGTC |
| M00075 | V$GATA1_01 | 0.843534 | 573 | 443 | (+) | SNNGATNNNN | CCAGATCAGT |
| M00076 | V$GATA2_01 | 0.838521 | 573 | 443 | (+) | NNNGATRNNN | CCAGATCAGT |
| M00075 | V$GATA1_01 | 0.824778 | 573 | 443 | (-) | SNNGATNNNN | CCAGATCAGT |
| M00076 | V$GATA2_01 | 0.813712 | 573 | 443 | (-) | NNNGATRNNN | CCAGATCAGT |
| M00222 | V$TH1E47_01 | 0.853467 | 569 | 447 | (-) | NNNNGNRTCTGGMWTT | AGATCCAGATCAGTCT |
| M00032 | V$CETS1P54_01 | 0.843835 | 568 | 448 | (-) | NCMGGAWGYN | CAGATCCAGA |
| M00253 | V$CAP_01 | 0.927058 | 567 | 449 | (+) | NCANNNNN | TCAGATCC |
| M00074 | V$CETS1P54_02 | 0.855694 | 567 | 449 | (-) | NNAMMGGAWRWNN | TCAGATCCAGATC |
| M00076 | V$GATA2_01 | 0.822282 | 567 | 449 | (+) | NNNGATRNNN | TCAGATCCAG |
| M00249 | V$CHOP_01 | 0.84318 | 560 | 456 | (-) | NNRTGCAATMCCC | GGCCATTTCAGAT |
| M00053 | V$CREL_01 | 0.845662 | 559 | 457 | (+) | SGGRNWTTCC | TGGCCATTTC |
| M00059 | V$YY1_01 | 0.866972 | 557 | 459 | (+) | NNNNNCCATNTWNNNWN | AATGGCCATTTCAGATC |
| M00254 | V$CAAT_01 | 0.856538 | 555 | 461 | (-) | NNNRRCCAATSA | TAAATGGCCATT |
| M00241 | V$NKX25_02 | 0.831926 | 553 | 463 | (+) | CWTAATTG | GTTAAATG |
| M00099 | V$S8_01 | 0.812389 | 551 | 465 | (-) | WNNANYYAATTANYNN | ATGTTAAATGGCCATT |
| M00059 | V$YY1_01 | 0.875491 | 550 | 466 | (-) | NNNNNCCATNTWNNNWN | AATGTTAAATGGCCATT |
| M00133 | V$TST1_01 | 0.913874 | 549 | 467 | (-) | NNKGAWTWANANTNN | GAATGTTAAATGGCC |
| M00248 | V$OCT1_07 | 0.841213 | 548 | 468 | (+) | TNTATGNTAATT | TGAATGTTAAAT |
| M00042 | V$SOX5_01 | 0.80465 | 548 | 468 | (-) | NNAACAATNN | TGAATGTTAA |
| M00160 | V$SRY_02 | 0.840869 | 547 | 469 | (-) | NWWAACAAWANN | CTGAATGTTAAA |
| M00209 | V$NFY_C | 0.801901 | 546 | 470 | (+) | NCTGATTGGYTASY | TCTGAATGTTAAAT |
| M00223 | V$STAT_01 | 0.853542 | 543 | 473 | (+) | TTCCCRKAA | TTCTCTGAA |
| M00223 | V$STAT_01 | 0.833627 | 543 | 473 | (-) | TTCCCRKAA | TTCTCTGAA |
| M00162 | V$OCT1_06 | 0.800391 | 542 | 474 | (+) | CWNAWTKWSATRYN | ATTCTCTGAATGTT |
| M00076 | V$GATA2_01 | 0.805142 | 538 | 478 | (+) | NNNGATRNNN | AAAGATTCTC |
| M00147 | V$HSF2_01 | 0.801848 | 537 | 479 | (+) | NGAANNWTCK | GAAAGATTCT |
| M00148 | V$SRY_01 | 0.960465 | 534 | 482 | (+) | AAACWAM | AAAGAAA |
| M00162 | V$OCT1_06 | 0.808203 | 532 | 484 | (-) | CWNAWTKWSATRYN | AAAAAGAAAGATTC |
| M00109 | V$CEBPB_01 | 0.820456 | 530 | 486 | (+) | RNRTKNNGMAAKNN | AAAAAAAGAAAGAT |
| M00160 | V$SRY_02 | 0.828538 | 527 | 489 | (+) | NWWAACAAWANN | TTTAAAAAAAGA |
| M00252 | V$TATA_01 | 0.827201 | 526 | 490 | (+) | STATAAAWRNNNNNN | TTTTAAAAAAAGAAA |
| M00216 | V$TATA_C | 0.890151 | 525 | 491 | (+) | NCTATAAAAR | TTTTTAAAAA |
| M00026 | V$RSRFC4_01 | 0.821117 | 525 | 491 | (-) | RNKCTATTTWTAGMWN | TTTTTAAAAAAAGAAA |
| M00116 | V$CEBPA_01 | 0.805632 | 525 | 491 | (+) | NNATTRCNNAANNN | TTTTTAAAAAAAGA |
| M00232 | V$MEF2_03 | 0.825948 | 521 | 495 | (+) | NNNNNWKCTAWAAATAGMNNNN | TTTTTTTTTAAAAAAAGAAAGA |
| M00116 | V$CEBPA_01 | 0.818928 | 521 | 495 | (-) | NNATTRCNNAANNN | TTTTTTTTTAAAAA |
| M00269 | V$XFD3_01 | 0.815503 | 521 | 495 | (-) | WNWGTMAACAWWMW | TTTTTTTTTAAAAA |
| M00160 | V$SRY_02 | 0.810922 | 521 | 495 | (-) | NWWAACAAWANN | TTTTTTTTTAAA |
| M00231 | V$MEF2_02 | 0.80542 | 521 | 495 | (+) | NNNNNNKCTAWAAATAGMNNNN | TTTTTTTTTAAAAAAAGAAAGA |
| M00160 | V$SRY_02 | 0.81973 | 520 | 496 | (-) | NWWAACAAWANN | TTTTTTTTTTAA |
| M00160 | V$SRY_02 | 0.814445 | 519 | 497 | (-) | NWWAACAAWANN | TTTTTTTTTTTA |
| M00160 | V$SRY_02 | 0.810041 | 518 | 498 | (-) | NWWAACAAWANN | TTTTTTTTTTTT |
| M00160 | V$SRY_02 | 0.810041 | 517 | 499 | (-) | NWWAACAAWANN | TTTTTTTTTTTT |
| M00160 | V$SRY_02 | 0.810041 | 516 | 500 | (-) | NWWAACAAWANN | TTTTTTTTTTTT |
| M00130 | V$HFH2_01 | 0.896574 | 515 | 501 | (+) | NAWTGTTTRTTT | ATTTTTTTTTTT |
| M00081 | V$EVI1_04 | 0.910502 | 514 | 502 | (-) | NGATANGANWAGATA | TATTTTTTTTTTTTT |
| M00130 | V$HFH2_01 | 0.906017 | 514 | 502 | (+) | NAWTGTTTRTTT | TATTTTTTTTTT |
| M00129 | V$HFH1_01 | 0.806662 | 514 | 502 | (+) | NAWTGTTTATWT | TATTTTTTTTTT |
| M00130 | V$HFH2_01 | 0.895037 | 513 | 503 | (+) | NAWTGTTTRTTT | ATATTTTTTTTT |
| M00042 | V$SOX5_01 | 0.812399 | 513 | 503 | (-) | NNAACAATNN | ATATTTTTTT |
| M00130 | V$HFH2_01 | 0.958278 | 511 | 505 | (+) | NAWTGTTTRTTT | AAATATTTTTTT |
| M00131 | V$HNF3B_01 | 0.875516 | 511 | 505 | (+) | NNNTRTTTRYTY | AAATATTTTTTT |
| M00267 | V$XFD1_01 | 0.854882 | 510 | 506 | (-) | YAWGTAAAYAWWRY | AAAATATTTTTTTT |
| M00100 | V$CDXA_01 | 0.929868 | 507 | 509 | (-) | MTTTATR | CATAAAA |
| M00216 | V$TATA_C | 0.902033 | 505 | 511 | (+) | NCTATAAAAR | AGCATAAAAT |
| M00162 | V$OCT1_06 | 0.862891 | 505 | 511 | (-) | CWNAWTKWSATRYN | AGCATAAAATATTT |
| M00162 | V$OCT1_06 | 0.829297 | 504 | 512 | (+) | CWNAWTKWSATRYN | GAGCATAAAATATT |
| M00072 | V$CP2_01 | 0.900943 | 497 | 519 | (-) | GCNMNAMCMAG | CTGAGTTGAGC |
| M00175 | V$AP4_Q5 | 0.81012 | 497 | 519 | (-) | NNCAGCTGNN | CTGAGTTGAG |
| M00037 | V$NFE2_01 | 0.819706 | 495 | 521 | (+) | TGCTGASTCAY | AGCTGAGTTGA |
| M00253 | V$CAP_01 | 0.900936 | 493 | 523 | (+) | NCANNNNN | TCAGCTGA |
| M00175 | V$AP4_Q5 | 0.960283 | 492 | 524 | (+) | NNCAGCTGNN | CTCAGCTGAG |
| M00175 | V$AP4_Q5 | 0.960283 | 492 | 524 | (-) | NNCAGCTGNN | CTCAGCTGAG |
| M00176 | V$AP4_Q6 | 0.87998 | 492 | 524 | (+) | CWCAGCTGGN | CTCAGCTGAG |
| M00176 | V$AP4_Q6 | 0.87998 | 492 | 524 | (-) | CWCAGCTGGN | CTCAGCTGAG |
| M00073 | V$DELTAEF1_01 | 0.838532 | 492 | 524 | (-) | NNNCACCTNAN | CTCAGCTGAGT |
| M00073 | V$DELTAEF1_01 | 0.812865 | 491 | 525 | (+) | NNNCACCTNAN | GCTCAGCTGAG |
| M00220 | V$SREBP1_01 | 0.80005 | 491 | 525 | (+) | NATCACGTGAY | GCTCAGCTGAG |
| M00122 | V$USF_02 | 0.83064 | 490 | 526 | (+) | NNRNCACGTGNYNN | TGCTCAGCTGAGTT |
| M00122 | V$USF_02 | 0.83064 | 490 | 526 | (-) | NNRNCACGTGNYNN | TGCTCAGCTGAGTT |
| M00148 | V$SRY_01 | 0.924031 | 481 | 535 | (-) | AAACWAM | TAAGTTT |
| M00162 | V$OCT1_06 | 0.803125 | 481 | 535 | (-) | CWNAWTKWSATRYN | TAAGTTTCATGCTC |
| M00162 | V$OCT1_06 | 0.88125 | 480 | 536 | (+) | CWNAWTKWSATRYN | ATAAGTTTCATGCT |
| M00161 | V$OCT1_05 | 0.860203 | 480 | 536 | (+) | MKNATTTGCATAYY | ATAAGTTTCATGCT |
| M00210 | V$OCT_C | 0.817851 | 480 | 536 | (+) | CTNATTTGCATAY | ATAAGTTTCATGC |
| M00203 | V$GATA_C | 0.923268 | 478 | 538 | (+) | NGATAAGNMNN | AGATAAGTTTC |
| M00272 | V$P53_02 | 0.847597 | 478 | 538 | (+) | NGRCWTGYCY | AGATAAGTTT |
| M00082 | V$EVI1_05 | 0.84517 | 478 | 538 | (+) | AGATAAGATAN | AGATAAGTTTC |
| M00272 | V$P53_02 | 0.81272 | 478 | 538 | (-) | NGRCWTGYCY | AGATAAGTTT |
| M00077 | V$GATA3_01 | 0.887461 | 477 | 539 | (+) | NNGATARNG | AAGATAAGT |
| M00278 | V$LMO2COM_02 | 0.872978 | 477 | 539 | (+) | NMGATANSG | AAGATAAGT |
| M00076 | V$GATA2_01 | 0.894903 | 476 | 540 | (+) | NNNGATRNNN | CAAGATAAGT |
| M00075 | V$GATA1_01 | 0.874136 | 476 | 540 | (+) | SNNGATNNNN | CAAGATAAGT |
| M00128 | V$GATA1_04 | 0.892463 | 475 | 541 | (+) | NNCWGATARNNNN | TCAAGATAAGTTT |
| M00011 | V$EVI1_06 | 0.88781 | 475 | 541 | (+) | ACAAGATAA | TCAAGATAA |
| M00127 | V$GATA1_03 | 0.895884 | 474 | 542 | (+) | RNSNNGATAANNGN | ATCAAGATAAGTTT |
| M00126 | V$GATA1_02 | 0.869375 | 474 | 542 | (+) | NNNNNGATANKGNN | ATCAAGATAAGTTT |
| M00160 | V$SRY_02 | 0.805931 | 471 | 545 | (+) | NWWAACAAWANN | ATTATCAAGATA |
| M00077 | V$GATA3_01 | 0.850687 | 470 | 546 | (-) | NNGATARNG | TATTATCAA |
| M00278 | V$LMO2COM_02 | 0.833895 | 470 | 546 | (-) | NMGATANSG | TATTATCAA |
| M00075 | V$GATA1_01 | 0.810958 | 470 | 546 | (-) | SNNGATNNNN | TATTATCAAG |
| M00241 | V$NKX25_02 | 0.882601 | 469 | 547 | (-) | CWTAATTG | TTATTATC |
| M00042 | V$SOX5_01 | 0.804004 | 469 | 547 | (-) | NNAACAATNN | TTATTATCAA |
| M00127 | V$GATA1_03 | 0.897354 | 468 | 548 | (-) | RNSNNGATAANNGN | TTTATTATCAAGAT |
| M00128 | V$GATA1_04 | 0.833333 | 468 | 548 | (-) | NNCWGATARNNNN | TTTATTATCAAGA |
| M00126 | V$GATA1_02 | 0.833125 | 468 | 548 | (-) | NNNNNGATANKGNN | TTTATTATCAAGAT |
| M00203 | V$GATA_C | 0.890028 | 467 | 549 | (-) | NGATAAGNMNN | ATTTATTATCA |
| M00082 | V$EVI1_05 | 0.806782 | 467 | 549 | (-) | AGATAAGATAN | ATTTATTATCA |
| M00137 | V$OCT1_03 | 0.893323 | 466 | 550 | (-) | NNNRTAATNANNN | TATTTATTATCAA |
| M00103 | V$CLOX_01 | 0.820972 | 464 | 552 | (+) | NNTATCGATTANYNW | GTTATTTATTATCAA |
| M00102 | V$CDP_02 | 0.819651 | 464 | 552 | (+) | NWNATCGATTANYNN | GTTATTTATTATCAA |
| M00132 | V$HNF1_01 | 0.918591 | 463 | 553 | (+) | GGTTAATNWTTAMMN | AGTTATTTATTATCA |
| M00206 | V$HNF1_C | 0.900029 | 463 | 553 | (+) | NGTTAATKAWTNACCAM | AGTTATTTATTATCAAG |
| M00268 | V$XFD2_01 | 0.9208 | 462 | 554 | (-) | WNWATAAACAWNNR | TAGTTATTTATTAT |
| M00269 | V$XFD3_01 | 0.823921 | 462 | 554 | (-) | WNWGTMAACAWWMW | TAGTTATTTATTAT |
| M00206 | V$HNF1_C | 0.809983 | 461 | 555 | (-) | NGTTAATKAWTNACCAM | ATAGTTATTTATTATCA |
| M00098 | V$PAX2_01 | 0.827964 | 460 | 556 | (+) | NNNNGTCANGNRTKANNNN | AATAGTTATTTATTATCAA |
| M00099 | V$S8_01 | 0.807838 | 460 | 556 | (+) | WNNANYYAATTANYNN | AATAGTTATTTATTAT |
| M00131 | V$HNF3B_01 | 0.854404 | 459 | 557 | (+) | NNNTRTTTRYTY | CAATAGTTATTT |
| M00105 | V$CDPCR3_01 | 0.816673 | 459 | 557 | (-) | CACCRATANNTATNG | CAATAGTTATTTATT |
| M00253 | V$CAP_01 | 0.879744 | 458 | 558 | (+) | NCANNNNN | TCAATAGT |
| M00268 | V$XFD2_01 | 0.8548 | 458 | 558 | (-) | WNWATAAACAWNNR | TCAATAGTTATTTA |
| M00160 | V$SRY_02 | 0.847915 | 458 | 558 | (-) | NWWAACAAWANN | TCAATAGTTATT |
| M00138 | V$OCT1_04 | 0.81995 | 458 | 558 | (-) | NNNNNNNWATGCAAATNNNWNNW | TCAATAGTTATTTATTATCAAGA |
| M00254 | V$CAAT_01 | 0.804575 | 458 | 558 | (-) | NNNRRCCAATSA | TCAATAGTTATT |
| M00131 | V$HNF3B_01 | 0.88959 | 455 | 561 | (-) | NNNTRTTTRYTY | ATGTCAATAGTT |
| M00269 | V$XFD3_01 | 0.823571 | 454 | 562 | (+) | WNWGTMAACAWWMW | AATGTCAATAGTTA |
| M00160 | V$SRY_02 | 0.820023 | 454 | 562 | (+) | NWWAACAAWANN | AATGTCAATAGT |
| M00041 | V$CREBP1CJUN_01 | 0.843889 | 453 | 563 | (-) | TGACGTYA | AAATGTCA |
| M00162 | V$OCT1_06 | 0.868359 | 452 | 564 | (-) | CWNAWTKWSATRYN | AAAATGTCAATAGT |
| M00148 | V$SRY_01 | 0.904264 | 448 | 568 | (+) | AAACWAM | TATCAAA |
| M00059 | V$YY1_01 | 0.810288 | 447 | 569 | (-) | NNNNNCCATNTWNNNWN | ATATCAAAATGTCAATA |
| M00162 | V$OCT1_06 | 0.929688 | 446 | 570 | (-) | CWNAWTKWSATRYN | AATATCAAAATGTC |
| M00160 | V$SRY_02 | 0.858485 | 446 | 570 | (+) | NWWAACAAWANN | AATATCAAAATG |
| M00109 | V$CEBPB_01 | 0.8368 | 445 | 571 | (-) | RNRTKNNGMAAKNN | AAATATCAAAATGT |
| M00075 | V$GATA1_01 | 0.804047 | 445 | 571 | (-) | SNNGATNNNN | AAATATCAAA |
| M00076 | V$GATA2_01 | 0.801534 | 445 | 571 | (-) | NNNGATRNNN | AAATATCAAA |
| M00162 | V$OCT1_06 | 0.868359 | 444 | 572 | (-) | CWNAWTKWSATRYN | TAAATATCAAAATG |
| M00135 | V$OCT1_01 | 0.801221 | 443 | 573 | (+) | NNNNWTATGCAAATNTNNN | ATAAATATCAAAATGTCAA |
| M00138 | V$OCT1_04 | 0.827687 | 441 | 575 | (+) | NNNNNNNWATGCAAATNNNWNNW | TAATAAATATCAAAATGTCAATA |
| M00129 | V$HFH1_01 | 0.807295 | 441 | 575 | (-) | NAWTGTTTATWT | TAATAAATATCA |
| M00268 | V$XFD2_01 | 0.9076 | 440 | 576 | (+) | WNWATAAACAWNNR | CTAATAAATATCAA |
| M00206 | V$HNF1_C | 0.875144 | 438 | 578 | (+) | NGTTAATKAWTNACCAM | TGCTAATAAATATCAAA |
| M00223 | V$STAT_01 | 0.806655 | 438 | 578 | (-) | TTCCCRKAA | TGCTAATAA |
| M00137 | V$OCT1_03 | 0.857369 | 437 | 579 | (+) | NNNRTAATNANNN | TTGCTAATAAATA |
| M00103 | V$CLOX_01 | 0.815819 | 437 | 579 | (-) | NNTATCGATTANYNW | TTGCTAATAAATATC |
| M00102 | V$CDP_02 | 0.813755 | 437 | 579 | (-) | NWNATCGATTANYNN | TTGCTAATAAATATC |
| M00206 | V$HNF1_C | 0.830984 | 436 | 580 | (-) | NGTTAATKAWTNACCAM | CTTGCTAATAAATATCA |
| M00272 | V$P53_02 | 0.828253 | 433 | 583 | (+) | NGRCWTGYCY | TGCCTTGCTA |
| M00116 | V$CEBPA_01 | 0.827923 | 433 | 583 | (-) | NNATTRCNNAANNN | TGCCTTGCTAATAA |
| M00272 | V$P53_02 | 0.867233 | 428 | 588 | (+) | NGRCWTGYCY | AAATTTGCCT |
| M00057 | V$COMP1_01 | 0.802515 | 428 | 588 | (-) | NNTNWKGATTGRCNRSRANMRRNN | AAATTTGCCTTGCTAATAAATATC |
| M00195 | V$OCT1_Q6 | 0.803221 | 427 | 589 | (-) | NNNNATGCAAATNAN | GAAATTTGCCTTGCT |
| M00158 | V$COUP_01 | 0.842361 | 426 | 590 | (+) | TGAMCTTTGMMCYT | TGAAATTTGCCTTG |
| M00133 | V$TST1_01 | 0.908807 | 425 | 591 | (+) | NNKGAWTWANANTNN | GTGAAATTTGCCTTG |
| M00050 | V$E2F_02 | 0.876701 | 423 | 593 | (-) | TTTSGCGC | GCGTGAAA |
| M00134 | V$HNF4_01 | 0.819419 | 423 | 593 | (-) | NNNRGGNCAAAGKTCANNN | GCGTGAAATTTGCCTTGCT |
| M00138 | V$OCT1_04 | 0.813258 | 423 | 593 | (-) | NNNNNNNWATGCAAATNNNWNNW | GCGTGAAATTTGCCTTGCTAATA |
| M00249 | V$CHOP_01 | 0.805738 | 423 | 593 | (+) | NNRTGCAATMCCC | GCGTGAAATTTGC |
| M00180 | V$E2F_Q6 | 0.861209 | 421 | 595 | (+) | NNGCGCGAAANTK | GAGCGTGAAATTT |
| M00162 | V$OCT1_06 | 0.803516 | 421 | 595 | (+) | CWNAWTKWSATRYN | GAGCGTGAAATTTG |
| M00235 | V$AHRARNT_01 | 0.856967 | 415 | 601 | (+) | KNNKNNTYGCGTGCMS | AGAGCTGAGCGTGAAA |
| M00175 | V$AP4_Q5 | 0.964635 | 414 | 602 | (-) | NNCAGCTGNN | AAGAGCTGAG |
| M00176 | V$AP4_Q6 | 0.939364 | 414 | 602 | (-) | CWCAGCTGGN | AAGAGCTGAG |
| M00175 | V$AP4_Q5 | 0.81284 | 414 | 602 | (+) | NNCAGCTGNN | AAGAGCTGAG |
| M00001 | V$MYOD_01 | 0.827165 | 413 | 603 | (-) | SRACAGGTGKYG | AAAGAGCTGAGC |
| M00005 | V$AP4_01 | 0.858069 | 409 | 607 | (-) | WGARYCAGCTGYGGNCNK | CTGAAAAGAGCTGAGCGT |
| M00128 | V$GATA1_04 | 0.829044 | 407 | 609 | (+) | NNCWGATARNNNN | AGCTGAAAAGAGC |
| M00127 | V$GATA1_03 | 0.811367 | 406 | 610 | (+) | RNSNNGATAANNGN | CAGCTGAAAAGAGC |
| M00176 | V$AP4_Q6 | 0.87998 | 404 | 612 | (-) | CWCAGCTGGN | TCCAGCTGAA |
| M00175 | V$AP4_Q5 | 0.868063 | 404 | 612 | (-) | NNCAGCTGNN | TCCAGCTGAA |
| M00073 | V$DELTAEF1_01 | 0.817414 | 404 | 612 | (-) | NNNCACCTNAN | TCCAGCTGAAA |
| M00184 | V$MYOD_Q6 | 0.806292 | 404 | 612 | (+) | NNCANCTGNY | TCCAGCTGAA |
| M00277 | V$LMO2COM_01 | 0.825841 | 403 | 613 | (-) | SNNCAGGTGNNN | TTCCAGCTGAAA |
| M00073 | V$DELTAEF1_01 | 0.825211 | 403 | 613 | (+) | NNNCACCTNAN | TTCCAGCTGAA |
| M00050 | V$E2F_02 | 0.828381 | 402 | 614 | (+) | TTTSGCGC | TTTCCAGC |
| M00032 | V$CETS1P54_01 | 0.843835 | 400 | 616 | (-) | NCMGGAWGYN | TTTTTCCAGC |
| M00074 | V$CETS1P54_02 | 0.857003 | 399 | 617 | (-) | NNAMMGGAWRWNN | ATTTTTCCAGCTG |
| M00180 | V$E2F_Q6 | 0.819721 | 399 | 617 | (-) | NNGCGCGAAANTK | ATTTTTCCAGCTG |
| M00025 | V$ELK1_02 | 0.807469 | 399 | 617 | (-) | NNNNCCGGAARYNN | ATTTTTCCAGCTGA |
| M00024 | V$E2F_01 | 0.870958 | 398 | 618 | (-) | TWSGCGCGAAAAYKR | TATTTTTCCAGCTGA |
| M00109 | V$CEBPB_01 | 0.819217 | 397 | 619 | (-) | RNRTKNNGMAAKNN | ATATTTTTCCAGCT |
| M00148 | V$SRY_01 | 0.904264 | 393 | 623 | (-) | AAACWAM | TTTGATA |
| M00075 | V$GATA1_01 | 0.804047 | 393 | 623 | (+) | SNNGATNNNN | TTTGATATTT |
| M00076 | V$GATA2_01 | 0.801534 | 393 | 623 | (+) | NNNGATRNNN | TTTGATATTT |
| M00160 | V$SRY_02 | 0.844099 | 390 | 626 | (-) | NWWAACAAWANN | AATTTTGATATT |
| M00162 | V$OCT1_06 | 0.842187 | 390 | 626 | (+) | CWNAWTKWSATRYN | AATTTTGATATTTT |
| M00109 | V$CEBPB_01 | 0.822684 | 389 | 627 | (+) | RNRTKNNGMAAKNN | TAATTTTGATATTT |
| M00162 | V$OCT1_06 | 0.934766 | 388 | 628 | (+) | CWNAWTKWSATRYN | ATAATTTTGATATT |
| M00161 | V$OCT1_05 | 0.897393 | 388 | 628 | (+) | MKNATTTGCATAYY | ATAATTTTGATATT |
| M00195 | V$OCT1_Q6 | 0.830513 | 388 | 628 | (-) | NNNNATGCAAATNAN | ATAATTTTGATATTT |
| M00210 | V$OCT_C | 0.83017 | 388 | 628 | (+) | CTNATTTGCATAY | ATAATTTTGATAT |
| M00137 | V$OCT1_03 | 0.870012 | 385 | 631 | (+) | NNNRTAATNANNN | CTAATAATTTTGA |
| M00042 | V$SOX5_01 | 0.855021 | 385 | 631 | (+) | NNAACAATNN | CTAATAATTT |
| M00138 | V$OCT1_04 | 0.845044 | 384 | 632 | (-) | NNNNNNNWATGCAAATNNNWNNW | TCTAATAATTTTGATATTTTTCC |
| M00223 | V$STAT_01 | 0.868414 | 383 | 633 | (-) | TTCCCRKAA | TTCTAATAA |
| M00223 | V$STAT_01 | 0.826569 | 383 | 633 | (+) | TTCCCRKAA | TTCTAATAA |
| M00137 | V$OCT1_03 | 0.862505 | 382 | 634 | (+) | NNNRTAATNANNN | ATTCTAATAATTT |
| M00162 | V$OCT1_06 | 0.828906 | 380 | 636 | (-) | CWNAWTKWSATRYN | AAATTCTAATAATT |
| M00162 | V$OCT1_06 | 0.819141 | 379 | 637 | (-) | CWNAWTKWSATRYN | AAAATTCTAATAAT |
| M00195 | V$OCT1_Q6 | 0.800491 | 378 | 638 | (+) | NNNNATGCAAATNAN | CAAAATTCTAATAAT |
| M00136 | V$OCT1_02 | 0.811586 | 376 | 640 | (+) | NNGAATATKCANNNN | ATCAAAATTCTAATA |
| M00148 | V$SRY_01 | 0.904264 | 375 | 641 | (+) | AAACWAM | TATCAAA |
| M00138 | V$OCT1_04 | 0.809076 | 374 | 642 | (+) | NNNNNNNWATGCAAATNNNWNNW | ATATCAAAATTCTAATAATTTTG |
| M00162 | V$OCT1_06 | 0.921875 | 373 | 643 | (-) | CWNAWTKWSATRYN | AATATCAAAATTCT |
| M00161 | V$OCT1_05 | 0.892744 | 373 | 643 | (-) | MKNATTTGCATAYY | AATATCAAAATTCT |
| M00160 | V$SRY_02 | 0.844099 | 373 | 643 | (+) | NWWAACAAWANN | AATATCAAAATT |
| M00109 | V$CEBPB_01 | 0.845963 | 372 | 644 | (-) | RNRTKNNGMAAKNN | AAATATCAAAATTC |
| M00075 | V$GATA1_01 | 0.804047 | 372 | 644 | (-) | SNNGATNNNN | AAATATCAAA |
| M00195 | V$OCT1_Q6 | 0.804039 | 372 | 644 | (+) | NNNNATGCAAATNAN | AAATATCAAAATTCT |
| M00076 | V$GATA2_01 | 0.801534 | 372 | 644 | (-) | NNNGATRNNN | AAATATCAAA |
| M00162 | V$OCT1_06 | 0.842187 | 371 | 645 | (-) | CWNAWTKWSATRYN | TAAATATCAAAATT |
| M00203 | V$GATA_C | 0.833178 | 369 | 647 | (-) | NGATAAGNMNN | AGTAAATATCA |
| M00201 | V$CEBP_C | 0.833078 | 369 | 647 | (-) | NGWNTKNKGYAAKNSAYA | AGTAAATATCAAAATTCT |
| M00138 | V$OCT1_04 | 0.843371 | 368 | 648 | (+) | NNNNNNNWATGCAAATNNNWNNW | TAGTAAATATCAAAATTCTAATA |
| M00268 | V$XFD2_01 | 0.8764 | 367 | 649 | (+) | WNWATAAACAWNNR | CTAGTAAATATCAA |
| M00269 | V$XFD3_01 | 0.849526 | 367 | 649 | (+) | WNWGTMAACAWWMW | CTAGTAAATATCAA |
| M00223 | V$STAT_01 | 0.850517 | 365 | 651 | (-) | TTCCCRKAA | TGCTAGTAA |
| M00206 | V$HNF1_C | 0.843642 | 365 | 651 | (+) | NGTTAATKAWTNACCAM | TGCTAGTAAATATCAAA |
| M00129 | V$HFH1_01 | 0.816783 | 353 | 663 | (+) | NAWTGTTTATWT | TAAAATTTATTC |
| M00100 | V$CDXA_01 | 0.929868 | 351 | 665 | (-) | MTTTATR | CATAAAA |
| M00216 | V$TATA_C | 0.902033 | 349 | 667 | (+) | NCTATAAAAR | AGCATAAAAT |
| M00162 | V$OCT1_06 | 0.88125 | 349 | 667 | (-) | CWNAWTKWSATRYN | AGCATAAAATTTAT |
| M00162 | V$OCT1_06 | 0.816406 | 348 | 668 | (+) | CWNAWTKWSATRYN | GAGCATAAAATTTA |
| M00240 | V$NKX25_01 | 0.852443 | 343 | 673 | (-) | TYAAGTG | CAGTTGA |
| M00253 | V$CAP_01 | 0.923115 | 342 | 674 | (+) | NCANNNNN | TCAGTTGA |
| M00227 | V$VMYB_02 | 0.884477 | 342 | 674 | (-) | NSYAACGGN | TCAGTTGAG |
| M00183 | V$MYB_Q6 | 0.90249 | 341 | 675 | (-) | NNNAACKGNC | CTCAGTTGAG |
| M00175 | V$AP4_Q5 | 0.834603 | 341 | 675 | (+) | NNCAGCTGNN | CTCAGTTGAG |
| M00175 | V$AP4_Q5 | 0.834603 | 341 | 675 | (-) | NNCAGCTGNN | CTCAGTTGAG |
| M00073 | V$DELTAEF1_01 | 0.812865 | 341 | 675 | (-) | NNNCACCTNAN | CTCAGTTGAGC |
| M00072 | V$CP2_01 | 0.810463 | 341 | 675 | (-) | GCNMNAMCMAG | CTCAGTTGAGC |
| M00097 | V$PAX6_01 | 0.814929 | 340 | 676 | (-) | NNNNTTCACGCWTSANTKNNN | ACTCAGTTGAGCATAAAATTT |
| M00175 | V$AP4_Q5 | 0.81012 | 336 | 680 | (+) | NNCAGCTGNN | CTCAACTCAG |
| M00072 | V$CP2_01 | 0.805746 | 335 | 681 | (+) | GCNMNAMCMAG | TCTCAACTCAG |
| M00253 | V$CAP_01 | 0.976836 | 331 | 685 | (+) | NCANNNNN | TCATTCTC |
| M00148 | V$SRY_01 | 0.923256 | 329 | 687 | (-) | AAACWAM | TTTCATT |
| M00162 | V$OCT1_06 | 0.847266 | 324 | 692 | (+) | CWNAWTKWSATRYN | TTTTTTTTCATTCT |
| M00269 | V$XFD3_01 | 0.803578 | 322 | 694 | (-) | WNWGTMAACAWWMW | TTTTTTTTTTCATT |
| M00160 | V$SRY_02 | 0.824427 | 321 | 695 | (-) | NWWAACAAWANN | TTTTTTTTTTTC |
| M00138 | V$OCT1_04 | 0.851527 | 320 | 696 | (-) | NNNNNNNWATGCAAATNNNWNNW | TTTTTTTTTTTTCATTCTCAACT |
| M00160 | V$SRY_02 | 0.810041 | 320 | 696 | (-) | NWWAACAAWANN | TTTTTTTTTTTT |
| M00081 | V$EVI1_04 | 0.904166 | 319 | 697 | (-) | NGATANGANWAGATA | TTTTTTTTTTTTTCA |
| M00160 | V$SRY_02 | 0.810041 | 319 | 697 | (-) | NWWAACAAWANN | TTTTTTTTTTTT |
| M00081 | V$EVI1_04 | 0.893395 | 318 | 698 | (-) | NGATANGANWAGATA | TTTTTTTTTTTTTTC |
| M00160 | V$SRY_02 | 0.810041 | 318 | 698 | (-) | NWWAACAAWANN | TTTTTTTTTTTT |
| M00081 | V$EVI1_04 | 0.893395 | 317 | 699 | (-) | NGATANGANWAGATA | TTTTTTTTTTTTTTT |
| M00160 | V$SRY_02 | 0.810041 | 317 | 699 | (-) | NWWAACAAWANN | TTTTTTTTTTTT |
| M00081 | V$EVI1_04 | 0.893395 | 316 | 700 | (-) | NGATANGANWAGATA | TTTTTTTTTTTTTTT |
| M00160 | V$SRY_02 | 0.810041 | 316 | 700 | (-) | NWWAACAAWANN | TTTTTTTTTTTT |
| M00081 | V$EVI1_04 | 0.893395 | 315 | 701 | (-) | NGATANGANWAGATA | TTTTTTTTTTTTTTT |
| M00160 | V$SRY_02 | 0.810041 | 315 | 701 | (-) | NWWAACAAWANN | TTTTTTTTTTTT |
| M00081 | V$EVI1_04 | 0.893395 | 314 | 702 | (-) | NGATANGANWAGATA | TTTTTTTTTTTTTTT |
| M00160 | V$SRY_02 | 0.810041 | 314 | 702 | (-) | NWWAACAAWANN | TTTTTTTTTTTT |
| M00081 | V$EVI1_04 | 0.893395 | 313 | 703 | (-) | NGATANGANWAGATA | TTTTTTTTTTTTTTT |
| M00160 | V$SRY_02 | 0.810041 | 313 | 703 | (-) | NWWAACAAWANN | TTTTTTTTTTTT |
| M00081 | V$EVI1_04 | 0.893395 | 312 | 704 | (-) | NGATANGANWAGATA | TTTTTTTTTTTTTTT |
| M00160 | V$SRY_02 | 0.810041 | 312 | 704 | (-) | NWWAACAAWANN | TTTTTTTTTTTT |
| M00081 | V$EVI1_04 | 0.893395 | 311 | 705 | (-) | NGATANGANWAGATA | TTTTTTTTTTTTTTT |
| M00160 | V$SRY_02 | 0.810041 | 311 | 705 | (-) | NWWAACAAWANN | TTTTTTTTTTTT |
| M00081 | V$EVI1_04 | 0.893395 | 310 | 706 | (-) | NGATANGANWAGATA | TTTTTTTTTTTTTTT |
| M00160 | V$SRY_02 | 0.810041 | 310 | 706 | (-) | NWWAACAAWANN | TTTTTTTTTTTT |
| M00160 | V$SRY_02 | 0.807105 | 309 | 707 | (-) | NWWAACAAWANN | CTTTTTTTTTTT |
| M00160 | V$SRY_02 | 0.827657 | 308 | 708 | (-) | NWWAACAAWANN | TCTTTTTTTTTT |
| M00147 | V$HSF2_01 | 0.886876 | 301 | 715 | (+) | NGAANNWTCK | AGAAAACTCT |
| M00147 | V$HSF2_01 | 0.884288 | 301 | 715 | (-) | NGAANNWTCK | AGAAAACTCT |
| M00146 | V$HSF1_01 | 0.853486 | 301 | 715 | (+) | RGAANRTTCN | AGAAAACTCT |
| M00146 | V$HSF1_01 | 0.832217 | 301 | 715 | (-) | RGAANRTTCN | AGAAAACTCT |
| M00260 | V$HLF_01 | 0.845244 | 298 | 718 | (+) | RTTACRYAAT | GTTAGAAAAC |
| M00117 | V$CEBPB_02 | 0.85891 | 296 | 720 | (+) | NKNTTGCNYAAYNN | ACGTTAGAAAACTC |
| M00116 | V$CEBPA_01 | 0.82675 | 296 | 720 | (+) | NNATTRCNNAANNN | ACGTTAGAAAACTC |
| M00227 | V$VMYB_02 | 0.861011 | 295 | 721 | (-) | NSYAACGGN | GACGTTAGA |
| M00041 | V$CREBP1CJUN_01 | 0.905833 | 294 | 722 | (+) | TGACGTYA | CGACGTTA |
| M00039 | V$CREB_01 | 0.825197 | 294 | 722 | (-) | TGACGTMA | CGACGTTA |
| M00178 | V$CREB_Q4 | 0.817769 | 292 | 724 | (-) | NSTGACGTMANN | CCCGACGTTAGA |
| M00177 | V$CREB_Q2 | 0.805509 | 292 | 724 | (-) | NSTGACGTAANN | CCCGACGTTAGA |
| M00178 | V$CREB_Q4 | 0.80313 | 292 | 724 | (+) | NSTGACGTMANN | CCCGACGTTAGA |
| M00017 | V$ATF_01 | 0.815106 | 291 | 725 | (-) | CNSTGACGTNNNYC | GCCCGACGTTAGAA |
| M00076 | V$GATA2_01 | 0.813261 | 284 | 732 | (+) | NNNGATRNNN | GATCATGGCC |
| M00075 | V$GATA1_01 | 0.858835 | 281 | 735 | (+) | SNNGATNNNN | GCTGATCATG |
| M00076 | V$GATA2_01 | 0.839423 | 281 | 735 | (+) | NNNGATRNNN | GCTGATCATG |
| M00075 | V$GATA1_01 | 0.828727 | 281 | 735 | (-) | SNNGATNNNN | GCTGATCATG |
| M00076 | V$GATA2_01 | 0.815516 | 281 | 735 | (-) | NNNGATRNNN | GCTGATCATG |
| M00127 | V$GATA1_03 | 0.804508 | 279 | 737 | (+) | RNSNNGATAANNGN | CAGCTGATCATGGC |
| M00175 | V$AP4_Q5 | 0.978237 | 277 | 739 | (-) | NNCAGCTGNN | AACAGCTGAT |
| M00175 | V$AP4_Q5 | 0.949402 | 277 | 739 | (+) | NNCAGCTGNN | AACAGCTGAT |
| M00176 | V$AP4_Q6 | 0.946379 | 277 | 739 | (-) | CWCAGCTGGN | AACAGCTGAT |
| M00184 | V$MYOD_Q6 | 0.910549 | 277 | 739 | (-) | NNCANCTGNY | AACAGCTGAT |
| M00184 | V$MYOD_Q6 | 0.909315 | 277 | 739 | (+) | NNCANCTGNY | AACAGCTGAT |
| M00176 | V$AP4_Q6 | 0.869957 | 277 | 739 | (+) | CWCAGCTGGN | AACAGCTGAT |
| M00220 | V$SREBP1_01 | 0.834867 | 277 | 739 | (-) | NATCACGTGAY | AACAGCTGATC |
| M00001 | V$MYOD_01 | 0.952362 | 276 | 740 | (-) | SRACAGGTGKYG | CAACAGCTGATC |
| M00277 | V$LMO2COM_01 | 0.883445 | 276 | 740 | (-) | SNNCAGGTGNNN | CAACAGCTGATC |
| M00001 | V$MYOD_01 | 0.819094 | 276 | 740 | (+) | SRACAGGTGKYG | CAACAGCTGATC |
| M00277 | V$LMO2COM_01 | 0.812382 | 276 | 740 | (+) | SNNCAGGTGNNN | CAACAGCTGATC |
| M00220 | V$SREBP1_01 | 0.811738 | 276 | 740 | (+) | NATCACGTGAY | CAACAGCTGAT |
| M00122 | V$USF_02 | 0.867309 | 275 | 741 | (+) | NNRNCACGTGNYNN | TCAACAGCTGATCA |
| M00122 | V$USF_02 | 0.867309 | 275 | 741 | (-) | NNRNCACGTGNYNN | TCAACAGCTGATCA |
| M00071 | V$E47_02 | 0.871477 | 274 | 742 | (-) | NNNMRCAGGTGTTMNN | GTCAACAGCTGATCAT |
| M00183 | V$MYB_Q6 | 0.867635 | 274 | 742 | (+) | NNNAACKGNC | GTCAACAGCT |
| M00066 | V$TAL1ALPHAE47_01 | 0.838622 | 274 | 742 | (+) | NNNAACAGATGKTNNN | GTCAACAGCTGATCAT |
| M00066 | V$TAL1ALPHAE47_01 | 0.831057 | 274 | 742 | (-) | NNNAACAGATGKTNNN | GTCAACAGCTGATCAT |
| M00071 | V$E47_02 | 0.81646 | 274 | 742 | (+) | NNNMRCAGGTGTTMNN | GTCAACAGCTGATCAT |
| M00065 | V$TAL1BETAE47_01 | 0.811994 | 274 | 742 | (-) | NNNAACAGATGKTNNN | GTCAACAGCTGATCAT |
| M00070 | V$TAL1BETAITF2_01 | 0.811179 | 274 | 742 | (+) | NNNAACAGATGKTNNN | GTCAACAGCTGATCAT |
| M00227 | V$VMYB_02 | 0.801143 | 274 | 742 | (+) | NSYAACGGN | GTCAACAGC |
| M00005 | V$AP4_01 | 0.89438 | 272 | 744 | (-) | WGARYCAGCTGYGGNCNK | ATGTCAACAGCTGATCAT |
| M00269 | V$XFD3_01 | 0.83269 | 271 | 745 | (+) | WNWGTMAACAWWMW | TATGTCAACAGCTG |
| M00040 | V$CREBP1_01 | 0.895531 | 270 | 746 | (-) | TTACGTAA | TTATGTCA |
| M00039 | V$CREB_01 | 0.857644 | 270 | 746 | (-) | TGACGTMA | TTATGTCA |
| M00109 | V$CEBPB_01 | 0.844973 | 270 | 746 | (-) | RNRTKNNGMAAKNN | TTATGTCAACAGCT |
| M00113 | V$CREB_02 | 0.839337 | 270 | 746 | (-) | NNGNTGACGYNN | TTATGTCAACAG |
| M00040 | V$CREBP1_01 | 0.827529 | 270 | 746 | (+) | TTACGTAA | TTATGTCA |
| M00041 | V$CREBP1CJUN_01 | 0.814444 | 270 | 746 | (-) | TGACGTYA | TTATGTCA |
| M00155 | V$ARP1_01 | 0.800785 | 270 | 746 | (-) | TGARCCYTTGAMCCYW | TTATGTCAACAGCTGA |
| M00228 | V$VBP_01 | 0.863787 | 269 | 747 | (+) | GTTACRTMAK | CTTATGTCAA |
| M00178 | V$CREB_Q4 | 0.863958 | 268 | 748 | (-) | NSTGACGTMANN | CCTTATGTCAAC |
| M00177 | V$CREB_Q2 | 0.842716 | 268 | 748 | (-) | NSTGACGTAANN | CCTTATGTCAAC |
| M00179 | V$CREBP1_Q2 | 0.8426 | 268 | 748 | (-) | NSTGACGTMASN | CCTTATGTCAAC |
| M00160 | V$SRY_02 | 0.816207 | 268 | 748 | (-) | NWWAACAAWANN | CCTTATGTCAAC |
| M00278 | V$LMO2COM_02 | 0.813679 | 268 | 748 | (-) | NMGATANSG | CCTTATGTC |
| M00087 | V$IK2_01 | 0.86322 | 263 | 753 | (-) | NNNYGGGAWNNN | GAGTTCCTTATG |
| M00032 | V$CETS1P54_01 | 0.828897 | 263 | 753 | (-) | NCMGGAWGYN | GAGTTCCTTA |
| M00074 | V$CETS1P54_02 | 0.868783 | 262 | 754 | (-) | NNAMMGGAWRWNN | TGAGTTCCTTATG |
| M00025 | V$ELK1_02 | 0.81053 | 262 | 754 | (-) | NNNNCCGGAARYNN | TGAGTTCCTTATGT |
| M00037 | V$NFE2_01 | 0.832126 | 259 | 757 | (+) | TGCTGASTCAY | AAATGAGTTCC |
| M00162 | V$OCT1_06 | 0.825 | 257 | 759 | (+) | CWNAWTKWSATRYN | CAAAATGAGTTCCT |
| M00205 | V$GRE_C | 0.802372 | 255 | 761 | (+) | GGTACAANNTGTYCTK | TGCAAAATGAGTTCCT |
| M00059 | V$YY1_01 | 0.838467 | 253 | 763 | (-) | NNNNNCCATNTWNNNWN | CATGCAAAATGAGTTCC |
| M00145 | V$BRN2_01 | 0.988886 | 251 | 765 | (+) | NNCATNSRWAATNMRN | TTCATGCAAAATGAGT |
| M00248 | V$OCT1_07 | 0.819165 | 251 | 765 | (+) | TNTATGNTAATT | TTCATGCAAAAT |
| M00117 | V$CEBPB_02 | 0.866807 | 249 | 767 | (-) | NKNTTGCNYAAYNN | CATTCATGCAAAAT |
| M00190 | V$CEBP_Q2 | 0.851675 | 249 | 767 | (-) | NNNTTGCNNAANNN | CATTCATGCAAAAT |
| M00106 | V$CDPCR3HD_01 | 0.841984 | 249 | 767 | (+) | NATYGATSSS | CATTCATGCA |
| M00253 | V$CAP_01 | 0.903894 | 248 | 768 | (+) | NCANNNNN | GCATTCAT |
| M00098 | V$PAX2_01 | 0.846106 | 248 | 768 | (-) | NNNNGTCANGNRTKANNNN | GCATTCATGCAAAATGAGT |
| M00097 | V$PAX6_01 | 0.803904 | 246 | 770 | (-) | NNNNTTCACGCWTSANTKNNN | GCGCATTCATGCAAAATGAGT |
| M00147 | V$HSF2_01 | 0.809612 | 245 | 771 | (-) | NGAANNWTCK | CGCGCATTCA |
| M00203 | V$GATA_C | 0.86269 | 230 | 786 | (+) | NGATAAGNMNN | AGATATGTGTC |
| M00001 | V$MYOD_01 | 0.840354 | 230 | 786 | (+) | SRACAGGTGKYG | AGATATGTGTCT |
| M00278 | V$LMO2COM_02 | 0.834569 | 229 | 787 | (+) | NMGATANSG | TAGATATGT |
| M00077 | V$GATA3_01 | 0.831192 | 229 | 787 | (+) | NNGATARNG | TAGATATGT |
| M00075 | V$GATA1_01 | 0.851925 | 228 | 788 | (+) | SNNGATNNNN | GTAGATATGT |
| M00076 | V$GATA2_01 | 0.833559 | 228 | 788 | (+) | NNNGATRNNN | GTAGATATGT |
| M00128 | V$GATA1_04 | 0.842831 | 227 | 789 | (+) | NNCWGATARNNNN | TGTAGATATGTGT |
| M00126 | V$GATA1_02 | 0.864688 | 226 | 790 | (+) | NNNNNGATANKGNN | GTGTAGATATGTGT |
| M00127 | V$GATA1_03 | 0.821656 | 226 | 790 | (+) | RNSNNGATAANNGN | GTGTAGATATGTGT |
| M00109 | V$CEBPB_01 | 0.84101 | 224 | 792 | (+) | RNRTKNNGMAAKNN | ATGTGTAGATATGT |
| M00129 | V$HFH1_01 | 0.801392 | 222 | 794 | (+) | NAWTGTTTATWT | AGATGTGTAGAT |
| M00253 | V$CAP_01 | 0.931493 | 220 | 796 | (+) | NCANNNNN | TCAGATGT |
| M00076 | V$GATA2_01 | 0.880469 | 220 | 796 | (+) | NNNGATRNNN | TCAGATGTGT |
| M00075 | V$GATA1_01 | 0.852419 | 220 | 796 | (+) | SNNGATNNNN | TCAGATGTGT |
| M00217 | V$USF_C | 0.814156 | 220 | 796 | (-) | NCACGTGN | TCAGATGT |
| M00184 | V$MYOD_Q6 | 0.899753 | 219 | 797 | (-) | NNCANCTGNY | GTCAGATGTG |
| M00175 | V$AP4_Q5 | 0.841676 | 219 | 797 | (+) | NNCAGCTGNN | GTCAGATGTG |
| M00176 | V$AP4_Q6 | 0.813831 | 219 | 797 | (+) | CWCAGCTGGN | GTCAGATGTG |
| M00175 | V$AP4_Q5 | 0.805767 | 219 | 797 | (-) | NNCAGCTGNN | GTCAGATGTG |
| M00277 | V$LMO2COM_01 | 0.847376 | 218 | 798 | (+) | SNNCAGGTGNNN | CGTCAGATGTGT |
| M00001 | V$MYOD_01 | 0.83937 | 218 | 798 | (-) | SRACAGGTGKYG | CGTCAGATGTGT |
| M00122 | V$USF_02 | 0.827533 | 217 | 799 | (+) | NNRNCACGTGNYNN | TCGTCAGATGTGTA |
| M00122 | V$USF_02 | 0.827533 | 217 | 799 | (-) | NNRNCACGTGNYNN | TCGTCAGATGTGTA |
| M00199 | V$AP1_C | 0.813994 | 215 | 801 | (+) | NTGASTCAG | GTTCGTCAG |
| M00172 | V$AP1FJ_Q2 | 0.839117 | 214 | 802 | (-) | RSTGACTNMNW | TGTTCGTCAGA |
| M00174 | V$AP1_Q6 | 0.827755 | 214 | 802 | (-) | NNTGACTCANN | TGTTCGTCAGA |
| M00188 | V$AP1_Q4 | 0.821897 | 214 | 802 | (-) | RSTGACTMANN | TGTTCGTCAGA |
| M00177 | V$CREB_Q2 | 0.845132 | 213 | 803 | (-) | NSTGACGTAANN | GTGTTCGTCAGA |
| M00178 | V$CREB_Q4 | 0.829631 | 213 | 803 | (-) | NSTGACGTMANN | GTGTTCGTCAGA |
| M00017 | V$ATF_01 | 0.842906 | 212 | 804 | (-) | CNSTGACGTNNNYC | CGTGTTCGTCAGAT |
| M00272 | V$P53_02 | 0.863423 | 209 | 807 | (+) | NGRCWTGYCY | TAACGTGTTC |
| M00217 | V$USF_C | 0.817175 | 209 | 807 | (+) | NCACGTGN | TAACGTGT |
| M00217 | V$USF_C | 0.801744 | 209 | 807 | (-) | NCACGTGN | TAACGTGT |
| M00123 | V$MYCMAX_02 | 0.841608 | 207 | 809 | (-) | NANCACGTGNNW | TTTAACGTGTTC |
| M00183 | V$MYB_Q6 | 0.841079 | 207 | 809 | (+) | NNNAACKGNC | TTTAACGTGT |
| M00236 | V$ARNT_01 | 0.823475 | 205 | 811 | (+) | NNNNNCACGTGNNNNN | ACTTTAACGTGTTCGT |
| M00162 | V$OCT1_06 | 0.839844 | 202 | 814 | (+) | CWNAWTKWSATRYN | CTAACTTTAACGTG |
| M00227 | V$VMYB_02 | 0.802948 | 201 | 815 | (+) | NSYAACGGN | TCTAACTTT |
| M00101 | V$CDXA_02 | 0.981893 | 198 | 818 | (+) | WWTWMTR | ATTTCTA |
| M00100 | V$CDXA_01 | 0.919568 | 198 | 818 | (+) | MTTTATR | ATTTCTA |
| M00109 | V$CEBPB_01 | 0.841258 | 196 | 820 | (-) | RNRTKNNGMAAKNN | CGATTTCTAACTTT |
| M00076 | V$GATA2_01 | 0.853857 | 194 | 822 | (+) | NNNGATRNNN | TGCGATTTCT |
| M00075 | V$GATA1_01 | 0.839092 | 194 | 822 | (+) | SNNGATNNNN | TGCGATTTCT |
| M00175 | V$AP4_Q5 | 0.906964 | 186 | 830 | (-) | NNCAGCTGNN | CGCTGCTGTG |
| M00176 | V$AP4_Q6 | 0.846154 | 186 | 830 | (-) | CWCAGCTGGN | CGCTGCTGTG |
| M00175 | V$AP4_Q5 | 0.841676 | 186 | 830 | (+) | NNCAGCTGNN | CGCTGCTGTG |
| M00176 | V$AP4_Q6 | 0.823854 | 186 | 830 | (+) | CWCAGCTGGN | CGCTGCTGTG |
| M00002 | V$E47_01 | 0.824628 | 184 | 832 | (+) | NSNGCAGGTGKNCNN | GGCGCTGCTGTGCGA |
| M00175 | V$AP4_Q5 | 0.853645 | 183 | 833 | (-) | NNCAGCTGNN | GGGCGCTGCT |
| M00176 | V$AP4_Q6 | 0.845152 | 183 | 833 | (-) | CWCAGCTGGN | GGGCGCTGCT |
| M00008 | V$SP1_01 | 0.849377 | 182 | 834 | (+) | GRGGCRGGGW | TGGGCGCTGC |
| M00050 | V$E2F_02 | 0.871702 | 181 | 835 | (+) | TTTSGCGC | TTGGGCGC |
| M00255 | V$GC_01 | 0.860118 | 180 | 836 | (+) | NRGGGGCGGGGCNK | TTTGGGCGCTGCTG |
| M00189 | V$AP2_Q6 | 0.810662 | 176 | 840 | (-) | MKCCCSCNGGCG | TGTATTTGGGCG |
| M00186 | V$SRF_Q6 | 0.842561 | 173 | 843 | (-) | GNCCAWATAWGGMN | GCCTGTATTTGGGC |
| M00272 | V$P53_02 | 0.829426 | 171 | 845 | (-) | NGRCWTGYCY | GGGCCTGTAT |
| M00189 | V$AP2_Q6 | 0.801241 | 165 | 851 | (+) | MKCCCSCNGGCG | GTTCCAGGGCCT |
| M00032 | V$CETS1P54_01 | 0.838403 | 163 | 853 | (-) | NCMGGAWGYN | GGGTTCCAGG |
| M00085 | V$ZID_01 | 0.81843 | 163 | 853 | (+) | NGGCTCYATCAYC | GGGTTCCAGGGCC |
| M00211 | V$PADS_C | 0.838206 | 160 | 856 | (+) | NGTGGTCTC | TGTGGGTTC |
| M00053 | V$CREL_01 | 0.830645 | 160 | 856 | (+) | SGGRNWTTCC | TGTGGGTTCC |
| M00253 | V$CAP_01 | 0.874322 | 156 | 860 | (+) | NCANNNNN | GCATTGTG |
| M00187 | V$USF_Q6 | 0.814929 | 156 | 860 | (-) | GYCACGTGNC | GCATTGTGGG |
| M00042 | V$SOX5_01 | 0.803358 | 156 | 860 | (-) | NNAACAATNN | GCATTGTGGG |
| M00057 | V$COMP1_01 | 0.801557 | 151 | 865 | (+) | NNTNWKGATTGRCNRSRANMRRNN | ACCCTGCATTGTGGGTTCCAGGGC |
| M00008 | V$SP1_01 | 0.801246 | 151 | 865 | (-) | GRGGCRGGGW | ACCCTGCATT |
| M00255 | V$GC_01 | 0.813572 | 149 | 867 | (-) | NRGGGGCGGGGCNK | AAACCCTGCATTGT |
| M00158 | V$COUP_01 | 0.835343 | 148 | 868 | (+) | TGAMCTTTGMMCYT | TAAACCCTGCATTG |
| M00008 | V$SP1_01 | 0.804077 | 146 | 870 | (-) | GRGGCRGGGW | ACTAAACCCT |
| M00223 | V$STAT_01 | 0.826821 | 138 | 878 | (-) | TTCCCRKAA | TTTCAAGAA |
| M00148 | V$SRY_01 | 0.960465 | 134 | 882 | (-) | AAACWAM | TTTCTTT |
| M00109 | V$CEBPB_01 | 0.837543 | 124 | 892 | (-) | RNRTKNNGMAAKNN | TGATTACTTCTTTC |
| M00113 | V$CREB_02 | 0.822771 | 123 | 893 | (+) | NNGNTGACGYNN | GTGATTACTTCT |
| M00188 | V$AP1_Q4 | 0.870082 | 122 | 894 | (+) | RSTGACTMANN | AGTGATTACTT |
| M00173 | V$AP1_Q2 | 0.868228 | 122 | 894 | (+) | RSTGACTNMNW | AGTGATTACTT |
| M00172 | V$AP1FJ_Q2 | 0.852681 | 122 | 894 | (+) | RSTGACTNMNW | AGTGATTACTT |
| M00075 | V$GATA1_01 | 0.818361 | 122 | 894 | (+) | SNNGATNNNN | AGTGATTACT |
| M00174 | V$AP1_Q6 | 0.807446 | 122 | 894 | (+) | NNTGACTCANN | AGTGATTACTT |
| M00137 | V$OCT1_03 | 0.869617 | 121 | 895 | (-) | NNNRTAATNANNN | CAGTGATTACTTC |
| M00253 | V$CAP_01 | 0.912765 | 120 | 896 | (+) | NCANNNNN | TCAGTGAT |
| M00159 | V$CEBP_01 | 0.870989 | 120 | 896 | (+) | NNTKTGGWNANNN | TCAGTGATTACTT |
| M00185 | V$NFY_Q6 | 0.812179 | 119 | 897 | (-) | TRRCCAATSRN | CTCAGTGATTA |
| M00037 | V$NFE2_01 | 0.811219 | 117 | 899 | (+) | TGCTGASTCAY | TGCTCAGTGAT |
| M00035 | V$VMAF_01 | 0.800981 | 114 | 902 | (-) | NNNTGCTGACTCAGCANNN | GTTTGCTCAGTGATTACTT |
| M00159 | V$CEBP_01 | 0.905673 | 113 | 903 | (+) | NNTKTGGWNANNN | TGTTTGCTCAGTG |
| M00190 | V$CEBP_Q2 | 0.839529 | 113 | 903 | (+) | NNNTTGCNNAANNN | TGTTTGCTCAGTGA |
| M00116 | V$CEBPA_01 | 0.818146 | 113 | 903 | (+) | NNATTRCNNAANNN | TGTTTGCTCAGTGA |
| M00131 | V$HNF3B_01 | 0.979859 | 110 | 906 | (+) | NNNTRTTTRYTY | AAATGTTTGCTC |
| M00130 | V$HFH2_01 | 0.942907 | 110 | 906 | (+) | NAWTGTTTRTTT | AAATGTTTGCTC |
| M00129 | V$HFH1_01 | 0.830698 | 110 | 906 | (+) | NAWTGTTTATWT | AAATGTTTGCTC |
| M00269 | V$XFD3_01 | 0.862855 | 109 | 907 | (-) | WNWGTMAACAWWMW | CAAATGTTTGCTCA |
| M00042 | V$SOX5_01 | 0.802067 | 109 | 907 | (-) | NNAACAATNN | CAAATGTTTG |
| M00160 | V$SRY_02 | 0.86054 | 108 | 908 | (-) | NWWAACAAWANN | CCAAATGTTTGC |
| M00141 | V$LYF1_01 | 0.87631 | 104 | 912 | (-) | TTTGGGAGR | ATTCCCAAA |
| M00253 | V$CAP_01 | 0.962543 | 102 | 914 | (+) | NCANNNNN | TCATTCCC |
| M00087 | V$IK2_01 | 0.959424 | 102 | 914 | (-) | NNNYGGGAWNNN | TCATTCCCAAAT |
| M00159 | V$CEBP_01 | 0.875203 | 102 | 914 | (-) | NNTKTGGWNANNN | TCATTCCCAAATG |
| M00109 | V$CEBPB_01 | 0.838782 | 102 | 914 | (-) | RNRTKNNGMAAKNN | TCATTCCCAAATGT |
| M00116 | V$CEBPA_01 | 0.829488 | 102 | 914 | (+) | NNATTRCNNAANNN | TCATTCCCAAATGT |
| M00086 | V$IK1_01 | 0.921741 | 101 | 915 | (-) | NNNTGGGAATRCC | ATCATTCCCAAAT |
| M00088 | V$IK3_01 | 0.862265 | 101 | 915 | (-) | TNYTGGGAATACC | ATCATTCCCAAAT |
| M00253 | V$CAP_01 | 0.936422 | 99 | 917 | (+) | NCANNNNN | TCATCATT |
| M00255 | V$GC_01 | 0.808917 | 99 | 917 | (-) | NRGGGGCGGGGCNK | TCATCATTCCCAAA |
| M00075 | V$GATA1_01 | 0.813425 | 97 | 919 | (-) | SNNGATNNNN | AATCATCATT |
| M00076 | V$GATA2_01 | 0.807397 | 97 | 919 | (-) | NNNGATRNNN | AATCATCATT |
| M00075 | V$GATA1_01 | 0.870188 | 94 | 922 | (-) | SNNGATNNNN | GCAAATCATC |
| M00076 | V$GATA2_01 | 0.835814 | 94 | 922 | (-) | NNNGATRNNN | GCAAATCATC |
| M00185 | V$NFY_Q6 | 0.820658 | 92 | 924 | (+) | TRRCCAATSRN | CAGCAAATCAT |
| M00254 | V$CAAT_01 | 0.858797 | 90 | 926 | (+) | NNNRRCCAATSA | ATCAGCAAATCA |
| M00175 | V$AP4_Q5 | 0.801143 | 90 | 926 | (+) | NNCAGCTGNN | ATCAGCAAAT |
| M00253 | V$CAP_01 | 0.908822 | 88 | 928 | (+) | NCANNNNN | TCATCAGC |
| M00159 | V$CEBP_01 | 0.880389 | 88 | 928 | (-) | NNTKTGGWNANNN | TCATCAGCAAATC |
| M00075 | V$GATA1_01 | 0.884008 | 86 | 930 | (-) | SNNGATNNNN | GATCATCAGC |
| M00076 | V$GATA2_01 | 0.858818 | 86 | 930 | (-) | NNNGATRNNN | GATCATCAGC |
| M00076 | V$GATA2_01 | 0.837167 | 83 | 933 | (-) | NNNGATRNNN | AGTGATCATC |
| M00075 | V$GATA1_01 | 0.826752 | 83 | 933 | (-) | SNNGATNNNN | AGTGATCATC |
| M00253 | V$CAP_01 | 0.912765 | 81 | 935 | (+) | NCANNNNN | TCAGTGAT |
| M00127 | V$GATA1_03 | 0.802058 | 81 | 935 | (-) | RNSNNGATAANNGN | TCAGTGATCATCAG |
| M00101 | V$CDXA_02 | 0.995062 | 73 | 943 | (-) | WWTWMTR | TATTATT |
| M00042 | V$SOX5_01 | 0.876978 | 72 | 944 | (-) | NNAACAATNN | GTATTATTTT |
| M00160 | V$SRY_02 | 0.81973 | 71 | 945 | (-) | NWWAACAAWANN | AGTATTATTTTC |
| M00240 | V$NKX25_01 | 0.894602 | 68 | 948 | (+) | TYAAGTG | GCAAGTA |
| M00131 | V$HNF3B_01 | 0.907547 | 66 | 950 | (-) | NNNTRTTTRYTY | AAGCAAGTATTA |
| M00272 | V$P53_02 | 0.887749 | 66 | 950 | (-) | NGRCWTGYCY | AAGCAAGTAT |
| M00162 | V$OCT1_06 | 0.821484 | 63 | 953 | (-) | CWNAWTKWSATRYN | CGTAAGCAAGTATT |
| M00032 | V$CETS1P54_01 | 0.84465 | 61 | 955 | (+) | NCMGGAWGYN | TCCGTAAGCA |
| M00227 | V$VMYB_02 | 0.837545 | 61 | 955 | (-) | NSYAACGGN | TCCGTAAGC |
| M00035 | V$VMAF_01 | 0.856559 | 55 | 961 | (-) | NNNTGCTGACTCAGCANNN | TTTTGCTCCGTAAGCAAGT |
| M00072 | V$CP2_01 | 0.848199 | 51 | 965 | (-) | GCNMNAMCMAG | CTGGTTTTGCT |
| M00254 | V$CAAT_01 | 0.850042 | 48 | 968 | (-) | NNNRRCCAATSA | TTACTGGTTTTG |
| M00185 | V$NFY_Q6 | 0.810637 | 47 | 969 | (-) | TRRCCAATSRN | TTTACTGGTTT |
| M00131 | V$HNF3B_01 | 0.873574 | 46 | 970 | (+) | NNNTRTTTRYTY | CTTTACTGGTTT |
| M00148 | V$SRY_01 | 0.927132 | 36 | 980 | (+) | AAACWAM | AAACTCC |
| M00008 | V$SP1_01 | 0.803228 | 36 | 980 | (-) | GRGGCRGGGW | AAACTCCCTT |
| M00053 | V$CREL_01 | 0.91129 | 34 | 982 | (-) | SGGRNWTTCC | GAAAACTCCC |
| M00052 | V$NFKAPPAB65_01 | 0.861557 | 34 | 982 | (-) | GGGRATTTCC | GAAAACTCCC |
| M00054 | V$NFKAPPAB_01 | 0.850687 | 34 | 982 | (-) | GGGAMTTYCC | GAAAACTCCC |
| M00183 | V$MYB_Q6 | 0.837344 | 34 | 982 | (+) | NNNAACKGNC | GAAAACTCCC |
| M00033 | V$P300_01 | 0.859577 | 33 | 983 | (-) | NNNRGGAGTNNNNS | TGAAAACTCCCTTC |
| M00053 | V$CREL_01 | 0.836763 | 33 | 983 | (-) | SGGRNWTTCC | TGAAAACTCC |
| M00147 | V$HSF2_01 | 0.827726 | 33 | 983 | (-) | NGAANNWTCK | TGAAAACTCC |
| M00255 | V$GC_01 | 0.826311 | 33 | 983 | (-) | NRGGGGCGGGGCNK | TGAAAACTCCCTTC |
| M00147 | V$HSF2_01 | 0.819593 | 33 | 983 | (+) | NGAANNWTCK | TGAAAACTCC |
| M00208 | V$NFKB_C | 0.808787 | 33 | 983 | (-) | NGGGACTTTCCA | TGAAAACTCCCT |
| M00148 | V$SRY_01 | 0.923256 | 31 | 985 | (+) | AAACWAM | AATGAAA |
| M00212 | V$POLY_C | 0.860469 | 31 | 985 | (+) | CAATAAAACCYYYYKCTN | AATGAAAACTCCCTTCTT |
| M00194 | V$NFKB_Q6 | 0.815713 | 31 | 985 | (+) | NGGGGAMTTTCCNN | AATGAAAACTCCCT |
| M00162 | V$OCT1_06 | 0.821484 | 29 | 987 | (-) | CWNAWTKWSATRYN | CAAATGAAAACTCC |
| M00240 | V$NKX25_01 | 0.852443 | 28 | 988 | (+) | TYAAGTG | TCAAATG |
| M00160 | V$SRY_02 | 0.832355 | 24 | 992 | (+) | NWWAACAAWANN | TTGATCAAATGA |
| M00087 | V$IK2_01 | 0.85144 | 17 | 999 | (-) | NNNYGGGAWNNN | TTATTCCTTGAT |
| M00240 | V$NKX25_01 | 0.88566 | 14 | 1002 | (-) | TYAAGTG | TACTTAT |
| M00083 | V$MZF1_01 | 0.897242 | 6 | 1010 | (+) | NGNGGGGA | AGTGGGGC |
| M00189 | V$AP2_Q6 | 0.826746 | 3 | 1013 | (-) | MKCCCSCNGGCG | TGGAGTGGGGCT |
| M00084 | V$MZF1_02 | 0.814546 | 3 | 1013 | (+) | KNNNKAGGGGNAA | TGGAGTGGGGCTA |
| M00255 | V$GC_01 | 0.83415 | 2 | 1014 | (+) | NRGGGGCGGGGCNK | ATGGAGTGGGGCTA |

**Table F.** TFBSs predicted for motif 1 of HbI Short variant promoter using JASPAR CORE database.

| Model ID | Model name | Score | Relative score | Start | End | Strand | predicted site sequence |
| --- | --- | --- | --- | --- | --- | --- | --- |
| MA0260.1 | che-1 | 9.637 | 0.999982482449056 | -196 | -191 | -1 | GAAACC |
| MA0185.1 | Deaf1 | 7.403 | 0.982137204900323 | -193 | -188 | 1 | TTCGTC |
| MA0109.1 | Hltf | 7.989 | 0.96278206962497 | -202 | -193 | -1 | AACCTTATTC |
| MA0254.1 | vvl | 7.065 | 0.942392193191509 | -179 | -174 | 1 | TATGAA |
| MA0398.1 | SUM1 | 9.894 | 0.91997445957927 | -176 | -168 | 1 | GAAATTTAT |
| MA0356.1 | PHO2 | 6.093 | 0.918923453467486 | -173 | -168 | -1 | ATAAAT |
| MA0292.1 | ECM22 | 8.136 | 0.911672914854768 | -189 | -183 | -1 | ATCCCGA |
| MA0393.1 | STE12 | 7.069 | 0.909974623624032 | -196 | -190 | -1 | CGAAACC |
| MA0362.1 | RDS2 | 8.359 | 0.906081559804815 | -190 | -184 | 1 | GTCGGGA |
| MA0388.1 | SPT23 | 7.982 | 0.899951749313451 | -176 | -169 | 1 | GAAATTTA |
| MA0275.1 | ASG1 | 6.095 | 0.882419306969049 | -189 | -184 | 1 | TCGGGA |
| MA0158.1 | HOXA5 | 6.437 | 0.879640272279523 | -174 | -167 | -1 | AATAAATT |
| MA0356.1 | PHO2 | 4.766 | 0.873518144938539 | -174 | -169 | -1 | TAAATT |
| MA0356.1 | PHO2 | 4.760 | 0.873312845879629 | -175 | -170 | -1 | AAATTT |
| MA0356.1 | PHO2 | 4.760 | 0.873312845879629 | -175 | -170 | 1 | AAATTT |
| MA0182.1 | CG4328 | 5.610 | 0.86622966505901 | -172 | -166 | 1 | TTTATTC |
| MA0158.1 | HOXA5 | 6.022 | 0.865024346918901 | -178 | -171 | 1 | ATGAAATT |
| MA0388.1 | SPT23 | 6.346 | 0.859442208981846 | -177 | -170 | -1 | AAATTTCA |
| MA0033.1 | FOXL1 | 5.542 | 0.856197009355974 | -179 | -172 | -1 | ATTTCATA |
| MA0174.1 | CG42234 | 6.075 | 0.854322467730258 | -172 | -166 | 1 | TTTATTC |
| MA0151.1 | ARID3A | 6.028 | 0.851117048557595 | -178 | -173 | 1 | ATGAAA |
| MA0165.1 | Abd-B | 6.181 | 0.848545474204444 | -172 | -166 | 1 | TTTATTC |
| MA0380.1 | SIP4 | 5.441 | 0.847772803569964 | -190 | -184 | -1 | TCCCGAC |
| MA0410.1 | UGA3 | 7.319 | 0.846625787368909 | -191 | -184 | 1 | CGTCGGGA |
| MA0468.1 | DUX4 | 3.411 | 0.84282852815686 | -175 | -165 | 1 | AAATTTATTCT |
| MA0466.1 | CEBPB | 2.021 | 0.841123760618751 | -181 | -171 | -1 | AATTTCATAGC |
| MA0448.1 | H2.0 | 5.198 | 0.834658174922453 | -172 | -166 | 1 | TTTATTC |
| MA0436.1 | YPR022C | 5.833 | 0.834618890706663 | -191 | -185 | -1 | CCCGACG |
| MA0298.1 | FZF1 | 5.610 | 0.833359139872015 | -180 | -175 | 1 | CTATGA |
| MA0187.1 | Dll | 5.575 | 0.832790376982396 | -176 | -170 | -1 | AAATTTC |
| MA0319.1 | HSF1 | 6.847 | 0.831767105026708 | -170 | -163 | -1 | GTAGAATA |
| MA0164.1 | Nr2e3 | 6.922 | 0.830568760825101 | -198 | -192 | -1 | AAACCTT |
| MA0027.1 | En1 | 5.145 | 0.830158373075178 | -176 | -166 | -1 | GAATAAATTTC |
| MA0135.1 | Lhx3 | 9.760 | 0.829635672366114 | -175 | -163 | 1 | AAATTTATTCTAC |
| MA0387.1 | SPT2 | 4.626 | 0.827742132704954 | -180 | -171 | -1 | AATTTCATAG |
| MA0102.3 | CEBPA | 2.413 | 0.826897387224214 | -182 | -172 | -1 | ATTTCATAGCG |
| MA0151.1 | ARID3A | 5.399 | 0.826577864606885 | -172 | -167 | -1 | AATAAA |
| MA0185.1 | Deaf1 | 4.440 | 0.825410241421729 | -190 | -185 | 1 | GTCGGG |
| MA0398.1 | SUM1 | 6.627 | 0.82413618730456 | -177 | -169 | -1 | TAAATTTCA |
| MA0151.1 | ARID3A | 5.307 | 0.822988667590247 | -173 | -168 | -1 | ATAAAT |
| MA0356.1 | PHO2 | 3.286 | 0.822877710407384 | -170 | -165 | -1 | AGAATA |
| MA0393.1 | STE12 | 1.956 | 0.822422751781893 | -177 | -171 | 1 | TGAAATT |
| MA0340.1 | MOT3 | 3.192 | 0.820385724489732 | -198 | -193 | 1 | AAGGTT |
| MA0349.1 | OPI1 | 4.972 | 0.819477255019577 | -187 | -181 | -1 | CGATCCC |
| MA0444.1 | CG34031 | 6.186 | 0.819106313034942 | -180 | -174 | -1 | TTCATAG |
| MA0275.1 | ASG1 | 4.148 | 0.818419728781981 | -188 | -183 | 1 | CGGGAT |
| MA0021.1 | Dof3 | 4.362 | 0.818348034268571 | -182 | -177 | -1 | ATAGCG |
| MA0356.1 | PHO2 | 3.096 | 0.816376573541898 | -202 | -197 | -1 | TTATTC |
| MA0356.1 | PHO2 | 3.096 | 0.816376573541898 | -171 | -166 | 1 | TTATTC |
| MA0158.1 | HOXA5 | 4.629 | 0.815964144443369 | -174 | -167 | 1 | AATTTATT |
| MA0484.1 | HNF4G | 5.111 | 0.815131817207171 | -177 | -163 | -1 | GTAGAATAAATTTCA |
| MA0546.1 | PHA-4 | 3.188 | 0.81471945975796 | -174 | -165 | -1 | AGAATAAATT |
| MA0148.3 | FOXA1 | 2.086 | 0.814546251516201 | -178 | -164 | 1 | ATGAAATTTATTCTA |
| MA0293.1 | ECM23 | 6.045 | 0.814543731734092 | -189 | -179 | -1 | AGCGATCCCGA |
| MA0132.1 | Pdx1 | 5.029 | 0.813134065294963 | -201 | -196 | -1 | CTTATT |
| MA0216.2 | CAD | 2.836 | 0.81299227912208 | -174 | -164 | -1 | TAGAATAAATT |
| MA0485.1 | Hoxc9 | 7.023 | 0.812954351226674 | -176 | -164 | -1 | TAGAATAAATTTC |
| MA0417.1 | YAP5 | 3.227 | 0.811170196687768 | -197 | -192 | -1 | AAACCT |
| MA0056.1 | MZF1_1-4 | 4.916 | 0.809768936774344 | -189 | -184 | 1 | TCGGGA |
| MA0301.1 | GAT3 | 5.641 | 0.809730655116885 | -189 | -181 | -1 | CGATCCCGA |
| MA0176.1 | CG15696 | 5.132 | 0.809122402918138 | -172 | -166 | 1 | TTTATTC |
| MA0403.1 | TBF1 | 5.725 | 0.807962914546301 | -199 | -192 | -1 | AAACCTTA |
| MA0465.1 | CDX2 | 3.630 | 0.807464054443621 | -173 | -163 | -1 | GTAGAATAAAT |
| MA0328.1 | MATALPHA2 | 7.737 | 0.806793818438332 | -179 | -171 | 1 | TATGAAATT |
| MA0008.1 | HAT5 | 6.670 | 0.806514742289694 | -174 | -167 | 1 | AATTTATT |
| MA0164.1 | Nr2e3 | 6.240 | 0.806206760727597 | -199 | -193 | 1 | TAAGGTT |
| MA0254.1 | vvl | 4.208 | 0.805890628194314 | -170 | -165 | 1 | TATTCT |
| MA0453.1 | nub | 8.815 | 0.805699732846537 | -179 | -168 | 1 | TATGAAATTTAT |
| MA0356.1 | PHO2 | 2.781 | 0.805598372949119 | -179 | -174 | -1 | TTCATA |
| MA0523.1 | TCF7L2 | 4.565 | 0.805525512905877 | -183 | -170 | -1 | AAATTTCATAGCGA |
| MA0457.1 | PHDP | 5.012 | 0.80502015477911 | -175 | -169 | -1 | TAAATTT |
| MA0206.1 | abd-A | 5.630 | 0.8043030186751 | -172 | -166 | 1 | TTTATTC |
| MA0318.1 | HMRA2 | 5.351 | 0.803777298810699 | -179 | -172 | 1 | TATGAAAT |
| MA0356.1 | PHO2 | 2.716 | 0.803374299810926 | -176 | -171 | 1 | GAAATT |
| MA0183.1 | CG7056 | 4.909 | 0.802792604163007 | -171 | -164 | -1 | TAGAATAA |
| MA0295.1 | FHL1 | 5.935 | 0.802584953726028 | -195 | -188 | -1 | GACGAAAC |
| MA0222.1 | exd | 5.777 | 0.802449539343465 | -179 | -172 | -1 | ATTTCATA |
| MA0448.1 | H2.0 | 4.294 | 0.800248972468284 | -180 | -174 | -1 | TTCATAG |
| MA0349.1 | OPI1 | 4.318 | 0.800085570174109 | -196 | -190 | -1 | CGAAACC |

**Table G.** TFBSs predicted for motif 1 of HbI Long variant promoter using JASPAR CORE database.

| Model ID | Model name | Score | Relative score | Start | End | Strand | predicted site sequence |
| --- | --- | --- | --- | --- | --- | --- | --- |
| MA0018.2 | CREB1 | 11.569 | 1.00001610713187 | -48 | -41 | -1 | TGACGTCA |
| MA0018.2 | CREB1 | 11.569 | 1.00001610713187 | -48 | -41 | 1 | TGACGTCA |
| MA0096.1 | bZIP910 | 11.694 | 0.999988573314069 | -49 | -43 | 1 | ATGACGT |
| MA0096.1 | bZIP910 | 11.694 | 0.999988573314069 | -46 | -40 | -1 | ATGACGT |
| MA0089.1 | NFE2L1::MafG | 8.812 | 0.999988528354811 | -61 | -56 | -1 | CATGAC |
| MA0089.1 | NFE2L1::MafG | 8.812 | 0.999988528354811 | -44 | -39 | -1 | CATGAC |
| MA0089.1 | NFE2L1::MafG | 8.692 | 0.994791423381168 | -50 | -45 | 1 | GATGAC |
| MA0286.1 | CST6 | 12.956 | 0.98250105765721 | -49 | -41 | 1 | ATGACGTCA |
| MA0286.1 | CST6 | 12.956 | 0.98250105765721 | -48 | -40 | -1 | ATGACGTCA |
| MA0588.1 | TGA1 | 12.166 | 0.969160664117876 | -51 | -41 | 1 | CGATGACGTCA |
| MA0492.1 | JUND (var.2) | 16.093 | 0.962114716964235 | -53 | -39 | 1 | GTCGATGACGTCATG |
| MA0067.1 | Pax2 | 8.055 | 0.960306655890571 | -45 | -38 | 1 | CGTCATGG |
| MA0488.1 | JUN | 15.656 | 0.958231009149488 | -52 | -40 | 1 | TCGATGACGTCAT |
| MA0071.1 | RORA_1 | 12.831 | 0.957639597140774 | -67 | -58 | 1 | AAAAAGGTCA |
| MA0526.1 | USF2 | 13.278 | 0.952514095736006 | -61 | -51 | 1 | GTCATGTGGTC |
| MA0093.2 | USF1 | 13.537 | 0.951046194556644 | -62 | -52 | -1 | ACCACATGACC |
| MA0492.1 | JUND (var.2) | 14.713 | 0.948040145007353 | -50 | -36 | -1 | ATCCATGACGTCATC |
| MA0588.1 | TGA1 | 11.277 | 0.945465041784856 | -48 | -38 | -1 | CCATGACGTCA |
| MA0104.3 | Mycn | 11.082 | 0.944124755105325 | -59 | -52 | -1 | ACCACATG |
| MA0488.1 | JUN | 13.331 | 0.932909206881452 | -49 | -37 | -1 | TCCATGACGTCAT |
| MA0514.1 | Sox3 | 11.078 | 0.92960417344514 | -72 | -63 | -1 | TTTTTGTCTT |
| MA0067.1 | Pax2 | 7.263 | 0.929205804296677 | -62 | -55 | 1 | GGTCATGT |
| MA0109.1 | Hltf | 7.018 | 0.928560462671827 | -68 | -59 | -1 | GACCTTTTTG |
| MA0512.1 | Rxra | 10.822 | 0.92020985041023 | -66 | -56 | 1 | AAAAGGTCATG |
| MA0093.2 | USF1 | 10.533 | 0.911558207594865 | -61 | -51 | 1 | GTCATGTGGTC |
| MA0515.1 | Sox6 | 9.905 | 0.903301441936724 | -72 | -63 | -1 | TTTTTGTCTT |
| MA0442.1 | SOX10 | 6.636 | 0.899054900725468 | -69 | -64 | -1 | TTTTGT |
| MA0284.1 | CIN5 | 10.479 | 0.897285712776773 | -50 | -41 | -1 | TGACGTCATC |
| MA0130.1 | ZNF354C | 6.331 | 0.886867302367815 | -56 | -51 | -1 | GACCAC |
| MA0271.1 | ARG80 | 5.319 | 0.885717846319175 | -48 | -43 | 1 | TGACGT |
| MA0271.1 | ARG80 | 5.319 | 0.885717846319175 | -46 | -41 | -1 | TGACGT |
| MA0129.1 | TGA1A | 7.503 | 0.885423204438983 | -48 | -42 | -1 | GACGTCA |
| MA0129.1 | TGA1A | 7.503 | 0.885423204438983 | -47 | -41 | 1 | GACGTCA |
| MA0160.1 | NR4A2 | 8.368 | 0.885207510186178 | -64 | -57 | 1 | AAGGTCAT |
| MA0227.1 | hth | 6.994 | 0.88390330432487 | -48 | -43 | 1 | TGACGT |
| MA0227.1 | hth | 6.994 | 0.88390330432487 | -46 | -41 | -1 | TGACGT |
| MA0147.2 | Myc | 7.098 | 0.878999764958928 | -60 | -51 | 1 | TCATGTGGTC |
| MA0058.2 | MAX | 7.164 | 0.875730890794475 | -60 | -51 | -1 | GACCACATGA |
| MA0064.1 | PBF | 5.740 | 0.875633761075617 | -65 | -61 | 1 | AAAGG |
| MA0417.1 | YAP5 | 5.289 | 0.873963050389523 | -71 | -66 | 1 | AGACAA |
| MA0382.1 | SKO1 | 7.931 | 0.873234157282879 | -46 | -39 | 1 | ACGTCATG |
| MA0043.1 | HLF | 9.423 | 0.871176571670281 | -50 | -39 | 1 | GATGACGTCATG |
| MA0562.1 | PIF5 | 7.598 | 0.87019111278048 | -60 | -53 | 1 | TCATGTGG |
| MA0128.1 | EmBP-1 | 8.544 | 0.869182779363801 | -60 | -53 | 1 | TCATGTGG |
| MA0020.1 | Dof2 | 6.086 | 0.8684839414205 | -65 | -60 | 1 | AAAGGT |
| MA0141.2 | Esrrb | 9.014 | 0.866719079680297 | -69 | -58 | 1 | ACAAAAAGGTCA |
| MA0043.1 | HLF | 9.161 | 0.864321310179483 | -50 | -39 | -1 | CATGACGTCATC |
| MA0464.1 | Bhlhe40 | 6.011 | 0.864312866793748 | -62 | -52 | -1 | ACCACATGACC |
| MA0084.1 | SRY | 7.594 | 0.863808502861034 | -73 | -65 | 1 | GAAGACAAA |
| MA0552.1 | PIL5 | 10.012 | 0.863529081774303 | -66 | -53 | 1 | AAAAGGTCATGTGG |
| MA0568.1 | MYC3 | 6.903 | 0.863227858990747 | -60 | -53 | -1 | CCACATGA |
| MA0568.1 | MYC3 | 6.903 | 0.863227858990747 | -60 | -53 | 1 | TCATGTGG |
| MA0526.1 | USF2 | 6.180 | 0.860295487235648 | -62 | -52 | -1 | ACCACATGACC |
| MA0246.1 | so | 6.547 | 0.859298323701265 | -39 | -34 | 1 | GGATAC |
| MA0117.1 | Mafb | 6.191 | 0.856725209874602 | -50 | -43 | 1 | GATGACGT |
| MA0233.1 | mirr | 4.685 | 0.856486885116523 | -71 | -67 | 1 | AGACA |
| MA0254.1 | vvl | 5.229 | 0.854671901509728 | -40 | -35 | -1 | TATCCA |
| MA0002.2 | RUNX1 | 8.007 | 0.854213965851758 | -60 | -50 | 1 | TCATGTGGTCG |
| MA0560.1 | PIF3 | 7.833 | 0.853294234947798 | -62 | -53 | 1 | GGTCATGTGG |
| MA0351.1 | DOT6 | 11.796 | 0.853096362272484 | -57 | -37 | -1 | TCCATGACGTCATCGACCACA |
| MA0304.1 | GCR1 | 5.603 | 0.853076782853488 | -52 | -45 | 1 | TCGATGAC |
| MA0096.1 | bZIP910 | 7.070 | 0.851897543410036 | -63 | -57 | -1 | ATGACCT |
| MA0037.2 | GATA3 | 5.228 | 0.851260838323882 | -71 | -64 | 1 | AGACAAAA |
| MA0078.1 | Sox17 | 7.046 | 0.850944979468093 | -70 | -62 | -1 | CTTTTTGTC |
| MA0514.1 | Sox3 | 5.749 | 0.85073307704525 | -70 | -61 | -1 | CCTTTTTGTC |
| MA0143.3 | Sox2 | 5.365 | 0.847424236334906 | -68 | -61 | -1 | CCTTTTTG |
| MA0247.2 | tin | 6.708 | 0.847183115678226 | -62 | -53 | 1 | GGTCATGTGG |
| MA0592.1 | ESRRA | 9.277 | 0.846267639029555 | -66 | -56 | 1 | AAAAGGTCATG |
| MA0334.1 | MET32 | 6.599 | 0.845417620773614 | -57 | -51 | -1 | GACCACA |
| MA0409.1 | TYE7 | 7.428 | 0.845399786701181 | -60 | -54 | -1 | CACATGA |
| MA0264.1 | ceh-22 | 8.566 | 0.845319789121125 | -62 | -52 | -1 | ACCACATGACC |
| MA0464.1 | Bhlhe40 | 4.277 | 0.840915864746505 | -61 | -51 | 1 | GTCATGTGGTC |
| MA0447.1 | gt | 8.670 | 0.838774187245393 | -49 | -40 | -1 | ATGACGTCAT |
| MA0447.1 | gt | 8.670 | 0.838774187245393 | -49 | -40 | 1 | ATGACGTCAT |
| MA0561.1 | PIF4 | 5.976 | 0.837370820276001 | -61 | -54 | -1 | CACATGAC |
| MA0288.1 | CUP9 | 6.624 | 0.837185216147445 | -49 | -41 | -1 | TGACGTCAT |
| MA0288.1 | CUP9 | 6.624 | 0.837185216147445 | -48 | -40 | 1 | TGACGTCAT |
| MA0350.1 | TOD6 | 10.634 | 0.836204428159958 | -57 | -37 | -1 | TCCATGACGTCATCGACCACA |
| MA0562.1 | PIF5 | 5.978 | 0.835724337269941 | -60 | -53 | -1 | CCACATGA |
| MA0086.1 | sna | 6.136 | 0.835301411980938 | -59 | -54 | 1 | CATGTG |
| MA0113.2 | NR3C1 | 6.918 | 0.834667229517744 | -72 | -58 | 1 | AAGACAAAAAGGTCA |
| MA0414.1 | XBP1 | 5.100 | 0.834343236562675 | -54 | -48 | -1 | ATCGACC |
| MA0332.1 | MET28 | 5.706 | 0.83334372474105 | -58 | -53 | 1 | ATGTGG |
| MA0004.1 | Arnt | 6.112 | 0.830868166588692 | -59 | -54 | -1 | CACATG |
| MA0004.1 | Arnt | 6.112 | 0.830868166588692 | -59 | -54 | 1 | CATGTG |
| MA0550.1 | BZR1 | 7.198 | 0.829639124848637 | -63 | -50 | -1 | CGACCACATGACCT |
| MA0284.1 | CIN5 | 7.691 | 0.829438036124969 | -48 | -39 | 1 | TGACGTCATG |
| MA0053.1 | MNB1A | 4.751 | 0.827847747021057 | -65 | -61 | 1 | AAAGG |
| MA0204.1 | Six4 | 5.353 | 0.825572433099554 | -39 | -34 | 1 | GGATAC |
| MA0129.1 | TGA1A | 5.912 | 0.825469589986974 | -64 | -58 | 1 | AAGGTCA |
| MA0130.1 | ZNF354C | 4.916 | 0.824934520817588 | -41 | -36 | -1 | ATCCAT |
| MA0595.1 | SREBF1 | 7.101 | 0.824808592882539 | -49 | -40 | -1 | ATGACGTCAT |
| MA0595.1 | SREBF1 | 7.101 | 0.824808592882539 | -49 | -40 | 1 | ATGACGTCAT |
| MA0560.1 | PIF3 | 6.382 | 0.824572293359049 | -60 | -51 | -1 | GACCACATGA |
| MA0357.1 | PHO4 | 4.052 | 0.824399970650606 | -60 | -53 | -1 | CCACATGA |
| MA0357.1 | PHO4 | 4.052 | 0.824399970650606 | -60 | -53 | 1 | TCATGTGG |
| MA0044.1 | HMG-1 | 5.346 | 0.823532862264614 | -73 | -65 | -1 | TTTGTCTTC |
| MA0260.1 | che-1 | 4.903 | 0.821901013771282 | -73 | -68 | 1 | GAAGAC |
| MA0408.1 | TOS8 | 6.365 | 0.821521477986254 | -50 | -43 | -1 | ACGTCATC |
| MA0408.1 | TOS8 | 6.365 | 0.821521477986254 | -46 | -39 | 1 | ACGTCATG |
| MA0130.1 | ZNF354C | 4.828 | 0.821082870798916 | -53 | -48 | -1 | ATCGAC |
| MA0340.1 | MOT3 | 3.192 | 0.820385724489732 | -72 | -67 | 1 | AAGACA |
| MA0340.1 | MOT3 | 3.192 | 0.820385724489732 | -64 | -59 | 1 | AAGGTC |
| MA0307.1 | GLN3 | 3.711 | 0.818238073549229 | -38 | -34 | 1 | GATAC |
| MA0059.1 | MYC::MAX | 8.687 | 0.818147120421481 | -62 | -52 | 1 | GGTCATGTGGT |
| MA0012.1 | br_Z3 | 6.832 | 0.818042020791186 | -72 | -62 | 1 | AAGACAAAAAG |
| MA0442.1 | SOX10 | 4.805 | 0.817767607423551 | -67 | -62 | -1 | CTTTTT |
| MA0596.1 | SREBF2 | 7.466 | 0.816740545228373 | -49 | -40 | -1 | ATGACGTCAT |
| MA0596.1 | SREBF2 | 7.466 | 0.816740545228373 | -49 | -40 | 1 | ATGACGTCAT |
| MA0326.1 | MAC1 | 3.896 | 0.816703969932673 | -57 | -50 | 1 | TGTGGTCG |
| MA0534.1 | EcR::usp | 6.508 | 0.816331176613599 | -56 | -42 | 1 | GTGGTCGATGACGTC |
| MA0445.1 | D | 7.652 | 0.816203306086118 | -72 | -62 | -1 | CTTTTTGTCTT |
| MA0418.1 | YAP6 | 11.933 | 0.815863472204631 | -55 | -36 | 1 | TGGTCGATGACGTCATGGAT |
| MA0309.1 | GZF3 | 4.933 | 0.81583551030215 | -51 | -44 | 1 | CGATGACG |
| MA0509.1 | Rfx1 | 7.597 | 0.813621256961956 | -47 | -34 | 1 | GACGTCATGGATAC |
| MA0035.3 | Gata1 | 3.165 | 0.811192974584725 | -73 | -63 | -1 | TTTTTGTCTTC |
| MA0199.1 | Optix | 4.572 | 0.809766332678752 | -39 | -35 | 1 | GGATA |
| MA0217.1 | caup | 4.231 | 0.808390585697071 | -71 | -67 | 1 | AGACA |
| MA0067.1 | Pax2 | 4.182 | 0.808218779346189 | -51 | -44 | -1 | CGTCATCG |
| MA0321.1 | INO2 | 5.890 | 0.807890601064158 | -60 | -52 | 1 | TCATGTGGT |
| MA0307.1 | GLN3 | 3.442 | 0.807760903641786 | -50 | -46 | 1 | GATGA |
| MA0059.1 | MYC::MAX | 8.218 | 0.806288168551965 | -61 | -51 | -1 | GACCACATGAC |
| MA0021.1 | Dof3 | 4.061 | 0.805550441604879 | -65 | -60 | 1 | AAAGGT |
| MA0210.1 | ara | 3.359 | 0.805461584870566 | -71 | -67 | 1 | AGACA |
| MA0466.1 | CEBPB | -1.420 | 0.805054714500035 | -50 | -40 | 1 | GATGACGTCAT |
| MA0261.1 | lin-14 | 4.041 | 0.804592591928992 | -73 | -68 | 1 | GAAGAC |
| MA0261.1 | lin-14 | 4.041 | 0.804592591928992 | -56 | -51 | -1 | GACCAC |
| MA0511.1 | RUNX2 | 6.721 | 0.803633475099496 | -63 | -49 | 1 | AGGTCATGTGGTCGA |
| MA0054.1 | myb.Ph3 | 5.485 | 0.802756764219967 | -66 | -58 | -1 | TGACCTTTT |
| MA0416.1 | YAP3 | 6.316 | 0.802045542049586 | -49 | -42 | 1 | ATGACGTC |
| MA0416.1 | YAP3 | 6.316 | 0.802045542049586 | -47 | -40 | -1 | ATGACGTC |

**Table H.** Tabulated Blastn results of HbII promoter-gene against HbI incomplete gene sequence.

| Query Id | Subject Id | % similarity | length | Misma  tches | Gap openings | Start query | End query | Start subject | End subject | E-value | Bit score |
| --- | --- | --- | --- | --- | --- | --- | --- | --- | --- | --- | --- |
| HbI_Incomplete Gene | HbII_Gene | 84.45 | 238 | 31 | 5 | 451 | 686 | 3891 | 4124 | 2.0E-67 | 246 |
|  |  | 84.89 | 225 | 25 | 4 | 1433 | 1651 | 6582 | 6803 | 9.0E-67 | 244 |
|  |  | 75 | 216 | 19 | 3 | 450 | 664 | 1710 | 1891 | 7.0E-43 | 165 |
|  |  | 86.92 | 214 | 27 | 1 | 452 | 664 | 2268 | 2481 | 1.0E-70 | 257 |
|  |  | 87.79 | 213 | 25 | 1 | 454 | 665 | 281 | 493 | 9.0E-73 | 264 |
|  |  | 85.45 | 213 | 22 | 4 | 1433 | 1639 | 3894 | 4103 | 1.0E-64 | 237 |
|  |  | 86.32 | 212 | 28 | 1 | 453 | 663 | 6581 | 6792 | 7.0E-68 | 248 |
|  |  | 84.43 | 212 | 25 | 3 | 1433 | 1638 | 281 | 490 | 2.0E-62 | 230 |
|  |  | 84.43 | 212 | 25 | 3 | 1433 | 1638 | 2270 | 2479 | 2.0E-62 | 230 |
|  |  | 80.14 | 146 | 16 | 3 | 1433 | 1572 | 1714 | 1852 | 4.0E-33 | 132 |
|  |  | 80.17 | 121 | 11 | 3 | 3275 | 3382 | 6792 | 6672 | 4.0E-26 | 109 |
|  |  | 77.69 | 121 | 14 | 3 | 3275 | 3382 | 2480 | 2360 | 1.0E-21 | 95.1 |
|  |  | 75.21 | 121 | 17 | 4 | 3275 | 3382 | 491 | 371 | 3.0E-16 | 77 |
|  |  | 77.5 | 120 | 13 | 5 | 3276 | 3382 | 4102 | 3984 | 5.0E-19 | 86 |
|  |  | 76.92 | 78 | 15 | 2 | 3179 | 3256 | 1639 | 1713 | 3.0E-09 | 53.6 |
|  |  | 90.91 | 77 | 5 | 2 | 1546 | 1621 | 4086 | 4011 | 2.0E-23 | 100 |
|  |  | 89.61 | 77 | 6 | 2 | 1546 | 1621 | 6774 | 6699 | 3.0E-22 | 96.9 |
|  |  | 88 | 75 | 8 | 1 | 572 | 645 | 4085 | 4011 | 1.0E-20 | 91.5 |
|  |  | 86.67 | 75 | 9 | 1 | 572 | 645 | 6773 | 6699 | 1.0E-19 | 87.8 |
|  |  | 82.67 | 75 | 12 | 1 | 572 | 645 | 472 | 398 | 3.0E-15 | 73.4 |
|  |  | 81.08 | 74 | 13 | 1 | 332 | 404 | 2271 | 2198 | 1.0E-13 | 68 |
|  |  | 79.73 | 74 | 14 | 1 | 3193 | 3266 | 2035 | 2107 | 6.0E-12 | 62.6 |
|  |  | 90.28 | 72 | 6 | 1 | 1551 | 1621 | 2458 | 2387 | 1.0E-21 | 95.1 |
|  |  | 87.5 | 72 | 8 | 1 | 1551 | 1621 | 469 | 398 | 5.0E-19 | 86 |
|  |  | 86.11 | 72 | 9 | 1 | 575 | 645 | 2458 | 2387 | 6.0E-18 | 82.4 |
|  |  | 77.19 | 57 | 6 | 3 | 3319 | 3368 | 1831 | 1887 | 2.0E-04 | 37.4 |
|  |  | 85.19 | 54 | 8 | 0 | 3329 | 3382 | 1852 | 1799 | 6.0E-12 | 62.6 |
|  |  | 83.33 | 48 | 5 | 2 | 1609 | 1654 | 3923 | 3877 | 2.0E-06 | 44.6 |
|  |  | 81.4 | 43 | 1 | 3 | 1546 | 1582 | 1872 | 1831 | 8.0E-04 | 35.6 |
|  |  | 82.93 | 41 | 1 | 1 | 607 | 641 | 1871 | 1831 | 2.0E-06 | 44.6 |
|  |  | 92.11 | 38 | 2 | 1 | 3318 | 3355 | 4050 | 4086 | 1.0E-08 | 51.8 |
|  |  | 92.11 | 38 | 2 | 1 | 3318 | 3355 | 6738 | 6774 | 1.0E-08 | 51.8 |
|  |  | 84.21 | 38 | 5 | 1 | 1425 | 1462 | 1896 | 1860 | 7.0E-05 | 39.2 |
|  |  | 82.35 | 34 | 6 | 0 | 3009 | 3042 | 2813 | 2780 | 8.0E-04 | 35.6 |
|  |  | 93.94 | 33 | 2 | 0 | 3318 | 3350 | 2426 | 2458 | 1.0E-08 | 51.8 |
|  |  | 90.91 | 33 | 3 | 0 | 3318 | 3350 | 437 | 469 | 4.0E-07 | 46.4 |
|  |  | 87.88 | 33 | 4 | 0 | 1609 | 1641 | 1743 | 1711 | 5.0E-06 | 42.8 |
|  |  | 90.62 | 32 | 3 | 0 | 1609 | 1640 | 310 | 279 | 2.0E-06 | 44.6 |
|  |  | 90.32 | 31 | 3 | 0 | 633 | 663 | 1743 | 1713 | 5.0E-06 | 42.8 |
|  |  | 87.1 | 31 | 4 | 0 | 633 | 663 | 3923 | 3893 | 7.0E-05 | 39.2 |
|  |  | 90 | 30 | 3 | 0 | 633 | 662 | 310 | 281 | 2.0E-05 | 41 |
|  |  | 89.29 | 28 | 3 | 0 | 3275 | 3302 | 3893 | 3920 | 2.0E-04 | 37.4 |
|  |  | 88.89 | 27 | 3 | 0 | 3276 | 3302 | 281 | 307 | 8.0E-04 | 35.6 |

**Table I.** Tabulated Blastn results of Consensus sequence 1 against mollusk (taxid:6447) database reference genomic sequences.

| Query | GI | Reference | Organism | %  similarity | length | Misma  tches | Gap  openings | Start  query | End  query | Start  subject | End  subject | E-value | Bit score |
| --- | --- | --- | --- | --- | --- | --- | --- | --- | --- | --- | --- | --- | --- |
| Consensus1 | 902505392 | NW_013305125.1 | Biomphalaria glabrata | 80 | 45 | 8 | 1 | 15 | 58 | 109 | 65 | 2.8 | 37.4 |
| Consensus1 | 902371499 | NW_013435045.1 | Biomphalaria glabrata | 81.395 | 43 | 7 | 1 | 17 | 58 | 20275 | 20233 | 0.81 | 39.2 |
| Consensus1 | 902371499 | NW_013435045.1 | Biomphalaria glabrata | 81.395 | 43 | 7 | 1 | 17 | 58 | 20869 | 20827 | 0.81 | 39.2 |
| Consensus1 | 902389107 | NW_013421391.1 | Biomphalaria glabrata | 81.395 | 43 | 7 | 1 | 17 | 58 | 894 | 936 | 0.81 | 39.2 |
| Consensus1 | 902505392 | NW_013305125.1 | Biomphalaria glabrata | 81.395 | 43 | 7 | 1 | 17 | 58 | 175 | 133 | 0.81 | 39.2 |
| Consensus1 | 902371499 | NW_013435045.1 | Biomphalaria glabrata | 80.952 | 42 | 7 | 1 | 17 | 57 | 20557 | 20598 | 2.8 | 37.4 |
| Consensus1 | 902389107 | NW_013421391.1 | Biomphalaria glabrata | 80.488 | 41 | 7 | 1 | 17 | 56 | 1489 | 1529 | 9.9 | 35.6 |
| Consensus1 | 761390853 | NW_011934815.1 | Crassostrea gigas | 78.571 | 42 | 9 | 0 | 17 | 58 | 84818 | 84859 | 9.9 | 35.6 |
| Consensus1 | 902685602 | NW_013193912.1 | Biomphalaria glabrata | 77.551 | 49 | 11 | 0 | 20 | 68 | 347422 | 347470 | 0.81 | 39.2 |
| Consensus1 | 902388817 | NW_013421681.1 | Biomphalaria glabrata | 86.667 | 30 | 4 | 0 | 25 | 54 | 18188 | 18159 | 2.8 | 37.4 |
| Consensus1 | 761388173 | NW_011937495.1 | Crassostrea gigas | 78.846 | 52 | 8 | 2 | 27 | 77 | 41406 | 41455 | 0.81 | 39.2 |
| Consensus1 | 761390858 | NW_011934810.1 | Crassostrea gigas | 86.667 | 30 | 4 | 0 | 27 | 56 | 69231 | 69260 | 2.8 | 37.4 |
| Consensus1 | 761389426 | NW_011936242.1 | Crassostrea gigas | 88.889 | 27 | 3 | 0 | 27 | 53 | 139608 | 139582 | 9.9 | 35.6 |
| Consensus1 | 761388805 | NW_011936863.1 | Crassostrea gigas | 87.5 | 32 | 3 | 1 | 28 | 58 | 122844 | 122813 | 2.8 | 37.4 |
| Consensus1 | 761388589 | NW_011937079.1 | Crassostrea gigas | 86.207 | 29 | 4 | 0 | 30 | 58 | 251460 | 251432 | 9.9 | 35.6 |
| Consensus1 | 676517172 | NW_008710950.1 | Lottia gigantea | 75.806 | 62 | 10 | 2 | 30 | 90 | 639820 | 639877 | 0.23 | 41 |
| Consensus1 | 761389650 | NW_011936018.1 | Crassostrea gigas | 76.471 | 51 | 11 | 1 | 31 | 80 | 457673 | 457623 | 9.9 | 35.6 |
| Consensus1 | 761389670 | NW_011935998.1 | Crassostrea gigas | 78.723 | 47 | 9 | 1 | 31 | 76 | 686801 | 686755 | 2.8 | 37.4 |
| Consensus1 | 761389947 | NW_011935721.1 | Crassostrea gigas | 89.286 | 28 | 3 | 0 | 31 | 58 | 4611 | 4638 | 2.8 | 37.4 |
| Consensus1 | 902354741 | NW_013451803.1 | Biomphalaria glabrata | 80 | 40 | 8 | 0 | 32 | 71 | 2534 | 2495 | 2.8 | 37.4 |
| Consensus1 | 902541565 | NW_013282595.1 | Biomphalaria glabrata | 82.857 | 35 | 6 | 0 | 32 | 66 | 224055 | 224089 | 2.8 | 37.4 |
| Consensus1 | 902358332 | NW_013448212.1 | Biomphalaria glabrata | 89.286 | 28 | 3 | 0 | 32 | 59 | 475819 | 475792 | 2.8 | 37.4 |
| Consensus1 | 761389893 | NW_011935775.1 | Crassostrea gigas | 86.207 | 29 | 4 | 0 | 32 | 60 | 213767 | 213739 | 9.9 | 35.6 |
| Consensus1 | 761389244 | NW_011936424.1 | Crassostrea gigas | 88.889 | 27 | 3 | 0 | 32 | 58 | 57661 | 57635 | 9.9 | 35.6 |
| Consensus1 | 761390702 | NW_011934966.1 | Crassostrea gigas | 92.593 | 27 | 1 | 1 | 33 | 58 | 88362 | 88336 | 2.8 | 37.4 |
| Consensus1 | 761389482 | NW_011936186.1 | Crassostrea gigas | 92.308 | 26 | 1 | 1 | 33 | 58 | 124864 | 124840 | 9.9 | 35.6 |
| Consensus1 | 761388760 | NW_011936908.1 | Crassostrea gigas | 82.051 | 39 | 6 | 1 | 35 | 72 | 289417 | 289379 | 9.9 | 35.6 |
| Consensus1 | 761389755 | NW_011935913.1 | Crassostrea gigas | 91.667 | 24 | 2 | 0 | 36 | 59 | 22354 | 22377 | 9.9 | 35.6 |
| Consensus1 | 902520438 | NW_013297095.1 | Biomphalaria glabrata | 84.211 | 38 | 5 | 1 | 37 | 73 | 39648 | 39611 | 0.81 | 39.2 |
| Consensus1 | 902520438 | NW_013297095.1 | Biomphalaria glabrata | 84.211 | 38 | 5 | 1 | 37 | 73 | 39733 | 39696 | 0.81 | 39.2 |
| Consensus1 | 902494104 | NW_013316394.1 | Biomphalaria glabrata | 84.211 | 38 | 5 | 1 | 37 | 73 | 1377 | 1414 | 0.81 | 39.2 |
| Consensus1 | 902478209 | NW_013332289.1 | Biomphalaria glabrata | 80.392 | 51 | 9 | 1 | 38 | 87 | 50419 | 50369 | 0.019 | 44.6 |
| Consensus1 | 902357556 | NW_013448988.1 | Biomphalaria glabrata | 92 | 25 | 2 | 0 | 39 | 63 | 5585 | 5609 | 2.8 | 37.4 |
| Consensus1 | 902372650 | NW_013433894.1 | Biomphalaria glabrata | 87.097 | 31 | 4 | 0 | 42 | 72 | 211 | 241 | 0.81 | 39.2 |
| Consensus1 | 761390684 | NW_011934984.1 | Crassostrea gigas | 86.207 | 29 | 4 | 0 | 42 | 70 | 351206 | 351178 | 9.9 | 35.6 |
| Consensus1 | 523418782 | NW_004798267.1 | Aplysia californica | 92.593 | 27 | 1 | 1 | 45 | 71 | 53977 | 54002 | 2.8 | 37.4 |
| Consensus1 | 761390477 | NW_011935191.1 | Crassostrea gigas | 95.455 | 22 | 1 | 0 | 45 | 66 | 337104 | 337125 | 9.9 | 35.6 |
| Consensus1 | 761389836 | NW_011935832.1 | Crassostrea gigas | 95.455 | 22 | 1 | 0 | 45 | 66 | 91078 | 91057 | 9.9 | 35.6 |
| Consensus1 | 902290705 | NW_013515138.1 | Biomphalaria glabrata | 75.41 | 61 | 13 | 1 | 46 | 104 | 7452 | 7392 | 0.23 | 41 |
| Consensus1 | 761389416 | NW_011936252.1 | Crassostrea gigas | 82.353 | 34 | 6 | 0 | 46 | 79 | 123007 | 122974 | 9.9 | 35.6 |
| Consensus1 | 676510841 | NW_008708707.1 | Lottia gigantea | 83.784 | 37 | 4 | 1 | 46 | 80 | 1090271 | 1090235 | 2.8 | 37.4 |
| Consensus1 | 676509931 | NW_008708367.1 | Lottia gigantea | 83.784 | 37 | 5 | 1 | 47 | 83 | 306336 | 306301 | 2.8 | 37.4 |
| Consensus1 | 761390391 | NW_011935277.1 | Crassostrea gigas | 82.051 | 39 | 5 | 1 | 49 | 87 | 301453 | 301417 | 2.8 | 37.4 |
| Consensus1 | 761388686 | NW_011936982.1 | Crassostrea gigas | 82.353 | 34 | 6 | 0 | 49 | 82 | 167338 | 167371 | 9.9 | 35.6 |
| Consensus1 | 676506060 | NW_008706747.1 | Lottia gigantea | 84.848 | 33 | 3 | 1 | 51 | 83 | 4155186 | 4155216 | 9.9 | 35.6 |
| Consensus1 | 761389856 | NW_011935812.1 | Crassostrea gigas | 83.333 | 36 | 5 | 1 | 52 | 87 | 36273 | 36239 | 9.9 | 35.6 |
| Consensus1 | 676510841 | NW_008708707.1 | Lottia gigantea | 100 | 19 | 0 | 0 | 52 | 70 | 2246653 | 2246671 | 9.9 | 35.6 |
| Consensus1 | 482651634 | NC_021146.1 | Sepia pharaonis | 87.5 | 32 | 3 | 1 | 53 | 83 | 8239 | 8270 | 2.8 | 37.4 |
| Consensus1 | 761390564 | NW_011935104.1 | Crassostrea gigas | 73.684 | 57 | 6 | 2 | 54 | 101 | 124232 | 124288 | 9.9 | 35.6 |
| Consensus1 | 761389849 | NW_011935819.1 | Crassostrea gigas | 80 | 45 | 7 | 1 | 54 | 98 | 89559 | 89517 | 0.81 | 39.2 |
| Consensus1 | 523419613 | NW_004797436.1 | Aplysia californica | 83.784 | 37 | 6 | 0 | 56 | 92 | 131989 | 132025 | 0.23 | 41 |
| Consensus1 | 761388634 | NW_011937034.1 | Crassostrea gigas | 92 | 25 | 2 | 0 | 57 | 81 | 179999 | 180023 | 2.8 | 37.4 |
| Consensus1 | 523419628 | NW_004797421.1 | Aplysia californica | 87.5 | 32 | 3 | 1 | 58 | 89 | 796785 | 796755 | 2.8 | 37.4 |
| Consensus1 | 902387048 | NW_013423450.1 | Biomphalaria glabrata | 80.488 | 41 | 8 | 0 | 58 | 98 | 18407 | 18367 | 0.81 | 39.2 |
| Consensus1 | 761390859 | NW_011934809.1 | Crassostrea gigas | 84.211 | 38 | 5 | 1 | 60 | 96 | 619933 | 619896 | 0.81 | 39.2 |
| Consensus1 | 761388403 | NW_011937265.1 | Crassostrea gigas | 82.857 | 35 | 6 | 0 | 60 | 94 | 240856 | 240890 | 2.8 | 37.4 |
| Consensus1 | 523419770 | NW_004797279.1 | Aplysia californica | 82.5 | 40 | 4 | 2 | 61 | 98 | 1753611 | 1753573 | 9.9 | 35.6 |
| Consensus1 | 761388729 | NW_011936939.1 | Crassostrea gigas | 83.333 | 36 | 5 | 1 | 61 | 95 | 72772 | 72807 | 9.9 | 35.6 |
| Consensus1 | 523419373 | NW_004797676.1 | Aplysia californica | 88.889 | 36 | 2 | 2 | 62 | 96 | 594550 | 594516 | 0.23 | 41 |
| Consensus1 | 902591642 | NW_013254356.1 | Biomphalaria glabrata | 87.179 | 39 | 4 | 1 | 62 | 100 | 5581 | 5618 | 0.019 | 44.6 |
| Consensus1 | 902453345 | NW_013357153.1 | Biomphalaria glabrata | 83.784 | 37 | 5 | 1 | 62 | 98 | 33355 | 33390 | 2.8 | 37.4 |
| Consensus1 | 761389517 | NW_011936151.1 | Crassostrea gigas | 80.488 | 41 | 5 | 1 | 62 | 99 | 306256 | 306216 | 2.8 | 37.4 |
| Consensus1 | 761389445 | NW_011936223.1 | Crassostrea gigas | 90.323 | 31 | 2 | 1 | 62 | 92 | 30134 | 30105 | 0.81 | 39.2 |
| Consensus1 | 761390037 | NW_011935631.1 | Crassostrea gigas | 89.655 | 29 | 2 | 1 | 62 | 90 | 47679 | 47652 | 9.9 | 35.6 |
| Consensus1 | 523419751 | NW_004797298.1 | Aplysia californica | 86.486 | 37 | 4 | 1 | 64 | 99 | 1309063 | 1309027 | 0.23 | 41 |
| Consensus1 | 902359146 | NW_013447398.1 | Biomphalaria glabrata | 82.857 | 35 | 6 | 0 | 64 | 98 | 810 | 844 | 2.8 | 37.4 |
| Consensus1 | 761389911 | NW_011935757.1 | Crassostrea gigas | 82.051 | 39 | 7 | 0 | 64 | 102 | 358625 | 358663 | 0.81 | 39.2 |
| Consensus1 | 761389581 | NW_011936087.1 | Crassostrea gigas | 82.051 | 39 | 7 | 0 | 64 | 102 | 451413 | 451375 | 0.81 | 39.2 |
| Consensus1 | 761389987 | NW_011935681.1 | Crassostrea gigas | 79.487 | 39 | 8 | 0 | 64 | 102 | 412383 | 412345 | 9.9 | 35.6 |
| Consensus1 | 902392839 | NW_013417659.1 | Biomphalaria glabrata | 100 | 20 | 0 | 0 | 65 | 84 | 11402 | 11383 | 2.8 | 37.4 |
| Consensus1 | 902395605 | NW_013414893.1 | Biomphalaria glabrata | 100 | 20 | 0 | 0 | 65 | 84 | 145 | 126 | 2.8 | 37.4 |
| Consensus1 | 902370904 | NW_013435640.1 | Biomphalaria glabrata | 100 | 20 | 0 | 0 | 65 | 84 | 412 | 393 | 2.8 | 37.4 |
| Consensus1 | 761389568 | NW_011936100.1 | Crassostrea gigas | 83.784 | 37 | 5 | 1 | 66 | 101 | 81434 | 81398 | 2.8 | 37.4 |
| Consensus1 | 761390335 | NW_011935333.1 | Crassostrea gigas | 87.5 | 32 | 4 | 0 | 67 | 98 | 110653 | 110684 | 0.23 | 41 |
| Consensus1 | 523418852 | NW_004798197.1 | Aplysia californica | 87.5 | 32 | 3 | 1 | 69 | 100 | 85139 | 85169 | 2.8 | 37.4 |
| Consensus1 | 523419481 | NW_004797568.1 | Aplysia californica | 92.308 | 26 | 2 | 0 | 69 | 94 | 403455 | 403430 | 0.81 | 39.2 |
| Consensus1 | 902309860 | NW_013496684.1 | Biomphalaria glabrata | 86.667 | 30 | 4 | 0 | 69 | 98 | 19053 | 19082 | 2.8 | 37.4 |
| Consensus1 | 761389949 | NW_011935719.1 | Crassostrea gigas | 89.655 | 29 | 1 | 1 | 69 | 97 | 36791 | 36765 | 2.8 | 37.4 |
| Consensus1 | 761390339 | NW_011935329.1 | Crassostrea gigas | 86.667 | 30 | 4 | 0 | 70 | 99 | 7151 | 7122 | 2.8 | 37.4 |
| Consensus1 | 761390568 | NW_011935100.1 | Crassostrea gigas | 86.207 | 29 | 4 | 0 | 70 | 98 | 49938 | 49966 | 9.9 | 35.6 |
| Consensus1 | 761387880 | NW_011937788.1 | Crassostrea gigas | 89.286 | 28 | 3 | 0 | 70 | 97 | 167897 | 167924 | 2.8 | 37.4 |
| Consensus1 | 902586011 | NW_013256279.1 | Biomphalaria glabrata | 90.323 | 31 | 2 | 1 | 71 | 100 | 2885 | 2915 | 0.81 | 39.2 |
| Consensus1 | 761390227 | NW_011935441.1 | Crassostrea gigas | 84.848 | 33 | 3 | 1 | 71 | 103 | 71294 | 71264 | 9.9 | 35.6 |
| Consensus1 | 523419136 | NW_004797913.1 | Aplysia californica | 80.851 | 47 | 7 | 2 | 72 | 117 | 276379 | 276424 | 2.8 | 37.4 |
| Consensus1 | 676510841 | NW_008708707.1 | Lottia gigantea | 92.308 | 26 | 2 | 0 | 73 | 98 | 1330623 | 1330598 | 0.81 | 39.2 |
| Consensus1 | 676506060 | NW_008706747.1 | Lottia gigantea | 92 | 25 | 2 | 0 | 73 | 97 | 2017071 | 2017095 | 2.8 | 37.4 |
| Consensus1 | 523418262 | NW_004798787.1 | Aplysia californica | 87.5 | 32 | 3 | 1 | 74 | 105 | 35680 | 35650 | 2.8 | 37.4 |
| Consensus1 | 902685370 | NW_013194144.1 | Biomphalaria glabrata | 92.857 | 28 | 1 | 1 | 74 | 101 | 89557 | 89531 | 0.81 | 39.2 |
| Consensus1 | 761389049 | NW_011936619.1 | Crassostrea gigas | 92 | 25 | 2 | 0 | 74 | 98 | 29912 | 29888 | 2.8 | 37.4 |
| Consensus1 | 676510665 | NW_008708637.1 | Lottia gigantea | 92 | 25 | 2 | 0 | 74 | 98 | 177880 | 177856 | 2.8 | 37.4 |
| Consensus1 | 761390422 | NW_011935246.1 | Crassostrea gigas | 80.488 | 41 | 7 | 1 | 75 | 115 | 107108 | 107069 | 9.9 | 35.6 |
| Consensus1 | 761389497 | NW_011936171.1 | Crassostrea gigas | 86.207 | 29 | 4 | 0 | 75 | 103 | 98435 | 98463 | 9.9 | 35.6 |
| Consensus1 | 761389926 | NW_011935742.1 | Crassostrea gigas | 95.455 | 22 | 1 | 0 | 79 | 100 | 181889 | 181910 | 9.9 | 35.6 |
| Consensus1 | 676513982 | NW_008709945.1 | Lottia gigantea | 96 | 25 | 1 | 0 | 79 | 103 | 1066056 | 1066080 | 0.23 | 41 |
| Consensus1 | 523419770 | NW_004797279.1 | Aplysia californica | 83.333 | 36 | 6 | 0 | 80 | 115 | 3044266 | 3044301 | 0.81 | 39.2 |
| Consensus1 | 676513917 | NW_008709919.1 | Lottia gigantea | 100 | 20 | 0 | 0 | 80 | 99 | 99684 | 99665 | 2.8 | 37.4 |
| Consensus1 | 761387860 | NW_011937808.1 | Crassostrea gigas | 86.667 | 30 | 4 | 0 | 81 | 110 | 798754 | 798783 | 2.8 | 37.4 |
| Consensus1 | 761389039 | NW_011936629.1 | Crassostrea gigas | 100 | 20 | 0 | 0 | 85 | 104 | 729578 | 729597 | 2.8 | 37.4 |
| Consensus1 | 902666173 | NW_013213287.1 | Biomphalaria glabrata | 95.652 | 23 | 1 | 0 | 91 | 113 | 10143 | 10121 | 2.8 | 37.4 |
| Consensus1 | 523419645 | NW_004797404.1 | Aplysia californica | 83.784 | 37 | 5 | 1 | 109 | 145 | 1364790 | 1364755 | 2.8 | 37.4 |
| Consensus1 | 523419626 | NW_004797423.1 | Aplysia californica | 87.879 | 33 | 3 | 1 | 109 | 141 | 576367 | 576398 | 0.81 | 39.2 |
| Consensus1 | 761390248 | NW_011935420.1 | Crassostrea gigas | 86.207 | 29 | 4 | 0 | 178 | 206 | 123948 | 123976 | 9.9 | 35.6 |
| Consensus1 | 761389547 | NW_011936121.1 | Crassostrea gigas | 95.652 | 23 | 1 | 0 | 180 | 202 | 325046 | 325068 | 2.8 | 37.4 |

**Table J.** Tabulated Blastn results of Consensus sequence 2 against mollusk (taxid:6447) database reference genomic sequences.

| Query | GI | Reference | Organism | % similarity | length | Mismatches | Gap openings | Start query | End Query | Start subject | End Subject | E-value | Bit score |
| --- | --- | --- | --- | --- | --- | --- | --- | --- | --- | --- | --- | --- | --- |
| Consensus2 | 902685530 | NW_013193984.1 | Biomphalaria glabrata | 92 | 25 | 2 | 0 | 6 | 30 | 71101 | 71077 | 0.94 | 37.4 |
| Consensus2 | 523419072 | NW_004797977.1 | Aplysia californica | 85.294 | 34 | 4 | 1 | 2 | 34 | 393290 | 393257 | 3.3 | 35.6 |
| Consensus2 | 523418709 | NW_004798340.1 | Aplysia californica | 86.207 | 29 | 4 | 0 | 27 | 55 | 186014 | 185986 | 3.3 | 35.6 |
| Consensus2 | 761380808 | NW_011942122.1 | Crassostrea gigas | 91.667 | 24 | 2 | 0 | 18 | 41 | 226359 | 226382 | 3.3 | 35.6 |
| Consensus2 | 902530289 | NW_013287244.1 | Biomphalaria glabrata | 100 | 19 | 0 | 0 | 27 | 45 | 46761 | 46779 | 3.3 | 35.6 |
| Consensus2 | 902394215 | NW_013416283.1 | Biomphalaria glabrata | 91.667 | 24 | 2 | 0 | 66 | 89 | 72345 | 72368 | 3.3 | 35.6 |
| Consensus2 | 902303663 | NW_013502881.1 | Biomphalaria glabrata | 100 | 19 | 0 | 0 | 51 | 69 | 10037 | 10055 | 3.3 | 35.6 |
| Consensus2 | 902348424 | NW_013458120.1 | Biomphalaria glabrata | 100 | 19 | 0 | 0 | 27 | 45 | 991 | 973 | 3.3 | 35.6 |
| Consensus2 | 761389459 | NW_011936209.1 | Crassostrea gigas | 92.308 | 26 | 1 | 1 | 36 | 60 | 156613 | 156588 | 3.3 | 35.6 |

**Table K.** Tabulated Blastn results of Consensus sequence 3 against mollusk (taxid:6447) database reference genomic sequences.

| Query | GI | Reference | Organism | % similarity | length | Misma  tches | Gap  openings | Start  query | End  query | Start  subject | End  subject | E-value | Bit score |
| --- | --- | --- | --- | --- | --- | --- | --- | --- | --- | --- | --- | --- | --- |
| Consensus3 | 761389211 | NW_011936457.1 | Crassostrea gigas | 83.673 | 49 | 5 | 3 | 12 | 59 | 269077 | 269123 | 0.012 | 42.8 |
| Consensus3 | 902374715 | NW_013431829.1 | Biomphalaria glabrata | 90 | 30 | 3 | 0 | 12 | 41 | 142803 | 142832 | 0.042 | 41 |
| Consensus3 | 523419388 | NW_004797661.1 | Aplysia californica | 85 | 40 | 2 | 2 | 12 | 49 | 559771 | 559734 | 0.042 | 41 |
| Consensus3 | 761390061 | NW_011935607.1 | Crassostrea gigas | 90 | 30 | 3 | 0 | 23 | 52 | 222912 | 222883 | 0.042 | 41 |
| Consensus3 | 761389050 | NW_011936618.1 | Crassostrea gigas | 84 | 50 | 4 | 4 | 12 | 59 | 84596 | 84643 | 0.042 | 41 |
| Consensus3 | 676510470 | NW_008708556.1 | Lottia gigantea | 83.333 | 42 | 3 | 2 | 24 | 61 | 184064 | 184105 | 0.15 | 39.2 |
| Consensus3 | 902343930 | NW_013462614.1 | Biomphalaria glabrata | 92.308 | 26 | 2 | 0 | 24 | 49 | 19571 | 19596 | 0.15 | 39.2 |
| Consensus3 | 761388252 | NW_011937416.1 | Crassostrea gigas | 92.308 | 26 | 2 | 0 | 25 | 50 | 229397 | 229422 | 0.15 | 39.2 |
| Consensus3 | 761382397 | NW_011940652.1 | Crassostrea gigas | 81.395 | 43 | 7 | 1 | 10 | 52 | 2619 | 2660 | 0.15 | 39.2 |
| Consensus3 | 902685793 | NW_013193721.1 | Biomphalaria glabrata | 81.579 | 38 | 7 | 0 | 7 | 44 | 119003 | 119040 | 0.52 | 37.4 |
| Consensus3 | 761390374 | NW_011935294.1 | Crassostrea gigas | 92 | 25 | 2 | 0 | 33 | 57 | 800823 | 800799 | 0.52 | 37.4 |
| Consensus3 | 902389507 | NW_013420991.1 | Biomphalaria glabrata | 80.952 | 42 | 7 | 1 | 20 | 61 | 21982 | 22022 | 0.52 | 37.4 |
| Consensus3 | 902308588 | NW_013497956.1 | Biomphalaria glabrata | 92.593 | 27 | 1 | 1 | 36 | 61 | 320 | 294 | 0.52 | 37.4 |
| Consensus3 | 761390694 | NW_011934974.1 | Crassostrea gigas | 84.848 | 33 | 5 | 0 | 29 | 61 | 202654 | 202686 | 0.52 | 37.4 |
| Consensus3 | 761390222 | NW_011935446.1 | Crassostrea gigas | 89.286 | 28 | 3 | 0 | 25 | 52 | 231340 | 231313 | 0.52 | 37.4 |
| Consensus3 | 761389891 | NW_011935777.1 | Crassostrea gigas | 95.652 | 23 | 1 | 0 | 36 | 58 | 62098 | 62120 | 0.52 | 37.4 |
| Consensus3 | 761388758 | NW_011936910.1 | Crassostrea gigas | 85.294 | 34 | 3 | 1 | 27 | 60 | 140053 | 140084 | 0.52 | 37.4 |
| Consensus3 | 761388368 | NW_011937300.1 | Crassostrea gigas | 90 | 30 | 2 | 1 | 22 | 51 | 213458 | 213430 | 0.52 | 37.4 |
| Consensus3 | 761381169 | NW_011941761.1 | Crassostrea gigas | 92 | 25 | 2 | 0 | 33 | 57 | 4973 | 4997 | 0.52 | 37.4 |
| Consensus3 | 676512829 | NW_008709463.1 | Lottia gigantea | 83.784 | 37 | 5 | 1 | 26 | 62 | 489468 | 489433 | 0.52 | 37.4 |
| Consensus3 | 676512829 | NW_008709463.1 | Lottia gigantea | 81.818 | 33 | 6 | 0 | 1 | 33 | 1413821 | 1413789 | 6.3 | 33.7 |
| Consensus3 | 902566678 | NW_013272627.1 | Biomphalaria glabrata | 89.655 | 29 | 2 | 1 | 25 | 52 | 298567 | 298539 | 1.8 | 35.6 |
| Consensus3 | 523419498 | NW_004797551.1 | Aplysia californica | 88.889 | 27 | 3 | 0 | 25 | 51 | 726349 | 726323 | 1.8 | 35.6 |
| Consensus3 | 523419458 | NW_004797591.1 | Aplysia californica | 88.889 | 27 | 3 | 0 | 25 | 51 | 158852 | 158878 | 1.8 | 35.6 |
| Consensus3 | 523419458 | NW_004797591.1 | Aplysia californica | 88.889 | 27 | 3 | 0 | 25 | 51 | 160460 | 160486 | 1.8 | 35.6 |
| Consensus3 | 523419367 | NW_004797682.1 | Aplysia californica | 88.889 | 27 | 3 | 0 | 19 | 45 | 43145 | 43119 | 1.8 | 35.6 |
| Consensus3 | 523418611 | NW_004798438.1 | Aplysia californica | 92.308 | 26 | 1 | 1 | 17 | 42 | 139636 | 139660 | 1.8 | 35.6 |
| Consensus3 | 676506995 | NW_008707167.1 | Lottia gigantea | 91.667 | 24 | 2 | 0 | 36 | 59 | 237659 | 237636 | 1.8 | 35.6 |
| Consensus3 | 676507027 | NW_008707180.1 | Lottia gigantea | 91.667 | 24 | 2 | 0 | 26 | 49 | 513786 | 513763 | 1.8 | 35.6 |
| Consensus3 | 676507098 | NW_008707205.1 | Lottia gigantea | 84.375 | 32 | 1 | 1 | 30 | 61 | 231011 | 231038 | 1.8 | 35.6 |
| Consensus3 | 676510641 | NW_008708627.1 | Lottia gigantea | 81.081 | 37 | 3 | 1 | 25 | 61 | 97812 | 97780 | 1.8 | 35.6 |
| Consensus3 | 761388906 | NW_011936762.1 | Crassostrea gigas | 91.667 | 24 | 2 | 0 | 22 | 45 | 219381 | 219358 | 1.8 | 35.6 |
| Consensus3 | 902454666 | NW_013355832.1 | Biomphalaria glabrata | 92.308 | 26 | 1 | 1 | 26 | 51 | 31159 | 31135 | 1.8 | 35.6 |
| Consensus3 | 902368156 | NW_013438388.1 | Biomphalaria glabrata | 91.667 | 24 | 2 | 0 | 28 | 51 | 26880 | 26903 | 1.8 | 35.6 |
| Consensus3 | 902353393 | NW_013453151.1 | Biomphalaria glabrata | 91.667 | 24 | 2 | 0 | 38 | 61 | 756 | 779 | 1.8 | 35.6 |
| Consensus3 | 902686244 | NW_013193270.1 | Biomphalaria glabrata | 91.667 | 24 | 2 | 0 | 26 | 49 | 82800 | 82777 | 1.8 | 35.6 |
| Consensus3 | 902686234 | NW_013193280.1 | Biomphalaria glabrata | 100 | 19 | 0 | 0 | 10 | 28 | 70657 | 70675 | 1.8 | 35.6 |
| Consensus3 | 902629021 | NW_013243413.1 | Biomphalaria glabrata | 91.667 | 24 | 2 | 0 | 38 | 61 | 88 | 65 | 1.8 | 35.6 |
| Consensus3 | 761390394 | NW_011935274.1 | Crassostrea gigas | 95.455 | 22 | 1 | 0 | 34 | 55 | 40438 | 40459 | 1.8 | 35.6 |
| Consensus3 | 761390389 | NW_011935279.1 | Crassostrea gigas | 91.667 | 24 | 2 | 0 | 22 | 45 | 633086 | 633109 | 1.8 | 35.6 |
| Consensus3 | 761390129 | NW_011935539.1 | Crassostrea gigas | 91.667 | 24 | 2 | 0 | 8 | 31 | 192963 | 192940 | 1.8 | 35.6 |
| Consensus3 | 761389568 | NW_011936100.1 | Crassostrea gigas | 91.667 | 24 | 2 | 0 | 24 | 47 | 33950 | 33927 | 1.8 | 35.6 |
| Consensus3 | 761388772 | NW_011936896.1 | Crassostrea gigas | 100 | 19 | 0 | 0 | 43 | 61 | 41512 | 41494 | 1.8 | 35.6 |
| Consensus3 | 761388545 | NW_011937123.1 | Crassostrea gigas | 95.455 | 22 | 1 | 0 | 30 | 51 | 131756 | 131777 | 1.8 | 35.6 |
| Consensus3 | 523419770 | NW_004797279.1 | Aplysia californica | 88.889 | 27 | 3 | 0 | 25 | 51 | 948957 | 948983 | 1.8 | 35.6 |
| Consensus3 | 523419639 | NW_004797410.1 | Aplysia californica | 87.097 | 31 | 3 | 1 | 32 | 61 | 437789 | 437819 | 1.8 | 35.6 |
| Consensus3 | 523419615 | NW_004797434.1 | Aplysia californica | 86.207 | 29 | 4 | 0 | 9 | 37 | 208262 | 208234 | 1.8 | 35.6 |
| Consensus3 | 676508076 | NW_008707617.1 | Lottia gigantea | 95.455 | 22 | 1 | 0 | 40 | 61 | 2017208 | 2017187 | 1.8 | 35.6 |
| Consensus3 | 676508076 | NW_008707617.1 | Lottia gigantea | 91.304 | 23 | 2 | 0 | 40 | 62 | 3178313 | 3178291 | 6.3 | 33.7 |
| Consensus3 | 676509754 | NW_008708301.1 | Lottia gigantea | 84.848 | 33 | 3 | 1 | 31 | 61 | 2880619 | 2880587 | 1.8 | 35.6 |
| Consensus3 | 676509754 | NW_008708301.1 | Lottia gigantea | 88.462 | 26 | 3 | 0 | 19 | 44 | 1404299 | 1404274 | 6.3 | 33.7 |
| Consensus3 | 676510064 | NW_008708401.1 | Lottia gigantea | 88.889 | 27 | 3 | 0 | 34 | 60 | 55745 | 55771 | 1.8 | 35.6 |
| Consensus3 | 676510064 | NW_008708401.1 | Lottia gigantea | 100 | 18 | 0 | 0 | 33 | 50 | 2904885 | 2904902 | 6.3 | 33.7 |
| Consensus3 | 676510220 | NW_008708458.1 | Lottia gigantea | 95.455 | 22 | 1 | 0 | 24 | 45 | 3368355 | 3368376 | 1.8 | 35.6 |
| Consensus3 | 676510220 | NW_008708458.1 | Lottia gigantea | 83.871 | 31 | 1 | 1 | 31 | 61 | 2011332 | 2011306 | 6.3 | 33.7 |
| Consensus3 | 676512290 | NW_008709259.1 | Lottia gigantea | 95.455 | 22 | 1 | 0 | 40 | 61 | 498109 | 498088 | 1.8 | 35.6 |
| Consensus3 | 676512477 | NW_008709333.1 | Lottia gigantea | 91.667 | 24 | 2 | 0 | 22 | 45 | 594448 | 594425 | 1.8 | 35.6 |
| Consensus3 | 676515497 | NW_008710536.1 | Lottia gigantea | 82.5 | 40 | 4 | 2 | 13 | 51 | 21944 | 21907 | 1.8 | 35.6 |
| Consensus3 | 676515497 | NW_008710536.1 | Lottia gigantea | 91.304 | 23 | 2 | 0 | 39 | 61 | 366549 | 366527 | 6.3 | 33.7 |
| Consensus3 | 676517172 | NW_008710950.1 | Lottia gigantea | 95.455 | 22 | 1 | 0 | 17 | 38 | 1637439 | 1637418 | 1.8 | 35.6 |
| Consensus3 | 902686142 | NW_013193372.1 | Biomphalaria glabrata | 100 | 19 | 0 | 0 | 1 | 19 | 95558 | 95576 | 1.8 | 35.6 |
| Consensus3 | 902374827 | NW_013431717.1 | Biomphalaria glabrata | 86.111 | 36 | 1 | 3 | 25 | 58 | 125258 | 125291 | 6.3 | 33.7 |
| Consensus3 | 761390924 | NW_011934744.1 | Crassostrea gigas | 100 | 18 | 0 | 0 | 1 | 18 | 66547 | 66530 | 6.3 | 33.7 |
| Consensus3 | 761390783 | NW_011934885.1 | Crassostrea gigas | 100 | 18 | 0 | 0 | 26 | 43 | 715905 | 715922 | 6.3 | 33.7 |
| Consensus3 | 761390471 | NW_011935197.1 | Crassostrea gigas | 100 | 18 | 0 | 0 | 1 | 18 | 32799 | 32816 | 6.3 | 33.7 |
| Consensus3 | 761390434 | NW_011935234.1 | Crassostrea gigas | 83.784 | 37 | 4 | 2 | 1 | 37 | 47434 | 47468 | 6.3 | 33.7 |
| Consensus3 | 761390376 | NW_011935292.1 | Crassostrea gigas | 100 | 18 | 0 | 0 | 40 | 57 | 20799 | 20816 | 6.3 | 33.7 |
| Consensus3 | 761390053 | NW_011935615.1 | Crassostrea gigas | 95.238 | 21 | 1 | 0 | 25 | 45 | 112714 | 112734 | 6.3 | 33.7 |
| Consensus3 | 761389826 | NW_011935842.1 | Crassostrea gigas | 100 | 18 | 0 | 0 | 1 | 18 | 16963 | 16980 | 6.3 | 33.7 |
| Consensus3 | 761389742 | NW_011935926.1 | Crassostrea gigas | 91.304 | 23 | 2 | 0 | 29 | 51 | 119549 | 119527 | 6.3 | 33.7 |
| Consensus3 | 761389697 | NW_011935971.1 | Crassostrea gigas | 100 | 18 | 0 | 0 | 22 | 39 | 522760 | 522777 | 6.3 | 33.7 |
| Consensus3 | 761389531 | NW_011936137.1 | Crassostrea gigas | 85.714 | 28 | 4 | 0 | 33 | 60 | 32034 | 32007 | 6.3 | 33.7 |
| Consensus3 | 761389447 | NW_011936221.1 | Crassostrea gigas | 92 | 25 | 1 | 1 | 15 | 38 | 75178 | 75202 | 6.3 | 33.7 |
| Consensus3 | 761389442 | NW_011936226.1 | Crassostrea gigas | 100 | 18 | 0 | 0 | 1 | 18 | 869265 | 869282 | 6.3 | 33.7 |
| Consensus3 | 761389207 | NW_011936461.1 | Crassostrea gigas | 100 | 18 | 0 | 0 | 42 | 59 | 217938 | 217921 | 6.3 | 33.7 |
| Consensus3 | 761389165 | NW_011936503.1 | Crassostrea gigas | 88.462 | 26 | 3 | 0 | 23 | 48 | 28720 | 28745 | 6.3 | 33.7 |
| Consensus3 | 761389147 | NW_011936521.1 | Crassostrea gigas | 100 | 18 | 0 | 0 | 45 | 62 | 705011 | 705028 | 6.3 | 33.7 |
| Consensus3 | 761388936 | NW_011936732.1 | Crassostrea gigas | 100 | 18 | 0 | 0 | 1 | 18 | 339626 | 339643 | 6.3 | 33.7 |
| Consensus3 | 761388133 | NW_011937535.1 | Crassostrea gigas | 86.667 | 30 | 2 | 1 | 25 | 54 | 3860 | 3887 | 6.3 | 33.7 |
| Consensus3 | 761388068 | NW_011937600.1 | Crassostrea gigas | 100 | 18 | 0 | 0 | 1 | 18 | 205547 | 205564 | 6.3 | 33.7 |
| Consensus3 | 761388000 | NW_011937668.1 | Crassostrea gigas | 91.304 | 23 | 2 | 0 | 32 | 54 | 249714 | 249692 | 6.3 | 33.7 |
| Consensus3 | 761387864 | NW_011937804.1 | Crassostrea gigas | 100 | 18 | 0 | 0 | 22 | 39 | 194188 | 194205 | 6.3 | 33.7 |
| Consensus3 | 761387680 | NW_011937988.1 | Crassostrea gigas | 91.304 | 23 | 2 | 0 | 38 | 60 | 220370 | 220348 | 6.3 | 33.7 |
| Consensus3 | 761387634 | NW_011938034.1 | Crassostrea gigas | 82.857 | 35 | 5 | 1 | 26 | 60 | 597238 | 597205 | 6.3 | 33.7 |
| Consensus3 | 761380814 | NW_011942116.1 | Crassostrea gigas | 91.304 | 23 | 2 | 0 | 25 | 47 | 124508 | 124486 | 6.3 | 33.7 |
| Consensus3 | 761380805 | NW_011942125.1 | Crassostrea gigas | 91.304 | 23 | 2 | 0 | 33 | 55 | 78383 | 78361 | 6.3 | 33.7 |
| Consensus3 | 676506502 | NW_008706946.1 | Lottia gigantea | 95.238 | 21 | 1 | 0 | 40 | 60 | 35 | 15 | 6.3 | 33.7 |
| Consensus3 | 761390607 | NW_011935061.1 | Crassostrea gigas | 91.304 | 23 | 2 | 0 | 36 | 58 | 1093732 | 1093710 | 6.3 | 33.7 |
| Consensus3 | 761389750 | NW_011935918.1 | Crassostrea gigas | 91.304 | 23 | 2 | 0 | 37 | 59 | 172538 | 172560 | 6.3 | 33.7 |
| Consensus3 | 761389310 | NW_011936358.1 | Crassostrea gigas | 91.304 | 23 | 2 | 0 | 38 | 60 | 1242450 | 1242428 | 6.3 | 33.7 |
| Consensus3 | 761389225 | NW_011936443.1 | Crassostrea gigas | 100 | 18 | 0 | 0 | 1 | 18 | 91030 | 91013 | 6.3 | 33.7 |
| Consensus3 | 761388389 | NW_011937279.1 | Crassostrea gigas | 85.714 | 28 | 4 | 0 | 34 | 61 | 393802 | 393775 | 6.3 | 33.7 |
| Consensus3 | 761388331 | NW_011937337.1 | Crassostrea gigas | 92 | 25 | 1 | 1 | 36 | 60 | 744227 | 744204 | 6.3 | 33.7 |
| Consensus3 | 523419618 | NW_004797431.1 | Aplysia californica | 85.714 | 28 | 4 | 0 | 33 | 60 | 667336 | 667363 | 6.3 | 33.7 |
| Consensus3 | 523419597 | NW_004797452.1 | Aplysia californica | 78.947 | 38 | 8 | 0 | 24 | 61 | 795830 | 795793 | 6.3 | 33.7 |
| Consensus3 | 523419584 | NW_004797465.1 | Aplysia californica | 88.462 | 26 | 3 | 0 | 8 | 33 | 648014 | 648039 | 6.3 | 33.7 |
| Consensus3 | 676506060 | NW_008706747.1 | Lottia gigantea | 80.556 | 36 | 3 | 1 | 26 | 61 | 381809 | 381840 | 6.3 | 33.7 |
| Consensus3 | 676510462 | NW_008708555.1 | Lottia gigantea | 82.353 | 34 | 2 | 1 | 28 | 61 | 85719 | 85690 | 6.3 | 33.7 |
| Consensus3 | 676510841 | NW_008708707.1 | Lottia gigantea | 78.571 | 42 | 7 | 1 | 22 | 61 | 2144995 | 2144954 | 6.3 | 33.7 |
| Consensus3 | 676511569 | NW_008708986.1 | Lottia gigantea | 85.714 | 28 | 4 | 0 | 34 | 61 | 2039113 | 2039086 | 6.3 | 33.7 |
| Consensus3 | 676511569 | NW_008708986.1 | Lottia gigantea | 84.848 | 33 | 4 | 1 | 9 | 41 | 3939939 | 3939970 | 6.3 | 33.7 |
| Consensus3 | 676511569 | NW_008708986.1 | Lottia gigantea | 81.081 | 37 | 4 | 1 | 10 | 46 | 5756855 | 5756822 | 6.3 | 33.7 |
| Consensus3 | 676511826 | NW_008709073.1 | Lottia gigantea | 83.871 | 31 | 5 | 0 | 31 | 61 | 1763364 | 1763334 | 6.3 | 33.7 |
| Consensus3 | 676512020 | NW_008709146.1 | Lottia gigantea | 100 | 18 | 0 | 0 | 26 | 43 | 2203583 | 2203566 | 6.3 | 33.7 |
| Consensus3 | 676512203 | NW_008709220.1 | Lottia gigantea | 95.238 | 21 | 1 | 0 | 24 | 44 | 1564765 | 1564785 | 6.3 | 33.7 |
| Consensus3 | 676512203 | NW_008709220.1 | Lottia gigantea | 95.238 | 21 | 1 | 0 | 24 | 44 | 1573445 | 1573465 | 6.3 | 33.7 |
| Consensus3 | 676512380 | NW_008709295.1 | Lottia gigantea | 83.871 | 31 | 1 | 1 | 31 | 61 | 858823 | 858797 | 6.3 | 33.7 |
| Consensus3 | 676512736 | NW_008709421.1 | Lottia gigantea | 89.286 | 28 | 2 | 1 | 25 | 51 | 384301 | 384274 | 6.3 | 33.7 |
| Consensus3 | 676513035 | NW_008709540.1 | Lottia gigantea | 100 | 18 | 0 | 0 | 44 | 61 | 2068015 | 2068032 | 6.3 | 33.7 |
| Consensus3 | 676513444 | NW_008709715.1 | Lottia gigantea | 83.871 | 31 | 1 | 1 | 31 | 61 | 3177010 | 3176984 | 6.3 | 33.7 |
| Consensus3 | 676513444 | NW_008709715.1 | Lottia gigantea | 100 | 18 | 0 | 0 | 42 | 59 | 3965738 | 3965721 | 6.3 | 33.7 |

**Table L.** Most relevant TF predicted for HbII and HbIII promoters with their location relative to the TSS and function.

| **Transcription Factor** | **HbII** | **HbIII** | **Function** |
| --- | --- | --- | --- |
| RORα (retinoid-related orphan receptor- α) | -297,-316 | -276, -275 | Regulates the activity of the transcription factor hypoxia-inducible factor 1 (HIF-1), involved in cell adaptation mechanism under hypoxic conditions by up-regulating the transcription of oxygen delivery-proteins [1, 2]. |
| ROX1(hypoxic regulon) | -290 | -269 | A heme dependent expression factor that represses the expression of a group of hypoxic genes in *Saccharomyces cerevisiae* under aerobic conditions [3]. |
| NF-E2 | -293 | -286 | Involved in the regulation of globin gene transcription, found in the upstream region of the vertebrate alpha and beta globin gene clusters [4, 5]. |
| GATA-1  GATA-2 | -189, -307  -188, -306 | -164, -286  -163, -285 | TFs that bind an enhancer signal commonly found in globin genes involved in the development and function of erythroid cells [6, 7]. Regulate transcription of genes involved in the development and proliferation of hematopoietic stem cells [8]. |
| GLN3 | -306 | -285 | Member of the GATA family found in yeast, regulates nitrogen catabolic gene expression [9]. |
| Oct-1 | -286 | -265 | Member of the POU family, is important for tissue and cell-specific transcription as well as for the transcription of a number of housekeeping genes and regulation of the γ-globin gene [5, 10]. |
| MafG | -308 | -287 | Member of the Maf family, factors that can heterodimerize with other basic-zipper proteins to act as activators or repressors of gene expression and differentiation of erythroid cells [11]. May be induced after exposure to hydrogen peroxide [12]. |
| CREB | -308 | -288 | Stimulus-induced transcriptional activator in different in proliferation, differentiation, and adaptive responses [13]. CREB (cyclic AMP responsive element binding protein) plays a key role in the responses to pulmonary hypoxia [14]. |
| STAT-4 STAT-6 | -192 | -160 | Member of signal transducer and activator of transcription protein family involved in development, proliferation and immune defense [15, 16]. |
| p53 | -281 | -260 | A tumor suppressor protein that responds to cellular stress and mediates a variety of anti-proliferative processes [17]. |
| CdxA | -327, -332 | -305, -310 | The chicken orthologue to mouse Cdx1, a member of the caudal-related homeobox transcription factor gene family, involved in the regulation of patterning and development, including hematopoiesis [18, 19]. |

**Table M.** Most relevant TF predicted for HbI_SV and HbI_LV promoters with their location relative to the TSS and function.

| **Transcription Factor** | **HbI_SV** | **HbI_LV** | **Function** |
| --- | --- | --- | --- |
| SRF (serum-response factor): MCM1 | -112, -115 | NA | SRF (serum-response factor) and MCM1*,* are involved in the cellular response to extracellular signals and the cell- type-specific regulation of transcription [20]. |
| GATA-1 | -297,-640,  -738 | -181, -313 | See Table L |
| Oct-1 | -726 | -284 | See Table L |
| Oct-6 | -727 | NA | POU family member, plays a pivotal role in intracellular regulation of Schwann cells, found throughout the entire peripheral nervous system [21]. |
| NF-kappa B | -486 | -983 | Regulatory factor involved in inflammation, immunity, differentiation, cell growth, tumorigenesis and apoptosis. NF-Kappa B is regulated by H_2_S [22]. |
| AP-1 | -429 | NA | Dimeric basic region-leucine zipper complex composed of Jun, Fos or ATF subunits which regulates cell proliferation, death, survival and differentiation [23]. |
| NF-1 (Nuclear factor I) | -700 | -403 | CCAAT box-binding transcription factor (CTF) [24]. |
| C/EBP alpha (CCAAT/enhancer-binding protein alpha) | NA | -904,-580, -504 | Transcriptional activator involved in immune and inflammatory responses, can cooperate with EKLF (CACC-box binding protein) to activate the β-globin promoter [25]. |
| AhR (Aryl Hydrocarbon Receptor) | NA | -245 | Basic helix-loop-helix Per-Arnt-Sim (bHLH/PAS) superfamily member, involved in regulation of xenobiotic-metabolizing enzymes [26]. AhR is activated during adult stage β-globin gene expression [27]. |
| AP-4 | NA | -742 | A transcription factor that activates both viral and cellular genes [28]. |
| CREB | NA | -54 | See Table L. |

References

1. Kim EJ, Yoo YG, Yang WK, Lim YS, Na TY, Lee IK, et al.: **Transcriptional activation of HIF-1 by RORα and its role in hypoxia signaling**. *Arteriosclerosis, Thrombosis, and Vascular Biology* 2008:1796–1802.

2. Jolly S, Journiac N, Naudet F, Gautheron V, Mariani J, et al.: **Cell-Autonomous and Non-Cell-Autonomous Neuroprotective Functions of ROR  in Neurons and Astrocytes during Hypoxia**. *Journal of Neuroscience* 2011:14314–14323.

3. Balasubramanian B, Lowry C V, Zitomer RS: **The Rox1 repressor of the Saccharomyces cerevisiae hypoxic genes is a specific DNA-binding protein with a high-mobility-group motif.** *Mol Cell Biol* 1993, **13**:6071–6078.

4. Andrews N: **The NF-E2 transcription factor**. *Int J Biochem Cell Biol* 1998, **30**:429–432.

5. Hardison R: **Hemoglobins from Bacteria to Man: Evolution of Different Patterns Of Gene Expression**. *J Exp Biol* 1998, **1117**:1099–1117.

6. Katsumura KR, Devilbiss AW, Pope NJ, Johnson KD, Bresnick EH: **Transcriptional Mechanisms Underlying Hemoglobin Synthesis**. *Cold Spring Harb Perspect Med* 2013, **3**:1–20.

7. Forget BG, Hardison RC: **The Normal Structure and Regulation of Human Globin Gene Clusters**. In *Disorders of Hemoglobins: Genetics, Pathophysiology, and Clinical Management*. 2nd edition.; 2009:46–61.

8. Nakajima T, Kitagawa K, Ohhata T, Sakai S, Uchida C, Shibata K, et al.: **Regulation of GATA binding protein 2 levels via ubiquitin-dependent degradation by Fbw7: involvement of cyclin B-cyclin-dependent kinase 1-mediated phosphorylation of Thr176 in GATA binding protein 2**. *J Biol Chem* 2015, **290**:10368–10381.

9. Kulkarni AA, Abul-Hamd AT, Rai R, El Berry H, Cooper TG: **Gln3p Nuclear Localization and Interaction with Ure2p in Saccharomyces cerevisiae**. *J Biol Chem* 2001, **276**:32136–32144.

10. Xu XS, Hong X, Wang G: **Induction of endogenous gamma-globin gene expression with decoy oligonucleotide targeting Oct-1 transcription factor consensus sequence.** *Journal of hematology & oncology* 2009:15.

11. Kataoka K, Igarashi K, Itoh K, Fujiwara KT, Noda M, Yamamoto M, et al.: **Small Maf proteins heterodimerize with Fos and may act as competitive repressors of the NF-E2 transcription factor.** *Mol Cell Biol* 1995, **15**:2180–2190.

12. Crawford DR, Leahy KP, Wang Y, Schools GP, Kochheiser JC, Davies KJA: **Oxidative stress induces the levels of a MafG homolog in hamster HA-1 cells**. *Free Radic Biol Med* 1996, **21**:521–525.

13. Shaywitz AJ, Greenberg ME: **CREB: A Stimulus -Induced Transcription Factor Activated by a Diverse Array of Extracellular Signals**. *Annu Rev Biochem* 1999, **68**:821–861.

14. Leonard MO, Howell K, Madden SF, Costello CM, Higgins DG, Taylor CT, et al.: **Hypoxia selectively activates the CREB family of transcription factors in the In vivo lung**. *Am J Respir Crit Care Med* 2008, **178**:977–983.

15. Liang Y, Pan H-F, Ye D-Q: **Therapeutic potential of STAT4 in autoimmunity.** *Expert Opin Ther Targets* 2014, **18**:1–16.

16. Litterst CM, Pfitzner E: **Transcriptional Activation by STAT6 Requires the Direct Interaction with NCoA-1**. *J Biol Chem* 2001, **276**:45713–45721.

17. Fridman JS, Lowe SW: **Control of apoptosis by p53.** *Oncogene* 2003, **22**:9030–9040.

18. Gaunt SJ, Drage D, Cockley A: **Vertebrate caudal gene expression gradients investigated by use of chick cdx-A/lacZ and mouse cdx-1/lacZ reporters in transgenic mouse embryos: Evidence for an intron enhancer**. *Mech Dev* 2003, **120**:573–586.

19. Béland M, Pilon N, Houle M, Oh K, Sylvestre J-R, Prinos P, et al.: **Cdx1 autoregulation is governed by a novel Cdx1-LEF1 transcription complex.** *Mol Cell Biol* 2004, **24**:5028–5038.

20. Treisman R, Ammerer G: **The SRF and MCM1 transcription factors.** *Curr Opin Genet Dev* 1992, **2**:221–226.

21. Jaegle M, Meijer D: **Role of Oct-6 in Schwann cell differentiation**. *Microsc Res Tech* 1998, **41**:372–378.

22. Sen N, Paul BD, Gadalla MM, Mustafa AK, Sen T, Xu R, et al.: **Hydrogen sulfide-linked sulfhydration of NF-κB mediates its antiapoptotic actions**. *Molecular Cell* 2012:13–24.

23. Shaulian E, Karin M: **AP-1 as a regulator of cell life and death.** *Nat Cell Biol* 2002, **4**:E131–136.

24. Mermod N, O’Neill EA, Kelly TJ, Tjian R: **The proline-rich transcriptional activator of CTF/NF-I is distinct from the replication and DNA binding domain.** *Cell* 1989, **58**:741–753.

25. Gordon CT, Fox VJ, Najdovska S, Perkins AC: **C/EBPδ and C/EBPγ bind the CCAAT-box in the human β-globin promoter and modulate the activity of the CACC-box binding protein, EKLF**. *Biochim Biophys Acta - Gene Struct Expr* 2005, **1729**:74–80.

26. Tian J, Feng Y, Fu H, Xie HQ, Jiang JX, Zhao B: **The Aryl Hydrocarbon Receptor: A Key Bridging Molecule of External and Internal Chemical Signals**. *Environ Sci Technol* 2015, **49**:9518–9531.

27. Li B, Ding L, Li W, Story MD, Pace BS: **Characterization of the transcriptome profiles related to globin gene switching during in vitro erythroid maturation**. *BMC Genomics* 2012, **13**:153.

28. Hu YF, Lüscher B, Adinon A, Mermod N, Tjian R: **Transcription factor AP-4 contains multiple dimerization domains that regulate dimer specificity**. *Genes Dev* 1990, **4**:1741–1752.
